# Association of Potentially Damaging De Novo Gene Variants With Neurologic Outcomes in Congenital Heart Disease

**DOI:** 10.1001/jamanetworkopen.2022.53191

**Published:** 2023-01-26

**Authors:** Sarah U. Morton, Ami Norris-Brilliant, Sean Cunningham, Eileen King, Elizabeth Goldmuntz, Martina Brueckner, Thomas A. Miller, Nina H. Thomas, Chunyan Liu, Heather R. Adams, David C. Bellinger, John Cleveland, James F. Cnota, Anders M. Dale, Michele Frommelt, Bruce D. Gelb, P. Ellen Grant, Caren S. Goldberg, Hao Huang, Joshua M. Kuperman, Jennifer S. Li, Patrick S. McQuillen, Ashok Panigrahy, George A. Porter, Amy E. Roberts, Mark W. Russell, Christine E. Seidman, Madalina E. Tivarus, Evdokia Anagnoustou, Donald J. Hagler, Wendy K. Chung, Jane W. Newburger

**Affiliations:** 1 Division of Newborn Medicine, Department of Pediatrics, Boston Children’s Hospital, Boston, Massachusetts; 2 Fetal Neonatal Neuroimaging and Developmental Science Center, Boston Children’s Hospital, Boston, Massachusetts; 3 Department of Pediatrics, Harvard Medical School, Boston, Massachusetts; 4 Department of Genetics, Harvard Medical School, Boston, Massachusetts; 5 Department of Psychiatry, Icahn School of Medicine at Mount Sinai, New York, New York; 6 Department of Pediatrics, Division of General Pediatrics, University of Utah, Salt Lake City; 7 Department of Pediatrics, University of Cincinnati, Cincinnati, Ohio; 8 Division of Biostatistics and Epidemiology, Cincinnati Children’s Hospital Medical Center, Cincinnati, Ohio; 9 Division of Cardiology, Children’s Hospital of Philadelphia, Pennsylvania; 10 Department of Pediatrics, Perelman School of Medicine, University of Pennsylvania, Philadelphia; 11 Departments of Genetics and Pediatrics, Yale University School of Medicine, New Haven, Connecticut; 12 Department of Pediatrics, Primary Children’s Hospital, University of Utah, Salt Lake City; 13 Division of Pediatric Cardiology, Maine Medical Center, Portland; 14 Department of Child and Adolescent Psychiatry and Behavioral Sciences and Center for Human Phenomic Science, Children’s Hospital of Philadelphia, Philadelphia, Pennsylvania; 15 Department of Psychiatry, University of Pennsylvania, Philadelphia; 16 Departments of Neurology and Pediatrics, University of Rochester Medical Center, Rochester, New York; 17 Departments of Neurology and Psychiatry, Boston Children’s Hospital, Boston, Massachusetts; 18 Departments of Neurology and Psychiatry, Harvard Medical School, Boston, Massachusetts; 19 Departments of Surgery and Pediatrics, Keck School of Medicine, University of Southern California, Los Angeles; 20 Heart Institute, Cincinnati Children’s Hospital Medical Center, Cincinnati, Ohio; 21 Center for Multimodal Imaging and Genetics, University of California San Diego, La Jolla; 22 Department of Radiology, School of Medicine, University of California San Diego, La Jolla; 23 Departments of Cognitive Science and Neuroscience, University of California San Diego, La Jolla; 24 Department of Pediatrics, Medical College of Wisconsin, Milwaukee; 25 Mindich Child Health and Development Institute and Department of Pediatrics, Icahn School of Medicine at Mount Sinai, New York, New York; 26 Department of Radiology, Boston Children’s Hospital, Boston, Massachusetts; 27 Department of Radiology, Harvard Medical School, Boston, Massachusetts; 28 Department of Pediatrics, C.S. Mott Children’s Hospital, University of Michigan, Ann Arbor; 29 Department of Radiology, Children’s Hospital of Philadelphia, University of Pennsylvania, Philadelphia; 30 Division of Pediatric Cardiology, Duke University, Durham, North Carolina; 31 Departments of Pediatrics and Neurology, University of California, San Francisco; 32 Department of Pediatric Radiology, Children’s Hospital of Pittsburgh, University of Pittsburgh Medical Center, Pittsburgh, Pennsylvania; 33 Department of Cardiology, Boston Children’s Hospital, Boston, Massachusetts; 34 Division of Genetics and Genomics, Boston Children’s Hospital, Boston, Massachusetts; 35 Cardiovascular Division, Brigham and Women’s Hospital, Boston, Massachusetts; 36 Howard Hughes Medical Institute, Chevy Chase, Maryland; 37 Departments of Imaging Sciences and Neuroscience, University of Rochester Medical Center, Rochester, New York; 38 Department of Pediatrics, University of Toronto, Holland Bloorview Kids Rehabilitation Hospital, Toronto, Ontario, Canada; 39 Departments of Pediatrics and Medicine, Columbia University Medical Center, New York, New York

## Abstract

**Importance:**

Neurodevelopmental disabilities are commonly associated with congenital heart disease (CHD), but medical and sociodemographic factors explain only one-third of the variance in outcomes.

**Objective:**

To examine whether potentially damaging de novo variants (dDNVs) in genes not previously linked to neurodevelopmental disability are associated with neurologic outcomes in CHD and, post hoc, whether some dDNVs or rare putative loss-of-function variants (pLOFs) in specific gene categories are associated with outcomes.

**Design, Setting, and Participants:**

This cross-sectional study was conducted from September 2017 to June 2020 in 8 US centers. Inclusion criteria were CHD, age 8 years or older, and available exome sequencing data. Individuals with pathogenic gene variants in known CHD- or neurodevelopment-related genes were excluded. Cases and controls were frequency-matched for CHD class, age group, and sex.

**Exposures:**

Heterozygous for (cases) or lacking (controls) dDNVs in genes not previously associated with neurodevelopmental disability. Participants were separately stratified as heterozygous or not heterozygous for dDNVs and/or pLOFs in 4 gene categories: chromatin modifying, constrained, high level of brain expression, and neurodevelopmental risk.

**Main Outcomes and Measures:**

Main outcomes were neurodevelopmental assessments of academic achievement, intelligence, fine motor skills, executive function, attention, memory, social cognition, language, adaptive functioning, and anxiety and depression, as well as 7 structural, diffusion, and functional brain magnetic resonance imaging metrics.

**Results:**

The study cohort included 221 participants in the post hoc analysis and 219 in the case-control analysis (109 cases [49.8%] and 110 controls [50.2%]). Of those 219 participants (median age, 15.0 years [IQR, 10.0-21.2 years]), 120 (54.8%) were male. Cases and controls had similar primary outcomes (reading composite, spelling, and math computation on the Wide Range Achievement Test, Fourth Edition) and secondary outcomes. dDNVs and/or pLOFs in chromatin-modifying genes were associated with lower mean (SD) verbal comprehension index scores (91.4 [20.4] vs 103.4 [17.8]; *P* = .01), Social Responsiveness Scale, Second Edition, scores (57.3 [17.2] vs 49.4 [11.2]; *P* = .03), and Wechsler Adult Intelligence Scale, Fourth Edition, working memory scores (73.8 [16.4] vs 97.2 [15.7]; *P* = .03), as well as higher likelihood of autism spectrum disorder (28.6% vs 5.2%; *P* = .01). dDNVs and/or pLOFs in constrained genes were associated with lower mean (SD) scores on the Wide Range Assessment of Memory and Learning, Second Edition (immediate story memory: 9.7 [3.7] vs 10.7 [3.0]; *P* = .03; immediate picture memory: 7.8 [3.1] vs 9.0 [2.9]; *P* = .008). Adults with dDNVs and/or pLOFs in genes with a high level of brain expression had greater Conners adult attention-deficit hyperactivity disorder rating scale scores (mean [SD], 55.5 [15.4] vs 46.6 [12.3]; *P* = .007).

**Conclusions and Relevance:**

The study findings suggest neurodevelopmental outcomes are not associated with dDNVs as a group but may be worse in individuals with dDNVs and/or pLOFs in some gene sets, such as chromatin-modifying genes. Future studies should confirm the importance of specific gene variants to brain function and structure.

## Key Points

**Question:**

Are damaging de novo variants in genes not previously associated with neurodevelopmental risk (dDNV-NR) associated with worse neurologic findings in individuals with congenital heart disease (CHD)?

**Findings:**

In this cross-sectional study of 221 patients with CHD, dDNV-NRs as a group were not associated with neurologic outcomes. In post hoc analyses, dDNVs and rare putative loss-of-function (pLOF) variants, especially in chromatin-modifying genes, were associated with worse neurodevelopmental and brain magnetic resonance imaging (MRI) metrics.

**Meaning:**

In this study, worse neurodevelopmental and brain MRI outcomes were not associated with dDNV-NRs overall; the association of neurodevelopmental impairments with subgroups of gene variants based on function or expression should be assessed in larger studies.

## Teaser

This cross-sectional study investigates whether there are associations between potentially damaging de novo variants in genes not previously associated with neurodevelopmental risk and neurologic outcomes among individuals with congenital heart disease in the US.

## Introduction

Neurodevelopmental disabilities (NDDs) are common among patients with congenital heart disease (CHD).^1,2^ The growing population of children and adults with CHD has exposed the high prevalence of neuropsychologic impairment in this population.^3,4^ Specifically, individuals with CHD demonstrate greater risk of impaired reasoning, learning, executive function, language skills, and social skills compared with peers without CHD.^5,6,7,8,9^ Impairments in these domains may lead to poor school performance, strained interpersonal relationships, and behavior problems. Children with CHD are more likely to require remedial services, including tutoring and special education, as well as physical, occupational, and speech therapy.^1^ As these children reach adulthood, NDDs can limit educational achievement, employability, and quality of life.^10,11^ Known risk factors such as family socioeconomic status, prematurity, prolonged intensive care unit stay, and cardiac complexity only explain approximately 30% of observed variation in outcomes after cardiac surgery in infancy,^12^ suggesting that as-yet-undescribed genetic and epigenetic factors may play an important role.^13^

Genetic variants are associated with the risk of CHD.^14,15^ Recent studies suggest that many patients with CHD have de novo variants associated with damage to gene function (dDNVs), but they are in genes not previously associated with CHD.^16,17,18,19^ An excess burden of dDNVs is found in patients with CHD in combination with both NDDs and extracardiac anomalies compared with those with CHD alone (28% vs 3%, respectively).^16,17,18,19^ Many of the genes with heterozygous variants are in constrained genes, are highly expressed during fetal brain development, and have pleiotropic effects. Genes with dDNVs in patients with CHD and NDDs were enriched for functions related to chromatin modification or have a known association with NDDs.^16^ Further, dDNVs in autism-associated genes have been identified in patients with CHD, emphasizing the overlap in the developmental processes directing cardiac and neural development.^17^ However, the degree to which all dDNVs contribute to NDD risk among patients with CHD is unknown. We hypothesized that these dDNVs in putative disease-related genes contribute jointly to the risk of NDDs and the risk for CHD through these pleiotropic effects.^20^

We sought to compare neurodevelopmental, behavioral, and brain magnetic resonance imaging (MRI) measures in individuals with CHD who had dDNVs in genes not previously associated with neurodevelopmental risk (dDNV-NRs) compared with those without such variants. The study population was drawn from a registry of individuals in parent-proband trios in the Pediatric Cardiac Genomics Consortium (PCGC)^21^ or the Pediatric Heart Network’s Single Ventricle Reconstruction (SVR)^22^ study. Patients with pathogenic single nucleotide variants in genes already associated with NDDs or pathogenic copy number variants (CNVs) were excluded from the study. We assessed the association of dDNVs and rare putative loss-of-function variants (pLOFs) with neurodevelopmental measures of academic achievement (primary outcome), intelligence, executive functions, visual-spatial skills, memory, attention, language, adaptive function, social cognition, and mental health as well as brain MRI metrics.

## Methods

The Genomic Basis of Neurodevelopmental and Brain Outcomes in Congenital Heart Disease was a multicenter cross-sectional study used to test the association of dDNVs with neurodevelopmental and brain MRI outcomes in individuals with CHD. The study protocol was conceived and developed by investigators of the PCGC and was approved by the institutional review board of each participating site and the administrative coordinating center. Reliance agreements were approved at each study center. Written informed consent was obtained from participants or their parents; written informed assent was obtained from competent older children. Study oversight was provided by an independent data monitoring committee appointed by the National Heart, Lung, and Blood Institute. This study followed the Strengthening the Reporting of Observational Studies in Epidemiology (STROBE) reporting guideline^23^ and was performed from September 2017 to June 2020 in 8 US centers.

All participants had been previously enrolled in the PCGC^21^ (N = 197) or SVR^22^ trial (N = 24) (eFigure 1 in Supplement 1). Inclusion criteria included diagnosis of CHD, age 8 years or older, available DNA sequencing data, heterozygous for a dDNV-NR (cases) or absence of such variants (controls), and informed consent or assent. Controls were frequency matched with cases based on CHD categories (1 or 2 ventricles; with or without arch obstruction), age category (8-12 years, 13-17 years, or >17 years), and natal sex. Cases drawn from the SVR cohort were matched with controls from within the SVR cohort. We excluded individuals with cardiac transplant, cardiac surgical procedure within 6 months of enrollment, a known monogenic condition associated with abnormalities of the brain or heart as of April 2017, presence of pathogenic CNV, severe acquired brain injury that would overshadow the associations of a genetic variation with outcomes in the opinion of the center investigator, or inability to communicate in English or Spanish. Presence of pathogenic CNVs was determined using previously published analyses.^24^ Exclusion criteria for brain MRI included contraindication for having a brain MRI scan, claustrophobia or inability to lie still while in the MRI scanner for the required time (sedation not allowed), or pregnancy. Magnetic resonance imaging and neuropsychologic testing could occur over 2 days, with a maximal interval of 6 months between tests. All participants were included in post hoc analyses and all but 2 in case-control analysis.

Race and ethnicity were assessed by self-report and were included in the analysis because race and ethnicity have been associated with differences in outcomes among patients with CHD. Race included Asian, Black or African American, White, and more than 1 race. Ethnicity included Hispanic/Latino and not Hispanic/Latino.

We analyzed neurodevelopmental and brain MRI outcomes according to the presence or absence of each of the possible variant classes: (1) dDNV-NR, (2) dDNV and/or pLOF in genes with a high level of brain expression (HBE), (3) dDNV and/or pLOF in NDD, (4) dDNV and/or pLOF in chromatin-modifying genes, or (5) dDNV and/or pLOF in genes with a pLI score greater than 0.5 (eMethods and eTable 1 in Supplement 1). The same genetic variant may meet the criterion for more than 1 of these categories (eTable 2 and eFigure 2 in Supplement 1). As each of the 5 groups were analyzed independently, correlation between the groups was not considered.

### Study Outcomes

Neurodevelopmental assessments were performed by a licensed psychologist or supervised psychometrician. Standardized instruments were used to assess the domains of academic achievement, intelligence, fine motor function, executive function, attention, memory, social cognition, language, adaptive functioning, anxiety, and depression (eMethods and eTable 3 in Supplement 1).

Acquisition and processing of MRI data closely followed the approach of the Adolescent Brain Cognitive Development Study.^25^ Details are available in the eMethods in Supplement 1. MRI was performed on 3T scanners, either Prisma (Siemens Healthineers; 90 participants, 6 sites) or MR750 (General Electric; 10 participants, 1 site). Scan sessions included volumetric T1-weighted and T2-weighted structural MRI sequences, 1 diffusion MRI sequence, and 4 repetitions of a resting-state functional MRI (rs-fMRI) sequence. Clinical imaging variables determined by a neuroradiology core reader included metrics of acquired brain injury, namely number of focal infarcts, number of foci with high or low T2 signal, and presence of other abnormalities. Seven quantitative global MRI metrics with at least 1 from each MRI modality were calculated for each participant. MRI measures included in this primary analysis were 4 structural measures (whole brain volume, mean cortical thickness, cortical surface area, and ventricular volume), 2 diffusion measures (mean white matter diffusivity, mean white matter directionality), and 1 functional connectivity measure (mean within-network correlation of the default mode network during rs-fMRI).

### Statistical Analysis

The study was designed to compare mean achievement scores in participants with and without dDNV-NRs using 2-sided, 2-sample tests conducted at the 0.05 level of significance. The SD of achievement scores was assumed to be 15 in each group. If the true effect size was 0.4 SDs, samples of 100 participants in each group would provide 80% power to detect a mean difference of 6.0 points (ie, 0.4 SD).

Demographic and clinical characteristics of participants were compared according to their case-control status and to the presence or absence of rare variants using the Wilcoxon rank sum test for continuous variables and the exact χ^2^ test for categorical variables.

The primary outcomes were reading composite, spelling, and math computation based on the Wide Range Achievement Test, Fourth Edition (WRAT-4). Secondary outcomes included other measures that assessed intelligence, academic achievement, neurodevelopment, and behavioral health as well as brain MRI metrics. Some neurocognitive outcomes were designed for specific age ranges so could be measured in only a subset of participants.

Mixed-effects multivariable models were used for each ND or MRI outcome, with the testing site as the random effect. Small testing sites with fewer than 6 participants were grouped together. In addition to the genetic grouping variable, the variables on which cases and controls were frequency matched (CHD category, age, and sex) were included as stratification variables (ie, covariates) in analyses of ND and MRI outcomes. Maternal educational level was also included as an additional covariate in models of ND outcomes.

For MRI outcomes, mixed-effects multivariable models were additionally adjusted for scanner type (General Electric vs Siemens). Mean motion was included as an additional covariate in analyses of rs-fMRI.

We used SAS, version 9.4 (SAS institute) and R, version 4.0.2 (R Project for Statistical Computing) for the statistical analyses unless otherwise specified, and statistical significance was defined as 2-sided *P* < .05. In this hypothesis-generating study, we did not adjust for multiple testing.

## Results

### Study Population

The study cohort included 221 participants in the post hoc analysis and 219 in the case-control analysis (109 cases [49.8%] and 110 controls [50.2%]), with 2 controls included only in rare-variant analysis. Of the 219 participants, 99 (45.2%) were female and 120 (54.8%) were male. The median age was 15.0 years (IQR, 10.0-21.2 years) (Table 1); almost one-third of participants (69 [31.5%]) were older than 18 years. For race, 3 of 190 (1.6%) were Asian; 2 of 190 (1.1%), Black or African American; 179 of 190 (94.2%), White; and 6 of 190 (3.2%), more than 1 race. For ethnicity, 20 of 190 (10.5%) were Hispanic/Latino and 170 of 190 (89.5%) were not Hispanic/Latino. The majority (123 [56.2%]) had biventricular CHD without arch obstruction (Table 1). The median number of cardiac surgeries was 3.0 (IQR, 1.0-3.0), and approximately half of the participants (84 [49.4%]) had their first cardiac operation in the neonatal period. The highest maternal educational level was high school for 31 participants (14.2%), some college or college for 127 (58.0%), and a postgraduate degree for 61 (27.9%).

**Table 1.** Participant Characteristics

Characteristic	Participants^a^		*P* value^b^
	Case (n = 109)	Control (n = 110)	
Age group at enrollment, y^c^			
8-12	55 (50.5)	57 (51.8)	.98
13-17	19 (17.4)	19 (17.3)	
≥18	35 (32.1)	34 (30.9)	
Sex^c^			
Female	54 (49.5)	45 (40.9)	.22
Male	55 (50.5)	65 (59.1)	
CHD diagnosis^c^			
Biventricle without arch obstruction	62 (56.9)	61 (55.5)	.61
Biventricle with arch obstruction	9 (8.3)	6 (5.5)	
Single ventricle without arch obstruction	18 (16.5)	25 (22.7)	
Single ventricle with arch obstruction	20 (18.3)	18 (16.4)	
Fyler code^c^			
Tetralogy of Fallot	17 (15.6)	11 (10.0)	NA
Double outlet right ventricle	5 (4.6)	10 (9.1)	
Atrial septal defect–secundum	22 (20.2)	29 (26.4)	
Hypoplastic left heart syndrome	8 (7.3)	5 (4.5)	
Coarctation of the aorta	11 (10.1)	9 (8.2)	
D-transposition of the great arteries	7 (6.4)	13 (11.8)	
Isolated ventricular septal defect	31 (28.4)	26 (23.6)	
Atrioventricular canal	6 (5.5)	12 (10.9)	
Heterotaxy findings	10 (9.2)	17 (15.5)	
Race, No./total No. (%)^d^^,^^e^			
Asian	0	3/95 (3.2)	.41
Black or African American	1/95 (1.1)	1/95 (1.1)	
White	91/95 (95.8)	88/95 (92.6)	
>1 Race	3/95 (3.2)	3/95 (3.2)	
Ethnicity, No./total No. (%)^d^^,^^e^			
Hispanic/Latino	11/95 (11.6)	9/95 (9.5)	.81
Not Hispanic/Latino	84/95 (88.4)	86/95 (90.5)	
Mother’s highest educational level^c^			
High school or less or other	14 (12.8)	17 (15.5)	.37
College or some college	60 (55.0)	67 (60.9)	
Postgraduate degree	35 (32.1)	26 (23.6)	
Birth weight^c^			
Participants, No.	106	104	NA
Range, kg	1.2-4.7	1.2-4.4	.24
Median (IQR), kg	3.1 (2.8-3.5)	3.3 (2.9-3.6)	
Gestational age^c^			
Participants, No.	106	100	NA
Range, wk	29.0-42.0	27.7-43.1	.87
Median (IQR), wk	39.0 (37.6-40.0)	39.0 (38.0-40.0)	
Total cardiac catheterizations, No.^c^			
Participants	108	107	NA
Range	0.0-30.0	0.0-16.0	.94
Median (IQR)	2.0 (0.0-4.0)	2.0 (0.0-4.0)	
Total cardiac surgeries, No.^c^			
Participants	109	110	NA
Range	0.0-8.0	0.0-11.0	.73
Median (IQR)	2.0 (1.0-3.0)	3.0 (1.0-3.0)	
Total open cardiac surgeries, No.^d^			
Participants	97	98	NA
Range	0.0-6.0	0.0-6.	.75
Median (IQR)	2.0 (1.0-3.0)	2.0 (1.0-3.0)	
Age at first cardiac surgery^d^^,^^f^			
Participants, No.	84	86	NA
Range, d	0 to 12 530	−6 to 9416	.46
Median (IQR), d	58.0 (0.0-946.0)	14.5 (0.0-694.0)	
Age >30 d at first cardiac surgery, No./total No. (%)^d^			
No	39/84 (46.4)	47/86 (54.7)	.36
Yes	45/84 (53.6)	39/86 (45.3)	
Ever received ECMO^c^			
No	106 (97.2)	106 (96.4)	.68
Yes	2 (1.8)	4 (3.6)	
Unknown	1 (0.9)	0	
Seizures complication, No./total No. (%)^d^			
No	96/97 (99.0)	97/98 (99.0)	>.99
Yes	1/97 (1.0)	1/98 (1.0)	
No stroke complication, No./total No. (%)^d^	97/97 (100)	98/98 (100)	NA
In-hospital cardiac arrest^d^			
No	95 (87.2)	91 (82.7)	.28
Yes	2 (1.8)	6 (5.5)	
Unknown	13 (11.8)	1 (0.9)	
Out-of-hospital cardiac arrest^d^			
No	95 (87.2)	97 (88.2)	.50
Yes	2 (1.8)	0	
Unknown	12 (11.0)	13 (11.8)	
MRI manufacturer, No./total No. (%)^d^			
GE medical systems	3 (2.8)	1 (0.9)	NA
Siemens	44 (40.4)	46 (41.8)	
No MRI	62 (56.9)	63 (57.3)	

Abbreviations: CHD, congenital heart disease; ECMO, extracorporeal membrane oxygenation; GE, General Electric; MRI, magnetic resonance imaging; NA, not applicable.

^a^ Data are presented as the number (percentage) of participants unless otherwise indicated.

^b^ The Wilcoxon rank sum test was used for continuous variables and the Fisher exact test for categorical variables (the “unknown,” “don’t know or refused,” and “other, don’t know or refused” categories were excluded for the test).

^c^ Variable data were available in both the Pediatric Cardiac Genomics Consortium and Pediatric Heart Network Single Ventricle Reconstruction cohorts.

^d^ Variable data were available in the Pediatric Cardiac Genomics Consortium cohort only.

^e^ Race and ethnicity were assessed by self-report and were included in the analysis because race and ethnicity have been associated with differences in outcomes among patients with CHD.

^f^ Negative values reflect prenatal intervention.

### Primary Outcome

Table 2^26,27,28,29,30^ provides neurocognitive and behavioral scores according to case or control status. For most measures, mean scores of both cases and controls were similar to population norms. Compared with controls, cases did not have significantly different performance in the primary outcomes measures of math (median score, 97.5 [IQR, 88.0-108.0] vs 100.0 [IQR, 90.0-111.0]; *P* = .67), reading (median score, 108.0 [IQR, 96.0-117.0] vs 103.0 [IQR, 92.5-112.0]; *P* = .16), and spelling (median score, 106.0 [IQR, 95.0-116.0] vs 102.0 [IQR, 92.0-110.0]; *P* = .11) domains assessed by WRAT-4 composite scores. Scores of cases and controls also did not differ significantly on measures of intelligence, fine motor function, executive function, attention, memory, social cognition, language, adaptive functioning, and anxiety and depression (Table 2 and eTable 4 in Supplement 1).

**Table 2.** Neurodevelopmental Outcomes by Case or Control Status

Neurodevelopmental test^a^	Cases	Controls	*P* value from mixed model
**Academics**			
Reading composite, WRAT-4 (≥8 y)^b^			
No.	95	96	NA
Score, median (IQR)	108.0 (96.0-117.0)	103.0 (92.5-112.0)	.16
Spelling, WRAT-4 (≥8 y)^b^			
No.	95	96	NA
Score, median (IQR)	106.0 (95.0-116.0)	102.0 (92.0-110.0)	.11
Math computation, WRAT-4 (≥8 y)^b^			
No.	104	107	NA
Score, median (IQR)	97.5 (88.0-108.0)	100.0 (90.0-111.0)	.67
Learning problems, Conners-3 Parent (<18 y)^c^			
No.	56	59	NA
Score, median (IQR)	51.0 (42.0-68.0)	53.0 (44.0-64.0)	.19
Learning problems, Conners-3 Teacher (<18 y)^c^			
No.	30	43	NA
Score, median (IQR)	49.0 (45.0-63.0)	51.0 (43.0-67.0)	.26
**Intelligence**			
Full-Scale IQ All Ages Standard Score, WISC-V (<16 y) or WAIS-IV (≥16 y)^b^			
No.	104	107	NA
Score, median (IQR)	99.0 (87.5-109.5)	98.0 (83.0-108.0)	.37
Verbal Comprehension Index All Ages Standard Score, WISC-V (<16 y) or WAIS-IV (≥16 y)^b^			
No.	108	109	NA
Score, median (IQR)	103.0 (95.5-114.0)	100.0 (89.0-116.0)	.28
Fluid Reasoning Index, WISC-V (<16 y)^b^			
No.	63	63	NA
Score, median (IQR)	100.0 (85.0-115.0)	97.0 (85.0-106.0)	.46
Perceptual Reasoning Index, WAIS-IV (≥16 y)^b^			
No.	43	44	NA
Score, median (IQR)	98.0 (88.0-105.0)	98.0 (88.0-113.0)	.80
**Executive function**			
Global Executive Composite, BRIEF-2 Parent (<18 y)^c^			
No.	69	70	NA
Score, median (IQR)	53.0 (46.0-63.0)	55.0 (45.0-63.0)	.98
Global Executive Composite, BRIEF-2 Teacher (<18 y)^c^			
No.	41	49	NA
Score, median (IQR)	50.0 (46.0-63.0)	52.0 (47.0-65.0)	.56
Tower Total Achievement Score, D-KEFS (≥8 y)^b^			
No.	103	106	NA
Score, median (IQR)	9.0 (8.0-11.0)	10.0 (8.0-11.0)	.74
**Memory**			
Delayed recall: story memory recall^b^			
No.	108	110	NA
Score, median (IQR)	11.0 (9.0-12.0)	10.0 (9.0-12.0)	.21
Delayed recall: picture memory recognition^b^			
No.	104	107	NA
Score, median (IQR)	10.0 (7.5-12.0)	9.0 (8.0-12.0)	.98
Delayed recall: story recognition^b^			
No.	96	97	NA
Score, median (IQR)	11.0 (9.0-13.0)	11.0 (10.0-13.0)	.80
**Social responsiveness**			
Positive for ASD, ADOS-2 (≥8 y)^b^			
No, No. (%)	4 (36.4)	6 (54.5)	NA
Yes, No. (%)	7 (63.6)	5 (45.5)	NA
SRS-2 total score (≥8 y)^c^			
No.	99	100	NA
Score, median (IQR)	48.0 (42.0-56.0)	46.5 (42.0-53.5)	.17
**Language deficits**			
Listening comprehension, WIAT-III (≥8 y)^b^			
No.	93	94	NA
Score, median (IQR)	105.0 (96.0-115.0)	105.0 (95.0-116.0)	.55
Oral expression, WIAT-III (≥8 y)^b^			
No.	94	93	NA
Score, median (IQR)	106.0 (94.0-114.0)	103.0 (93.0-114.0)	>.99
**Fine motor skills**			
VMI-6 (≥8 y)^b^			
No.	104	106	NA
Score, median (IQR)	85.0 (75.0-98.0)	87.5 (76.0-96.0)	.91
**Adaptive functioning**			
Adaptive behavior composite, Vineland-3 (≥8 y)^b^			
No.	78	82	NA
Score, median (IQR)	103.0 (92.0-112.0)	104.0 (92.0-110.0)	.97
**Anxiety and depression**			
T-score, MASC-2 (<18 y)^c^			
No.	51	56	NA
Score, median (IQR)	51.0 (46.0-61.0)	55.0 (47.5-60.0)	.91
T-score, CDI-2 (<18 y)^c^			
No.	53	59	NA
Score, median (IQR)	47.0 (42.0-54.0)	50.0 (43.0-59.0)	.94
Total raw score, BAI (≥18 y)^c^			
No.	37	35	
Score, median (IQR)	8.0 (5.0-17.0)	7.0 (2.0-10.0)	.14
Total raw score, BDI-II (≥18 y)^c^			
No.	37	35	NA
Score, median (IQR)	5.0 (3.0-15.0)	6.0 (3.0-10.0)	.20

Abbreviations: ADOS-2, Autism Diagnostic Observation Schedule, Second Edition; ASD, autism spectrum disorder; BAI, Beck Anxiety Inventory; BDI-II, Beck Depression Inventory, Second Edition; BRIEF-2, Behavior Rating Inventory of Executive Function, Second Edition; CDI-2, Children’s Depression Index, Second Edition; D-KEFS, Delis-Kaplan Executive Function System; MASC-2, Multidimensional Anxiety Scale for Children, Second Edition; NA, not applicable; SRS-2, Social Responsiveness Scale, Second Edition; Vineland-3, Vineland Adaptive Behavior Scales, Third Edition; VMI-6, Beery-Buktenica Developmental Test of Visual Motor Integration Test, Sixth Edition; WAIS-IV, Wechsler Adult Intelligence Scale, Fourth Edition; WIAT-III, Wechsler Individual Achievement Test, Third Edition; WISC-V, Wechsler Intelligence Scale for Children, Fifth Edition; WRAT-4, Wide Range Achievement Test, Fourth Edition.

^a^ The normative mean score is 100, and the SD is 15 for all tests, with these exceptions: BRIEF-2^26^; CDI-2; Conners’ Scales; MASC-2 (mean [SD], 50 [10]); D-KEFS (mean [SD], 10 [3])^27^; SRS-2 (reports T score ≥76 for severe, 66-75 for moderate, 60-65 for mild, and <60 for typical symptoms)^28^; and BAI and BDI-II (score, 30-63 for severe, 17-29 for moderate, 10-16 for mild, and 0-9 for minimal symptoms).^29,30^

^b^ A lower score indicates more impairment.

^c^ A higher score indicates more impairment.

### Brain MRI Measures

The number and size of focal infarcts did not differ by dDNV-NR heterozygous status (eTable 5 in Supplement 1). None of the quantitative MRI measures (whole brain volume, mean cortical thickness, cortical surface area, ventricular volume, mean white matter diffusivity, mean white matter fractional anisotropy, and mean within-network correlation of the default mode network from Gordon parcellation) differed significantly by dDNV-NR status (eTable 6 in Supplement 1).

### Post Hoc Analyses

Presence of a dDNV and/or pLOF in a chromatin-modifying gene was associated with a lower mean (SD) verbal comprehension index (91.4 [20.4] vs 103.4 [17.8]; *P* = .01) and working memory score (73.8 [16.4] vs 97.2 [15.7]; *P* = .03) (Table 3 and eTable 7 in Supplement 1). A dDNV and/or pLOF in a chromatin-modifying gene was also associated with impaired social responsiveness (Social Responsiveness Scale, Second Edition [SRS-2]^28^: mean [SD] score, 57.3 [17.2] vs 49.4 [11.2]; *P* = .03) and increased likelihood of autism spectrum disorder based on the SRS-2 and Autism Diagnostic Observation Schedule^31^ composite outcome (4 of 16 cases [28.6%] vs 8 of 202 controls [5.2%]; *P* = .01). Presence of a dDNV and/or pLOF in an HBE gene was associated with increased adult attention-deficit/hyperactivity disorder symptoms (mean [SD] scores on Conners adult attention-deficit hyperactivity disorder rating scale,^32^ 55.5 [15.4] vs 46.6 [12.3]; *P* = .007), whereas presence of a dDNV and/or pLOF in a gene with a high pLI score was associated with lower Wide Range Assessment of Memory and Learning, Second Edition,^33^ scores for immediate recall of story (mean [SD], 9.7 [3.7] vs 10.7 [3.0]; *P* = .03) and picture memory (mean [SD], 7.8 [3.1] vs 9.0 [2.9]; *P* = .008). Presence of a dDNV and/or pLOF in an NDD risk gene was not associated with any ND outcome, though this study specifically excluded participants with a dDNV in a gene already associated with NDD.

**Table 3.** Neurodevelopmental Outcomes in Post Hoc Analysis

Neurodevelopmental test^a^	Presence of chromatin dDNV and/or pLOF variant		*P* value from mixed model
	Yes (n = 16)	No (n = 202)	
**Academics**			
WRAT-4 (≥8 y)^b^			
Reading composite			
No.	15	177	NA
Score, range	54-131	54-137	.08
Score, mean (SD)	95.5 (20.30)	104.2 (14.97)	
Spelling			
No.	15	177	NA
Score, range	54-117	54-145	.15
Score, mean (SD)	95.4 (15.9)	103.4 (16.5)	
Math computation			
No.	16	193	NA
Score, range	54-124	41-139	.51
Score, mean (SD)	94.0 (19.9)	97.8 (18.0)	
**Intelligence**			
WISC-V (<16 y) or WAIS-IV (≥16 y)^b^			
Full-Scale IQ all ages standard score			
No.	16	193	NA
Score, range	39-129	39-136	.13
Score, mean (SD)	89.9 (20.5)	97.8 (17.5)	
Verbal Comprehension Index all ages standard score			
No.	16	200	NA
Score, range	49-130	44-145	.01
Score, mean (SD)	91.4 (20.4)	103.4 (17.8)	
WAIS-IV (≥16 y)^b^			
Working memory			
No.	5	87	NA
Score, range	49-95	49-136	.03
Score, mean (SD)	73.8 (16.4)	97.2 (15.7)	
**ADOS-2 and SRS-2**			
Composite outcome defined by ADOS-2 and SRS-2 total score, No. (%)^c^			
Nonspectrum	10 (71.4)	147 (94.8)	.01
Autism or autism spectrum	4 (28.6)	8 (5.2)	
SRS-2 total score^c^			
No.	15	183	NA
Score, range	38-99	11-99	.03
Score, mean (SD)	57.3 (17.2)	49.4 (11.2)	

Abbreviations: ADOS-2, Autism Diagnostic Observation Schedule, Second Edition; dDNV, damaging de novo variant; NA, not applicable; pLOF, potential loss of function; SRS-2, Social Responsiveness Scale, Second Edition; WAIS-IV, Wechsler Adult Intelligence Scale, Fourth Edition; WISC-V, Wechsler Intelligence Scale for Children, Fifth Edition; WRAT-4, Wide Range Achievement Test, Fourth Edition.

^a^ The normative mean score is 100, and the SD is 15 for all tests, with these exceptions: SRS-2 (reports T score ≥76 for severe, 66-75 for moderate, 60-65 for mild, and <60 for typical symptoms).^28^

^b^ A lower score indicates more impairment.

^c^ A higher score indicates more impairment.

Further, none of the 7 MRI metrics was different when participants were analyzed according to presence or absence of a dDNV and/or pLOF in an HBE or chromatin-modifying gene. Despite a lack of difference in brain volumes or cortical thickness, the presence of a dDNV and/or pLOF in an NDD risk gene was associated with increased ventricular volume (mean [SD], 24 792 [16 598] mm^3^ vs 14 325 [9863] mm^3^; *P* = .02) and increased mean (SD) cerebral white matter diffusivity (0.80 [0.04] vs 0.78 [0.02]; *P* = .0496). Presence of a dDNV and/or pLOF in a gene with a high pLI score was associated with lower rs-fMRI within-network correlation of the default mode network (mean [SD], 0.25 [0.04] vs 0.26 [0.08]; *P* = .03).

## Discussion

In this cohort of participants with CHD, from which individuals with established dDNVs that cause NDD were excluded, the presence of a dDNV-NR was not significantly associated with any academic, neuropsychologic, or behavior outcomes. Further, there was no association with findings on structural MRI or with 7 global metrics of structure, white matter organization, and function. These findings suggest that dDNV-NRs are not, as a group, associated with worse ND performance or multimodal brain MRI outcomes at school age or older among individuals with CHD who do not have dDNVs or CNVs that have been associated with risk for NDD.

In contrast, post hoc analyses that assessed the combined cohort according to the presence or absence of dDNV and/or pLOF found that participants with chromatin-modifying gene variants had lower verbal comprehension index and working memory scores as well as a higher likelihood of an autism spectrum disorder. These associations were found despite exclusion of dDNVs and pLOFs in chromatin-modifying genes known to be associated with NDD risk, such as *CHD7* and *KMT2D.* Our findings are consistent with prior studies from the overall PCGC cohort that found associations between damaging variants in chromatin-modifying genes and adverse ND outcomes assessed with only dichotomous screening questions in a medical history questionnaire.^16^ Chromatin-modifying genes regulate gene expression by altering the accessibility of DNA for transcription. They are often concurrently expressed in both the developing heart and the brain, and alteration in the function of a single chromatin-modifying gene can impact the expression of a large number of genes. Thus, it is possible that dDNVs and pLOFs in chromatin-modifying genes simultaneously alter both heart and brain development. Future studies should test the functional impact of relevant variants during development. The associations between dDNVs, pLOFs, neurocognitive function, and MRI metrics varied according to the specific gene categories, indicating that future studies may uncover genotype-phenotype correlations that help explain NDD among patients with CHD.

Our ability to estimate the clinical relevance of genetic variants as a group for individual outcomes was limited due to the genetic heterogeneity in disease risk, the relatively low population-attributable risk of any given variant, and imperfect estimation of the impact of genetic variants. In this study, we found that dDNV-NRs as a group were not associated with NDD risk among individuals with CHD. There are many potential explanations for this observation. There is likely significant heterogeneity in how individual genes impact brain structure, so analyzing groups of many genes can cause the signal of individual genes or functional pathways to be lost. Genes and CNVs already associated with NDDs that were excluded from this analysis may be the most significant drivers of measured outcomes. dDNV-NRs may have a small-magnitude effect not detectable in our cohort, particularly among groups of patients with CHD with a high burden of acquired central nervous system injury, such as those with single-ventricle heart disease. Not all variants estimated to be damaging will result in altered protein function, while some estimated to be benign but excluded from this analysis might lead to changes in function and a measurable phenotype. Better-populated subset analyses based on gene function may improve ability to detect genetic risk.

### Limitations

Limitations to this study include the sample size, which increases the likelihood of a type II error. The study of individual genes with high genetic constraint and, therefore, few variants in the human population will be underpowered to detect clinical impact without a large sample size. Further, genes may be constrained due to essential functions unrelated to the structure or function of the brain and heart, so using only genetic constraint as an inclusion criterion may not enrich genes relevant to the measured outcomes. Also, survivor bias could have reduced our ability to identify genes concurrently associated with early death in this cohort of older children and adults. Genes may influence NDD risk via interaction with environmental exposures such as hypoxia-ischemia; such nongenetic risk factors for adverse NDD outcomes across the cohort could have lessened the measurable impact of the variants in our study. In addition, resiliency during subsequent development may minimize the effect of some genes that impact vulnerability or resistance to neonatal brain injury, and therefore, those impacts may be less apparent by school age.

Additional pragmatic limitations include the heterogeneity of CHD types and patient ages, which may have diminished the variance in outcomes attributed to genetic factors. The post hoc analyses were not based on matched groups. We did not adjust statistical significance for multiple comparisons in this hypothesis-generating study. The patient population had a high level of maternal education, and the participants’ neurodevelopment in most domains was average or higher. We cannot exclude the possibility that factors associated with a higher maternal educational level mitigated some of the adverse effects of genetic variants.^34,35^ Parental and teacher questionnaires were not returned for all participants, which could introduce bias. Additionally, most of the participants were White, which is not reflective of the US population, and we could not assess the association of social determinants of health (eg, race, poverty, and access to care) with neurologic outcomes. The core MRI measures addressed only a subset of potential associations with brain structure and function. We focused on single nucleotide variants and small insertions or deletions in coding regions but did not assess the impact of CNVs or other structural variants or of noncoding variants. Rare variants were identified without consideration of genetic ancestry, and gene lists did not discriminate between associations with known recessive or dominant genetic conditions. The genetic basis of some NDDs is polygenic,^36^ and analysis of a single variant may not be sufficient to detect effects. Future work will include the in-depth study of individual neurodevelopmental measures, starting with MRI phenotypes to identify genetic associations, and consideration of other genetic risks such as those related to gene-gene interactions and common variants.

## Conclusions

In this study, we found that the presence of a dDNV-NR was not associated with adverse ND performance or brain MRI findings among individuals with CHD at school age or older. However, dDNVs and/or pLOFs, especially in chromatin-modifying genes, may be associated with worse ND and behavioral performance. dDNVs and pLOFs in constrained and known NDD genes were also associated with changes in brain ventricular volumes and diffusion metrics and rs-fMRI networks, respectively. Innovations that increase the scale of participant engagement, depth of clinical and genetic characterization, and capture of relevant outcomes are needed to confirm our post hoc analyses, the findings of which suggest an association of ND impairments with subgroups of dDNVs and pLOFs based on gene function or expression.

## Notes

Supplement 1. **eTable 1.** Ad Hoc Gene Lists **eTable 2.** Gene Variants **eTable 3.** Neurodevelopmental Tests **eTable 4.** Full Results for Neurodevelopmental Outcomes by Case-Control Status **eTable 5.** Brain MRI Findings by Case-Control Status **eTable 6.** MRI Differences by Case-Control Status and Post Hoc Analysis **eTable 7.** Neurodevelopmental Outcomes in Post Hoc Analysis **eFigure 1.** Patient Enrollment Flow Diagram **eFigure 2.** Venn Diagram of Shared Genes Between the 4 Gene Lists Used in the Ad Hoc Analysis
eMethods.

Supplement 2.
Data Sharing Statement
