# Supplementary material for: Association of Potentially Damaging De Novo Gene Variants With Neurologic Outcomes in Congenital Heart Disease
Source: JAMA Netw Open. 2023 Jan 26;6(1):e2253191. doi: 10.1001/jamanetworkopen.2022.53191 (PMC9880793; doi:10.1001/jamanetworkopen.2022.53191)
Supplement: Supplement 1. — eTable 1. Ad Hoc Gene Lists eTable 2. Gene Variants eTable 3. Neurodevelopmental Tests eTable 4. Full Results for Neurodevelopmental Outcomes by Case-Control Status eTable 5. Brain MRI Findings by Case-Control Status eTable 6. MRI Differences by Case-Control Status and Post Hoc Analysis eTable 7. Neurodevelopmental Outcomes in Post Hoc Analysis eFigure 1. Patient Enrollment Flow Diagram eFigure 2. Venn Diagram of Shared Genes Between the 4 Gene Lists Used in the Ad Hoc Analysis eMethods. [file jamanetwopen-e2253191-s001.pdf]

Supplementary Online Content

Morton SU, Norris-Brilliant A, Cunningham S, et al. Association of potentially damaging de novo gene variants with neurologic outcomes in congenital heart disease. *JAMA Netw Open*. 2023;6(1):e2253191. doi:10.1001/jamanetworkopen.2022.53191

eTable 1. Ad Hoc Gene Lists
eTable 2. Gene Variants
eTable 3. Neurodevelopmental Tests
eTable 4. Full Results for Neurodevelopmental Outcomes by Case-Control Status
eTable 5. Brain MRI Findings by Case-Control Status
eTable 6. MRI Differences by Case-Control Status and Post Hoc Analysis
eTable 7. Neurodevelopmental Outcomes in Post Hoc Analysis
eFigure 1. Patient Enrollment Flow Diagram
eFigure 2. Venn Diagram of Shared Genes Between the 4 Gene Lists Used in the Ad Hoc Analysis
eMethods.

© 2023 Morton SU et al. *JAMA Network Open*.

eTable 1: ad hoc gene lists			
Chromatin Modifying Genes	HBE Genes	High pLI Genes	NDD Genes
ACTB	AAAS	MARCH5	ABCB7
ACTL6A	AACS	MARCH6	ABCD1
ACTL6B	AAGAB	MARCH7	ACSL4
ACTR6	AAMP	MARCH9	ACTB
ACTR8	AAR2	SEPT5	ACTG1
AEBP2	AARS	SEPT6	ACTL6B
AICDA	AARSD1	SEPT7	ADAR
ANP32E	ABCA2	SEPT9	ADNP
AOF1	ABCD3	SEPT11	AFF2
APBB1	ABCE1	AAK1	AFF3
APITD1	ABCF1	AAMP	AFF4
APOBEC1	ABCF2	ABCA2	AFG3L2
ARID1A	ABCG2	ABCB7	AGTR2
ARID1B	ABHD12	ABCD1	AHDC1
ARID2	ABHD17A	ABCE1	AIFM1
ARID4A	ABHD17B	ABHD13	ALDH18A1
ARID4B	ABHD17C	ABHD17C	ALG13
ARID5A	ABHD4	ABHD2	ANK2
ARRB1	ABI1	ABHD8	ANKH
ASF1A	ABI2	ABI1	ANKRD11
ASF1B	ABL1	ABL1	AP1S2
ASH1L	ABLIM1	ABLIM1	AP2S1
ASH2L	ACACA	ABR	AR
ASXL1	ACAD9	AC005358.1	ARCN1
ASXL2	ACAP3	AC006486.9	ARHGEF6
ATF2	ACAT2	AC007952.5	ARHGEF9
ATM	ACBD3	AC073610.5	ARID1A
ATRX	ACIN1	AC104534.3	ARID1B
ATXN7	ACLY	AC117834.1	ARID2
ATXN7L3	ACO1	ACACA	ARSE
AURKA	ACO2	ACAN	ARX
AURKB	ACOT7	ACAP2	ASH1L
AURKC	ACP1	ACE2	ASXL1
BABAM1	ACSL3	ACER3	ASXL2
BAG6	ACSL4	ACHE	ASXL3
BAHCC1	ACTB	ACIN1	ATAD3A
BAHD1	ACTG1	ACOT7	ATP1A3
BANP	ACTL6A	ACRC	ATP6AP2
BAP1	ACTN1	ACSL4	ATP6V1B2
BAP18	ACTN4	ACTA2	ATP7A
BAZ1A	ACTR1A	ACTB	ATRX
BAZ1B	ACTR1B	ACTC1	AUTS2
BAZ2A	ACTR2	ACTL6A	BAZ2B
BCOR	ACTR3	ACTN1	BCAP31
BCORL1	ACTR8	ACTN2	BCKDK
BHMG1	ACVR1B	ACTN4	BCL11A
BMI1	ACVR2B	ACTR1A	BHLHA9
BPTF	ADAM10	ACTR2	BMP2

© 2023 Morton SU et al. *JAMA Network Open*.

BRCA1	ADAM17	ACTR3	BPIFB6
BRCA2	ADAMTS9	ACTR3B	BPTF
BRCC3	ADAR	ACVR1B	BRAF
BRD1	ADCY6	ACVR2A	BRPF1
BRD2	ADD1	ACVR2B	BRWD3
BRD3	ADD3	ADAM10	CACNA1A
BRD4	ADH5	ADAM17	CACNA1D
BRD7	ADI1	ADAM23	CACNA1E
BRD8	ADIPOR1	ADAMTS1	CACNA1H
BRD9	ADIPOR2	ADAMTS10	CACNA2D3
BRDT	ADNP	ADAMTS2	CACNB4
BRE	ADNP2	ADAMTS6	CAD
BRMS1	ADO	ADAP1	CAMK2A
BRMS1L	ADPRH	ADARB1	CAMK2B
BRPF1	ADPRHL2	ADARB2	CAMTA1
BRPF3	ADRBK1	ADCY1	CAPRIN1
C11orf30	ADRM1	ADCY2	CASK
C14orf169	ADSL	ADCY5	CBL
C17orf49	ADSS	ADCY9	CCDC22
CABIN1	AEBP2	ADCYAP1	CCDC88A
CAMK2D	AEN	ADD1	CCND2
CARM1	AES	ADD2	CD96
CASC5	AFF4	ADD3	CDH15
CBX2	AGAP1	ADIPOR1	CDK13
CBX3	AGAP3	ADNP	CDK16
CBX4	AGAP4	ADNP2	CDKL5
CBX6	AGAP5	ADORA2A	CDON
CBX7	AGAP6	ADRA2C	CELF4
CBX8	AGAP7	ADRB2	CHAMP1
CCDC101	AGAP8	ADRBK1	CHD2
CCNB1	AGFG1	ADRBK2	CHD4
CDC73	AGGF1	ADRM1	CHD7
CDK1	AGO1	AEBP2	CHD8
CDK2	AGO2	AES	CHRNA2
CDK9	AGO3	AF196779.12	CHRNA4
CDKN2A	AGPAT1	AFF1	CHRNB2
CDY1	AGPAT6	AFF2	CIC
CDY1B	AGPS	AFF3	CLCN4
CDY2A	AGRN	AFF4	CLIC2
CDY2B	AGTPBP1	AFTPH	CLTC
CDYL	AHCTF1	AGAP1	CNKSR2
CECR2	AHCYL1	AGAP2	CNOT3
CENPA	AHDC1	AGAP3	CNTN4
CENPC	AHSA1	AGFG1	CNTNAP2
CENPH	AHSA2	AGO1	COL4A1
CENPI	AIF1L	AGO2	COL4A2
CENPK	AIMP1	AGO3	COL4A3BP
CENPL	AIMP2	AGO4	COL6A1
CENPM	AJUBA	AGPAT1	CORO1A
CENPN	AK2	AGPAT3	CREBBP
CENPO	AK3	AGPS	CSNK1G1

© 2023 Morton SU et al. *JAMA Network Open*.

CENPP	AKAP1	AHCTF1	CSNK2A1
CENPQ	AKAP12	AHCYL1	CTCF
CENPR	AKAP2	AHCYL2	CTNNB1
CENPT	AKAP8	AHDC1	CTNND2
CENPU	AKAP8L	AHNAK	CUL3
CENPV	AKIRIN2	AHR	CUL4B
CENPW	AKR1A1	AHSA1	CUX1
CHD1	AKR7A2	AIFM1	DCX
CHD1L	AKR7A3	AJAP1	DDX3X
CHD2	AKT1	AJUBA	DEAF1
CHD3	AKT3	AKAP1	DEPDC5
CHD4	ALAD	AKAP11	DHDDS
CHD5	ALDH16A1	AKAP13	DHX30
CHD6	ALDH18A1	AKAP14	DIP2A
CHD7	ALDH1A3	AKAP17A	DIP2B
CHD8	ALDH2	AKAP4	DIP2C
CHD9	ALDH7A1	AKAP5	DKC1
CHEK1	ALDH9A1	AKAP6	DLG3
CHRAC1	ALDOA	AKAP8	DLG4
CLOCK	ALG10	AKIRIN1	DLL4
COPRS	ALG10B	AKIRIN2	DMD
CPA4	ALG2	AKT1	DNM1
CREBBP	ALG3	AKT2	DNMT3A
CRTC2	ALKBH1	AKT3	DPYSL2
CSRP2BP	ALKBH5	ALAS2	DSCAM
CTBP1	ALS2	ALB	DYNC1H1
CTCF	ALX3	ALCAM	DYRK1A
CTCFL	ALX4	ALDH1A1	EBF3
CTD-2132N18.3	ALYREF	ALG13	EBP
CTNNB1	AMBRA1	ALKBH5	EED
CTR9	AMD1	ALYREF	EEF1A2
CUL4B	AMFR	AMBRA1	EFTUD2
CXXC1	AMMECR1L	AMD1	EHMT1
DAPK3	AMOT	AMER1	EIF2S3
DAXX	AMOTL1	AMER3	EIF3G
DDB1	AMOTL2	AMIGO1	ELAVL3
DDB2	AMPD2	AMIGO2	EMC1
DEK	AMT	AMMECR1	EMX2
DKFZp686C08112	ANAPC1	AMMECR1L	EP300
DMAP1	ANAPC16	AMOT	EPB41L1
DMRTC2	ANAPC2	ANAPC1	ERBIN
DNAJC2	ANAPC4	ANAPC2	ERMARD
DNMT1	ANAPC5	ANAPC7	EZH2
DNMT3A	ANAPC7	ANGPT1	FAM111A
DNMT3B	ANKFY1	ANGPT2	FBXO11
DOT1L	ANKHD1	ANK1	FBXO25
DPF3	ANKLE2	ANK2	FGF12
DPPA2	ANKRD10	ANK3	FGFR1
DPPA3	ANKRD11	ANKFY1	FLNA
DPY30	ANKRD13A	ANKHD1	FMR1
DR1	ANKRD13B	ANKHD1-EIF4EBP3	FOXG1

© 2023 Morton SU et al. *JAMA Network Open*.

DTX3L	ANKRD17	ANKIB1	FOXP1
EDF1	ANKRD27	ANKRD11	FOXP2
EED	ANKRD28	ANKRD12	FOXP3
EHMT1	ANKRD40	ANKRD13C	FRMD7
EHMT2	ANKRD52	ANKRD17	FRMPD4
EID1	ANKRD6	ANKRD28	FTSJ1
ELK4	ANLN	ANKRD34A	FZD5
ELP3	ANO6	ANKRD46	GABBR2
ELP4	ANP32A	ANKRD50	GABRA1
EMSY	ANP32B	ANKRD52	GABRB2
ENY2	ANP32D	ANKS1A	GABRB3
EP300	ANP32E	ANKS1B	GABRG2
EP400	ANXA5	ANO1	GATAD2B
EPC1	ANXA6	ANO8	GCH1
EPC2	AP000304.12	ANP32A	GDI1
ESR1	AP1AR	ANP32B	GFAP
EYA1	AP1B1	ANP32E	GIGYF1
EYA2	AP1G1	ANTXR1	GIGYF2
EYA3	AP1M1	AP000783.1	GJC2
EYA4	AP1S1	AP1B1	GLI2
EZH1	AP1S3	AP1G1	GLI3
EZH2	AP2A1	AP1M1	GMNN
FAM175A	AP2A2	AP1S2	GNAI1
FBL	AP2B1	AP2A1	GNAO1
FLCN	AP2M1	AP2A2	GNAS
FOXA1	AP2S1	AP2B1	GNB1
FOXA2	AP3B1	AP2M1	GRIA1
FOXA3	AP3D1	AP2S1	GRIA2
FOXP3	AP3S1	AP3B1	GRIA3
FSHR	AP3S2	AP3B2	GRIN1
GATA2	AP4M1	AP3D1	GRIN2A
GATA3	APBA2	APBA1	GRIN2B
GATAD2B	APBB1	APBA2	GRIN2D
GCG	APCDD1	APBB1	GRIP1
GFI1B	APEH	APBB1IP	GSPT2
GSG2	APEX1	APC	HCFC1
GTF3C4	API5	APC2	HCN1
H2AFY	APLP1	APEX2	HDAC4
H2AFY2	APLP2	APH1A	HDAC8
H4_HUMAN	APMAP	API5	HDLBP
HAT1	APP	APLNR	HECTD4
HCFC1	APPBP2	APOO	HECW2
HDAC1	APRT	APPBP2	HESX1
HDAC10	AQR	APPL1	HIST1H1E
HDAC11	ARAF	AQR	HIST1H4C
HDAC2	ARCN1	AR	HIVEP2
HDAC3	ARF1	ARAF	HMGB3
HDAC4	ARF3	ARC	HNRNPH2
HDAC5	ARF4	ARCN1	HNRNPU
HDAC6	ARF5	ARF1	HPD
HDAC7	ARF6	ARF4	HPRT1

© 2023 Morton SU et al. *JAMA Network Open*.

HDAC8	ARFGAP1	ARF5	HRAS
HDAC9	ARFGAP2	ARF6	HSD17B10
HELLS	ARFRP1	ARFGEF1	HUWE1
HILS1	ARGLU1	ARFGEF2	IDS
HIPK4	ARHGAP1	ARFIP2	IFIH1
HIRA	ARHGAP11B	ARGLU1	IGBP1
HIST1H1B	ARHGAP19	ARHGAP1	IL1RAPL1
HIST1H2BA	ARHGAP21	ARHGAP21	ILF2
HIST1H4A	ARHGAP23	ARHGAP23	INTS6
HIST1H4B	ARHGAP28	ARHGAP25	IQSEC2
HIST1H4C	ARHGAP29	ARHGAP26	IRF2BPL
HIST1H4D	ARHGAP31	ARHGAP29	ITPR1
HIST1H4E	ARHGAP33	ARHGAP30	KANSL1
HIST1H4F	ARHGAP35	ARHGAP31	KAT2B
HIST1H4H	ARHGAP39	ARHGAP32	KAT6A
HIST1H4I	ARHGDIA	ARHGAP35	KAT6B
HIST1H4J	ARHGEF1	ARHGAP36	KATNAL2
HIST1H4K	ARHGEF10	ARHGAP4	KCNA2
HIST1H4L	ARHGEF17	ARHGAP42	KCNB1
HIST2H4A	ARHGEF2	ARHGAP44	KCNC1
HIST2H4B	ARHGEF25	ARHGAP5	KCNC3
HIST4H4	ARHGEF40	ARHGAP6	KCNH1
HJURP	ARHGEF7	ARHGDIG	KCNH5
HLCS	ARID1A	ARHGEF1	KCNJ11
HLTF	ARID2	ARHGEF11	KCNJ6
HMG20A	ARID3A	ARHGEF12	KCNMA1
HMG20B	ARID3B	ARHGEF17	KCNQ2
HMGA1	ARIH1	ARHGEF2	KCNQ3
HMGA2	ARL1	ARHGEF6	KCNQ5
HMGB1	ARL10	ARHGEF7	KCNT1
HMGB2	ARL2	ARHGEF9	KDM1A
HMGB3	ARL2BP	ARID1A	KDM5B
HMGB4	ARL3	ARID1B	KDM5C
HMGN3	ARL5A	ARID2	KDM6A
HMGN5	ARL5B	ARID3A	KDM6B
HMGXB4	ARL6IP1	ARID4A	KIAA0232
HNF1A	ARL6IP4	ARID4B	KIDINS220
HNRNPC	ARL6IP6	ARID5A	KIF11
HOPX	ARL8A	ARID5B	KIF1A
HR	ARL8B	ARIH1	KIF2A
HSF4	ARMC1	ARIH2	KIF4A
HUWE1	ARMCX2	ARL4A	KIF5C
IKZF1	ARNT	ARL4C	KIRREL3
IL1B	ARNT2	ARL8A	KITLG
ING1	ARPC1A	ARMC1	KLF8
ING2	ARPC2	ARMC8	KLHL15
ING3	ARPC3	ARMCX2	KMT2A
ING4	ARPC4	ARMCX4	KMT2B
ING5	ARPC5	ARNT	KMT2C
INO80	ARPC5L	ARNT2	KMT2D
INO80B	ARPP19	ARNTL	KMT2E

© 2023 Morton SU et al. *JAMA Network Open*.

INO80B-WBP1	ARRB1	ARPC1A	KMT5B
INO80C	ARV1	ARPC2	KPNA7
IRF4	ARVCF	ARPC4	KRAS
ITGB3BP	ASAP1	ARPC5	KRIT1
IWS1	ASAP2	ARPC5L	L1CAM
JADE1	ASB6	ARPP19	LAS1L
JADE2	ASCC2	ARRB1	LDB1
JADE3	ASF1B	ARRB2	LEO1
JAK2	ASH2L	ARRDC3	LGI1
JARID1C	ASIP	ARSE	LRP2
JARID2	ASNA1	ARX	LRRC4C
JDP2	ASNS	ASAP1	MAB21L2
JMJD1C	ASNSD1	ASAP2	MAF
JMJD6	ASPSCR1	ASB7	MAGEL2
K7ERQ8	ASUN	ASB9	MAGI2
KANSL1	ASXL1	ASCL1	MAGT1
KANSL2	ATAD1	ASF1A	MAOA
KANSL3	ATAD2	ASH1L	MAP1A
KAT2A	ATAD3A	ASH2L	MAP2K1
KAT2B	ATAD3B	ASIC1	MAP2K2
KAT5	ATAD3C	ASIC2	MAPK10
KAT6A	ATAT1	ASNA1	MBD5
KAT6B	ATF1	ASTN1	MBOAT7
KAT7	ATF2	ASTN2	MECP2
KAT8	ATF4	ASUN	MED12
KDM1A	ATF6B	ASXL2	MED13
KDM1B	ATF7IP	ASXL3	MED13L
KDM2A	ATG12	ATAD2	MEF2C
KDM2B	ATG2A	ATAD2B	MET
KDM3A	ATG4B	ATAD5	MID1
KDM3B	ATIC	ATCAY	MKX
KDM4A	ATN1	ATF1	MORC2
KDM4B	ATP11A	ATF2	MSL3
KDM4C	ATP13A1	ATF7	MSNP1AS
KDM4D	ATP13A2	ATF7IP	MTF1
KDM4E	ATP13A3	ATG13	MTOR
KDM5A	ATP1A1	ATG16L1	MYCN
KDM5B	ATP1B3	ATG4A	MYH10
KDM5C	ATP2A2	ATG4B	MYT1L
KDM5D	ATP2B1	ATG5	NAA10
KDM6A	ATP2C1	ATG9A	NAA15
KDM6B	ATP5A1	ATL1	NACC1
KDM7A	ATP5B	ATN1	NALCN
KDM8	ATP5C1	ATOH8	NCAPD2
KLF1	ATP5D	ATOX1	NCAPD3
KMT2A	ATP5G1	ATP11C	NCKAP1
KMT2B	ATP5G2	ATP13A1	NCOA1
KMT2C	ATP5G3	ATP13A3	NDP
KMT2D	ATP5H	ATP1A1	NDUFA1
KMT2E	ATP5J	ATP1A3	NEDD4L
L3MBTL1	ATP5J2	ATP1B1	NEXMIF

L3MBTL2	ATP5O	ATP2A2	NF1
L3MBTL3	ATP6AP1	ATP2B1	NFIA
L3MBTL4	ATP6V0B	ATP2B2	NFIX
LDB1	ATP6V0C	ATP2B3	NIPBL
LEF1	ATP6V0D1	ATP2C1	NKX2-1
LEO1	ATP6V1A	ATP5A1	NLGN3
LIF	ATP6V1B2	ATP5B	NLGN4X
LOXL2	ATP6V1E1	ATP6AP1	NLRP5
LPIN1	ATP8B2	ATP6AP2	NONO
LRWD1	ATPIF1	ATP6V0A1	NR1I3
M18BP	ATR	ATP6V0B	NR2F1
M18BP_HUMAN	ATRIP	ATP6V0C	NR3C2
MAP3K12	ATRX	ATP6V0D1	NRAS
MAP3K7	ATXN10	ATP6V1A	NRXN1
MAPK3	ATXN1L	ATP6V1B2	NRXN2
MAPK8	ATXN2	ATP6V1E1	NRXN3
MBD2	ATXN2L	ATP7A	NSD1
MBD3	ATXN7L3	ATP9A	NTRK2
MBIP	ATXN7L3B	ATRN	NUP155
MBTD1	AUP1	ATRNL1	NUS1
MCRS1	AURKA	ATRX	OCRL
MEAF6	AURKAIP1	ATXN1	OFD1
MECP2	AURKB	ATXN1L	OPHN1
MED24	AVL9	ATXN2	OTC
MEN1	AXIN1	ATXN2L	OTX2
METTL8	AXIN2	ATXN7	P4HB
MGEA5	AZI1	ATXN7L1	PACS1
MIS18A	AZIN1	ATXN7L2	PACS2
MIS18BP1	B3GALNT2	ATXN7L3	PAFAH1B1
MLL2	B3GNT5	AURKA	PAK3
MORF4L1	B4GALNT4	AUTS2	PAX5
MORF4L2	B4GALT2	AVPR2	PAX6
MRGBP	B4GALT3	AXIN2	PAX8
MS18A_HUMAN	B9D1	AZIN1	PCDH19
MS18B_HUMAN	BABAM1	B2M	PCGF2
MSL1	BACE1	B3GALT2	PDCD10
MSL2	BACH1	B3GNT2	PDE10A
MSL3	BAG1	B3GNT3	PDGFRB
MSL3P1	BAG2	B3GNT5	PDHA1
MT3	BAG6	B3GNT7	PGK1
MTA2	BAK1	B4GALT5	PHF12
MTF2	BANF1	B4GALT6	PHF2
MUC1	BAP1	BABAM1	PHF21A
MYB	BASP1	BACE1	PHF3
MYC	BAX	BACH1	PHF6
MYOCD	BAZ1B	BACH2	PHF8
MYOD1	BAZ2A	BAG3	PHIP
MYSM1	BBX	BAG6	PHOX2B
NAA50	BCAM	BAHD1	PIGA
NAA60	BCAP31	BAI1	PIH1D3
NACC2	BCAR1	BAI2	PIK3R2

© 2023 Morton SU et al. *JAMA Network Open*.

NAP1L2	BCAS2	BAI3	PLP1
NASP	BCAT1	BANF1	POGZ
NCOA1	BCCIP	BANP	PPM1D
NCOA2	BCKDK	BAP1	PPP1CB
NCOA3	BCL2L13	BARHL2	PPP1R9B
NCOR1	BCL7B	BARX1	PPP2R1A
NEK11	BCL7C	BASP1	PPP2R5D
NIPBL	BCL9	BATF	PPP3CA
NO66	BCL9L	BATF2	PPP5C
NOC2L	BCLAF1	BAZ1A	PQBP1
NOS1	BCOR	BAZ1B	PRKD1
NPM	BCR	BAZ2A	PRR12
NPM_HUMAN	BDP1	BAZ2B	PRRT2
NPM1	BECN1	BBX	PSMD12
NPM2	BEND3	BCAR1	PTCH1
NR3C1	BEX1	BCAR3	PTCHD1
NSD1	BEX2	BCL11A	PTDSS1
OGT	BGN	BCL11B	PTEN
OIP5	BICD2	BCL2	PTK7
OTUB1	BIN1	BCL2L1	PUF60
PADI2	BIRC5	BCL2L11	PURA
PADI4	BLCAP	BCL3	QKI
PAF1	BLM	BCL6	QRICH1
PAX5	BLMH	BCL9L	RAB11A
PAX7	BLOC1S6	BCOR	RAB11B
PAXBP1	BMI1	BCORL1	RAB39B
PAXIP1	BMP7	BCR	RAC1
PBRM1	BMPR1A	BDNF	RAD21
PCGF1	BMS1	BECN1	RAD51
PCGF2	BNIP2	BEGAIN	RAF1
PER1	BNIP3L	BEND3	RAI1
PER2	BOC	BEND4	RANBP17
PHB	BOLA2	BEX1	RANBP2
PHC1	BOLA2B	BEX4	RARB
PHF1	BOP1	BHLHE22	RBM10
PHF13	BORA	BHLHE40	RELN
PHF19	BPTF	BHLHE41	RERE
PHF2	BRAP	BICC1	RFX3
PHF20	BRD1	BICD2	RIMS1
PHF21A	BRD2	BIN1	RLIM
PHF8	BRD3	BIRC6	RNF113A
PIM3	BRD4	BLCAP	ROR2
PKN1	BRD7	BLNK	RORB
PML	BRD8	BMI1	RPL11
POGZ	BRF1	BMP10	RPS23
POLE3	BRICD5	BMP2	RPS6KA3
POLE4	BRIX1	BMP4	RRAS
POU5F1	BRK1	BMP6	RYR3
PPARGC1A	BRMS1L	BMPR1A	SAMD9L
PPM1F	BRPF1	BMPR1B	SATB1
PPP5C	BRPF3	BMPR2	SATB2

© 2023 Morton SU et al. *JAMA Network Open*.

PRDM13	BRWD1	BNC1	SCN1A
PRDM14	BSG	BNC2	SCN1B
PRDM2	BTAF1	BOD1L1	SCN2A
PRDM5	BTBD2	BPTF	SCN3A
PRDM6	BTBD9	BRAF	SCN8A
PRDM7	BTF3	BRD1	SCN9A
PRDM8	BTRC	BRD2	SCRIB
PRDM9	BUB1	BRD3	SET
PRKAA1	BUB1B	BRD4	SETBP1
PRKAA2	BUB3	BRD7	SETD1A
PRKCA	BUD31	BRINP1	SETD5
PRKCB	BYSL	BRINP2	SHANK1
PRKD1	BZW1	BRIX1	SHANK2
PRKD2	BZW2	BRK1	SHANK3
PRMT1	C10orf76	BRPF1	SHH
PRMT2	C11orf24	BRPF3	SIK1
PRMT3	C11orf30	BRS3	SIN3A
PRMT5	C11orf31	BRSK1	SIX3
PRMT6	C11orf49	BRSK2	SKI
PRMT7	C11orf58	BRWD1	SLC12A5
PRMT8	C11orf68	BRWD3	SLC16A2
PSME4	C11orf73	BSN	SLC1A2
PYGO2	C11orf95	BTAF1	SLC25A4
RAG1	C12orf10	BTBD11	SLC2A1
RAG2	C12orf43	BTBD18	SLC35A2
RB1	C12orf45	BTBD2	SLC6A1
RBBP4	C12orf57	BTF3	SLC6A8
RBBP4_HUMAN	C14orf1	BTF3L4	SLC9A6
RBBP5	C14orf166	BTG1	SLC9A9
RBBP7	C14orf2	BTG3	SMARCA2
RBBP7_HUMAN	C14orf37	BTK	SMARCA4
RBL1	C16orf72	BUB3	SMARCB1
RBL2	C16orf80	BZW1	SMARCC2
RBM14	C17orf103	C10orf12	SMARCE1
RCBTB1	C17orf70	C10orf53	SMC1A
RCOR1	C17orf96	C10orf54	SMC3
RERE	C18orf25	C11orf30	SMS
REST	C18orf32	C11orf73	SNAP25
RING1	C19orf10	C11orf84	SNRPB
RNF168	C19orf43	C12orf44	SNX3
RNF2	C19orf52	C12orf5	SON
RNF20	C19orf53	C12orf75	SOS1
RNF40	C1orf112	C14orf2	SOX11
RNF8	C1orf159	C16orf70	SOX2
RPS6KA4	C1orf198	C16orf72	SOX3
RPS6KA5	C1orf21	C17orf103	SOX5
RRP8	C1orf63	C17orf104	SPAST
RSF1	C1orf85	C17orf85	SPTAN1
RSF1_HUMAN	C1QBP	C18orf25	SRCAP
RTF1	C20orf27	C18orf8	SRGAP3
RUVB1_HUMAN	C2CD3	C19orf33	SRP54

© 2023 Morton SU et al. *JAMA Network Open*.

RUVBL1	C2CD5	C19orf68	SRPR
RUVBL2	C2orf15	C1GALT1	SRPX2
RYBP	C3orf58	C1GALT1C1	SRSF11
SALL1	C3orf70	C1orf21	STAG1
SAP130	C4orf27	C1orf226	STX1B
SATB1	C4orf29	C1orf51	STXBP1
SATB2	C5orf15	C1orf86	SUV420H1
SCMH1	C5orf24	C1QL3	SYN1
SET	C5orf51	C1QTNF1-AS1	SYNGAP1
SETD1A	C6orf106	C1QTNF5	SYP
SETD1B	C6orf62	C1R	SYT1
SETD2	C7orf26	C20orf112	TAF1
SETD3	C7orf55-LUC7L2	C20orf24	TAOK1
SETD7	C8orf37	C20orf78	TAOK2
SETD8	C8orf76	C2CD2L	TAZ
SETDB1	C9orf114	C2orf82	TBL1XR1
SETDB2	C9orf3	C3	TBR1
SETMAR	C9orf78	C4B	TBX1
SFMBT1	C9orf85	C4orf32	TBX18
SFPQ	C9orf91	C4orf48	TCF20
SIN3A	CA14	C5orf24	TCF4
SIN3B	CA2	C6orf1	TCF7L2
SIRT1	CA4	C6orf106	TEK
SIRT2	CAB39	C6orf136	TGIF1
SIRT3	CABIN1	C6orf62	THAP1
SIRT6	CACFD1	C7orf26	THOC2
SIRT7	CACHD1	C7orf60	THRA
SKI	CACNA2D2	C7orf73	TIMM8A
SKP1	CACNB3	C9orf147	TINF2
SMAD4	CACNG4	C9orf172	TLK2
SMARCA1	CACNG7	CA10	TM4SF20
SMARCA2	CACTIN	CAAP1	TM9SF4
SMARCA4	CACUL1	CABP1	TNRC6B
SMARCA5	CACYBP	CACNA1A	TRAF7
SMARCAD1	CAD	CACNA1B	TRIM23
SMARCAL1	CADM1	CACNA1C	TRIO
SMARCB1	CALD1	CACNA1D	TRIP12
SMARCC1	CALM1	CACNA1E	TSC1
SMARCC2	CALM3	CACNA1G	TSC2
SMARCD1	CALR	CACNA1I	TSPAN7
SMARCD2	CALU	CACNA2D1	TUBA1A
SMARCD3	CAMK2G	CACNA2D2	TUBB
SMARCE1	CAMSAP1	CACNA2D3	TUBB2A
SMCA5	CAND1	CACNB1	TUBB2B
SMCA5_HUMAN	CAND2	CACNG2	TUBB3
SMYD1	CANT1	CACNG3	TUBB4A
SMYD2	CANX	CACNG7	TUBG1
SMYD3	CAP1	CACNG8	TWIST1
SNAI2	CAPN15	CACTIN	TWIST2
SNCA	CAPN2	CACUL1	UBE2A
SNW1	CAPN7	CACYBP	UBN2

© 2023 Morton SU et al. *JAMA Network Open*.

SOX9	CAPNS1	CAD	UBR1
SP100	CAPRIN1	CADM1	UBTF
SP110	CAPZA2	CADM2	UPF3B
SP140	CAPZB	CADM3	USP15
SP140L	CARHSP1	CADM4	USP27X
SPI1	CARKD	CALCRL	USP7
SPIN1	CARM1	CALD1	USP9X
SRCAP	CARS	CALM1	VEZF1
SREBF1	CASC3	CALM2	VIP
SS18L1	CASD1	CALM3	WAC
SSRP1	CASKIN2	CALR	WDFY3
STRA13	CASP2	CAMK1D	WDR26
SUDS3	CASP3	CAMK2A	WDR45
SUPT3H	CAST	CAMK2B	WNT4
SUPT4H1	CAT	CAMK2G	YAP1
SUPT5H	CBFB	CAMK4	YWHAG
SUPT6H	CBLB	CAMKV	YY1
SUPT7L	CBLL1	CAMSAP1	ZBTB18
SUV39H1	CBX1	CAMSAP2	ZBTB20
SUV39H2	CBX2	CAMSAP3	ZC4H2
SUV420H1	CBX3	CAMTA1	ZDHHC15
SUV420H2	CBX5	CAMTA2	ZDHHC9
SUZ12	CCAR1	CAND1	ZEB2
SYCP3	CCAR2	CANX	ZIC1
TADA1	CCDC124	CAP2	ZIC2
TADA2A	CCDC34	CAPN15	ZMYM6
TADA2B	CCDC47	CAPN6	ZMYND11
TADA3	CCDC50	CAPRIN1	ZMYND8
TAF1	CCDC6	CAPZA1	ZNF462
TAF10	CCDC8	CAPZA2	ZNF599
TAF12	CCDC85C	CAPZB	ZNF711
TAF1L	CCDC97	CARD11	ZNF713
TAF5	CCM2	CARM1	ACHE
TAF5L	CCNA2	CASC3	ADA
TAF6L	CCNB1	CASC5	ADCY3
TAF7	CCNB2	CASK	ADSL
TAF9	CCND1	CASKIN1	AGAP2
TBL1X	CCND2	CASKIN2	AGO1
TBL1XR1	CCND3	CASZ1	AGO4
TBL1Y	CCNE1	CBFA2T2	AKAP9
TCF3	CCNF	CBFB	ALDH5A1
TDG	CCNG1	CBLB	AMPD1
TDRD3	CCNG2	CBLL1	AMT
TERF1	CCNI	CBX1	ANK3
TERF2	CCNJ	CBX2	ANXA1
TET1	CCNJL	CBX4	APBB1
TET2	CCNK	CBX5	APH1A
TET3	CCNL1	CBX6	ASAP2
TFAM	CCNL2	CBX7	ASPM
TGFB1	CCNY	CBX8	ASTN2
TLK1	CCT2	CCAR1	ATP10A

© 2023 Morton SU et al. *JAMA Network Open*.

TLK2	CCT3	CCAR2	ATP2B2
TNP1	CCT4	CCDC112	AVPR1A
TNRC18	CCT5	CCDC117	BRSK2
TOP1	CCT6A	CCDC121	BTAF1
TOP1MT	CCT7	CCDC132	CACNA1C
TOX	CCT8	CCDC182	CACNB2
TOX2	CCZ1	CCDC22	CASZ1
TOX3	CCZ1B	CCDC6	CC2D1A
TOX4	CD151	CCDC64	CCNG1
TP53	CD164	CCDC71L	CCT4
TP63	CD276	CCDC85C	CDC42BPB
TPR	CD2AP	CCDC88A	CDH13
TRIM16	CD2BP2	CCL2	CEP135
TRIM37	CD34	CCNA2	CEP41
TRIP12	CD3EAP	CCNB3	CGNL1
TRPS1	CD63	CCNC	CHD3
TRRAP	CD81	CCND1	CHMP1A
TSPYL2	CD99L2	CCND2	CHRNA7
TTF1	CDC123	CCND3	CIB2
TWIST1	CDC16	CCNE1	CLASP1
UBE2A	CDC20	CCNE2	CNR1
UBE2B	CDC23	CCNI	CNTN5
UBE2E1	CDC25A	CCNJ	CNTN6
UBE2N	CDC25B	CCNK	CNTNAP3
UBE2U	CDC27	CCNL1	CNTNAP4
UBN1	CDC34	CCNT2	CPEB4
UBR2	CDC37	CCNY	CSDE1
UBR5	CDC42	CCNYL1	CTTNBP2
UBTF	CDC42BPB	CCSER2	CUL7
UCN	CDC42EP4	CCT2	CYFIP1
UHRF1	CDC42SE1	CCT3	DAPP1
UHRF2	CDC42SE2	CCT4	DENR
UIMC1	CDC45	CCT5	DHCR7
USP15	CDC5L	CCT6A	DISC1
USP16	CDC7	CCT7	DLG2
USP17L2	CDC73	CCT8	DLGAP1
USP21	CDCA2	CD19	DLX3
USP22	CDCA3	CD22	DMPK
USP3	CDCA4	CD40	DOCK8
USP49	CDCA5	CD40LG	DPP10
USP51	CDCA7	CD47	EFR3A
USP7	CDCA7L	CD59	ELP4
UTP3	CDCA8	CD79A	EMSY
UTY	CDH11	CD79B	EP400
VEGFA	CDH2	CD81	ETFB
VPRBP	CDH24	CD86	FAM92B
VPS72	CDH5	CDC14B	FBN1
VRK1	CDIP1	CDC16	GABRG3
WAC	CDIPT	CDC23	GALNT8
WBP2	CDK1	CDC25A	GGNBP2
WBSCR22	CDK11A	CDC26	GPC4

© 2023 Morton SU et al. *JAMA Network Open*.

WDR5	CDK13	CDC27	GPHN
WDR61	CDK16	CDC34	GRID1
WDR82	CDK17	CDC37	GRIK2
WDR92	CDK2	CDC37L1	GRIK5
WHSC1	CDK4	CDC40	HIVEP3
WHSC1L1	CDK5RAP2	CDC42	HMGN1
YEATS2	CDK5RAP3	CDC42BPA	ICA1
YEATS4	CDK9	CDC42BPB	ITGB3
ZMYND11	CDKN1C	CDC42SE2	JARID2
ZNF335	CDKN2AIPNL	CDC5L	KCNJ10
ZNHIT1	CDON	CDC73	KCNS3
	CDR2L	CDCA3	KDM3B
	CDS2	CDH11	KDM4C
	CDT1	CDH2	KIAA1586
	CDV3	CDH4	KIF14
	CEBPZ	CDH5	LAMB1
	CECR2	CDH6	LMX1B
	CELF1	CDH7	LRP1
	CELF2	CDH8	LZTR1
	CELSR1	CDH9	MACROD2
	CELSR2	CDIP1	MEIS2
	CENPA	CDK1	MFRP
	CENPB	CDK12	MIR137
	CENPE	CDK13	MYO5A
	CENPF	CDK16	MYO9B
	CENPH	CDK17	NAV2
	CENPN	CDK19	NBEA
	CENPQ	CDK2	NCOR1
	CEP104	CDK2AP2	NFE2L3
	CEP120	CDK5R1	NINL
	CEP164	CDK5R2	NLGN1
	CEP170	CDK6	NLGN2
	CEP250	CDKL5	NR4A2
	CEP57	CDKN1B	NUAK1
	CEP68	CDKN1C	NUDCD2
	CEP78	CDKN2AIP	OR52M1
	CEP85	CDV3	OTUD7A
	CERS2	CDX2	OXTR
	CERS5	CDYL	P2RX5
	CETN3	CEBPA	P4HA2
	CFDP1	CEBPD	PAH
	CFL1	CECR2	PAK2
	CFL2	CECR6	PARD3B
	CFLAR	CELF1	PER2
	CGGBP1	CELF2	PHB
	CHAF1A	CELF3	PHRF1
	CHAF1B	CELF4	PLCB1
	CHAMP1	CELF5	PLXNA4
	CHCHD2	CELSR1	PLXNB1
	CHCHD3	CELSR2	POMGNT1
	CHCHD4	CELSR3	PON1

© 2023 Morton SU et al. *JAMA Network Open*.

	CHD1	CEND1	PREX1
	CHD3	CENPB	PRICKLE1
	CHD4	CENPI	PRICKLE2
	CHD7	CEP170	PRKCB
	CHD8	CEP170B	PRKD2
	CHEK1	CEP350	PRKN
	CHERP	CEP68	PRODH
	CHFR	CEPT1	PTCHD1-AS
	CHMP3	CETN2	PTPN11
	CHMP4B	CFH	PYHIN1
	CHMP7	CFL1	RAB2A
	CHORDC1	CFLAR	RAB43
	CHST1	CFP	RALGAPB
	CHSY1	CGGBP1	RBFOX1
	CHTF18	CHAF1A	RBM27
	CHTF8	CHAMP1	ROBO2
	CHTOP	CHD1	RPS10P2-AS1
	CIC	CHD2	SAE1
	CIPC	CHD3	SBF1
	CIRBP	CHD4	SEMA5A
	CIRH1A	CHD5	SETD2
	CITED2	CHD6	SHOX
	CIZ1	CHD7	SLC35B1
	CKAP2	CHD8	SLC38A10
	CKAP2L	CHD9	SLC6A3
	CKAP4	CHERP	SLC7A3
	CKAP5	CHIC1	SLC7A5
	CKB	CHIC2	SLITRK5
	CKLF	CHM	SMAD4
	CKS2	CHMP1B	SNX5
	CLASP1	CHMP4B	SPARCL1
	CLASRP	CHMP6	SPEN
	CLCN6	CHMP7	SRPRA
	CLDN12	CHORDC1	ST8SIA2
	CLDN6	CHP1	STXBP5
	CLDND1	CHRM1	SUPT16H
	CLIC1	CHRM3	TAF6
	CLIC4	CHRM4	TANC2
	CLINT1	CHST1	TBC1D31
	CLIP2	CHSY1	TBCK
	CLIP3	CHTF8	TERF2
	CLK1	CHTOP	TET2
	CLK2	CHUK	TMLHE
	CLK3	CIC	TRAPPC9
	CLNS1A	CIT	TRPC6
	CLPP	CITED2	TRPM1
	CLPTM1	CKAP4	TSHZ3
	CLPTM1L	CKAP5	UBE3A
	CLSTN1	CLASP1	UBE3C
	CLTA	CLASP2	UBR5
	CLTC	CLCF1	UNC79

© 2023 Morton SU et al. *JAMA Network Open*.

	CLUH	CLCN3	USP45
	CMAS	CLCN4	VIL1
	CMIP	CLCN5	VPS13B
	CMPK1	CLDN11	WDFY4
	CMTM3	CLDN2	WWOX
	CMTR1	CLDN3	ZC3H4
	CNBP	CLDN5	ZMIZ1
	CNDP2	CLINT1	ZNF292
	CNIH1	CLIP1	ZNF804A
	CNIH2	CLIP2	
	CNN2	CLIP3	
	CNN3	CLK2	
	CNOT1	CLOCK	
	CNOT11	CLPP	
	CNOT2	CLPTM1	
	CNOT3	CLPX	
	CNOT6	CLSTN1	
	CNOT7	CLSTN3	
	CNOT8	CLTA	
	CNPY2	CLTC	
	CNPY4	CLUH	
	CNTFR	CLVS1	
	CNTRL	CMAS	
	COA3	CMIP	
	COA5	CMPK1	
	COG1	CMTR1	
	COG4	CNBP	
	COG8	CNIH1	
	COL18A1	CNIH2	
	COL1A1	CNIH3	
	COL1A2	CNKSR2	
	COL2A1	CNN3	
	COL3A1	CNNM2	
	COL4A1	CNOT1	
	COL4A2	CNOT10	
	COL5A2	CNOT11	
	COLEC12	CNOT2	
	COLGALT1	CNOT3	
	COMMD4	CNOT4	
	COPA	CNOT6L	
	COPB1	CNOT7	
	COPB2	CNP	
	COPE	CNR1	
	COPG1	CNTFR	
	COPG2	CNTNAP5	
	COPS2	COG3	
	COPS3	COL11A1	
	COPS4	COL11A2	
	COPS5	COL12A1	
	COPS6	COL1A1	
	COPS7A	COL1A2	

© 2023 Morton SU et al. *JAMA Network Open*.

	COPS7B	COL27A1	
	COPS8	COL2A1	
	COPZ1	COL3A1	
	COQ2	COL4A1	
	COQ5	COL4A3BP	
	CORO1B	COL4A5	
	CORO1C	COL4A6	
	COTL1	COL5A1	
	COX15	COL5A2	
	COX16	COL6A1	
	COX19	COLCA2	
	COX20	COLEC12	
	COX4I1	COPA	
	COX5A	COPB1	
	COX5B	COPB2	
	COX6A1	COPE	
	COX6B1	COPS2	
	COX6C	COPS3	
	COX7A2	COPS4	
	COX8A	COPS5	
	CPE	COPS6	
	CPLX2	COPS7B	
	CPNE1	COPS8	
	CPSF1	COPZ1	
	CPSF2	CORO1A	
	CPSF3	CORO1C	
	CPSF3L	COX5B	
	CPSF4	COX7B	
	CPSF6	COX7C	
	CPSF7	CPE	
	CRABP1	CPEB1	
	CRABP2	CPEB3	
	CRAMP1L	CPEB4	
	CRB2	CPLX1	
	CREB1	CPLX2	
	CREBBP	CPSF6	
	CREBZF	CPSF7	
	CRELD2	CRADD	
	CRIP2	CREB1	
	CRIPT	CREB3L1	
	CRK	CREBBP	
	CRKL	CREBL2	
	CRLF1	CREBRF	
	CRLF3	CREM	
	CRLS1	CRH	
	CRMP1	CRIM1	
	CRTC2	CRK	
	CRY2	CRMP1	
	CS	CRTC1	
	CSDE1	CRTC2	
	CSE1L	CRTC3	

© 2023 Morton SU et al. *JAMA Network Open*.

	CSK	CRX	
	CSNK1A1L	CS	
	CSNK1D	CSDE1	
	CSNK1E	CSE1L	
	CSNK1G2	CSF1	
	CSNK1G3	CSF2	
	CSNK2A1	CSK	
	CSNK2A2	CSMD1	
	CSNK2B	CSNK1A1	
	CSRP1	CSNK1D	
	CSRP2	CSNK1E	
	CSRP2BP	CSNK1G1	
	CSTF1	CSNK1G2	
	CSTF2	CSNK1G3	
	CSTF2T	CSNK2A1	
	CSTF3	CSNK2A2	
	CTAGE1	CSNK2B	
	CTAGE5	CSRNP2	
	CTAGE8	CSRNP3	
	CTAGE9	CSTF1	
	CTBP1	CSTF2	
	CTBP2	CSTF3	
	CTC1	CTB-102L5.4	
	CTCF	CTBP1	
	CTDNEP1	CTC-273B12.7	
	CTDP1	CTC-432M15.3	
	CTDSP1	CTCF	
	CTDSP2	CTD-2260A17.2	
	CTGF	CTD-2545M3.6	
	CTNNA1	CTDNEP1	
	CTNNB1	CTDSP1	
	CTNNBL1	CTDSPL2	
	CTNND1	CTIF	
	CTPS1	CTLA4	
	CTR9	CTNNA1	
	CTSA	CTNNB1	
	CTSB	CTNND1	
	CTSD	CTNND2	
	CTSV	CTPS1	
	CTSZ	CTPS2	
	CTTN	CTR9	
	CTXN1	CUL1	
	CUEDC2	CUL2	
	CUL1	CUL3	
	CUL2	CUL4A	
	CUL3	CUL4B	
	CUL4A	CUL5	
	CUL4B	CUL9	
	CUL7	CUX1	
	CUTA	CUX2	
	CUX1	CWC15	

© 2023 Morton SU et al. *JAMA Network Open*.

	CWC15	CX3CL1	
	CXADR	CXCL13	
	CXCL12	CXCR5	
	CXCR2	CXorf21	
	CXCR4	CXorf22	
	CXorf38	CXorf23	
	CXXC1	CXorf30	
	CXXC5	CXorf40A	
	CYB5A	CXorf56	
	CYB5B	CXorf65	
	CYB5R3	CXXC1	
	CYC1	CXXC4	
	CYFIP1	CXXC5	
	CYHR1	CYB561	
	CYP51A1	CYBB	
	CYTH2	CYCS	
	DAAM1	CYFIP1	
	DAB1	CYFIP2	
	DAB2IP	CYLC1	
	DAG1	CYLD	
	DAK	CYP26B1	
	DALRD3	CYP46A1	
	DARS	CYR61	
	DAXX	CYTH1	
	DAZAP1	CYTH3	
	DAZAP2	DAAM1	
	DBF4	DAAM2	
	DBI	DAB1	
	DBN1	DAB2IP	
	DBNL	DACH1	
	DCAF11	DACT3	
	DCAF12	DAG1	
	DCAF13	DAGLA	
	DCAF15	DAPK1	
	DCAF7	DAZAP1	
	DCAF8	DAZL	
	DCAKD	DBF4	
	DCBLD2	DBN1	
	DCHS1	DCAF10	
	DCK	DCAF12L1	
	DCLRE1C	DCAF15	
	DCP1A	DCAF5	
	DCP2	DCAF7	
	DCPS	DCAF8	
	DCTN1	DCAF8	
	DCTN2	DCAF8L2	
	DCTN3	DCC	
	DCTN4	DCHS1	
	DCTN5	DCLK1	
	DCTN6	DCLK2	
	DCTPP1	DCLRE1B	

© 2023 Morton SU et al. *JAMA Network Open*.

	DCUN1D4	DCP1A	
	DCUN1D5	DCP2	
	DDA1	DCTN2	
	DDB1	DCTN4	
	DDI2	DCUN1D1	
	DDIT4	DCUN1D3	
	DDOST	DCUN1D5	
	DDR1	DDA1	
	DDX1	DDB1	
	DDX11	DDHD1	
	DDX17	DDI2	
	DDX18	DDIT3	
	DDX19A	DDR2	
	DDX20	DDX1	
	DDX21	DDX17	
	DDX23	DDX19A	
	DDX27	DDX21	
	DDX39A	DDX23	
	DDX39B	DDX24	
	DDX3X	DDX26B	
	DDX41	DDX27	
	DDX42	DDX39A	
	DDX46	DDX39B	
	DDX47	DDX3X	
	DDX49	DDX3Y	
	DDX5	DDX4	
	DDX52	DDX42	
	DDX54	DDX46	
	DDX56	DDX5	
	DDX6	DDX53	
	DEAF1	DDX6	
	DEGS1	DEDD	
	DEK	DEF6	
	DENND2A	DENND1A	
	DENND5A	DENND4A	
	DENR	DENND4B	
	DESI1	DENND5B	
	DGAT1	DENR	
	DGCR2	DERL2	
	DGCR8	DESI2	
	DGKD	DET1	
	DGKZ	DGCR8	
	DHCR24	DGKB	
	DHCR7	DGKI	
	DHDDS	DHX15	
	DHRS1	DHX30	
	DHX15	DHX37	
	DHX16	DHX40	
	DHX29	DHX9	
	DHX30	DIAPH1	
	DHX32	DIAPH2	

© 2023 Morton SU et al. *JAMA Network Open*.

	DHX33	DICER1	
	DHX34	DIDO1	
	DHX36	DIO1	
	DHX37	DIO2	
	DHX38	DIO3	
	DHX40	DIP2B	
	DHX8	DIP2C	
	DHX9	DIS3L2	
	DICER1	DKC1	
	DIP2B	DKFZP761J1410	
	DIS3	DKK2	
	DKC1	DLC1	
	DLAT	DLG1	
	DLD	DLG2	
	DLG1	DLG3	
	DLG3	DLG4	
	DLG4	DLG5	
	DLG5	DLGAP1	
	DLGAP5	DLGAP2	
	DLK1	DLGAP3	
	DLL1	DLGAP4	
	DLST	DLK2	
	DMAP1	DLL1	
	DMPK	DLL4	
	DMWD	DLX1	
	DNA2	DLX2	
	DNAAF2	DLX6	
	DNAJA1	DMBX1	
	DNAJA2	DMD	
	DNAJB1	DMRT1	
	DNAJB12	DMRTA2	
	DNAJC1	DMRTB1	
	DNAJC10	DMRTC2	
	DNAJC11	DMTF1	
	DNAJC13	DMTN	
	DNAJC2	DMXL1	
	DNAJC5	DMXL2	
	DNAJC7	DNAJA2	
	DNAJC8	DNAJB14	
	DNAJC9	DNAJB6	
	DNM1	DNAJC11	
	DNM1L	DNAJC13	
	DNM2	DNAJC14	
	DNMT1	DNAJC2	
	DNMT3A	DNAJC5	
	DNMT3B	DNAJC6	
	DNPEP	DNAJC7	
	DNPH1	DNM1	
	DNTTIP2	DNM2	
	DOC2A	DNMT1	
	DOCK7	DNTTIP1	

© 2023 Morton SU et al. *JAMA Network Open*.

	DOHH	DOCK2	
	DOLPP1	DOCK3	
	DONSON	DOCK4	
	DOT1L	DOCK9	
	DPF2	DOPEY1	
	DPM1	DOT1L	
	DPM2	DPF1	
	DPP3	DPF2	
	DPP8	DPP10	
	DPP9	DPP8	
	DPY19L1	DPP9	
	DPY30	DPYSL2	
	DPYSL2	DPYSL3	
	DPYSL3	DPYSL5	
	DPYSL4	DR1	
	DPYSL5	DRD1	
	DR1	DRD2	
	DRAP1	DSCAM	
	DRG1	DSCAML1	
	DRG2	DSE	
	DROSHA	DSG1	
	DSCR3	DSP	
	DSTN	DST	
	DTL	DSTN	
	DTX3	DSTYK	
	DTX4	DTNA	
	DTYMK	DTX3	
	DUS1L	DUSP10	
	DUS3L	DUSP4	
	DUSP11	DUSP6	
	DUSP6	DUSP7	
	DUSP7	DUSP8	
	DUSP9	DUSP9	
	DUT	DYNC1H1	
	DVL1	DYNC1LI1	
	DVL3	DYNC1LI2	
	DXO	DYNLL1	
	DYNC1H1	DYNLL2	
	DYNC1I2	DYNLT3	
	DYNC1LI1	DYRK1A	
	DYNC1LI2	DYRK1B	
	DYNLL2	DYRK2	
	DYNLRB1	E2F1	
	DYNLT1	E2F3	
	DYRK1A	E2F5	
	DYRK1B	E2F7	
	DZIP1	E2F8	
	E2F1	EBF1	
	E2F3	EBF2	
	E2F4	EBF3	
	E2F5	EBP	

© 2023 Morton SU et al. *JAMA Network Open*.

	E2F6	ECE1	
	E2F8	EDA	
	E4F1	EDF1	
	EAF1	EDNRA	
	EBNA1BP2	EEA1	
	ECD	EED	
	ECI2	EEF1A1	
	ECT2	EEF1A2	
	EDC3	EEF1G	
	EDC4	EEF2	
	EDF1	EFEMP1	
	EED	EFHC2	
	EEF1A1	EFNA5	
	EEF1B2	EFNB1	
	EEF1D	EFNB2	
	EEF1E1	EFTUD2	
	EEF1G	EGLN1	
	EEF2	EGLN2	
	EFNA2	EGR2	
	EFNA5	EGR3	
	EFNB1	EHD1	
	EFNB2	EHF	
	EFNB3	EHMT1	
	EFR3A	EI24	
	EFS	EIF1	
	EFTUD2	EIF1AX	
	EGFL7	EIF1AY	
	EGLN1	EIF1B	
	EHD1	EIF2B3	
	EHMT1	EIF2B4	
	EHMT2	EIF2S1	
	EI24	EIF2S2	
	EID1	EIF2S3	
	EIF1	EIF3A	
	EIF1AD	EIF3B	
	EIF1AX	EIF3D	
	EIF1B	EIF3F	
	EIF2A	EIF3G	
	EIF2AK1	EIF3I	
	EIF2B1	EIF3J	
	EIF2B2	EIF3L	
	EIF2B5	EIF4A1	
	EIF2D	EIF4A2	
	EIF2S1	EIF4A3	
	EIF2S2	EIF4B	
	EIF2S3	EIF4E	
	EIF3A	EIF4E2	
	EIF3B	EIF4ENIF1	
	EIF3C	EIF4G1	
	EIF3CL	EIF4G2	
	EIF3D	EIF4G3	

© 2023 Morton SU et al. *JAMA Network Open*.

	EIF3E	EIF5	
	EIF3F	EIF5A	
	EIF3G	EIF5B	
	EIF3H	ELAC1	
	EIF3I	ELAVL1	
	EIF3K	ELAVL2	
	EIF3L	ELAVL3	
	EIF4A2	ELAVL4	
	EIF4A3	ELF2	
	EIF4B	ELF3	
	EIF4E	ELFN1	
	EIF4E2	ELFN2	
	EIF4EBP1	ELK1	
	EIF4EBP2	ELL	
	EIF4ENIF1	ELL2	
	EIF4G1	ELMO1	
	EIF4G2	ELMOD1	
	EIF4G3	ELMSAN1	
	EIF4H	ELOVL1	
	EIF5	ELOVL4	
	EIF5A	ELOVL5	
	EIF5AL1	ELOVL6	
	EIF5B	EMC4	
	EIF6	EMC6	
	ELAC2	EMC7	
	ELAVL1	EMC8	
	ELF2	EMD	
	ELMO2	EML1	
	ELOF1	EMX1	
	ELOVL2	EMX2	
	ELOVL5	EN1	
	ELOVL6	EN2	
	ELP2	ENAH	
	ELP3	ENG	
	ELP5	ENY2	
	EMB	EOMES	
	EMC10	EP300	
	EMC2	EP400	
	EMC4	EPAS1	
	EMC6	EPB41L1	
	EMC7	EPB41L2	
	EMC8	EPB41L4B	
	EMD	EPC1	
	EME1	EPC2	
	EMILIN1	EPHA4	
	EML1	EPHA6	
	EML3	EPHA7	
	EML4	EPHB1	
	EN2	EPHB2	
	ENAH	EPM2AIP1	
	ENC1	EPN1	

© 2023 Morton SU et al. *JAMA Network Open*.

	ENG	EPN2	
	ENO1	EPS15L1	
	ENO3	EPS8	
	ENOPH1	EPT1	
	ENSA	ERBB2IP	
	ENY2	ERBB4	
	EP300	ERC2	
	EP400	ERCC6L	
	EPB41	ERF	
	EPB41L2	ERG	
	EPC1	ERGIC1	
	EPHB2	ERH	
	EPHB4	ERN1	
	EPN1	ERO1L	
	EPO	ERP29	
	EPRS	ERP44	
	EPS15L1	ESCO1	
	ERAL1	ESPL1	
	ERBB2	ESR1	
	ERBB2IP	ESRP1	
	ERC1	ESRRA	
	ERCC2	ESX1	
	ERCC3	ETF1	
	ERCC6L	ETNK1	
	ERCC6L2	ETS1	
	ERF	ETS2	
	ERGIC1	ETV1	
	ERGIC2	ETV3	
	ERGIC3	ETV5	
	ERH	ETV6	
	ERI1	EVA1B	
	ERLIN2	EVI5L	
	ERO1L	EVL	
	ESF1	EWSR1	
	ESPL1	EXOC3	
	ESYT2	EXOC3L2	
	ETF1	EXOC5	
	ETFB	EXOC8	
	ETV6	EXOSC6	
	EVL	EXT1	
	EWSR1	EYA1	
	EXD2	EYA3	
	EXOC3	EZH2	
	EXOC5	F8	
	EXOSC10	F9	
	EXOSC2	FADD	
	EXOSC4	FADS2	
	EXOSC7	FAF1	
	EXOSC8	FAF2	
	EXOSC9	FAM115A	
	EXT1	FAM120A	

© 2023 Morton SU et al. *JAMA Network Open*.

	EXT2	FAM120C	
	EXTL3	FAM122B	
	EYA2	FAM131B	
	EYA3	FAM135A	
	EZH2	FAM135B	
	EZR	FAM155A	
	F2R	FAM155B	
	FADS1	FAM160A2	
	FADS2	FAM160B1	
	FAF2	FAM168A	
	FAM107B	FAM169A	
	FAM109A	FAM171A1	
	FAM114A2	FAM171A2	
	FAM115A	FAM193A	
	FAM120A	FAM193B	
	FAM127A	FAM196A	
	FAM127B	FAM196B	
	FAM127C	FAM199X	
	FAM134A	FAM208A	
	FAM134C	FAM211A	
	FAM136A	FAM214B	
	FAM149B1	FAM222A	
	FAM162A	FAM229B	
	FAM168A	FAM3A	
	FAM168B	FAM46A	
	FAM171A1	FAM46C	
	FAM171A2	FAM46D	
	FAM172A	FAM49B	
	FAM178A	FAM50A	
	FAM192A	FAM53B	
	FAM193A	FAM57B	
	FAM193B	FAM58A	
	FAM195B	FAM60A	
	FAM206A	FAM65A	
	FAM213A	FAM65B	
	FAM216A	FAM69A	
	FAM21A	FAM83H	
	FAM21B	FAM84A	
	FAM21C	FAM98A	
	FAM222B	FANCB	
	FAM32A	FAR1	
	FAM49B	FAR2	
	FAM53A	FAS	
	FAM53C	FASN	
	FAM57A	FAT3	
	FAM63B	FAT4	
	FAM64A	FAU	
	FAM65A	FAXC	
	FAM76B	FBL	
	FAM91A1	FBLN1	
	FAM96A	FBLN5	

© 2023 Morton SU et al. *JAMA Network Open*.

	FAM98A	FBN1	
	FAM98B	FBN2	
	FAR1	FBRS	
	FARP1	FBXL14	
	FARSA	FBXL16	
	FARSB	FBXL17	
	FASN	FBXL19	
	FASTK	FBXL20	
	FAT1	FBXL3	
	FBF1	FBXL5	
	FBLN1	FBXO11	
	FBN2	FBXO15	
	FBRS	FBXO21	
	FBXL14	FBXO22	
	FBXL19	FBXO28	
	FBXL6	FBXO3	
	FBXO11	FBXO32	
	FBXO18	FBXO33	
	FBXO21	FBXO38	
	FBXO22	FBXO41	
	FBXO38	FBXO42	
	FBXO42	FBXO45	
	FBXO45	FBXO46	
	FBXO5	FBXO5	
	FBXW11	FBXW11	
	FBXW5	FBXW2	
	FBXW8	FBXW7	
	FCF1	FCER1G	
	FCHSD2	FCHO1	
	FECH	FCHO2	
	FEM1B	FCHSD2	
	FEM1C	FEM1C	
	FEN1	FER	
	FERMT2	FERMT2	
	FEZ1	FEZ1	
	FGD1	FEZF1	
	FGF19	FEZF2	
	FGFR1	FGB	
	FGFR1OP	FGD1	
	FGFR1OP2	FGD5	
	FGFR2	FGF10	
	FGFRL1	FGF12	
	FH	FGF13	
	FHL1	FGF14	
	FHOD3	FGF16	
	FIP1L1	FGF17	
	FIS1	FGF19	
	FIZ1	FGF20	
	FKBP10	FGF7	
	FKBP1A	FGF8	
	FKBP3	FGF9	

© 2023 Morton SU et al. *JAMA Network Open*.

	FKBP4	FGFBP1	
	FKBP5	FGFR1	
	FKBP8	FGFR2	
	FKBP9	FHL1	
	FLAD1	FIGN	
	FLII	FIP1L1	
	FLNA	FIZ1	
	FLNB	FJX1	
	FLOT2	FKBP1A	
	FLRT2	FKBP5	
	FLRT3	FKBP8	
	FLYWCH1	FLCN	
	FMNL2	FLI1	
	FN1	FLNA	
	FNBP1L	FLNC	
	FNBP4	FLRT2	
	FNDC3A	FLT1	
	FNDC4	FLT3	
	FNTA	FLT3LG	
	FNTB	FLT4	
	FOCAD	FMN2	
	FOXK1	FMNL1	
	FOXK2	FMNL2	
	FOXM1	FMR1	
	FOXP4	FNBP1	
	FPGS	FNBP1L	
	FRAT1	FNBP4	
	FREM2	FNDC3A	
	FRMD8	FNDC3B	
	FRS2	FNDC4	
	FSCN1	FNDC5	
	FSTL1	FNIP1	
	FTH1	FNIP2	
	FTL	FNTA	
	FTSJ3	FOSB	
	FUBP1	FOSL2	
	FUBP3	FOXA2	
	FURIN	FOXA3	
	FUS	FOXB1	
	FXR1	FOXC1	
	FXR2	FOXD1	
	FXYD6	FOXD3	
	FYN	FOXE1	
	FYTTD1	FOXF1	
	FZD1	FOXF2	
	FZD10	FOXG1	
	FZD2	FOXI1	
	FZD3	FOXJ1	
	FZD7	FOXJ2	
	FZR1	FOXJ3	
	G3BP1	FOXK1	

© 2023 Morton SU et al. *JAMA Network Open*.

	G3BP2	FOXL2	
	G6PC3	FOXN1	
	G6PD	FOXN3	
	GAB1	FOXN4	
	GABARAP	FOXO1	
	GABPA	FOXO3	
	GAK	FOXO4	
	GALK1	FOXP1	
	GALNT1	FOXP2	
	GALNT16	FOXP3	
	GALNT2	FOXP4	
	GANAB	FOXQ1	
	GAP43	FRMD4A	
	GAPDH	FRMD5	
	GAPVD1	FRMD7	
	GAR1	FRMPD4	
	GARS	FRS2	
	GART	FRS3	
	GAS1	FRY	
	GATAD1	FRYL	
	GATAD2A	FSCN1	
	GATAD2B	FST	
	GBA2	FSTL1	
	GBF1	FSTL3	
	GCLC	FTSJ1	
	GCN1L1	FUBP1	
	GCSH	FUBP3	
	GDE1	FURIN	
	GDI1	FUS	
	GDI2	FXR1	
	GEMIN4	FXR2	
	GEMIN5	FXYD6	
	GEMIN7	FXYD6-FXYD2	
	GET4	FYN	
	GFM1	FZD2	
	GGA1	FZD4	
	GGA2	FZD5	
	GGA3	FZD8	
	GGNBP2	FZR1	
	GHITM	G2E3	
	GID4	G3BP2	
	GIGYF1	G6PD	
	GINS1	GAB2	
	GINS2	GAB3	
	GINS4	GABARAPL1	
	GIPC1	GABBR1	
	GIT1	GABBR2	
	GJA1	GABPA	
	GJC1	GABPB1	
	GLDC	GABRA1	
	GLE1	GABRA2	

© 2023 Morton SU et al. *JAMA Network Open*.

	GLG1	GABRA4	
	GLI1	GABRA5	
	GLI2	GABRB1	
	GLI3	GABRB2	
	GLIS2	GABRB3	
	GLO1	GABRD	
	GLOD4	GABRG2	
	GLRX5	GABRG3	
	GLS	GAD2	
	GLTP	GADD45G	
	GLTSCR1	GAL3ST1	
	GLTSCR2	GALNT13	
	GLUD1	GANAB	
	GLUD2	GAPVD1	
	GLUL	GAS1	
	GLYR1	GAS2L1	
	GMFB	GAS7	
	GMNN	GATA1	
	GMPPA	GATA2	
	GMPR2	GATA3	
	GMPS	GATA6	
	GNA11	GATAD2A	
	GNA12	GATAD2B	
	GNA13	GBX2	
	GNAI2	GCH1	
	GNAI3	GCK	
	GNAQ	GCLM	
	GNAS	GCM1	
	GNB1	GCN1L1	
	GNB2	GDA	
	GNB2L1	GDF11	
	GNG2	GDF5	
	GNG5	GDF6	
	GNL1	GDF7	
	GNL2	GDI1	
	GNL3	GDI2	
	GNL3L	GEMIN7	
	GNPAT	GEMIN8	
	GNPDA1	GET4	
	GNS	GFPT1	
	GOLGA2	GFY	
	GOLGA6A	GGA1	
	GOLGA6B	GGH	
	GOLGA6C	GGNBP1	
	GOLGA6D	GGNBP2	
	GOLGA6L1	GGT7	
	GOLGA6L10	GID4	
	GOLGA6L6	GID8	
	GOLGA6L9	GIGYF2	
	GOLGA8A	GIT1	
	GOLGA8B	GIT2	

© 2023 Morton SU et al. *JAMA Network Open*.

	GOLGA8F	GJB1	
	GOLGA8G	GJC1	
	GOLM1	GK	
	GOLPH3	GLA	
	GOLPH3L	GLCE	
	GOLT1B	GLG1	
	GON4L	GLI2	
	GORASP2	GLI3	
	GOSR2	GLIS2	
	GOT1	GLRA2	
	GOT2	GLRX5	
	GPAA1	GLS	
	GPALPP1	GLTSCR1	
	GPAM	GLTSCR1L	
	GPATCH4	GLUL	
	GPATCH8	GLYR1	
	GPBP1	GMDS	
	GPC1	GMEB1	
	GPC2	GMEB2	
	GPC3	GMPS	
	GPC4	GNA11	
	GPI	GNA12	
	GPM6B	GNA13	
	GPN1	GNAI1	
	GPN3	GNAI2	
	GPR107	GNAL	
	GPR124	GNAO1	
	GPR125	GNAQ	
	GPR56	GNAS	
	GPRASP1	GNAZ	
	GPRC5B	GNB1	
	GPRC5C	GNB2	
	GPS1	GNB2L1	
	GPS2	GNG11	
	GPSM1	GNG12	
	GPSM2	GNG2	
	GPX1	GNG3	
	GPX3	GNL1	
	GRAMD1A	GNL3L	
	GRB10	GOLGA2	
	GRB2	GOLGA6L18	
	GREB1	GOLT1B	
	GRIK5	GON4L	
	GRINA	GORASP2	
	GRK6	GP1BB	
	GRN	GPATCH8	
	GRSF1	GPC3	
	GRWD1	GPC4	
	GSE1	GPER1	
	GSK3A	GPHN	
	GSK3B	GPKOW	

© 2023 Morton SU et al. *JAMA Network Open*.

	GSPT1	GPM6A	
	GSTA4	GPR101	
	GSTM3	GPR137	
	GSTP1	GPR143	
	GTF2B	GPR173	
	GTF2E1	GPR26	
	GTF2E2	GPR50	
	GTF2F1	GPR61	
	GTF2H1	GPR64	
	GTF2H3	GPR85	
	GTF2H4	GPR88	
	GTF2I	GPRASP2	
	GTF2IRD1	GPRIN1	
	GTF3A	GPS1	
	GTF3C1	GRAMD1B	
	GTF3C2	GRAMD4	
	GTF3C3	GRASP	
	GTF3C4	GRB10	
	GTF3C5	GRB2	
	GTF3C6	GREB1L	
	GTPBP1	GRHL2	
	GTPBP2	GRHL3	
	GTPBP4	GRIA1	
	GTSE1	GRIA2	
	GUCD1	GRIA3	
	GUK1	GRID1	
	GYG1	GRID2	
	GYLTL1B	GRIK2	
	GYS1	GRIK3	
	GZF1	GRIK5	
	H1FX	GRIN1	
	H2AFV	GRIN2A	
	H2AFX	GRIN2B	
	H2AFY	GRIN2D	
	H2AFY2	GRIP1	
	H2AFZ	GRIPAP1	
	H3F3A	GRK5	
	H3F3B	GRM3	
	HADH	GRM4	
	HADHA	GRM5	
	HAPLN1	GRM7	
	HARS	GSE1	
	HAT1	GSG1L	
	HAUS2	GSK3A	
	HAUS6	GSK3B	
	HAUS7	GSPT1	
	HBA1	GSPT2	
	HBA2	GSTT2B	
	HBE1	GTF2A1	
	HBG1	GTF2E1	
	HBG2	GTF2H1	

© 2023 Morton SU et al. *JAMA Network Open*.

	HBP1	GTF2I	
	HBS1L	GTF2IRD1	
	HBZ	GTF3C1	
	HCFC1	GTF3C2	
	HDAC2	GTPBP1	
	HDAC3	GTPBP4	
	HDAC6	GULP1	
	HDAC7	H1F0	
	HDDC3	H2AFY	
	HDGFRP2	H2AFY2	
	HDLBP	H2AFZ	
	HEATR1	H3F3B	
	HEATR2	HAPLN1	
	HEATR3	HAS2	
	HECTD1	HAS3	
	HECTD4	HAT1	
	HELLS	HAUS7	
	HELZ	HBG1	
	HERC2	HBG2	
	HES1	HCCS	
	HES5	HCFC1	
	HES6	HCFC2	
	HGS	HCN1	
	HIAT1	HCN4	
	HIBADH	HDAC1	
	HIC2	HDAC2	
	HIF1A	HDAC3	
	HIF1AN	HDAC4	
	HIGD2A	HDAC5	
	HINT1	HDAC6	
	HIP1	HDAC7	
	HIPK1	HDAC8	
	HIPK2	HDAC9	
	HIRA	HDGFRP2	
	HIRIP3	HDGFRP3	
	HIST3H2A	HDLBP	
	HJURP	HEATR1	
	HK2	HECA	
	HMBS	HECTD1	
	HMG20B	HECTD2	
	HMGA1	HECTD4	
	HMGA2	HECW1	
	HMGB1	HECW2	
	HMGB2	HELLS	
	HMGB3	HELZ	
	HMGCR	HEPACAM	
	HMGN1	HERC1	
	HMGN2	HERC2	
	HMGN4	HERC4	
	HMGXB3	HERPUD1	
	HMGXB4	HERPUD2	

© 2023 Morton SU et al. *JAMA Network Open*.

	HMMR	HES1	
	HMOX2	HES7	
	HN1	HEXIM1	
	HN1L	HEY2	
	HNRNPA0	HGF	
	HNRNPA1	HHEX	
	HNRNPA2B1	HHIP	
	HNRNPA3	HIAT1	
	HNRNPAB	HIC2	
	HNRNPC	HIF1A	
	HNRNPD	HIP1	
	HNRNPDL	HIPK1	
	HNRNPF	HIPK2	
	HNRNPH1	HIRA	
	HNRNPH2	HIST1H1B	
	HNRNPH3	HIST1H2AC	
	HNRNPK	HIST1H3J	
	HNRNPL	HIST2H2AB	
	HNRNPLL	HIST2H2BE	
	HNRNPM	HIST3H2A	
	HNRNPR	HIVEP1	
	HNRNPU	HIVEP2	
	HNRNPUL1	HIVEP3	
	HNRNPUL2	HK1	
	HNRNPUL2-BSCL2	HLA-DRA	
	HOMER3	HLF	
	HP1BP3	HLX	
	HPRT1	HM13	
	HS2ST1	HMBOX1	
	HS6ST1	HMBS	
	HSBP1	HMG20A	
	HSD11B2	HMG20B	
	HSD17B10	HMGA1	
	HSD17B12	HMGA2	
	HSD17B4	HMGB1	
	HSD17B8	HMGB2	
	HSD3B7	HMGB3	
	HSDL1	HMGCR	
	HSF1	HMGCS1	
	HSP90AA1	HMGN2	
	HSP90AB1	HMGXB3	
	HSP90B1	HMX1	
	HSPA4	HMX3	
	HSPA5	HNF1A	
	HSPA8	HNF1B	
	HSPA9	HNRNPA0	
	HSPBP1	HNRNPA1	
	HSPD1	HNRNPA2B1	
	HSPE1	HNRNPA3	
	HSPG2	HNRNPAB	
	HSPH1	HNRNPC	

© 2023 Morton SU et al. *JAMA Network Open*.

	HTATSF1	HNRNPD	
	HTRA2	HNRNPF	
	HTT	HNRNPH1	
	HUNK	HNRNPH2	
	HUWE1	HNRNPH3	
	HYAL2	HNRNPK	
	HYOU1	HNRNPL	
	HYPK	HNRNPLL	
	IARS	HNRNPM	
	IARS2	HNRNPR	
	ICMT	HNRNPU	
	ICT1	HNRNPUL1	
	ID1	HNRNPUL2	
	ID2	HOMER1	
	ID3	HOMER3	
	ID4	HOOK3	
	IDE	HORMAD1	
	IDH1	HOXA10	
	IDH2	HOXA11	
	IDH3A	HOXA13	
	IDH3B	HOXA3	
	IDH3G	HOXB3	
	IDI1	HOXB5	
	IER2	HOXB8	
	IER5L	HOXC6	
	IFNAR1	HP1BP3	
	IFRD1	HPCA	
	IFRD2	HPCAL1	
	IFRG15	HPN	
	IFT140	HPRT1	
	IFT172	HS2ST1	
	IFT52	HS3ST4	
	IFT74	HS6ST1	
	IGDCC3	HS6ST3	
	IGF1R	HSD17B10	
	IGF2	HSF1	
	IGF2BP1	HSF2	
	IGF2BP2	HSF5	
	IGF2BP3	HSP90AA1	
	IGF2R	HSP90AB1	
	IGFBP2	HSP90B1	
	IGFBP3	HSPA12A	
	IGFBP4	HSPA14	
	IGFBP5	HSPA4	
	IGSF9	HSPA5	
	IK	HSPA8	
	IKBKAP	HSPA9	
	IKBKB	HSPD1	
	IL17RD	HSPE1	
	IL6ST	HSPE1-MOB4	
	ILDR2	HSPH1	

© 2023 Morton SU et al. *JAMA Network Open*.

	ILF2	HTATSF1	
	ILF3	HTR2A	
	ILK	HTT	
	IMMT	HUNK	
	IMP4	HUWE1	
	IMPA1	HVCN1	
	IMPACT	HYOU1	
	IMPAD1	ICMT	
	IMPDH1	ID2	
	INA	ID3	
	INCENP	IDH2	
	ING1	IDS	
	ING4	IDS	
	INIP	IER2	
	INO80B	IER3	
	INPP5E	IER5	
	INPPL1	IER5L	
	INTS1	IFFO2	
	INTS2	IFNGR2	
	INTS3	IGBP1	
	INTS4	IGF1R	
	INTS5	IGF2BP1	
	INTS7	IGF2BP3	
	INTS8	IGF2R	
	IP6K1	IGFBP3	
	IP6K2	IGFBP5	
	IPO11	IGSF1	
	IPO13	IGSF9B	
	IPO4	IKBKB	
	IPO5	IKZF1	
	IPO7	IKZF2	
	IPO9	IKZF3	
	IQGAP3	IKZF4	
	IQSEC1	IKZF5	
	IRAK1	IL13RA1	
	IREB2	IL15	
	IRF2BP1	IL1RAPL1	
	IRS2	IL1RAPL2	
	IRS4	IL21	
	IRX3	IL21R	
	ISCA2	IL27	
	ISG20L2	IL2RG	
	ISOC1	IL34	
	IST1	IL6ST	
	ISY1	IL7	
	ISY1-RAB43	ILDR2	
	ISYNA1	ILF2	
	ITFG1	ILF3	
	ITFG3	ILKAP	
	ITGA5	INCENP	
	ITGB1	INF2	

© 2023 Morton SU et al. *JAMA Network Open*.

	ITGB5	ING2	
	ITM2A	ING3	
	ITM2B	ING5	
	ITM2C	INHBA	
	ITSN1	INHBB	
	IVD	INO80	
	IVNS1ABP	INO80B	
	JAG1	INO80D	
	JAGN1	INPP4A	
	JAK1	INPP4B	
	JAM3	INPP5A	
	JARID2	INPP5D	
	JKAMP	INSM2	
	JMJD6	INTS10	
	JMJD8	INTS12	
	JMY	INTS2	
	JUND	INTS3	
	JUP	INTS5	
	KANK2	INTS6	
	KANSL1	INTS8	
	KANSL2	IP6K1	
	KANSL3	IP6K2	
	KARS	IPMK	
	KAT2A	IPO13	
	KAT5	IPO5	
	KAT6A	IPO7	
	KAT7	IPO9	
	KATNA1	IPPK	
	KBTBD2	IQGAP1	
	KBTBD4	IQSEC1	
	KCMF1	IQSEC2	
	KCTD10	IRAK1	
	KCTD15	IREB2	
	KCTD17	IRF1	
	KCTD20	IRF2	
	KCTD5	IRF2BP1	
	KDELC1	IRF2BP2	
	KDELR1	IRF2BPL	
	KDELR2	IRF4	
	KDM1A	IRF6	
	KDM2A	IRF8	
	KDM3A	IRF9	
	KDM3B	IRS2	
	KDM4A	IRS4	
	KDM4B	IRX3	
	KDM5B	IRX5	
	KDM5C	ISL1	
	KDM6B	ISY1	
	KHDRBS1	ISY1-RAB43	
	KHDRBS3	ITCH	
	KHNYN	ITGB1	

© 2023 Morton SU et al. *JAMA Network Open*.

	KHSRP	ITGB8	
	KIAA0020	ITM2B	
	KIAA0100	ITM2C	
	KIAA0101	ITPK1	
	KIAA0195	ITPKA	
	KIAA0196	ITPKB	
	KIAA0226	ITPR1	
	KIAA0355	ITSN1	
	KIAA0368	IVNS1ABP	
	KIAA0430	IWS1	
	KIAA0895L	JADE1	
	KIAA0907	JADE2	
	KIAA0922	JADE3	
	KIAA0930	JAG1	
	KIAA0947	JAG2	
	KIAA1191	JAK1	
	KIAA1211	JAK2	
	KIAA1217	JAKMIP1	
	KIAA1279	JAKMIP2	
	KIAA1328	JARID2	
	KIAA1429	JAZF1	
	KIAA1432	JMJD1C	
	KIAA1524	JMJD6	
	KIAA1549	JMY	
	KIAA1551	JPH1	
	KIAA1715	JPH3	
	KIAA1731	JPH4	
	KIAA2013	JUNB	
	KIF11	JUND	
	KIF13B	KAL1	
	KIF15	KALRN	
	KIF18B	KANSL1	
	KIF1A	KANSL1L	
	KIF1B	KANSL2	
	KIF20A	KANSL3	
	KIF21A	KAT2A	
	KIF21B	KAT2B	
	KIF22	KAT6A	
	KIF23	KAT6B	
	KIF2A	KAT7	
	KIF2C	KATNAL1	
	KIF3A	KATNBL1	
	KIF4A	KAZN	
	KIF4B	KBTBD2	
	KIF5B	KBTBD6	
	KIF5C	KCMF1	
	KIFAP3	KCNA2	
	KIFC1	KCNA3	
	KIRREL	KCNA4	
	KLC1	KCNAB2	
	KLC2	KCNB1	

© 2023 Morton SU et al. *JAMA Network Open*.

	KLF7	KCNB2	
	KLHDC10	KCNC1	
	KLHDC2	KCND3	
	KLHDC3	KCNG2	
	KLHL13	KCNH1	
	KLHL17	KCNH2	
	KLHL7	KCNH3	
	KLHL9	KCNH4	
	KM-PA-2	KCNIP4	
	KMT2A	KCNJ3	
	KMT2B	KCNJ4	
	KMT2D	KCNJ6	
	KMT2E	KCNK12	
	KNOP1	KCNK3	
	KNSTRN	KCNK9	
	KPNA1	KCNMA1	
	KPNA2	KCNMB2	
	KPNA3	KCNMB4	
	KPNA4	KCNN1	
	KPNA6	KCNN2	
	KPNB1	KCNN3	
	KRAS	KCNQ2	
	KRBA2	KCNQ3	
	KRI1	KCNQ5	
	KRTCAP2	KCNV1	
	KTI12	KCTD1	
	KTN1	KCTD10	
	L3MBTL2	KCTD16	
	LAMA1	KDELR1	
	LAMB1	KDM1A	
	LAMB2	KDM2A	
	LAMC1	KDM2B	
	LAMP1	KDM3A	
	LAMTOR2	KDM3B	
	LAMTOR3	KDM4A	
	LAP3	KDM4B	
	LAPTM4A	KDM4E	
	LAPTM4B	KDM5A	
	LARP1	KDM5C	
	LARP7	KDM5D	
	LARS	KDM6A	
	LARS2	KDM6B	
	LAS1L	KDM7A	
	LASP1	KDR	
	LBH	KHDRBS1	
	LBR	KHSRP	
	LDB1	KIAA0232	
	LDHA	KIAA0247	
	LDLRAD3	KIAA0355	
	LEMD2	KIAA0368	
	LENG8	KIAA0430	

© 2023 Morton SU et al. *JAMA Network Open*.

	LEO1	KIAA0907	
	LEPROT	KIAA0922	
	LETM1	KIAA0947	
	LFNG	KIAA1211L	
	LGALSL	KIAA1239	
	LHX2	KIAA1244	
	LIG1	KIAA1429	
	LIG3	KIAA1468	
	LIMA1	KIAA1549	
	LIMD1	KIAA1671	
	LIMD2	KIAA2018	
	LIMS1	KIAA2022	
	LIMS3	KIAA2026	
	LIMS3L	KIF11	
	LIN28A	KIF13A	
	LIN28B	KIF1A	
	LIN54	KIF1B	
	LIN7C	KIF1C	
	LITAF	KIF23	
	LIX1L	KIF26B	
	LLGL1	KIF2A	
	LLPH	KIF3A	
	LMAN1	KIF3C	
	LMAN2	KIF4A	
	LMF2	KIF5A	
	LMNB1	KIF5B	
	LMNB2	KIF5C	
	LMO4	KIRREL	
	LONP1	KIRREL3	
	LOXL2	KIT	
	LPAR1	KITLG	
	LPCAT1	KLC2	
	LPCAT3	KLF12	
	LPHN1	KLF2	
	LPHN2	KLF3	
	LPPR3	KLF4	
	LRFN4	KLF5	
	LRIG1	KLF6	
	LRP1	KLF7	
	LRP10	KLF9	
	LRP3	KLHDC10	
	LRP5	KLHDC3	
	LRP6	KLHL10	
	LRPPRC	KLHL15	
	LRRC40	KLHL2	
	LRRC41	KLHL29	
	LRRC42	KLHL3	
	LRRC47	KLHL36	
	LRRC4B	KLHL4	
	LRRC58	KLHL9	
	LRRC59	KM-PA-2	

© 2023 Morton SU et al. *JAMA Network Open*.

	LRRC8A	KMT2A	
	LRRN1	KMT2B	
	LRWD1	KMT2C	
	LSG1	KMT2D	
	LSM12	KMT2E	
	LSM14A	KPNA1	
	LSM14B	KPNA2	
	LSM3	KPNA3	
	LSM4	KPNA4	
	LSR	KPNA6	
	LSS	KPNB1	
	LTN1	KRBOX4	
	LTV1	KRT1	
	LUC7L	KRT18	
	LUC7L3	KRT5	
	LUZP1	KRTAP17-1	
	LY6E	KRTAP6-3	
	LYAR	KSR2	
	LYPLA1	L1CAM	
	LYPLA2	L3MBTL3	
	LYPLAL1	LAMC1	
	LZTR1	LAMP1	
	LZTS2	LAMTOR1	
	M6PR	LAMTOR3	
	MAD1L1	LAPTM5	
	MAD2L1	LARGE	
	MAEA	LARP1	
	MAF1	LARP4	
	MAFG	LARP4B	
	MAGED1	LARP6	
	MAGED2	LAS1L	
	MAGOH	LASP1	
	MAGT1	LATS1	
	MAK16	LATS2	
	MAML1	LBX1	
	MAN1B1	LCK	
	MAN2A2	LCOR	
	MAN2B2	LCORL	
	MAN2C1	LCP1	
	MANF	LCP2	
	MAP1B	LDB1	
	MAP1LC3B2	LDB2	
	MAP1S	LDLRAD4	
	MAP2K1	LDOC1	
	MAP2K2	LEF1	
	MAP2K3	LEMD2	
	MAP2K4	LEMD3	
	MAP2K5	LENG8	
	MAP2K6	LEO1	
	MAP2K7	LEPR	
	MAP3K11	LEPROTL1	

© 2023 Morton SU et al. *JAMA Network Open*.

	MAP3K12	LGI1	
	MAP3K3	LHFP	
	MAP3K7	LHFPL3	
	MAP4	LHFPL4	
	MAP4K4	LHX2	
	MAP7	LHX5	
	MAP7D1	LHX6	
	MAPK1	LHX8	
	MAPK11	LHX9	
	MAPK14	LIF	
	MAPK1IP1L	LIMCH1	
	MAPK3	LIMK1	
	MAPK6	LIN28B	
	MAPK7	LIN52	
	MAPK8IP1	LIN54	
	MAPK8IP3	LIN7B	
	MAPKAP1	LIN9	
	MAPKAPK2	LINGO1	
	MAPRE1	LINGO2	
	MAPRE2	LIPT2	
	5-Mar	LMNA	
	6-Mar	LMNB1	
	MARCKS	LMNB2	
	MARCKSL1	LMO1	
	MARK2	LMO3	
	MARK3	LMO4	
	MARK4	LMOD1	
	MARS	LMTK2	
	MASP2	LMTK3	
	MAST2	LMX1A	
	MAT2A	LMX1B	
	MAT2B	LNPEP	
	MATR3	LOH12CR2	
	MAU2	LONP1	
	MAZ	LONRF1	
	MBD1	LOX	
	MBD3	LOXL1	
	MBD6	LPAR3	
	MBTD1	LPCAT3	
	MBTPS1	LPGAT1	
	MCFD2	LPHN1	
	MCL1	LPHN2	
	MCM10	LPHN3	
	MCM2	LPPR1	
	MCM3	LPPR4	
	MCM3AP	LRCH1	
	MCM4	LRCH2	
	MCM5	LRFN1	
	MCM6	LRFN3	
	MCM7	LRFN4	
	MCMBP	LRFN5	

© 2023 Morton SU et al. *JAMA Network Open*.

	MCRS1	LRP1	
	MCTS1	LRP12	
	MDC1	LRP2	
	MDH1	LRP5	
	MDH2	LRP6	
	MDK	LRP8	
	ME2	LRRC1	
	MEA1	LRRC25	
	MEAF6	LRRC4	
	MED1	LRRC41	
	MED12	LRRC47	
	MED16	LRRC4B	
	MED19	LRRC4C	
	MED20	LRRC7	
	MED21	LRRC8A	
	MED22	LRRC8B	
	MED23	LRRC8D	
	MED24	LRRN1	
	MED25	LRRN2	
	MED4	LRRTM1	
	MED6	LRRTM2	
	MED8	LRRTM3	
	MEGF6	LRTM2	
	MEGF8	LSAMP	
	MEIS2	LSM11	
	MELK	LSM12	
	MEMO1	LSM14B	
	MEN1	LSM6	
	MEPCE	LTBP3	
	MESDC2	LUC7L3	
	MEST	LUZP1	
	METAP2	LYL1	
	METRN	LYN	
	METTL13	LYPLA2	
	METTL16	LYZL4	
	METTL17	LZTS1	
	METTL23	LZTS3	
	METTL2A	MACF1	
	METTL2B	MACROD1	
	METTL3	MADCAM1	
	METTL9	MAF	
	MEX3A	MAF1	
	MEX3B	MAFB	
	MEX3C	MAFG	
	MEX3D	MAFK	
	MFAP1	MAGEA11	
	MFAP2	MAGED1	
	MFAP3	MAGED2	
	MFAP4	MAGEE1	
	MFF	MAGEL2	
	MFGE8	MAGI1	

© 2023 Morton SU et al. *JAMA Network Open*.

	MFN2	MAGI2	
	MFSD1	MAGOH	
	MFSD10	MAGT1	
	MFSD5	MAK16	
	MGAT4B	MAL	
	MGEA5	MALT1	
	MGRN1	MAML1	
	MIB1	MAML2	
	MICU2	MAML3	
	MID1	MAMLD1	
	MIDN	MAN1A2	
	MIEF1	MAOA	
	MIER2	MAOB	
	MIF	MAP1A	
	MINK1	MAP1B	
	MINPP1	MAP2	
	MIR124-2	MAP2K1	
	MIS12	MAP2K2	
	MIS18A	MAP2K4	
	MIS18BP1	MAP2K6	
	MKI67	MAP2K7	
	MKL1	MAP3K1	
	MKNK2	MAP3K10	
	MLEC	MAP3K12	
	MLF2	MAP3K13	
	MLLT1	MAP3K2	
	MLLT10	MAP3K3	
	MLLT4	MAP3K4	
	MLXIP	MAP3K5	
	MMACHC	MAP3K7	
	MMADHC	MAP3K8	
	MMD	MAP4	
	MMGT1	MAP4K1	
	MMP14	MAP4K4	
	MMP15	MAP4K5	
	MMP2	MAP7D1	
	MMS19	MAPK1	
	MMS22L	MAPK1IP1L	
	MN1	MAPK6	
	MOB1A	MAPK8	
	MOB3A	MAPK8IP1	
	MOB4	MAPK8IP2	
	MOGS	MAPK8IP3	
	MORC2	MAPKAP1	
	MORF4L2	MAPKAPK2	
	MPDU1	MAPRE1	
	MPDZ	MAPRE2	
	MPHOSPH10	MAPRE3	
	MPHOSPH8	MARK1	
	MPI	MARK2	
	MPND	MARK4	

© 2023 Morton SU et al. *JAMA Network Open*.

	MPP2	MAST1	
	MPP6	MAST3	
	MPRIP	MAT2A	
	MPV17	MATR3	
	MPZL1	MAU2	
	MRC2	MAX	
	MRGBP	MAZ	
	MRPL10	MBD1	
	MRPL12	MBD3	
	MRPL18	MBD5	
	MRPL2	MBD6	
	MRPL20	MBNL1	
	MRPL22	MBNL3	
	MRPL24	MBOAT2	
	MRPL28	MBTD1	
	MRPL30	MBTPS2	
	MRPL34	MCF2	
	MRPL37	MCL1	
	MRPL38	MCM6	
	MRPL39	MCMBP	
	MRPL4	MCTS1	
	MRPL40	MCU	
	MRPL45	MDGA1	
	MRPL49	MDGA2	
	MRPL50	MDGA2	
	MRPL54	MDH1	
	MRPL9	MDM2	
	MRPS10	MDM4	
	MRPS15	MECOM	
	MRPS17	MECP2	
	MRPS18B	MED1	
	MRPS2	MED12	
	MRPS21	MED12L	
	MRPS27	MED13	
	MRPS30	MED13L	
	MRPS35	MED14	
	MRPS7	MED15	
	MSANTD2	MED19	
	MSANTD3	MED26	
	MSANTD3-TMEFF1	MEF2A	
	MSANTD4	MEF2D	
	MSH2	MEGF9	
	MSH6	MEIS1	
	MSI1	MEIS2	
	MSI2	MEMO1	
	MSL1	MEN1	
	MSL2	MEOX2	
	MSL3	MEPCE	
	MSMP	MESDC1	
	MSN	MESDC2	
	MSTO1	MET	

© 2023 Morton SU et al. *JAMA Network Open*.

	MSX1	METRNL	
	MTA1	METTL16	
	MTA2	METTL9	
	MTAP	MEX3A	
	MTCH1	MEX3B	
	MTCH2	MEX3C	
	MTDH	MEX3D	
	MTF2	MFAP1	
	MTFR1	MFAP3L	
	MTFR1L	MFN2	
	MTHFD1	MGA	
	MTHFD1L	MGAT1	
	MTHFD2	MGAT4B	
	MTMR14	MGAT5	
	MTMR3	MGEA5	
	MTMR4	MICAL3	
	MTMR9	MID1	
	MTOR	MID1IP1	
	MTPN	MIEN1	
	MTSS1L	MIER2	
	MTX1	MIER3	
	MTX2	MINK1	
	MUM1	MINOS1	
	MUT	MITF	
	MVB12B	MKL1	
	MYADM	MKL2	
	MYBBP1A	MKLN1	
	MYBL2	MKRN1	
	MYC	MKX	
	MYCBP2	MLEC	
	MYCL	MLF2	
	MYCN	MLLT1	
	MYEF2	MLLT10	
	MYH10	MLLT11	
	MYH9	MLLT3	
	MYL12B	MLLT4	
	MYO10	MLLT6	
	MYO5A	MLXIP	
	MYO9B	MMGT1	
	MZT1	MMP14	
	N4BP1	MMP16	
	NAA11	MMP2	
	NAA15	MMP24	
	NAA20	MN1	
	NAA35	MNT	
	NAA38	MNX1	
	NAA40	MOB1A	
	NAA50	MOB4	
	NAA60	MOBP	
	NABP2	MON2	
	NACA2	MORC2	

© 2023 Morton SU et al. *JAMA Network Open*.

	NACC1	MORC3	
	NADK	MORC4	
	NADK2	MORF4L1	
	NAE1	MORF4L2	
	NAMPT	MORN4	
	NAP1L1	MOS	
	NAP1L4	MOSPD1	
	NAPA	MOSPD2	
	NARF	MOV10	
	NARS	MPC2	
	NASP	MPHOSPH10	
	NAT10	MPP1	
	NAV1	MPP6	
	NBR1	MPPED2	
	NCAPD2	MPRIP	
	NCAPD3	MRAS	
	NCAPG	MRC1	
	NCAPG2	MRC1L1	
	NCAPH	MRC2	
	NCAPH2	MRGBP	
	NCBP1	MRPL27	
	NCBP2	MRPL41	
	NCDN	MRTO4	
	NCKAP1	MSANTD1	
	NCL	MSANTD2	
	NCLN	MSH2	
	NCOA3	MSI1	
	NCOA5	MSI2	
	NCOA6	MSL1	
	NCOR1	MSL2	
	NCOR2	MSL3	
	NCS1	MSN	
	NCSTN	MSTN	
	NDC1	MTA1	
	NDC80	MTA2	
	NDE1	MTCH1	
	NDFIP1	MTF1	
	NDN	MTF2	
	NDOR1	MTM1	
	NDST1	MTMR1	
	NDUFA1	MTMR12	
	NDUFA10	MTMR3	
	NDUFA12	MTMR4	
	NDUFA2	MTOR	
	NDUFA4	MTPAP	
	NDUFA6	MTPN	
	NDUFA7	MTSS1	
	NDUFA8	MTSS1L	
	NDUFA9	MTUS2	
	NDUFB3	MUC5B	
	NDUFB5	MVB12B	

© 2023 Morton SU et al. *JAMA Network Open*.

	NDUFB6	MXD4	
	NDUFB7	MYB	
	NDUFB8	MYBL1	
	NDUFB9	MYC	
	NDUFS1	MYCBP	
	NDUFS2	MYCBP2	
	NDUFS6	MYCN	
	NDUFS7	MYEF2	
	NDUFS8	MYH10	
	NDUFV1	MYH11	
	NDUFV2	MYH9	
	NECAB2	MYLPF	
	NEDD1	MYNN	
	NEDD4	MYO16	
	NEDD8	MYO18A	
	NEFL	MYO5A	
	NEFM	MYO9B	
	NEK2	MYOG	
	NEK6	MYPOP	
	NEK9	MYRF	
	NELFA	MYT1	
	NELFB	MYT1L	
	NELFCD	N4BP1	
	NELFE	N4BP2L1	
	NEO1	NAA10	
	NES	NAA15	
	NET1	NAA25	
	NEURL4	NAA30	
	NF2	NAA35	
	NFATC3	NAA50	
	NFE2L1	NAA60	
	NFKB1	NAA60	
	NFX1	NAB2	
	NFYA	NABP2	
	NGFRAP1	NACC1	
	NHP2	NAF1	
	NHP2L1	NAMPT	
	NID1	NANOG	
	NID2	NANOS1	
	NIF3L1	NAP1L1	
	NIFK	NAP1L2	
	NIPSNAP1	NAP1L4	
	NIPSNAP3A	NAP1L5	
	NISCH	NASP	
	NKAIN1	NAT8L	
	NKIRAS2	NAV1	
	NKTR	NAV2	
	NLGN2	NAV3	
	NLK	NBEA	
	NMD3	NCALD	
	NME1	NCAM1	

© 2023 Morton SU et al. *JAMA Network Open*.

	NME1-NME2	NCAPG2	
	NME4	NCBP1	
	NMRAL1	NCDN	
	NMT1	NCKAP1	
	NNAT	NCKAP1L	
	NOB1	NCKAP5L	
	NOC4L	NCL	
	NOL10	NCLN	
	NOL11	NCOA1	
	NOL6	NCOA2	
	NOL7	NCOA3	
	NOL9	NCOA5	
	NOLC1	NCOA6	
	NOM1	NCOR1	
	NOMO1	NCOR2	
	NOMO2	NCS1	
	NOMO3	NCSTN	
	NONO	NDC1	
	NOP10	NDEL1	
	NOP14	NDFIP1	
	NOP16	NDFIP2	
	NOP2	NDN	
	NOP56	NDP	
	NOP58	NDST1	
	NOP9	NDUFA1	
	NOSIP	NDUFA2	
	NOTCH1	NDUFAB1	
	NOTCH2	NDUFAF4	
	NOTCH3	NDUFB11	
	NPC2	NDUFC2	
	NPDC1	NDUFC2-KCTD14	
	NPEPL1	NDUFS7	
	NPEPPS	NEDD4L	
	NPLOC4	NEDD8	
	NPM1	NEGR1	
	NPNT	NEK7	
	NR1H2	NELL2	
	NR2E1	NEURL1B	
	NR2F1	NEURL4	
	NR2F2	NEUROD1	
	NR2F6	NEUROD2	
	NR6A1	NEUROD6	
	NRARP	NEUROG2	
	NRAS	NF1	
	NRBP1	NF2	
	NRD1	NFASC	
	NREP	NFAT5	
	NRM	NFATC2	
	NSA2	NFATC3	
	NSD1	NFATC4	
	NSDHL	NFE2	

© 2023 Morton SU et al. *JAMA Network Open*.

	NSFL1C	NFE2L1	
	NSL1	NFIA	
	NSMCE4A	NFIB	
	NSUN2	NFIL3	
	NSUN5	NFIX	
	NT5C	NFKB1	
	NT5C3B	NFKB2	
	NT5DC2	NFKBIA	
	NTAN1	NFKBIE	
	NTN1	NFKBIZ	
	NUAK1	NGEF	
	NUAK2	NGF	
	NUBP1	NGFRAP1	
	NUBP2	NHLH2	
	NUCB1	NHP2L1	
	NUCKS1	NHS	
	NUDC	NIPA2	
	NUDCD2	NIPBL	
	NUDCD3	NISCH	
	NUDT16L1	NKAP	
	NUDT3	NKD1	
	NUDT4	NKRF	
	NUDT5	NKTR	
	NUDT9	NKX1-1	
	NUF2	NKX2-3	
	NUMA1	NKX2-5	
	NUMB	NKX3-2	
	NUP107	NLGN1	
	NUP133	NLGN2	
	NUP153	NLGN3	
	NUP155	NLGN4X	
	NUP160	NLK	
	NUP188	NMNAT2	
	NUP205	NMT1	
	NUP210	NNT	
	NUP214	NODAL	
	NUP43	NOG	
	NUP50	NOL4	
	NUP62	NOL6	
	NUP85	NONO	
	NUP88	NOP10	
	NUP93	NOP2	
	NUP98	NOP56	
	NUPL1	NOS1	
	NUS1	NOS1AP	
	NUSAP1	NOTCH1	
	NUTF2	NOTCH2	
	NVL	NOTCH2NL	
	NXF1	NOVA1	
	NXN	NOVA2	
	NYNRIN	NPAS1	

© 2023 Morton SU et al. *JAMA Network Open*.

	OARD1	NPAS2	
	OAT	NPAS3	
	OAZ1	NPAS4	
	OBSL1	NPEPPS	
	OCIAD1	NPLOC4	
	ODC1	NPM1	
	ODF2	NPTN	
	OGDH	NPTX1	
	OGFR	NPTXR	
	OGT	NR0B1	
	OLA1	NR1D1	
	OPA3	NR1H3	
	ORC2	NR2C1	
	ORC3	NR2C2	
	ORC5	NR2E1	
	ORC6	NR2F1	
	ORMDL3	NR2F2	
	OSBP	NR3C1	
	OSBPL11	NR3C2	
	OSTC	NR4A2	
	OTUB1	NR4A3	
	OTUD4	NR5A1	
	OTUD5	NR6A1	
	OTUD6B	NRBP1	
	OTX1	NRF1	
	OTX2	NRG1	
	OVCA2	NRG2	
	OXA1L	NRIP1	
	OXCT1	NRK	
	OXSR1	NRN1	
	P4HA1	NRP1	
	P4HB	NRXN1	
	PA2G4	NRXN2	
	PABPN1	NRXN3	
	PACS1	NSD1	
	PACS2	NSDHL	
	PACSIN2	NSG2	
	PAF1	NTF3	
	PAFAH1B1	NTN1	
	PAFAH1B2	NTN4	
	PAFAH1B3	NTNG1	
	PAGR1	NTNG2	
	PAICS	NTRK2	
	PAIP1	NTRK3	
	PAIP2	NUCKS1	
	PAIP2B	NUDC	
	PAK1	NUDT21	
	PAK2	NUDT4	
	PAK4	NUFIP2	
	PALD1	NUMA1	
	PALM	NUMBL	

© 2023 Morton SU et al. *JAMA Network Open*.

	PALM2-AKAP2	NUP153	
	PAN2	NUP155	
	PANK2	NUP188	
	PANK4	NUP205	
	PANX1	NUP62	
	PAPD4	NUP85	
	PAPD5	NUP98	
	PAPD7	NUPL1	
	PAPOLA	NUS1	
	PAPSS1	NUTF2	
	PARD6G	NXF1	
	PARK7	NXF2B	
	PARL	NXN	
	PARM1	NXNL2	
	PARP1	NXPH2	
	PARP2	NXT1	
	PARVA	NXT2	
	PATL1	NYAP1	
	PATZ1	OCRL	
	PAX6	ODF1	
	PAXBP1	OFD1	
	PAXIP1	OGDH	
	PBK	OGT	
	PBRM1	OLFM1	
	PBX1	OLIG1	
	PBX2	OLIG3	
	PCBP1	OMG	
	PCBP2	ONECUT1	
	PCBP4	ONECUT2	
	PCDH18	OPA1	
	PCDHAC2	OPA3	
	PCDHGC5	OPHN1	
	PCGF2	OPN1LW	
	PCGF3	OPN5	
	PCID2	OR10C1	
	PCIF1	OR12D2	
	PCM1	OR2H1	
	PCNP	OR6C1	
	PCNT	ORC2	
	PCNXL3	ORMDL3	
	PCYOX1	OSBP	
	PCYT1A	OSBPL11	
	PCYT2	OSBPL6	
	PDAP1	OSBPL9	
	PDCD11	OSGIN2	
	PDCD4	OSM	
	PDCD6IP	OSR1	
	PDCL	OTC	
	PDE7A	OTP	
	PDF	OTUB1	
	PDGFA	OTUD1	

© 2023 Morton SU et al. *JAMA Network Open*.

	PDGFRB	OTUD4	
	PDHA1	OTUD5	
	PDIA3	OTUD7A	
	PDIA4	OTX1	
	PDIA6	OTX2	
	PDK1	OVOL1	
	PDK3	OVOL2	
	PDPR	OXR1	
	PDS5A	OXSR1	
	PDS5B	OXT	
	PDXDC1	P2RY1	
	PDXP	P4HA1	
	PDZD11	P4HB	
	PDZRN3	PA2G4	
	PEA15	PABPC1	
	PEF1	PABPC4	
	PEG10	PABPC5	
	PEG3	PACS1	
	PELP1	PACS2	
	PES1	PACSIN1	
	PEX19	PAFAH1B1	
	PEX5	PAFAH1B2	
	PEX6	PAGE1	
	PFAS	PAGE4	
	PFDN2	PAGR1	
	PFDN5	PAGR1	
	PFDN6	PAIP1	
	PFKFB3	PAIP2	
	PFKL	PAK2	
	PFKM	PAK3	
	PFN1	PAK7	
	PFN2	PALM	
	PGAM4	PAN2	
	PGAM5	PAN3	
	PGD	PANK4	
	PGK1	PAPD5	
	PGLS	PAPD7	
	PGRMC1	PAPOLA	
	PGRMC2	PAPOLG	
	PGS1	PARD3	
	PHACTR4	PARD6B	
	PHAX	PARK7	
	PHB	PARP6	
	PHB2	PASD1	
	PHC1	PATL1	
	PHC2	PATZ1	
	PHF10	PAX1	
	PHF12	PAX2	
	PHF2	PAX5	
	PHF21B	PAX6	
	PHF23	PAX8	

© 2023 Morton SU et al. *JAMA Network Open*.

	PHF5A	PAXBP1	
	PHF6	PAXIP1	
	PHGDH	PBDC1	
	PHIP	PBRM1	
	PHLDA1	PBX1	
	PHLDB1	PBX2	
	PHLDB2	PCBP1	
	PHPT1	PCBP2	
	PHRF1	PCBP3	
	PHTF1	PCDH1	
	PHYKPL	PCDH10	
	PI4K2A	PCDH17	
	PI4KA	PCDH19	
	PIAS2	PCDH20	
	PIAS4	PCDH9	
	PICALM	PCDHAC2	
	PIEZO1	PCDHGC4	
	PIGF	PCF11	
	PIGQ	PCGF1	
	PIGS	PCGF2	
	PIGT	PCGF3	
	PIGX	PCGF5	
	PIK3R2	PCIF1	
	PIK3R3	PCLO	
	PIM1	PCMT1	
	PIN1	PCNA	
	PINK1	PCNP	
	PIP4K2B	PCNX	
	PIP5K1A	PCNXL3	
	PIP5K1C	PCSK1N	
	PITHD1	PCSK2	
	PITPNA	PCYT1B	
	PITPNB	PDCD10	
	PITRM1	PDCD6IP	
	PJA1	PDE10A	
	PJA2	PDE2A	
	PKDCC	PDE4A	
	PKIA	PDE4D	
	PKM	PDE7B	
	PKN2	PDE8B	
	PKNOX2	PDGFA	
	PKP4	PDGFB	
	PLAA	PDGFC	
	PLAGL1	PDGFRA	
	PLAGL2	PDGFRB	
	PLBD2	PDHA1	
	PLCB3	PDIA3	
	PLCG1	PDLIM4	
	PLEC	PDLIM7	
	PLEKHA5	PDPK1	
	PLEKHA8	PDS5A	

© 2023 Morton SU et al. *JAMA Network Open*.

	PLEKHG2	PDS5B	
	PLEKHJ1	PDXK	
	PLEKHO1	PDZD2	
	PLIN3	PDZD4	
	PLK1	PDZD8	
	PLK4	PDZRN3	
	PLOD2	PEA15	
	PLOD3	PEG10	
	PLRG1	PELI2	
	PLS3	PELP1	
	PLXNA1	PER2	
	PLXNA3	PES1	
	PLXNB1	PEX26	
	PLXNB2	PFDN1	
	PLXND1	PFDN6	
	PMF1	PFN1	
	PMM2	PFN3	
	PMPCA	PGAP2	
	PMPCB	PGD	
	PNISR	PGK1	
	PNKD	PGRMC1	
	PNN	PGRMC2	
	PNO1	PHACTR1	
	PNP	PHACTR3	
	PNPT1	PHB	
	PNRC1	PHC1	
	PNRC2	PHC2	
	POC1B	PHC3	
	PODXL	PHEX	
	POFUT2	PHF1	
	POGK	PHF12	
	POGLUT1	PHF13	
	POGZ	PHF19	
	POLA2	PHF2	
	POLD1	PHF20	
	POLD2	PHF20L1	
	POLD3	PHF21A	
	POLDIP2	PHF21B	
	POLDIP3	PHF23	
	POLE	PHF3	
	POLE3	PHF5A	
	POLG	PHF6	
	POLR1A	PHF8	
	POLR1B	PHIP	
	POLR1C	PHLDA1	
	POLR2A	PHLPP1	
	POLR2D	PHOSPHO1	
	POLR2E	PHOX2B	
	POLR2F	PHYHIP	
	POLR2G	PHYHIPL	
	POLR2I	PI4K2A	

© 2023 Morton SU et al. *JAMA Network Open*.

	POLR2J	PI4KB	
	POLR2M	PIAS1	
	POLR3C	PIAS2	
	POLR3D	PIAS3	
	POLR3E	PIAS4	
	POLR3K	PICALM	
	POLRMT	PIGA	
	POM121	PIGR	
	POM121C	PIH1D3	
	POMGNT1	PIK3AP1	
	POP4	PIK3C2B	
	POR	PIK3CA	
	POU3F4	PIK3CB	
	PPA1	PIK3CD	
	PPAN	PIK3R1	
	PPAN-P2RY11	PIM1	
	PPAP2B	PIM2	
	PPAT	PIM3	
	PPDPF	PIN1	
	PPFIA1	PIN4	
	PPHLN1	PINLYP	
	PPIA	PIP4K2B	
	PPIB	PIP5K1C	
	PPIC	PIRT	
	PPID	PITPNA	
	PPIE	PITPNB	
	PPIF	PITPNC1	
	PPIG	PITPNM2	
	PPIL1	PITPNM3	
	PPIL2	PITX1	
	PPIP5K2	PITX2	
	PPM1A	PITX3	
	PPM1F	PJA1	
	PPM1G	PKD1	
	PPM1L	PKIG	
	PPME1	PKM	
	PPP1CA	PKN2	
	PPP1CB	PKNOX1	
	PPP1CC	PKNOX2	
	PPP1R10	PLAG1	
	PPP1R12A	PLAGL1	
	PPP1R12C	PLAGL2	
	PPP1R14B	PLCB1	
	PPP1R15B	PLCB3	
	PPP1R16A	PLCG2	
	PPP1R2	PLCL2	
	PPP1R7	PLEKHA2	
	PPP1R8	PLEKHA5	
	PPP1R9B	PLEKHF2	
	PPP2CA	PLK1	
	PPP2CB	PLK2	

© 2023 Morton SU et al. *JAMA Network Open*.

	PPP2R1A	PLP1	
	PPP2R1B	PLRG1	
	PPP2R2A	PLS3	
	PPP2R2D	PLXNA1	
	PPP2R4	PLXNA4	
	PPP2R5A	PLXNB2	
	PPP2R5C	PLXNC1	
	PPP2R5D	PLXND1	
	PPP2R5E	PMEPA1	
	PPP3CA	PMP22	
	PPP3CB	PNISR	
	PPP4C	PNRC1	
	PPP4R1	PODXL2	
	PPP4R2	POFUT1	
	PPP5C	POGK	
	PPP6C	POGZ	
	PPP6R1	POLA1	
	PPP6R3	POLD3	
	PPRC1	POLR1A	
	PPT1	POLR1B	
	PPT2	POLR2A	
	PPWD1	POLR2B	
	PQBP1	POLR2H	
	PRC1	POMP	
	PRCC	PORCN	
	PRDM4	POT1	
	PRDX1	POU2AF1	
	PRDX2	POU2F1	
	PRDX3	POU2F2	
	PRDX4	POU3F1	
	PRDX5	POU3F2	
	PRDX6	POU3F3	
	PREB	POU3F4	
	PRELID1	POU4F1	
	PREP	POU4F3	
	PRICKLE4	POU5F1	
	PRIM1	POU6F1	
	PRKAB1	PPAP2B	
	PRKACA	PPARD	
	PRKACB	PPARGC1A	
	PRKAG1	PPARGC1B	
	PRKAR1A	PPAT	
	PRKAR2A	PPEF1	
	PRKAR2B	PPFIA1	
	PRKCI	PPFIA2	
	PRKCSH	PPFIA3	
	PRKD3	PPIA	
	PRKRA	PPIE	
	PRKRIR	PPIG	
	PRMT1	PPIL3	
	PRMT5	PPM1A	

© 2023 Morton SU et al. *JAMA Network Open*.

	PRMT7	PPM1E	
	PROM1	PPM1G	
	PROSER1	PPM1H	
	PRPF18	PPME1	
	PRPF19	PPP1CA	
	PRPF3	PPP1CB	
	PRPF31	PPP1CC	
	PRPF38B	PPP1R10	
	PRPF39	PPP1R12A	
	PRPF4	PPP1R13B	
	PRPF40A	PPP1R16B	
	PRPF4B	PPP1R37	
	PRPF6	PPP1R7	
	PRPF8	PPP1R8	
	PRPS1	PPP1R9B	
	PRPS2	PPP2CA	
	PRPSAP1	PPP2R1A	
	PRR11	PPP2R2A	
	PRR12	PPP2R2B	
	PRR14	PPP2R2C	
	PRRC2A	PPP2R2D	
	PRRC2B	PPP2R3A	
	PRRC2C	PPP2R4	
	PRRX2	PPP2R5A	
	PRTG	PPP2R5B	
	PRUNE	PPP2R5C	
	PSAP	PPP2R5D	
	PSAT1	PPP2R5E	
	PSEN1	PPP3CA	
	PSIP1	PPP3CB	
	PSKH1	PPP4R1	
	PSMA1	PPP4R2	
	PSMA3	PPP5C	
	PSMA4	PPP6C	
	PSMA6	PPP6R1	
	PSMA7	PPP6R3	
	PSMB1	PPRC1	
	PSMB2	PPTC7	
	PSMB3	PQBP1	
	PSMB4	PRAF2	
	PSMB5	PRCC	
	PSMB6	PRDM1	
	PSMB7	PRDM11	
	PSMC1	PRDM12	
	PSMC2	PRDM13	
	PSMC3	PRDM16	
	PSMC4	PRDM2	
	PSMC5	PRDM6	
	PSMC6	PRDM8	
	PSMD1	PREX1	
	PSMD11	PRICKLE1	

© 2023 Morton SU et al. *JAMA Network Open*.

	PSMD12	PRICKLE2	
	PSMD13	PRICKLE3	
	PSMD14	PRIMA1	
	PSMD2	PRKAB2	
	PSMD3	PRKACA	
	PSMD4	PRKAG2	
	PSMD5	PRKAR1A	
	PSMD6	PRKAR2B	
	PSMD7	PRKCB	
	PSMD8	PRKCD	
	PSME1	PRKCE	
	PSME3	PRKCG	
	PSME4	PRKCZ	
	PSMG1	PRKD2	
	PSPC1	PRKDC	
	PSRC1	PRKG1	
	PTBP1	PRKG2	
	PTBP2	PRKRIR	
	PTBP3	PRLR	
	PTCD3	PRMT1	
	PTCH1	PRMT5	
	PTDSS2	PRMT8	
	PTGES3	PROSER1	
	PTGFRN	PROX1	
	PTK2	PRPF19	
	PTK7	PRPF3	
	PTMA	PRPF31	
	PTMS	PRPF38A	
	PTN	PRPF38B	
	PTOV1	PRPF39	
	PTP4A1	PRPF4	
	PTP4A2	PRPF40B	
	PTPLAD1	PRPF4B	
	PTPN1	PRPF8	
	PTPN11	PRPS1	
	PTPN12	PRPS2	
	PTPN13	PRR12	
	PTPN23	PRR14L	
	PTPN9	PRR15	
	PTPRA	PRR15L	
	PTPRF	PRR19	
	PTPRS	PRR7	
	PTRHD1	PRRC2A	
	PTTG1IP	PRRC2B	
	PUF60	PRRC2C	
	PUM1	PRRT2	
	PUM2	PSAP	
	PVRL1	PSD	
	PVRL2	PSEN1	
	PVRL3	PSENEN	
	PWP1	PSIP1	

© 2023 Morton SU et al. *JAMA Network Open*.

	PWP2	PSMA1	
	PXDN	PSMA2	
	PYCR2	PSMA3	
	PYCRL	PSMA4	
	PYGB	PSMA5	
	PYGO2	PSMA6	
	QARS	PSMA7	
	QDPR	PSMB1	
	QKI	PSMB2	
	QRICH1	PSMB3	
	QSER1	PSMB5	
	QTRT1	PSMB7	
	R3HCC1	PSMC1	
	R3HDM1	PSMC2	
	R3HDM2	PSMC3	
	R3HDM4	PSMC5	
	RAB10	PSMC6	
	RAB11A	PSMD1	
	RAB11B	PSMD10	
	RAB11FIP3	PSMD11	
	RAB11FIP4	PSMD12	
	RAB14	PSMD13	
	RAB18	PSMD14	
	RAB1A	PSMD2	
	RAB1B	PSMD3	
	RAB21	PSMD4	
	RAB24	PSMD6	
	RAB28	PSME3	
	RAB2A	PSME4	
	RAB2B	PSPC1	
	RAB31	PTAR1	
	RAB34	PTBP1	
	RAB35	PTBP2	
	RAB4B	PTBP3	
	RAB4B-EGLN2	PTCH1	
	RAB5A	PTCHD1	
	RAB5B	PTGER4	
	RAB5C	PTGES	
	RAB6A	PTGES3	
	RAB6C	PTGS2	
	RAB7A	PTH1R	
	RAB8A	PTHLH	
	RABGGTB	PTK2	
	RABL6	PTK2B	
	RAC1	PTK7	
	RACGAP1	PTOV1	
	RAD18	PTP4A2	
	RAD21	PTPN1	
	RAD23A	PTPN11	
	RAD23B	PTPN12	
	RAD54L2	PTPN2	

© 2023 Morton SU et al. *JAMA Network Open*.

	RAD9A	PTPN4	
	RAE1	PTPN6	
	RAF1	PTPRA	
	RAI1	PTPRB	
	RAI14	PTPRC	
	RALA	PTPRCAP	
	RALBP1	PTPRD	
	RALGDS	PTPRF	
	RALY	PTPRK	
	RAMP2	PTPRM	
	RAN	PTPRS	
	RANBP1	PTPRT	
	RANBP10	PTPRZ1	
	RANBP2	PUF60	
	RANBP3	PUM1	
	RANBP9	PUM2	
	RANGAP1	PURA	
	RAP1A	PURB	
	RAP1B	PURG	
	RAP1GDS1	PVRL2	
	RAP2C	PVRL3	
	RAPGEF1	PVRL4	
	RARG	PWWP2A	
	RARS	PWWP2B	
	RASA1	PYGO1	
	RAVER1	PYGO2	
	RAVER2	QKI	
	RBBP4	QRICH1	
	RBBP5	QSER1	
	RBBP6	R3HDM1	
	RBBP7	R3HDM2	
	RBBP9	RAB10	
	RBCK1	RAB11A	
	RBFOX2	RAB11FIP2	
	RBM12	RAB11FIP3	
	RBM14	RAB11FIP4	
	RBM15B	RAB14	
	RBM17	RAB18	
	RBM18	RAB1A	
	RBM19	RAB1B	
	RBM22	RAB2A	
	RBM25	RAB30	
	RBM26	RAB33A	
	RBM27	RAB33B	
	RBM28	RAB35	
	RBM3	RAB39B	
	RBM33	RAB3A	
	RBM38	RAB3GAP2	
	RBM39	RAB43	
	RBM45	RAB4B	
	RBM4B	RAB5A	

© 2023 Morton SU et al. *JAMA Network Open*.

	RBM5	RAB5C	
	RBM8A	RAB6A	
	RBMS1	RAB6B	
	RBMS2	RAB7A	
	RBMX	RAB8A	
	RBPJ	RAB9A	
	RBPMS	RAB9B	
	RBX1	RABEP1	
	RCBTB1	RABGAP1	
	RCBTB2	RABL6	
	RCC1	RAC1	
	RCC2	RAC2	
	RCE1	RAD21	
	RCL1	RAD23B	
	RCN1	RAD54L2	
	RCN2	RAE1	
	RCN3	RAF1	
	RCOR1	RAI1	
	RCOR2	RAI14	
	RDX	RAI2	
	RECQL4	RALA	
	REEP3	RALGAPA1	
	REEP4	RALGAPB	
	REPIN1	RALGDS	
	REPS1	RALGPS1	
	RER1	RAN	
	RERE	RANBP1	
	REV3L	RANBP10	
	REXO1	RANBP2	
	RFC1	RANBP3	
	RFC2	RANBP9	
	RFC3	RAP1A	
	RFC4	RAP1B	
	RFK	RAP1GAP	
	RFWD2	RAP1GAP2	
	RFX4	RAP1GDS1	
	RGL2	RAP2A	
	RGMA	RAPGEF1	
	RGP1	RAPGEF2	
	RGS12	RAPGEF4	
	RGS19	RAPGEF5	
	RHEB	RAPGEF6	
	RHNO1	RAPH1	
	RHOA	RARA	
	RHOB	RARB	
	RHOBTB3	RARG	
	RHOG	RASA1	
	RHOQ	RASAL2	
	RHOT1	RASD2	
	RHOT2	RASGEF1A	
	RIOK3	RASGEF1B	

© 2023 Morton SU et al. *JAMA Network Open*.

	RLF	RASGRF1	
	RMDN3	RASGRP1	
	RMND5A	RASIP1	
	RNASEH2C	RASSF8	
	RNASEK-C17orf49	RAVER1	
	RND2	RB1	
	RNF10	RB1CC1	
	RNF11	RBBP4	
	RNF114	RBBP5	
	RNF115	RBBP6	
	RNF121	RBBP7	
	RNF123	RBFOX1	
	RNF126	RBFOX2	
	RNF14	RBFOX3	
	RNF144A	RBL2	
	RNF145	RBM10	
	RNF149	RBM14	
	RNF165	RBM14-RBM4	
	RNF167	RBM15	
	RNF181	RBM15B	
	RNF185	RBM17	
	RNF187	RBM20	
	RNF2	RBM22	
	RNF20	RBM25	
	RNF214	RBM26	
	RNF219	RBM27	
	RNF220	RBM3	
	RNF25	RBM33	
	RNF38	RBM38	
	RNF4	RBM39	
	RNF40	RBM4	
	RNF44	RBM41	
	RNF5	RBM42	
	RNF7	RBM47	
	RNF8	RBM5	
	RNH1	RBM6	
	RNPEP	RBM7	
	RNPEPL1	RBM8A	
	RNPS1	RBMX	
	ROBO1	RBMX2	
	ROR2	RBP4	
	RP11-1055B8.7	RBP7	
	RP11-1407O15.2	RBPJ	
	RP11-156P1.2	RBPMS	
	RP11-407N17.3	RBPMS2	
	RP11-468E2.2	RBX1	
	RP11-512M8.5	RC3H1	
	RP4-559A3.7	RC3H2	
	RPA1	RCAN2	
	RPA2	RCC1	
	RPAP1	RCC2	

© 2023 Morton SU et al. *JAMA Network Open*.

	RPF2	RCOR1	
	RPL10A	RCOR3	
	RPL12	RDH10	
	RPL13	RDX	
	RPL13A	REEP1	
	RPL14	REL	
	RPL15	RELA	
	RPL18A	RELB	
	RPL19	RELL1	
	RPL21	RELN	
	RPL22	REPS1	
	RPL22L1	RERE	
	RPL23	REST	
	RPL23A	RET	
	RPL26	REV1	
	RPL27	REV3L	
	RPL27A	REXO1	
	RPL3	RFC1	
	RPL30	RFWD2	
	RPL31	RFX1	
	RPL32	RFX2	
	RPL34	RFX3	
	RPL35	RFX4	
	RPL35A	RFX7	
	RPL36	RGAG1	
	RPL36AL	RGL1	
	RPL37	RGS1	
	RPL37A	RGS10	
	RPL38	RGS19	
	RPL39	RGS7	
	RPL4	RGS7BP	
	RPL5	RHBDD2	
	RPL6	RHEB	
	RPL7	RHOA	
	RPL7A	RHOC	
	RPL7L1	RHOQ	
	RPL8	RHOT1	
	RPLP0	RHOV	
	RPLP1	RHOXF1	
	RPN1	RIBC1	
	RPN2	RIC8B	
	RPRD1A	RICTOR	
	RPRD1B	RIF1	
	RPS10	RIMBP2	
	RPS11	RIMKLB	
	RPS12	RIMS1	
	RPS13	RIMS2	
	RPS14	RING1	
	RPS15	RLF	
	RPS18	RLIM	
	RPS19	RMND5A	

© 2023 Morton SU et al. *JAMA Network Open*.

	RPS2	RNASEK	
	RPS20	RND1	
	RPS21	RND3	
	RPS23	RNF103	
	RPS24	RNF11	
	RPS25	RNF111	
	RPS26	RNF113A	
	RPS27A	RNF113B	
	RPS27L	RNF126	
	RPS28	RNF138	
	RPS29	RNF139	
	RPS3	RNF144A	
	RPS3A	RNF145	
	RPS4X	RNF146	
	RPS5	RNF165	
	RPS6	RNF17	
	RPS6KA1	RNF170	
	RPS6KA2	RNF19B	
	RPS6KA3	RNF2	
	RPS6KA6	RNF208	
	RPS6KB2	RNF214	
	RPS7	RNF220	
	RPS8	RNF26	
	RPS9	RNF31	
	RPSA	RNF34	
	RPTOR	RNF38	
	RQCD1	RNF4	
	RRAGA	RNF40	
	RRAGB	RNF41	
	RRAGC	RNF43	
	RRAGD	RNF8	
	RRBP1	RNPS1	
	RRM1	ROBO2	
	RRM2	ROCK1	
	RRN3	ROCK2	
	RRP1	ROR1	
	RRP12	RORB	
	RRP15	RORC	
	RRP1B	RP11-101E3.5	
	RRP7A	RP11-1055B8.7	
	RRP9	RP11-105C20.2	
	RRS1	RP11-152F13.10	
	RSL1D1	RP11-159G9.5	
	RSPO1	RP11-295D22.1	
	RTCA	RP11-343C2.11	
	RTCB	RP11-508N12.4	
	RTEL1	RP11-565P22.6	
	RTEL1-TNFRSF6B	RP11-58C22.1	
	RTF1	RP11-598P20.5	
	RTFDC1	RP11-599B13.6	
	RTKN	RP11-603J24.9	

	RTN3	RP11-762I7.5	
	RTN4	RP11-793H13.10	
	RUFY2	RP2	
	RUVBL2	RPA2	
	RWDD1	RPGR	
	RXRA	RPL10	
	RXRB	RPL10A	
	RYK	RPL11	
	S1PR3	RPL13	
	SAC3D1	RPL13A	
	SAE1	RPL14	
	SAFB	RPL15	
	SAFB2	RPL17	
	SALL1	RPL17-C18orf32	
	SALL2	RPL18	
	SALL4	RPL18A	
	SAMD1	RPL19	
	SAMD14	RPL21	
	SAMD4B	RPL23A	
	SAMHD1	RPL24	
	SAMM50	RPL26	
	SAP130	RPL27	
	SAP30	RPL27A	
	SAP30BP	RPL28	
	SAR1A	RPL3	
	SAR1B	RPL30	
	SARNP	RPL31	
	SARS	RPL32	
	SARS2	RPL34	
	SART1	RPL35	
	SART3	RPL35A	
	SASH1	RPL36	
	SAV1	RPL38	
	SBF1	RPL39	
	SBF2	RPL4	
	SBK1	RPL5	
	SBNO1	RPL6	
	SCAF1	RPL7	
	SCAF11	RPL7A	
	SCAF4	RPL8	
	SCAF8	RPL9	
	SCAMP2	RPLP0	
	SCAMP3	RPLP2	
	SCAMP4	RPP25L	
	SCAP	RPRD2	
	SCCPDH	RPS10	
	SCD	RPS10-NUDT3	
	SCPEP1	RPS11	
	SCRIB	RPS12	
	SCUBE1	RPS13	
	SCUBE3	RPS14	

© 2023 Morton SU et al. *JAMA Network Open*.

	SCYL1	RPS15	
	SDAD1	RPS15A	
	SDC1	RPS16	
	SDC3	RPS18	
	SDCBP	RPS19	
	SDCCAG3	RPS2	
	SDE2	RPS23	
	SDF4	RPS24	
	SDHA	RPS25	
	SDHB	RPS26	
	SDHC	RPS27	
	SDHD	RPS27A	
	SDR39U1	RPS28	
	SEC13	RPS3	
	SEC14L1	RPS3A	
	SEC16A	RPS4X	
	SEC23A	RPS4Y1	
	SEC23B	RPS5	
	SEC23IP	RPS6	
	SEC24B	RPS6KA3	
	SEC24C	RPS6KA4	
	SEC31A	RPS6KA5	
	SEC61A1	RPS6KA6	
	SEC61A2	RPS6KB1	
	SEC61G	RPS7	
	SEC62	RPS8	
	SEC63	RPS9	
	SEH1L	RPSA	
	SEL1L	RPTOR	
	SELO	RQCD1	
	SELT	RRAGB	
	SEMA3F	RRAGC	
	SEMA4C	RRAGD	
	SEMA5B	RRAS2	
	SENP1	RREB1	
	SENP2	RRM1	
	SENP3	RRM2	
	SENP6	RS1	
	15-Sep	RSBN1L	
	SEPHS1	RSF1	
	SEPHS2	RSL24D1	
	SEPP1	RSPRY1	
	11-Sep	RSRC2	
	2-Sep	RTF1	
	3-Sep	RTN1	
	7-Sep	RTN4R	
	8-Sep	RTN4RL1	
	9-Sep	RTN4RL2	
	SEPW1	RTP4	
	SERBP1	RUNDC3A	
	SERF2	RUNX1	

© 2023 Morton SU et al. *JAMA Network Open*.

	SERINC1	RUNX1T1	
	SERINC3	RUNX2	
	SERINC4	RUNX3	
	SERP1	RUSC1	
	SERPINF1	RUSC2	
	SERPINH1	RUVBL1	
	SET	RUVBL2	
	SETD1A	RWDD1	
	SETD1B	RXRA	
	SETD2	RXRB	
	SETD5	RYK	
	SETD7	RYR2	
	SETD8	RYR3	
	SETDB1	S100PBP	
	SF1	S1PR1	
	SF3A1	SACM1L	
	SF3A2	SAE1	
	SF3A3	SAFB	
	SF3B1	SAFB2	
	SF3B14	SALL1	
	SF3B2	SALL4	
	SF3B3	SAMD4A	
	SF3B5	SAMD4B	
	SFPQ	SAP130	
	SFR1	SARNP	
	SFRP1	SARS	
	SFRP2	SART1	
	SFSWAP	SART3	
	SFXN1	SAT1	
	SGOL1	SATB1	
	SGOL2	SATB2	
	SGPL1	SBF1	
	SGTA	SBNO1	
	SH2B1	SCAF1	
	SH3BGRL	SCAF11	
	SH3BGRL3	SCAF4	
	SH3BP4	SCAF8	
	SH3GL1	SCAI	
	SH3GLB1	SCAMP1	
	SH3GLB2	SCAMP5	
	SH3PXD2A	SCARF2	
	SH3PXD2B	SCD	
	SHC1	SCFD1	
	SHCBP1	SCHIP1	
	SHFM1	SCML1	
	SHH	SCML2	
	SHISA2	SCN1A	
	SHKBP1	SCN2A	
	SHMT1	SCN3A	
	SHMT2	SCN5A	
	SHOC2	SCN8A	

© 2023 Morton SU et al. *JAMA Network Open*.

	SHROOM3	SCRIB	
	SIDT2	SCRT2	
	SIGMAR1	SCUBE1	
	SIN3A	SCUBE3	
	SIPA1	SEC11C	
	SIPA1L1	SEC14L1	
	SIRPA	SEC24B	
	SIRPB1	SEC24C	
	SIRPG	SEC61A1	
	SIRT1	SEC61G	
	SIRT2	SEC62	
	SIRT6	SEL1L	
	SIVA1	SEMA3A	
	SIX2	SEMA3F	
	SIX5	SEMA4C	
	SKA2	SEMA4D	
	SKA3	SEMA6A	
	SKI	SEMA6D	
	SKIDA1	SENP1	
	SKIV2L	SENP2	
	SKIV2L2	SENP3	
	SKP1	SENP5	
	SKP2	SENP6	
	SLAIN2	SEPHS1	
	SLBP	SERBP1	
	SLC12A7	SERINC3	
	SLC16A1	SERP2	
	SLC16A3	SERPINC1	
	SLC20A1	SERPINE2	
	SLC20A2	SERPING1	
	SLC22A17	SERTAD2	
	SLC24A5	SESN3	
	SLC25A1	SESTD1	
	SLC25A11	SET	
	SLC25A13	SETBP1	
	SLC25A17	SETD1A	
	SLC25A22	SETD1B	
	SLC25A23	SETD2	
	SLC25A24	SETD5	
	SLC25A27	SETD8	
	SLC25A3	SETDB1	
	SLC25A36	SETX	
	SLC25A37	SEZ6L	
	SLC25A39	SF1	
	SLC25A4	SF3A1	
	SLC25A46	SF3A2	
	SLC25A5	SF3A3	
	SLC25A51	SF3B1	
	SLC25A52	SF3B14	
	SLC27A4	SF3B2	
	SLC2A1	SF3B3	

© 2023 Morton SU et al. *JAMA Network Open*.

	SLC2A14	SF3B4	
	SLC2A3	SF3B5	
	SLC30A5	SFMBT1	
	SLC30A9	SFMBT2	
	SLC31A1	SFPQ	
	SLC35A4	SFRP1	
	SLC35B1	SFSWAP	
	SLC35B2	SGIP1	
	SLC35C2	SGMS1	
	SLC35E1	SGTA	
	SLC38A1	SH2B1	
	SLC38A10	SH3GLB1	
	SLC38A2	SH3KBP1	
	SLC38A4	SH3PXD2A	
	SLC39A10	SHANK1	
	SLC39A6	SHANK3	
	SLC3A2	SHB	
	SLC44A2	SHE	
	SLC4A1	SHFM1	
	SLC4A2	SHH	
	SLC6A6	SHISA6	
	SLC6A8	SHISA7	
	SLC7A5	SHISA9	
	SLC7A6	SHOC2	
	SLMO2	SHOX	
	SLTM	SHROOM4	
	SMAD2	SIAH1	
	SMAD3	SIAH2	
	SMAD4	SIK1	
	SMAD5	SIK2	
	SMAP1	SIK3	
	SMARCA1	SIM1	
	SMARCA4	SIN3A	
	SMARCAD1	SIN3B	
	SMARCB1	SIPA1L1	
	SMARCC1	SIPA1L2	
	SMARCC2	SIPA1L3	
	SMARCD1	SIRPA	
	SMARCE1	SIX1	
	SMC1A	SIX2	
	SMC2	SIX3	
	SMC3	SIX4	
	SMC4	SIX6	
	SMC5	SKI	
	SMCHD1	SKIDA1	
	SMDT1	SKIL	
	SMEK1	SKIV2L2	
	SMEK2	SKOR2	
	SMG5	SKP2	
	SMG6	SLAIN1	
	SMG7	SLAIN2	

© 2023 Morton SU et al. *JAMA Network Open*.

	SMG8	SLC10A3	
	SMIM15	SLC12A2	
	SMN1	SLC12A5	
	SMN2	SLC13A4	
	SMNDC1	SLC16A2	
	SMO	SLC17A7	
	SMPD4	SLC1A2	
	SMU1	SLC1A3	
	SMURF1	SLC1A6	
	SMURF2	SLC20A2	
	SMYD5	SLC22A23	
	SNAI1	SLC24A3	
	SNAP23	SLC25A11	
	SNAP47	SLC25A14	
	SNAPC2	SLC25A19	
	SNAPC3	SLC25A28	
	SND1	SLC25A5	
	SNN	SLC29A1	
	SNORA34	SLC2A1	
	SNRNP200	SLC2A13	
	SNRNP40	SLC30A1	
	SNRNP70	SLC30A10	
	SNRPA	SLC30A3	
	SNRPA1	SLC32A1	
	SNRPB	SLC35A1	
	SNRPB2	SLC35A2	
	SNRPD1	SLC35F1	
	SNRPD3	SLC38A1	
	SNRPE	SLC38A2	
	SNRPF	SLC38A4	
	SNW1	SLC38A5	
	SNX1	SLC39A10	
	SNX12	SLC39A8	
	SNX17	SLC39A9	
	SNX2	SLC40A1	
	SNX22	SLC41A1	
	SNX27	SLC41A2	
	SNX3	SLC44A1	
	SNX4	SLC48A1	
	SNX5	SLC4A1	
	SNX6	SLC4A2	
	SOD1	SLC4A4	
	SOGA1	SLC4A7	
	SOGA2	SLC4A8	
	SON	SLC5A3	
	SORT1	SLC5A3	
	SOX10	SLC6A1	
	SOX11	SLC6A14	
	SOX12	SLC6A17	
	SOX13	SLC6A3	
	SOX2	SLC6A6	

© 2023 Morton SU et al. *JAMA Network Open*.

	SOX3	SLC6A8	
	SOX4	SLC7A1	
	SOX9	SLC7A3	
	SP1	SLC8A1	
	SP2	SLC8A2	
	SP3	SLC9A1	
	SPAG5	SLC9A3	
	SPAG7	SLC9A6	
	SPAG9	SLC9A7	
	SPARC	SLIT1	
	SPAST	SLIT2	
	SPATA13	SLIT3	
	SPATS2	SLITRK1	
	SPC24	SLITRK3	
	SPC25	SLITRK4	
	SPCS2	SLITRK5	
	SPCS3	SLMAP	
	SPECC1L	SLTM	
	SPEN	SMAD1	
	SPG20	SMAD2	
	SPG21	SMAD3	
	SPG7	SMAD4	
	SPIN1	SMAD5	
	SPIRE1	SMAD7	
	SPNS1	SMAP1	
	SPON1	SMARCA1	
	SPOP	SMARCA2	
	SPPL3	SMARCA4	
	SPRED1	SMARCA5	
	SPRED2	SMARCAD1	
	SPRY1	SMARCB1	
	SPSB3	SMARCC1	
	SPSB4	SMARCC2	
	SPTAN1	SMARCD1	
	SPTBN1	SMARCE1	
	SPTLC1	SMC1A	
	SPTSSA	SMC2	
	SQLE	SMC3	
	SQSTM1	SMCHD1	
	SRC	SMEK1	
	SRCAP	SMEK2	
	SREBF1	SMG1	
	SREBF2	SMG6	
	SREK1	SMG7	
	SREK1IP1	SMIM11	
	SRF	SMIM12	
	SRGAP3	SMIM14	
	SRM	SMIM20	
	SRP14	SMNDC1	
	SRP19	SMPD3	
	SRP54	SMPX	

© 2023 Morton SU et al. *JAMA Network Open*.

	SRP68	SMS	
	SRP72	SMU1	
	SRP9	SMURF1	
	SRPK1	SMURF2	
	SRPRB	SNAI2	
	SRRD	SNAP23	
	SRRM1	SNAP25	
	SRRM2	SNAP91	
	SRRT	SNCA	
	SRSF1	SND1	
	SRSF11	SNED1	
	SRSF2	SNPH	
	SRSF3	SNRK	
	SRSF4	SNRNP200	
	SRSF5	SNRNP40	
	SRSF6	SNRNP70	
	SRSF7	SNRPA1	
	SRSF9	SNRPB	
	SS18	SNRPC	
	SSB	SNRPD1	
	SSBP3	SNRPD2	
	SSR1	SNRPD3	
	SSR2	SNRPE	
	SSR3	SNRPF	
	SSR4	SNRPG	
	SSRP1	SNW1	
	SSSCA1	SNX1	
	SSX2IP	SNX12	
	ST13	SNX18	
	STAG1	SNX2	
	STAG2	SNX27	
	STARD3	SNX30	
	STARD7	SNX9	
	STAU1	SOBP	
	STIL	SOCS1	
	STIP1	SOCS2	
	STK11	SOCS3	
	STK11IP	SOCS5	
	STK16	SOCS6	
	STK24	SOCS7	
	STK25	SOGA1	
	STK35	SOGA3	
	STK38	SON	
	STK4	SORCS1	
	STK40	SORT1	
	STOML2	SOS1	
	STOX2	SOS2	
	STRA6	SOST	
	STRAP	SOWAHA	
	STRIP1	SOWAHD	
	STRN3	SOX1	

© 2023 Morton SU et al. *JAMA Network Open*.

	STRN4	SOX10	
	STT3A	SOX11	
	STT3B	SOX12	
	STUB1	SOX13	
	STX12	SOX14	
	STX16	SOX17	
	STX5	SOX2	
	SUB1	SOX21	
	SUCLA2	SOX30	
	SUCLG2	SOX4	
	SUDS3	SOX5	
	SUFU	SOX6	
	SUGT1	SOX8	
	SULF1	SOX9	
	SULF2	SP1	
	SUMO1	SP2	
	SUMO2	SP3	
	SUMO3	SP4	
	SUN1	SP5	
	SUPT16H	SP7	
	SUPT20H	SP8	
	SUPT5H	SP9	
	SUPT6H	SPAG9	
	SUPV3L1	SPARC	
	SURF2	SPAST	
	SURF4	SPATA2	
	SUV39H1	SPATS2	
	SUV39H2	SPATS2L	
	SUV420H2	SPCS2	
	SUZ12	SPCS3	
	SWI5	SPDEF	
	SYDE1	SPDYA	
	SYMPK	SPECC1L	
	SYNCRIP	SPEG	
	SYNGAP1	SPEN	
	SYT11	SPHKAP	
	SYVN1	SPI1	
	SZRD1	SPIB	
	TAB2	SPIN1	
	TACC3	SPIN3	
	TADA1	SPIN4	
	TADA2A	SPOCK1	
	TADA3	SPOP	
	TAF15	SPPL3	
	TAF2	SPRED1	
	TAF5L	SPRTN	
	TAF6	SPRY2	
	TAF9	SPSB1	
	TAGLN2	SPTAN1	
	TAGLN3	SPTB	
	TALDO1	SPTBN1	

© 2023 Morton SU et al. *JAMA Network Open*.

	TAOK2	SPTBN4	
	TARBP2	SPTLC2	
	TARDBP	SPTSSA	
	TARS	SPTY2D1	
	TAX1BP1	SQLE	
	TBC1D1	SRC	
	TBC1D10B	SRCAP	
	TBC1D13	SRCIN1	
	TBC1D16	SREK1	
	TBC1D20	SRF	
	TBC1D9B	SRGAP1	
	TBCA	SRGAP2	
	TBCB	SRGAP3	
	TBCD	SRGN	
	TBL1Y	SRP14	
	TBL2	SRP54	
	TBL3	SRPK2	
	TBP	SRPR	
	TBRG1	SRRM1	
	TBRG4	SRRM2	
	TCEA1	SRRT	
	TCEAL8	SRSF1	
	TCEB1	SRSF11	
	TCEB2	SRSF3	
	TCEB3	SRSF4	
	TCERG1	SRSF5	
	TCF12	SRSF6	
	TCF20	SRSF7	
	TCF25	SRSF9	
	TCF3	SS18	
	TCF4	SS18L1	
	TCF7L1	SSBP2	
	TCOF1	SSBP3	
	TCP1	SSH2	
	TDP2	SSR3	
	TEAD1	SSR4	
	TEAD2	SSRP1	
	TECR	SSTR1	
	TEDDM1	SSU72	
	TENM3	SSX4	
	TENM4	ST13	
	TES	ST14	
	TESK1	ST18	
	TET3	ST3GAL1	
	TEX10	ST3GAL2	
	TEX261	ST5	
	TF	ST7	
	TFDP1	ST8SIA3	
	TFDP2	STAG1	
	TFDP3	STAG2	
	TFG	STAM	

	TFIP11	STAT1	
	TFRC	STAT2	
	TGFBR1	STAT3	
	TGIF1	STAT4	
	TGIF2-C20orf24	STAT5A	
	TGS1	STAT5B	
	THAP11	STAT6	
	THBS1	STAU1	
	THBS3	STAU2	
	THOC1	STIM1	
	THOC2	STIM2	
	THOC3	STIP1	
	THOC5	STK11	
	THOC6	STK38L	
	THOC7	STK39	
	THOP1	STK40	
	THRAP3	STMN2	
	THUMPD1	STRAP	
	THUMPD3	STRBP	
	THYN1	STRN	
	TIA1	STRN3	
	TIAL1	STRN4	
	TICRR	STS	
	TIMELESS	STT3B	
	TIMM10	STX12	
	TIMM17A	STX1A	
	TIMM21	STX1B	
	TIMM44	STX5	
	TIMM50	STXBP1	
	TIMM8B	STXBP5	
	TIMP2	STXBP5L	
	TINF2	SUB1	
	TIPIN	SUCO	
	TJAP1	SUFU	
	TJP1	SUGP1	
	TJP2	SUGP2	
	TK1	SULT4A1	
	TKT	SUMO1	
	TLE3	SUMO2	
	TLE4	SUPT16H	
	TLK1	SUPT5H	
	TLK2	SUPT6H	
	TLN1	SURF4	
	TM2D2	SUV39H1	
	TM9SF1	SUV39H2	
	TM9SF2	SUV420H1	
	TM9SF3	SUV420H2	
	TM9SF4	SUZ12	
	TMA7	SVEP1	
	TMBIM6	SVOP	
	TMED1	SWT1	

© 2023 Morton SU et al. *JAMA Network Open*.

	TMED10	SYAP1	
	TMED2	SYCE3	
	TMED4	SYCP1	
	TMED9	SYCP2	
	TMEFF1	SYDE1	
	TMEM109	SYK	
	TMEM11	SYMPK	
	TMEM123	SYN1	
	TMEM126A	SYNCRIP	
	TMEM127	SYNGAP1	
	TMEM128	SYNGR3	
	TMEM131	SYNJ2BP	
	TMEM132A	SYNPO	
	TMEM132C	SYNRG	
	TMEM138	SYP	
	TMEM147	SYT1	
	TMEM14B	SYT11	
	TMEM14C	SYT13	
	TMEM164	SYT14	
	TMEM165	SYT2	
	TMEM183A	SYT3	
	TMEM184B	SYT7	
	TMEM184C	SYVN1	
	TMEM189	SZRD1	
	TMEM2	TAB2	
	TMEM201	TAB3	
	TMEM214	TAC1	
	TMEM216	TADA2B	
	TMEM222	TAF1	
	TMEM223	TAF10	
	TMEM230	TAF12	
	TMEM234	TAF1L	
	TMEM243	TAF3	
	TMEM248	TAF4	
	TMEM256-PLSCR3	TAF5	
	TMEM258	TAF5L	
	TMEM259	TAF7	
	TMEM30A	TAF7L	
	TMEM33	TAGAP	
	TMEM39A	TAL1	
	TMEM39B	TANC2	
	TMEM43	TANK	
	TMEM47	TAOK1	
	TMEM50A	TAOK2	
	TMEM55A	TAOK3	
	TMEM55B	TARDBP	
	TMEM59	TASP1	
	TMEM63B	TAZ	
	TMEM65	TBC1D10B	
	TMEM66	TBC1D20	
	TMEM9	TBC1D25	

© 2023 Morton SU et al. *JAMA Network Open*.

	TMEM97	TBC1D3	
	TMEM98	TBC1D30	
	TMOD3	TBCEL	
	TMPO	TBL1X	
	TMSB10	TBL1XR1	
	TMSB4Y	TBR1	
	TMX1	TBX1	
	TNFAIP1	TBX15	
	TNFRSF19	TBX18	
	TNK2	TBX2	
	TNKS	TBX20	
	TNKS2	TBX21	
	TNPO1	TBX22	
	TNPO2	TBX3	
	TNPO3	TBX4	
	TNRC18	TBX5	
	TNRC6A	TCEA1	
	TNRC6C	TCEAL1	
	TNS3	TCEAL3	
	TOE1	TCEAL4	
	TOLLIP	TCEAL5	
	TOMM20	TCEAL8	
	TOMM22	TCEB1	
	TOMM34	TCEB3	
	TOMM40	TCERG1	
	TOMM5	TCF12	
	TOMM70A	TCF15	
	TONSL	TCF20	
	TOP1	TCF21	
	TOP2A	TCF4	
	TOP2B	TCF7L2	
	TOP3B	TCFL5	
	TOPBP1	TCHHL1	
	TOPORS	TCOF1	
	TOR1AIP1	TCP1	
	TOR1AIP2	TDRD1	
	TOR1B	TDRD5	
	TOX2	TEAD1	
	TOX3	TEAD3	
	TP53	TECPR2	
	TP53BP1	TEF	
	TP53BP2	TEFM	
	TP53I11	TEK	
	TPCN1	TENM1	
	TPD52L2	TENM2	
	TPI1	TENM3	
	TPM1	TENM4	
	TPM4	TERF1	
	TPP1	TERF2	
	TPP2	TERT	
	TPR	TESK1	

© 2023 Morton SU et al. *JAMA Network Open*.

	TPRG1L	TET1	
	TPT1	TET3	
	TPX2	TEX10	
	TRA2A	TEX11	
	TRA2B	TFAP2A	
	TRABD	TFAP2B	
	TRAF3	TFAP2C	
	TRAF4	TFAP2D	
	TRAF7	TFAP4	
	TRAM1	TFCP2L1	
	TRAP1	TFDP1	
	TRAPPC1	TFDP2	
	TRIL	TFE3	
	TRIM17	TFEB	
	TRIM24	TGFA	
	TRIM27	TGFB2	
	TRIM28	TGFB3	
	TRIM32	TGFBR1	
	TRIM35	TGIF2-C20orf24	
	TRIM37	THAP1	
	TRIM44	THAP11	
	TRIM59	THBS1	
	TRIM7	THBS2	
	TRIM8	THOC1	
	TRIO	THOC2	
	TRIOBP	THRAP3	
	TRIP12	THRB	
	TRIP13	THSD7A	
	TRIP6	TIAL1	
	TRMT1	TIAM1	
	TRMT112	TIMM8A	
	TRMT2A	TIMP2	
	TRMT6	TIMP3	
	TRNT1	TIPARP	
	TRO	TIPRL	
	TROAP	TJP1	
	TROVE2	TKTL1	
	TRPC4AP	TLE1	
	TRPM7	TLE3	
	TRRAP	TLE4	
	TRUB2	TLK1	
	TSC2	TLK2	
	TSC22D1	TLN1	
	TSEN15	TLN2	
	TSN	TLR7	
	TSNAX	TLX1	
	TSPAN13	TM4SF2	
	TSPAN14	TM9SF2	
	TSPAN18	TM9SF3	
	TSPAN3	TM9SF4	
	TSPAN31	TMCC1	

© 2023 Morton SU et al. *JAMA Network Open*.

	TSPAN6	TMED10	
	TSPAN9	TMED2	
	TSPYL1	TMEFF1	
	TSPYL2	TMEM100	
	TSR1	TMEM108	
	TSR2	TMEM11	
	TSSC4	TMEM115	
	TSTD2	TMEM119	
	TTC1	TMEM121	
	TTC3	TMEM123	
	TTC4	TMEM131	
	TTC9C	TMEM132B	
	TTF2	TMEM132C	
	TTI1	TMEM132D	
	TTK	TMEM135	
	TTLL12	TMEM164	
	TTLL4	TMEM167B	
	TTYH3	TMEM170B	
	TUBA1A	TMEM178A	
	TUBA1B	TMEM178B	
	TUBB	TMEM179	
	TUBB2A	TMEM183A	
	TUBB2B	TMEM185B	
	TUBB3	TMEM194A	
	TUBB4A	TMEM200A	
	TUBB4B	TMEM201	
	TUBB6	TMEM203	
	TUBG1	TMEM204	
	TUBGCP2	TMEM210	
	TUBGCP3	TMEM229A	
	TUBGCP4	TMEM248	
	TUBGCP6	TMEM259	
	TUFM	TMEM30C	
	TULP3	TMEM35	
	TUSC2	TMEM39A	
	TVP23A	TMEM47	
	TWF1	TMEM55B	
	TWIST1	TMEM57	
	TWISTNB	TMEM60	
	TWSG1	TMEM63B	
	TXLNA	TMEM72	
	TXN	TMEM89	
	TXN2	TMEM9B	
	TXNDC12	TMX2	
	TXNDC17	TNF	
	TXNDC5	TNFAIP3	
	TXNL1	TNFAIP8L1	
	TXNL4A	TNFRSF14	
	TXNRD1	TNFRSF1A	
	TYMP	TNFRSF1B	
	TYRO3	TNFRSF8	

© 2023 Morton SU et al. *JAMA Network Open*.

	TYSND1	TNFSF12	
	U2AF1L4	TNFSF12-TNFSF13	
	U2AF2	TNFSF13	
	U2SURP	TNFSF13B	
	UBA1	TNFSF8	
	UBA2	TNIK	
	UBA3	TNNC1	
	UBA5	TNPO1	
	UBA52	TNPO2	
	UBA7	TNR	
	UBAC1	TNRC18	
	UBAC2	TNRC6A	
	UBALD2	TNRC6B	
	UBAP1	TNRC6C	
	UBAP2	TNS3	
	UBAP2L	TOB1	
	UBC	TOB2	
	UBE2B	TOMM22	
	UBE2C	TOMM40	
	UBE2D1	TOMM40L	
	UBE2D2	TOMM70A	
	UBE2D3	TOP1	
	UBE2E1	TOP2A	
	UBE2E3	TOP2B	
	UBE2G1	TOPAZ1	
	UBE2G2	TOPBP1	
	UBE2H	TOPORS	
	UBE2I	TOR4A	
	UBE2K	TOX	
	UBE2L3	TOX3	
	UBE2M	TOX4	
	UBE2N	TP53	
	UBE2O	TP53BP1	
	UBE2Q1	TP53INP2	
	UBE2R2	TP63	
	UBE2S	TP73	
	UBE2V1	TPP2	
	UBE2Z	TPR	
	UBE3B	TPT1	
	UBE3C	TPX2	
	UBE4B	TRA2B	
	UBFD1	TRABD2B	
	UBIAD1	TRAF2	
	UBL7	TRAF3	
	UBN1	TRAF4	
	UBP1	TRAF6	
	UBQLN1	TRAM1	
	UBQLN2	TRAM2	
	UBQLN4	TRAPPC10	
	UBR2	TRAPPC8	
	UBR4	TRERF1	

© 2023 Morton SU et al. *JAMA Network Open*.

	UBR5	TREX1	
	UBR7	TRIB1	
	UBTD2	TRIB2	
	UBTF	TRIM11	
	UBXN1	TRIM13	
	UBXN4	TRIM2	
	UBXN6	TRIM24	
	UCHL1	TRIM27	
	UCHL3	TRIM28	
	UCHL5	TRIM3	
	UCK1	TRIM33	
	UCK2	TRIM39	
	UFC1	TRIM39-RPP21	
	UFD1L	TRIM41	
	UFSP2	TRIM46	
	UGCG	TRIM67	
	UGDH	TRIM71	
	UGGT1	TRIM8	
	UHRF1	TRIM9	
	UHRF2	TRIO	
	ULK1	TRIP12	
	UMPS	TRIP13	
	UNC119	TRMT1L	
	UNC119B	TROVE2	
	UNG	TRPC4AP	
	UNK	TRPC5	
	UPF1	TRPS1	
	UQCC2	TRRAP	
	UQCR10	TSC1	
	UQCRC1	TSC2	
	UQCRC2	TSC22D1	
	UQCRFS1	TSC22D2	
	UQCRH	TSC22D4	
	UQCRQ	TSHZ1	
	URI1	TSHZ2	
	UROD	TSHZ3	
	USB1	TSKS	
	USF1	TSKU	
	USF2	TSN	
	USMG5	TSPAN12	
	USP1	TSPAN14	
	USP10	TSPAN5	
	USP11	TSPAN7	
	USP14	TSPAN9	
	USP19	TSPYL2	
	USP20	TSPYL5	
	USP21	TSR2	
	USP22	TSSK6	
	USP25	TTBK1	
	USP28	TTBK2	
	USP3	TTC17	

© 2023 Morton SU et al. *JAMA Network Open*.

	USP33	TTC28	
	USP34	TTC7B	
	USP39	TTR	
	USP4	TTYH1	
	USP42	TTYH3	
	USP46	TUBA1A	
	USP47	TUBA1B	
	USP48	TUBB	
	USP49	TUBB2A	
	USP5	TUBB2B	
	USP6NL	TUBB3	
	USP7	TUBB4B	
	USP8	TULP4	
	USP9X	TUSC2	
	UTP15	TWF2	
	UTP3	TWIST2	
	UTP6	TWISTNB	
	VAC14	TXLNA	
	VAMP2	TXLNG	
	VAMP3	TXNDC2	
	VANGL2	TXNL1	
	VAPA	TYMS	
	VAPB	U2AF1	
	VARS	U2AF2	
	VASH1	U2SURP	
	VASP	UBA1	
	VAV2	UBA2	
	VBP1	UBA3	
	VCAN	UBA52	
	VCL	UBALD1	
	VCP	UBAP1	
	VCPKMT	UBAP2L	
	VDAC1	UBE2A	
	VDAC2	UBE2D2	
	VEGFA	UBE2E1	
	VEGFB	UBE2E2	
	VEZF1	UBE2F	
	VHL	UBE2G1	
	VIM	UBE2H	
	VIPAS39	UBE2I	
	VPRBP	UBE2J2	
	VPS16	UBE2K	
	VPS25	UBE2L3	
	VPS26A	UBE2M	
	VPS26B	UBE2N	
	VPS28	UBE2O	
	VPS29	UBE2Q1	
	VPS33A	UBE2QL1	
	VPS35	UBE2R2	
	VPS36	UBE2S	
	VPS41	UBE2Z	

© 2023 Morton SU et al. *JAMA Network Open*.

	VPS4A	UBE3A	
	VPS4B	UBE3C	
	VPS51	UBE4B	
	VPS52	UBFD1	
	VPS54	UBL3	
	VPS72	UBN1	
	VRK1	UBN2	
	VTA1	UBP1	
	VWA9	UBQLN1	
	WAC	UBQLN2	
	WAPAL	UBQLN4	
	WARS	UBR2	
	WASF1	UBR3	
	WASF2	UBR4	
	WASF3	UBR5	
	WASL	UBTD1	
	WBP1	UBTF	
	WBP11	UBXN1	
	WBP1L	UBXN7	
	WBP2	UCHL1	
	WBP5	UCK2	
	WBSCR22	UFD1L	
	WDFY3	UGCG	
	WDHD1	UHMK1	
	WDR1	UHRF2	
	WDR18	ULK1	
	WDR26	UNC119B	
	WDR36	UNC13A	
	WDR4	UNC5A	
	WDR43	UNC5D	
	WDR45B	UNC79	
	WDR46	UNCX	
	WDR48	UNK	
	WDR5	UNKL	
	WDR55	UPF1	
	WDR6	UPF2	
	WDR61	UPF3B	
	WDR70	UPRT	
	WDR74	URGCP	
	WDR75	USF2	
	WDR77	USP1	
	WDR82	USP10	
	WDR89	USP11	
	WDR90	USP12	
	WDTC1	USP14	
	WEE1	USP15	
	WHSC1	USP19	
	WIBG	USP22	
	WIPI2	USP24	
	WIZ	USP25	
	WLS	USP27X	

© 2023 Morton SU et al. *JAMA Network Open*.

	WNK1	USP3	
	WNT5A	USP32	
	WNT7B	USP33	
	WRB	USP34	
	WSB1	USP37	
	WTIP	USP42	
	WWC1	USP46	
	XAB2	USP47	
	XBP1	USP48	
	XPNPEP1	USP49	
	XPO1	USP5	
	XPO5	USP7	
	XPO6	USP8	
	XPO7	USP9X	
	XPOT	UTF1	
	XRCC1	UTP14A	
	XRCC3	UTP15	
	XRCC5	UTY	
	XRN2	UVRAG	
	XXYLT1	UXT	
	YAF2	VAMP2	
	YAP1	VANGL2	
	YARS	VAPB	
	YBX1	VASN	
	YBX3	VASP	
	YEATS2	VAV1	
	YEATS4	VAX1	
	YIF1B	VBP1	
	YIPF2	VCAM1	
	YIPF3	VCAN	
	YIPF5	VCP	
	YKT6	VCPIP1	
	YLPM1	VDAC1	
	YME1L1	VDAC2	
	YRDC	VDAC3	
	YTHDC1	VEZF1	
	YTHDF1	VGF	
	YTHDF2	VGLL1	
	YTHDF3	VGLL2	
	YWHAB	VIM	
	YWHAE	VKORC1L1	
	YWHAG	VMA21	
	YWHAH	VMP1	
	YWHAQ	VPRBP	
	YWHAZ	VPS26A	
	YY1	VPS26B	
	YY1AP1	VPS29	
	ZBED3	VPS35	
	ZBTB12	VPS37D	
	ZBTB16	VPS4A	
	ZBTB18	VPS54	

© 2023 Morton SU et al. *JAMA Network Open*.

	ZBTB39	VRTN	
	ZC3H11A	VWC2	
	ZC3H13	WAC	
	ZC3H14	WAPAL	
	ZC3H15	WAS	
	ZC3H18	WASF1	
	ZC3H3	WASF2	
	ZC3H4	WASF3	
	ZC3H7B	WASL	
	ZC3HAV1L	WBP11	
	ZC3HC1	WBP1L	
	ZCCHC11	WDFY3	
	ZCCHC14	WDR1	
	ZCCHC3	WDR13	
	ZCCHC8	WDR18	
	ZCRB1	WDR20	
	ZDHHC16	WDR26	
	ZDHHC3	WDR33	
	ZDHHC5	WDR37	
	ZDHHC8	WDR43	
	ZEB1	WDR44	
	ZFAND3	WDR45	
	ZFAND5	WDR48	
	ZFAND6	WDR5	
	ZFP36L1	WDR7	
	ZFP36L2	WDR82	
	ZFP41	WDTC1	
	ZFP57	WEE1	
	ZFP62	WFDC2	
	ZFP91	WHSC1	
	ZFP91-CNTF	WHSC1L1	
	ZFPL1	WIPF1	
	ZFR	WIZ	
	ZFYVE21	WNK1	
	ZGPAT	WNK2	
	ZHX1-C8ORF76	WNK3	
	ZIC1	WNT3	
	ZIC2	WNT3A	
	ZIK1	WNT5A	
	ZKSCAN4	WNT5B	
	ZMAT2	WNT9A	
	ZMAT3	WNT9B	
	ZMIZ1	WSB2	
	ZMIZ2	WSCD2	
	ZMYM2	WT1	
	ZMYM3	WTAP	
	ZMYM4	WWC3	
	ZMYND11	WWP1	
	ZMYND19	WWTR1	
	ZMYND8	XAB2	
	ZNF106	XIAP	

© 2023 Morton SU et al. *JAMA Network Open*.

	ZNF12	XK	
	ZNF124	XKR4	
	ZNF146	XKR6	
	ZNF207	XKR7	
	ZNF219	XPO1	
	ZNF22	XPO4	
	ZNF232	XPO5	
	ZNF24	XPO6	
	ZNF260	XPO7	
	ZNF275	XPOT	
	ZNF28	XPR1	
	ZNF280B	XRCC5	
	ZNF280C	XRCC6	
	ZNF281	XRN1	
	ZNF320	XYLT1	
	ZNF322	XYLT2	
	ZNF334	YAF2	
	ZNF335	YAP1	
	ZNF354C	YBX1	
	ZNF358	YBX2	
	ZNF362	YEATS2	
	ZNF385A	YIPF5	
	ZNF395	YIPF6	
	ZNF397	YLPM1	
	ZNF414	YPEL1	
	ZNF415	YPEL2	
	ZNF423	YTHDC1	
	ZNF426	YTHDC2	
	ZNF445	YTHDF1	
	ZNF451	YTHDF2	
	ZNF462	YTHDF3	
	ZNF468	YWHAB	
	ZNF48	YWHAE	
	ZNF480	YWHAG	
	ZNF488	YWHAH	
	ZNF496	YWHAQ	
	ZNF507	YWHAZ	
	ZNF512	YY1	
	ZNF512B	ZAP70	
	ZNF516	ZBED4	
	ZNF518B	ZBED5	
	ZNF521	ZBTB1	
	ZNF532	ZBTB10	
	ZNF534	ZBTB12	
	ZNF561	ZBTB16	
	ZNF562	ZBTB17	
	ZNF574	ZBTB18	
	ZNF578	ZBTB2	
	ZNF580	ZBTB20	
	ZNF598	ZBTB21	
	ZNF608	ZBTB33	

© 2023 Morton SU et al. *JAMA Network Open*.

	ZNF609	ZBTB34	
	ZNF610	ZBTB37	
	ZNF611	ZBTB38	
	ZNF618	ZBTB4	
	ZNF629	ZBTB43	
	ZNF639	ZBTB44	
	ZNF646	ZBTB46	
	ZNF664	ZBTB47	
	ZNF677	ZBTB5	
	ZNF687	ZBTB7A	
	ZNF692	ZBTB8OS	
	ZNF701	ZC3H11A	
	ZNF706	ZC3H12B	
	ZNF710	ZC3H12C	
	ZNF740	ZC3H13	
	ZNF746	ZC3H14	
	ZNF76	ZC3H15	
	ZNF765	ZC3H18	
	ZNF766	ZC3H4	
	ZNF768	ZC3H7A	
	ZNF771	ZC3H7B	
	ZNF780B	ZC4H2	
	ZNF813	ZCCHC11	
	ZNF816	ZCCHC14	
	ZNF83	ZCCHC2	
	ZNF860	ZCCHC24	
	ZNF880	ZCCHC6	
	ZRANB2	ZCCHC8	
	ZSCAN21	ZDHHC14	
	ZSWIM4	ZDHHC15	
	ZSWIM5	ZDHHC17	
	ZSWIM6	ZDHHC5	
	ZSWIM8	ZDHHC8	
	ZWILCH	ZDHHC9	
	ZWINT	ZEB1	
	ZYX	ZEB2	
	ZZZ3	ZER1	
		ZFAND3	
		ZFAND5	
		ZFC3H1	
		ZFHX2	
		ZFHX3	
		ZFHX4	
		ZFP36	
		ZFP36L1	
		ZFP36L2	
		ZFP62	
		ZFP82	
		ZFP91	
		ZFP92	
		ZFPM2	

© 2023 Morton SU et al. *JAMA Network Open*.

		ZFR	
		ZFX	
		ZFY	
		ZFYVE9	
		ZG16	
		ZHX1	
		ZIC1	
		ZIC2	
		ZIC3	
		ZIC5	
		ZKSCAN1	
		ZMAT2	
		ZMIZ1	
		ZMIZ2	
		ZMYM2	
		ZMYM3	
		ZMYM4	
		ZMYND11	
		ZMYND19	
		ZMYND8	
		ZNF101	
		ZNF131	
		ZNF146	
		ZNF148	
		ZNF182	
		ZNF202	
		ZNF217	
		ZNF219	
		ZNF236	
		ZNF24	
		ZNF280C	
		ZNF281	
		ZNF287	
		ZNF292	
		ZNF296	
		ZNF319	
		ZNF326	
		ZNF331	
		ZNF358	
		ZNF362	
		ZNF367	
		ZNF384	
		ZNF385D	
		ZNF407	
		ZNF423	
		ZNF428	
		ZNF444	
		ZNF445	
		ZNF449	
		ZNF460	
		ZNF462	

© 2023 Morton SU et al. *JAMA Network Open*.

		ZNF469	
		ZNF483	
		ZNF496	
		ZNF503	
		ZNF512	
		ZNF512B	
		ZNF513	
		ZNF516	
		ZNF521	
		ZNF526	
		ZNF532	
		ZNF536	
		ZNF541	
		ZNF563	
		ZNF579	
		ZNF580	
		ZNF592	
		ZNF598	
		ZNF608	
		ZNF609	
		ZNF618	
		ZNF625	
		ZNF628	
		ZNF629	
		ZNF638	
		ZNF644	
		ZNF652	
		ZNF654	
		ZNF687	
		ZNF691	
		ZNF704	
		ZNF706	
		ZNF710	
		ZNF711	
		ZNF746	
		ZNF750	
		ZNF770	
		ZNF777	
		ZNF784	
		ZNF787	
		ZNF800	
		ZNF81	
		ZNF813	
		ZNF827	
		ZNF850	
		ZNF865	
		ZNFX1	
		ZNRF1	
		ZNRF3	
		ZRANB1	
		ZRANB2	

© 2023 Morton SU et al. *JAMA Network Open*.

		ZRSR2	
		ZSWIM6	
		ZSWIM8	
		ZXDB	
		ZYG11B	
		ZZEF1	
		ZZZ3	

© 2023 Morton SU et al. *JAMA Network Open*.

eTable 2: Gene variants

Type	Chromosome	Position	Reference	Alternative	Class	Gene	Amino acid change	chromatin_gene	HBE_gene	NDD_gene	pLI_gene
pLOF	1	878099	G	GC	lof	SAMD11	p.A409fs	FALSE	FALSE	FALSE	FALSE
pLOF	1	900382	C	A	lof	KLHL17	p.Y580X	FALSE	TRUE	FALSE	FALSE
pLOF	1	2116727	TC	T	lof	FAAP20	p.E145fs	FALSE	FALSE	FALSE	FALSE
dDNV/pLOF	1	2337260	AG	A	lof	PEX10	p.Leu329fs	FALSE	FALSE	FALSE	FALSE
pLOF	1	3385463	T	TG	lof	ARHGEF16	p.L275fs	FALSE	FALSE	FALSE	FALSE
pLOF	1	3415312	G	C	lof	MEGF6	p.S1058X	FALSE	TRUE	FALSE	FALSE
pLOF	1	3679753	G	T	lof	CCDC27	p.Glu346*	FALSE	FALSE	FALSE	FALSE
pLOF	1	6270017	C	T	lof	RNF207	p.Q263X	FALSE	FALSE	FALSE	FALSE
pLOF	1	11718389	C	T	lof	FBXO44	p.R111X	FALSE	FALSE	FALSE	FALSE
pLOF	1	12082289	C	CT	lof	MIIP	p.Arg85fs	FALSE	FALSE	FALSE	FALSE
pLOF	1	12815741	G	A	lof	C1orf158	p.W68X	FALSE	FALSE	FALSE	FALSE
pLOF	1	16268676	G	A	lof	ZBTB17	p.Q734X	FALSE	FALSE	FALSE	TRUE
pLOF	1	17331558	AG	A	lof	ATP13A2	p.S99fs	FALSE	TRUE	FALSE	FALSE
pLOF	1	17698832	C	CA	lof	PADI6	p.T31fs	FALSE	FALSE	FALSE	FALSE
dDNV	1	17982581	G	A	misD	ARHGEF10L	p.G897R	FALSE	FALSE	FALSE	FALSE
dDNV	1	18960820	C	T	misD	PAX7	p.Arg37Trp	TRUE	TRUE	FALSE	FALSE
pLOF	1	19493548	G	T	lof	UBR4	p.Y1359X	FALSE	TRUE	FALSE	TRUE
dDNV	1	23760827	G	A	misD	ASAP3	p.T624M	FALSE	FALSE	FALSE	FALSE
pLOF	1	24201921	G	A	lof	CNR2	p.Q63X	FALSE	FALSE	FALSE	FALSE
pLOF	1	26314721	C	T	lof	PAFAH2	.	FALSE	FALSE	FALSE	FALSE
pLOF	1	27120882	A	AG	lof	PIGV	p.V119fs	FALSE	FALSE	FALSE	FALSE
pLOF	1	32126666	C	T	lof	COL16A1	.	FALSE	FALSE	FALSE	FALSE
pLOF	1	32673556	GA	G	lof	IQCC	p.G425fs	FALSE	FALSE	FALSE	FALSE

© 2023 Morton SU et al. *JAMA Network Open*.

pLOF	1	36921868	C	A	lof	MRPS15	p.G186X	FALSE	TRUE	FALSE	FALSE
pLOF	1	43111990	T	C	lof	CCDC30	.	FALSE	FALSE	FALSE	FALSE
pLOF	1	43621982	T	A	lof	FAM183A	p.X135K	FALSE	FALSE	FALSE	FALSE
pLOF	1	44482802	TCATTC	T	lof	SLC6A9	.	FALSE	FALSE	FALSE	FALSE
pLOF	1	45286377	C	T	lof	PTCH2	.	FALSE	FALSE	FALSE	FALSE
pLOF	1	45288993	GC	G	lof	PTCH2	p.A1060fs	FALSE	FALSE	FALSE	FALSE
dDNV	1	46976691	G	C	misD	DMBX1	p.E140Q	FALSE	FALSE	FALSE	TRUE
pLOF	1	47155310	AGGTGACGGAATGATCT	A	lof	EFCAB14	p.Q243fs	FALSE	FALSE	FALSE	FALSE
pLOF	1	51584441	C	T	lof	C1orf185	p.R76X	FALSE	FALSE	FALSE	FALSE
pLOF	1	53287121	T	G	lof	ZYG11B	p.Tyr685*	FALSE	FALSE	FALSE	TRUE
pLOF	1	53442354	G	C	lof	SCP2	.	FALSE	FALSE	FALSE	FALSE
pLOF	1	54661184	AG	A	lof	CYB5RL	p.P35fs	FALSE	FALSE	FALSE	FALSE
pLOF	1	55472702	G	A	lof	BSND	p.W102X	FALSE	FALSE	FALSE	FALSE
pLOF	1	62672651	G	GA	lof	L1TD1	p.G117fs	FALSE	FALSE	FALSE	FALSE
pLOF	1	63988799	C	T	lof	ITGB3BP	.	TRUE	FALSE	FALSE	FALSE
pLOF	1	72748169	C	T	lof	NEGR1	p.Met1?	FALSE	FALSE	FALSE	TRUE
pLOF	1	76378513	A	T	lof	MSH4	p.K918X	FALSE	FALSE	FALSE	FALSE
pLOF	1	86611813	GC	G	lof	COL24A1	p.A37fs	FALSE	FALSE	FALSE	FALSE
pLOF	1	93091493	T	C	lof	EVI5	.	FALSE	FALSE	FALSE	FALSE
pLOF	1	95290047	A	G	lof	SLC44A3	.	FALSE	FALSE	FALSE	FALSE

© 2023 Morton SU et al. *JAMA Network Open*.

pLOF	1	95712211	AAGGTATAATTTAGTACTATTGCAGATGGAAAAGCAACTGAATCGATAATTATACTCTGACAAATCTAGGCATATTCATGAGTTTCTTTTGTATAATGC	A	lof	RWDD3	p.R215fs	FALSE	FALSE	FALSE	FALSE
pLOF	1	1.01E+08	C	G	lof	CDC14A	p.Y139X	FALSE	FALSE	FALSE	FALSE
pLOF	1	1.08E+08	G	A	lof	VAV3	p.R584X	FALSE	FALSE	FALSE	FALSE
pLOF	1	1.09E+08	T	TA	lof	FNDC7	p.I463fs	FALSE	FALSE	FALSE	FALSE
pLOF	1	1.1E+08	A	AG	lof	EPS8L3	p.L470fs	FALSE	FALSE	FALSE	FALSE
pLOF	1	1.12E+08	A	G	lof	FAM212B	p.X283R	FALSE	FALSE	FALSE	FALSE
pLOF	1	1.13E+08	AT	A	lof	CTTNBP2NL	p.Ser549fs	FALSE	FALSE	FALSE	FALSE
pLOF	1	1.19E+08	A	T	lof	SPAG17	p.Y1558X	FALSE	FALSE	FALSE	FALSE
pLOF	1	1.19E+08	A	AG	lof	SPAG17	p.P1375fs	FALSE	FALSE	FALSE	FALSE
pLOF	1	1.2E+08	C	A	lof	NOTCH2	.	FALSE	TRUE	FALSE	TRUE
pLOF	1	1.45E+08	C	A	lof	PDE4DIP	p.E330X	FALSE	FALSE	FALSE	FALSE
pLOF	1	1.45E+08	C	CT	lof	TXNIP	p.Val180fs	FALSE	FALSE	FALSE	FALSE
pLOF	1	1.45E+08	TG	T	lof	ANKRD34A	p.G492fs	FALSE	FALSE	FALSE	TRUE
pLOF	1	1.47E+08	T	TA	lof	CHD1L	p.L36fs	TRUE	FALSE	FALSE	FALSE
pLOF	1	1.5E+08	AG	A	lof	SV2A	p.A226fs	FALSE	FALSE	FALSE	FALSE

© 2023 Morton SU et al. *JAMA Network Open*.

pLOF	1	1.51E+08	G	A	lof	C1orf56	p.W323X	FALSE	FALSE	FALSE	FALSE
pLOF	1	1.51E+08	G	A	lof	PI4KB	p.R333X	FALSE	FALSE	FALSE	TRUE
dDNV/pLOF	1	1.52E+08	C	T	lof	OAZ3	UNKNOWN	FALSE	FALSE	FALSE	FALSE
pLOF	1	1.53E+08	C	T	lof	IVL	p.Q446X	FALSE	FALSE	FALSE	FALSE
pLOF	1	1.54E+08	G	A	lof	SLC39A1	p.Q66X	FALSE	FALSE	FALSE	FALSE
pLOF	1	1.54E+08	C	T	lof	TPM3	NA	FALSE	FALSE	FALSE	FALSE
pLOF	1	1.54E+08	G	A	lof	C1orf43	p.R65X	FALSE	FALSE	FALSE	FALSE
pLOF	1	1.54E+08	G	C	lof	IL6R	.	FALSE	FALSE	FALSE	FALSE
pLOF	1	1.55E+08	C	G	lof	EFNA4	p.Y94X	FALSE	FALSE	FALSE	FALSE
pLOF	1	1.57E+08	CT	C	lof	MRPL24	p.K100fs	FALSE	TRUE	FALSE	FALSE
pLOF	1	1.61E+08	CTG	C	lof	CD84	p.Q139fs	FALSE	FALSE	FALSE	FALSE
pLOF	1	1.68E+08	C	A	lof	GPR161	p.E527X	FALSE	FALSE	FALSE	FALSE
pLOF	1	1.7E+08	TG	T	lof	SELL	p.P216fs	FALSE	FALSE	FALSE	FALSE
pLOF	1	1.7E+08	C	A	lof	SELE	NA	FALSE	FALSE	FALSE	FALSE
pLOF	1	1.72E+08	AG	A	lof	METTL13	p.R49fs	FALSE	TRUE	FALSE	FALSE
dDNV	1	1.72E+08	C	G	misD	DNM3	p.P747A	FALSE	FALSE	FALSE	FALSE
dDNV	1	1.77E+08	A	C	misD	PAPPA2	p.N1746T	FALSE	FALSE	FALSE	FALSE
dDNV	1	1.79E+08	G	T	misD	ABL2	p.S394Y	FALSE	FALSE	FALSE	FALSE
pLOF	1	1.79E+08	TC	T	lof	AXDND1	p.P94fs	FALSE	FALSE	FALSE	FALSE
pLOF	1	1.8E+08	GC	G	lof	CEP350	p.Ala1896fs	FALSE	FALSE	FALSE	TRUE
pLOF	1	1.84E+08	TG	T	lof	NCF2	p.H302fs	FALSE	FALSE	FALSE	FALSE
pLOF	1	1.84E+08	C	T	lof	RGL1	p.Q519X	FALSE	FALSE	FALSE	TRUE
pLOF	1	1.97E+08	C	A	lof	CFHR4	p.C73X	FALSE	FALSE	FALSE	FALSE
pLOF	1	2.01E+08	A	T	lof	CACNA1S	p.C1338X	FALSE	FALSE	FALSE	FALSE
pLOF	1	2.01E+08	C	T	lof	CACNA1S	p.W417X	FALSE	FALSE	FALSE	FALSE
pLOF	1	2.01E+08	C	T	lof	IGFN1	p.Q404X	FALSE	FALSE	FALSE	FALSE

© 2023 Morton SU et al. *JAMA Network Open*.

pLOF	1	2.01E+08	AC	A	lof	LAD1	.	FALSE	FALSE	FALSE	FALSE
pLOF	1	2.03E+08	C	T	lof	PPP1R12B	p.R207X	FALSE	FALSE	FALSE	FALSE
dDNV	1	2.03E+08	G	A	misD	MYOG	p.T189M	FALSE	FALSE	FALSE	TRUE
pLOF	1	2.03E+08	AG	A	lof	ADORA1	p.Val174fs	FALSE	FALSE	FALSE	FALSE
pLOF	1	2.03E+08	T	C	lof	MYBPH	.	FALSE	FALSE	FALSE	FALSE
dDNV	1	2.04E+08	G	T	misD	ATP2B4	p.D621Y	FALSE	FALSE	FALSE	FALSE
pLOF	1	2.04E+08	C	T	lof	PIK3C2B	p.W256X	FALSE	FALSE	FALSE	TRUE
pLOF	1	2.06E+08	A	AC	lof	PM20D1	p.S207fs	FALSE	FALSE	FALSE	FALSE
dDNV	1	2.08E+08	G	A	misD	CR2	p.C154Y	FALSE	FALSE	FALSE	FALSE
pLOF	1	2.08E+08	A	G	lof	CR2	NA	FALSE	FALSE	FALSE	FALSE
pLOF	1	2.1E+08	T	TA	lof	LAMB3	p.Y27fs	FALSE	FALSE	FALSE	FALSE
pLOF	1	2.12E+08	AG	A	lof	TRAF5	p.G9fs	FALSE	FALSE	FALSE	FALSE
pLOF	1	2.16E+08	AG	A	lof	USH2A	p.L3567X	FALSE	FALSE	FALSE	FALSE
pLOF	1	2.16E+08	CT	C	lof	USH2A	p.P1145fs	FALSE	FALSE	FALSE	FALSE
pLOF	1	2.23E+08	CCCT	C	lof	HHIPL2	p.E468fs	FALSE	FALSE	FALSE	FALSE
pLOF	1	2.23E+08	G	T	lof	FAM177B	.	FALSE	FALSE	FALSE	FALSE
dDNV	1	2.23E+08	G	A	misD	DISP1	p.R631Q	FALSE	FALSE	FALSE	FALSE
pLOF	1	2.25E+08	TA	T	lof	DNAH14	p.L1805fs	FALSE	FALSE	FALSE	FALSE
dDNV/pLOF	1	2.25E+08	G	A	lof	DNAH14	p.Trp1808*	FALSE	FALSE	FALSE	FALSE
pLOF	1	2.26E+08	C	T	lof	EPHX1	p.R338X	FALSE	FALSE	FALSE	FALSE
pLOF	1	2.27E+08	G	T	lof	ADCK3	p.E634X	FALSE	FALSE	FALSE	FALSE
pLOF	1	2.28E+08	C	A	lof	C1orf35	.	FALSE	FALSE	FALSE	FALSE
pLOF	1	2.28E+08	TCA	T	lof	OBSCN	p.V3251fs	FALSE	FALSE	FALSE	FALSE
pLOF	1	2.3E+08	T	C	lof	URB2	NA	FALSE	FALSE	FALSE	FALSE
dDNV	1	2.31E+08	T	C	misD	GNPAT	p.I262T	FALSE	TRUE	FALSE	FALSE
pLOF	1	2.32E+08	G	A	lof	DISC1	p.W670X	FALSE	FALSE	TRUE	FALSE
pLOF	1	2.33E+08	G	GA	lof	PCNXL2	p.F1772fs	FALSE	FALSE	FALSE	FALSE

pLOF	1	2.35E+08	T	TAC	lof	TARBP1	p.T1246fs	FALSE	FALSE	FALSE	FALSE
dDNV	1	2.38E+08	A	G	misD	RYR2	p.Met4058Val	FALSE	FALSE	FALSE	TRUE
pLOF	1	2.38E+08	G	A	lof	ZP4	p.Q236X	FALSE	FALSE	FALSE	FALSE
pLOF	1	2.42E+08	G	A	lof	CHML	p.Gln473*	FALSE	FALSE	FALSE	FALSE
pLOF	1	2.49E+08	C	CAA	lof	ZNF672	p.Arg41fs	FALSE	FALSE	FALSE	FALSE
dDNV/pLOF	1	2.49E+08	AGCGCCACGCTGGT	A	lof	ZNF672	p.S239fs	FALSE	FALSE	FALSE	FALSE
pLOF	2	10199057	C	A	lof	CYS1	p.E134X	FALSE	FALSE	FALSE	FALSE
pLOF	2	11960624	AAG	A	lof	LPIN1	p.K918fs	TRUE	FALSE	FALSE	FALSE
pLOF	2	21232182	G	A	lof	APOB	p.R2520X	FALSE	FALSE	FALSE	FALSE
pLOF	2	25964922	GCA	G	lof	ASXL2	p.C1428fs	TRUE	FALSE	TRUE	TRUE
pLOF	2	27293033	TC	T	lof	AGBL5	p.S855fs	FALSE	FALSE	FALSE	FALSE
dDNV	2	27445073	C	T	misD	CAD	p.R122W	FALSE	TRUE	TRUE	TRUE
pLOF	2	27706245	T	TA	lof	IFT172	.	FALSE	TRUE	FALSE	FALSE
pLOF	2	28812887	C	T	lof	PLB1	p.Q678X	FALSE	FALSE	FALSE	FALSE
pLOF	2	29287883	CA	C	lof	C2orf71	p.Cys1240fs	FALSE	FALSE	FALSE	FALSE
pLOF	2	29606630	AC	A	lof	ALK	p.V417fs	FALSE	FALSE	FALSE	FALSE
pLOF	2	30986012	G	GT	lof	CAPN13	p.T237fs	FALSE	FALSE	FALSE	FALSE
pLOF	2	37032808	G	A	lof	VIT	.	FALSE	FALSE	FALSE	FALSE
pLOF	2	37321291	G	T	lof	GPATCH11	p.E225X	FALSE	FALSE	FALSE	FALSE
pLOF	2	37543642	G	C	lof	PRKD3	p.S9X	FALSE	TRUE	FALSE	FALSE
dDNV/pLOF	2	47136161	C	G	lof	MCFD2	NA	FALSE	TRUE	FALSE	FALSE
pLOF	2	48915301	G	T	lof	LHCGR	p.C545X	FALSE	FALSE	FALSE	FALSE
pLOF	2	55253349	GCACC	G	lof	RTN4	p.Gly628fs	FALSE	TRUE	FALSE	FALSE
pLOF	2	55253354	A	AT	lof	RTN4	p.Gly628fs	FALSE	TRUE	FALSE	FALSE

© 2023 Morton SU et al. *JAMA Network Open*.

pLOF	2	55872531	GT	G	lof	PNPT1	p.T592fs	FALSE	TRUE	FALSE	FALSE
pLOF	2	68740211	G	A	lof	APLF	.	FALSE	FALSE	FALSE	FALSE
pLOF	2	70488452	G	A	lof	PCYOX1	p.W143X	FALSE	TRUE	FALSE	FALSE
pLOF	2	74652826	T	C	lof	WDR54	p.X335R	FALSE	FALSE	FALSE	FALSE
pLOF	2	74758465	G	A	lof	HTRA2	.	FALSE	TRUE	FALSE	FALSE
pLOF	2	75921366	C	A	lof	GCFC2	.	FALSE	FALSE	FALSE	FALSE
pLOF	2	88474314	TC	T	lof	THNSL2	p.F127fs	FALSE	FALSE	FALSE	FALSE
pLOF	2	88874920	G	T	lof	EIF2AK3	p.S694X	FALSE	FALSE	FALSE	FALSE
pLOF	2	1.03E+08	GA	G	lof	IL1RL1	p.R62fs	FALSE	FALSE	FALSE	FALSE
pLOF	2	1.1E+08	A	AG	lof	SH3RF3	p.G540fs	FALSE	FALSE	FALSE	FALSE
dDNV	2	1.28E+08	C	T	misD	MYO7B	p.R2003W	FALSE	FALSE	FALSE	FALSE
pLOF	2	1.28E+08	AC	A	lof	LIMS2	.	FALSE	FALSE	FALSE	FALSE
pLOF	2	1.31E+08	GC	G	lof	PTPN18	p.Pro67fs	FALSE	FALSE	FALSE	FALSE
pLOF	2	1.32E+08	AG	A	lof	AMER3	p.G823fs	FALSE	FALSE	FALSE	TRUE
dDNV	2	1.32E+08	C	T	misD	ARHGEF4	p.P423L	FALSE	FALSE	FALSE	FALSE
pLOF	2	1.33E+08	G	T	lof	GPR39	p.Glu416*	FALSE	FALSE	FALSE	FALSE
dDNV/pLOF	2	1.36E+08	G	A	lof	MAP3K19	p.R702X	FALSE	FALSE	FALSE	FALSE
pLOF	2	1.36E+08	CAG	C	lof	ZRANB3	p.L586fs	FALSE	FALSE	FALSE	FALSE
pLOF	2	1.38E+08	C	T	lof	THSD7B	p.R723X	FALSE	FALSE	FALSE	FALSE
pLOF	2	1.39E+08	A	C	lof	HNMT	.	FALSE	FALSE	FALSE	FALSE
dDNV	2	1.41E+08	C	T	misD	LRP1B	p.S3662N	FALSE	FALSE	FALSE	FALSE
pLOF	2	1.52E+08	G	A	lof	NMI	p.Q247X	FALSE	FALSE	FALSE	FALSE
pLOF	2	1.52E+08	C	CTTGA	lof	NEB	p.E8100fs	FALSE	FALSE	FALSE	FALSE
pLOF	2	1.57E+08	AT	A	lof	GPD2	p.Ile703fs	FALSE	FALSE	FALSE	FALSE
pLOF	2	1.59E+08	C	T	lof	ACVR1	p.W478X	FALSE	FALSE	FALSE	FALSE
pLOF	2	1.61E+08	AAATT	A	lof	LY75	p.Glu773fs	FALSE	FALSE	FALSE	FALSE
pLOF	2	1.65E+08	T	TAAAG	lof	GRB14	.	FALSE	FALSE	FALSE	FALSE

© 2023 Morton SU et al. *JAMA Network Open*.

pLOF	2	1.71E+08	A	G	lof	MYO3B	.	FALSE	FALSE	FALSE	FALSE
pLOF	2	1.74E+08	G	GA	lof	MLTK	p.Arg617fs	FALSE	FALSE	FALSE	FALSE
pLOF	2	1.75E+08	G	A	lof	GPR155	p.R764X	FALSE	FALSE	FALSE	FALSE
pLOF	2	1.79E+08	G	A	lof	TTN	p.R21604X	FALSE	FALSE	FALSE	FALSE
pLOF	2	1.89E+08	G	A	lof	GULP1	p.Trp112*	FALSE	FALSE	FALSE	TRUE
pLOF	2	2.02E+08	C	T	lof	NIF3L1	p.R241X	FALSE	TRUE	FALSE	FALSE
pLOF	2	2.04E+08	CAG	C	lof	ABI2	p.S125fs	FALSE	TRUE	FALSE	FALSE
pLOF	2	2.06E+08	A	G	lof	PARD3B	.	FALSE	FALSE	TRUE	FALSE
pLOF	2	2.07E+08	AT	A	lof	NDUFS1	p.I225fs	FALSE	TRUE	FALSE	FALSE
pLOF	2	2.09E+08	CA	C	lof	IDH1	p.L352fs	FALSE	TRUE	FALSE	FALSE
pLOF	2	2.19E+08	C	A	lof	SLC11A1	p.Y258X	FALSE	FALSE	FALSE	FALSE
pLOF	2	2.19E+08	AAGAT	A	lof	VIL1	p.K391fs	FALSE	FALSE	TRUE	FALSE
pLOF	2	2.2E+08	G	A	lof	CFAP65	p.R922X	FALSE	FALSE	FALSE	FALSE
pLOF	2	2.2E+08	G	A	lof	CNPPD1	p.R76X	FALSE	FALSE	FALSE	FALSE
pLOF	2	2.28E+08	T	G	lof	COL4A3	p.Y283X	FALSE	FALSE	FALSE	FALSE
pLOF	2	2.28E+08	G	A	lof	COL4A3	.	FALSE	FALSE	FALSE	FALSE
pLOF	2	2.31E+08	C	A	lof	SP110	.	TRUE	FALSE	FALSE	FALSE
pLOF	2	2.32E+08	G	T	lof	CAB39	p.E340X	FALSE	TRUE	FALSE	FALSE
dDNV	2	2.34E+08	C	T	misD	DGKD	p.Arg962Cys	FALSE	TRUE	FALSE	FALSE
dDNV	2	2.35E+08	T	A	misD	UGT1A6	p.L237H	FALSE	FALSE	FALSE	FALSE
dDNV/pLOF	2	2.35E+08	A	AT	lof	TRPM8	p.Y235fs	FALSE	FALSE	FALSE	FALSE
pLOF	2	2.38E+08	ACGCCAGT	A	lof	PRLH	p.Y42fs	FALSE	FALSE	FALSE	FALSE
pLOF	2	2.39E+08	G	GC	lof	FAM132B	p.Gly124fs	FALSE	FALSE	FALSE	FALSE
pLOF	2	2.41E+08	C	CGTGGATGAAGAGCT	lof	PRR21	p.R12fs	FALSE	FALSE	FALSE	FALSE

© 2023 Morton SU et al. *JAMA Network Open*.

pLOF	2	2.42E+08	AG	A	lof	PASK	p.Trp241fs	FALSE	FALSE	FALSE	FALSE
pLOF	2	2.42E+08	TTC	T	lof	STK25	p.E371fs	FALSE	TRUE	FALSE	FALSE
pLOF	3	9695344	C	T	lof	MTMR14	p.R67X	FALSE	TRUE	FALSE	FALSE
pLOF	3	9877003	GA	G	lof	TTLL3	p.D860fs	FALSE	FALSE	FALSE	FALSE
pLOF	3	9979296	T	TC	lof	CRELD1	p.Glu104fs	FALSE	FALSE	FALSE	FALSE
pLOF	3	10254918	GA	G	lof	IRAK2	p.E186fs	FALSE	FALSE	FALSE	FALSE
pLOF	3	10320063	T	TG	lof	TATDN2	p.W688fs	FALSE	FALSE	FALSE	FALSE
pLOF	3	12780071	CA	C	lof	TMEM40	p.V124fs	FALSE	FALSE	FALSE	FALSE
pLOF	3	14200140	G	A	lof	XPC	p.R415X	FALSE	FALSE	FALSE	FALSE
pLOF	3	14745975	A	AC	lof	C3orf20	p.Y337fs	FALSE	FALSE	FALSE	FALSE
pLOF	3	20042879	G	A	lof	PP2D1	p.Q245X	FALSE	FALSE	FALSE	FALSE
dDNV	3	27333034	G	C	misD	NEK10	p.H473D	FALSE	FALSE	FALSE	FALSE
dDNV/pLOF	3	32611912	C	G	lof	DYNC1LI1	NA	FALSE	TRUE	FALSE	TRUE
pLOF	3	36879869	CA	C	lof	TRANK1	p.L1793fs	FALSE	FALSE	FALSE	FALSE
dDNV	3	38592918	C	G	misD	SCN5A	p.Ala1649Pro	FALSE	FALSE	FALSE	TRUE
pLOF	3	38793799	G	A	lof	SCN10A	p.Q556X	FALSE	FALSE	FALSE	FALSE
pLOF	3	39374845	C	A	lof	CCR8	p.C341X	FALSE	FALSE	FALSE	FALSE
pLOF	3	43759162	G	A	lof	ABHD5	.	FALSE	FALSE	FALSE	FALSE
pLOF	3	48602273	TC	T	lof	COL7A1	p.G2920fs	FALSE	FALSE	FALSE	FALSE
pLOF	3	48716340	C	A	lof	NCKIPSD	p.E581X	FALSE	FALSE	FALSE	FALSE
pLOF	3	49053766	AG	A	lof	DALRD3	p.Leu385fs	FALSE	TRUE	FALSE	FALSE
pLOF	3	49163457	CAGAG	C	lof	LAMB2	p.P761fs	FALSE	TRUE	FALSE	FALSE
pLOF	3	49850818	T	C	lof	UBA7	.	FALSE	TRUE	FALSE	FALSE
pLOF	3	51990710	A	AC	lof	GPR62	p.T348fs	FALSE	FALSE	FALSE	FALSE
pLOF	3	52430632	A	G	lof	DNAH1	.	FALSE	FALSE	FALSE	FALSE
pLOF	3	52512570	C	T	lof	NISCH	p.Q427X	FALSE	TRUE	FALSE	TRUE

© 2023 Morton SU et al. *JAMA Network Open*.

pLOF	3	52512 570	C	T	lof	NISC H	p.Gln 427*	FALS E	TRUE	FALS E	TRUE
pLOF	3	52825 797	G	A	lof	ITIH1	NA	FALS E	FALS E	FALS E	FALS E
pLOF	3	56114 964	T	A	lof	ERC2	p.K50 8X	FALS E	FALS E	FALS E	TRUE
pLOF	3	57303 701	G	T	lof	APPL 1	p.E70 6X	FALS E	FALS E	FALS E	TRUE
dDNV /pLOF	3	69082 783	G	A	lof	TMF1	p.R77 3X	FALS E	FALS E	FALS E	FALS E
pLOF	3	73523 667	AT	A	lof	PDZR N3	p.Met 8fs	FALS E	TRUE	FALS E	TRUE
pLOF	3	97668 731	G	GA	lof	MINA	p.L33 9fs	FALS E	FALS E	FALS E	FALS E
pLOF	3	1.02E +08	C	T	lof	ZPLD 1	p.R28 4X	FALS E	FALS E	FALS E	FALS E
pLOF	3	1.08E +08	G	A	lof	MYH1 5	p.R18 7X	FALS E	FALS E	FALS E	FALS E
pLOF	3	1.08E +08	TG	T	lof	KIAA 1524	p.Asn 553fs	FALS E	TRUE	FALS E	FALS E
pLOF	3	1.12E +08	C	T	lof	TMP RSS7	p.R14 X	FALS E	FALS E	FALS E	FALS E
pLOF	3	1.13E +08	CTT	C	lof	GTPB P8	p.Phe 14fs	FALS E	FALS E	FALS E	FALS E
pLOF	3	1.21E +08	G	A	lof	POLQ	p.Q23 50X	FALS E	FALS E	FALS E	FALS E
pLOF	3	1.21E +08	CA	C	lof	GOL GB1	p.M22 47fs	FALS E	FALS E	FALS E	FALS E
pLOF	3	1.25E +08	G	GTC	lof	HEG1	p.Ser 7fs	FALS E	FALS E	FALS E	FALS E
pLOF	3	1.26E +08	T	TA	lof	TXNR D3NB	p.K66 _R67 delins X	FALS E	FALS E	FALS E	FALS E
pLOF	3	1.27E +08	TA	T	lof	TPRA 1	p.V30 6fs	FALS E	FALS E	FALS E	FALS E
pLOF	3	1.31E +08	C	A	lof	NEK1 1	p.Tyr 29*	TRUE	FALS E	FALS E	FALS E
pLOF	3	1.31E +08	AC	A	lof	NEK1 1	p.Y47 9X	TRUE	FALS E	FALS E	FALS E
pLOF	3	1.38E +08	G	A	lof	NME9	p.R14 2X	FALS E	FALS E	FALS E	FALS E
dDNV /pLOF	3	1.39E +08	G	C	lof	COP B2	p.S28 3X	FALS E	TRUE	FALS E	TRUE
pLOF	3	1.49E +08	GA	G	lof	CPA3	p.N21 1fs	FALS E	FALS E	FALS E	FALS E
pLOF	3	1.49E +08	G	A	lof	CPA3	.	FALS E	FALS E	FALS E	FALS E
pLOF	3	1.49E +08	C	A	lof	HPS3	p.Y56 2X	FALS E	FALS E	FALS E	FALS E
pLOF	3	1.51E +08	A	G	lof	GPR8 7	p.X35 9Q	FALS E	FALS E	FALS E	FALS E

© 2023 Morton SU et al. *JAMA Network Open*.

pLOF	3	1.51E+08	G	GT	lof	P2RY13	p.N249fs	FALSE	FALSE	FALSE	FALSE
pLOF	3	1.54E+08	C	CT	lof	GPR149	.	FALSE	FALSE	FALSE	FALSE
pLOF	3	1.55E+08	C	CT	lof	PLCH1	p.S1318fs	FALSE	FALSE	FALSE	FALSE
pLOF	3	1.59E+08	T	TGGGCGTGGCC	lof	MFSD1	p.M1fs	FALSE	TRUE	FALSE	FALSE
pLOF	3	1.6E+08	TAAAGA	T	lof	SMC4	p.K397fs	FALSE	TRUE	FALSE	FALSE
pLOF	3	1.6E+08	G	GGTGT	lof	SMC4	p.Q801fs	FALSE	TRUE	FALSE	FALSE
pLOF	3	1.6E+08	TC	T	lof	ARL14	p.I42fs	FALSE	FALSE	FALSE	FALSE
dDNV	3	1.65E+08	G	A	misD	SI	p.P475S	FALSE	FALSE	FALSE	FALSE
pLOF	3	1.66E+08	AT	A	lof	BCHE	p.N209fs	FALSE	FALSE	FALSE	FALSE
pLOF	3	1.72E+08	CA	C	lof	GHSR	p.W215fs	FALSE	FALSE	FALSE	FALSE
pLOF	3	1.79E+08	GCAGTA	G	lof	ZMAT3	p.Y71fs	FALSE	TRUE	FALSE	FALSE
pLOF	3	1.83E+08	TC	T	lof	MCF2L2	p.G187fs	FALSE	FALSE	FALSE	FALSE
pLOF	3	1.84E+08	C	A	lof	ECE2	p.S230X	FALSE	FALSE	FALSE	FALSE
pLOF	3	1.85E+08	A	G	lof	VPS8	p.Met1?	FALSE	FALSE	FALSE	FALSE
pLOF	3	1.9E+08	C	T	lof	TMEM207	.	FALSE	FALSE	FALSE	FALSE
pLOF	3	1.93E+08	TC	T	lof	ATP13A5	p.E107fs	FALSE	FALSE	FALSE	FALSE
pLOF	3	1.97E+08	C	T	lof	SENP5	p.Q269X	FALSE	FALSE	FALSE	TRUE
pLOF	3	1.98E+08	C	T	lof	IQCG	p.W330X	FALSE	FALSE	FALSE	FALSE
pLOF	4	657557	A	G	lof	PDE6B	.	FALSE	FALSE	FALSE	FALSE
pLOF	4	995352	G	A	lof	IDUA	.	FALSE	FALSE	FALSE	FALSE
pLOF	4	1165675	AG	A	lof	SPON2	p.L62fs	FALSE	FALSE	FALSE	FALSE
pLOF	4	2210193	G	A	lof	POLN	p.Q79X	FALSE	FALSE	FALSE	FALSE
pLOF	4	9909936	CT	C	lof	SLC2A9	p.K316fs	FALSE	FALSE	FALSE	FALSE
pLOF	4	22390565	C	T	lof	ADGRA3	p.W910X	FALSE	FALSE	FALSE	FALSE
pLOF	4	25270817	A	AG	lof	PI4K2B	p.R422fs	FALSE	FALSE	FALSE	FALSE
pLOF	4	38117333	C	T	lof	TBC1D1	p.R948X	FALSE	TRUE	FALSE	FALSE

© 2023 Morton SU et al. *JAMA Network Open*.

pLOF	4	42965062	G	T	lof	GRXCR1	p.Glu180*	FALSE	FALSE	FALSE	FALSE
pLOF	4	48501484	TAG	T	lof	FRYL	p.L2999fs	FALSE	FALSE	FALSE	TRUE
dDNV/pLOF	4	48584641	TAAAC	T	lof	FRYL	p.V619fs	FALSE	FALSE	FALSE	TRUE
pLOF	4	57181627	G	GCT	lof	KIAA1211	p.K653fs	FALSE	TRUE	FALSE	FALSE
pLOF	4	68930574	G	GA	lof	TMPRSS11F	p.H282fs	FALSE	FALSE	FALSE	FALSE
pLOF	4	72316150	G	GTCTTT	lof	SLC4A4	p.L366fs	FALSE	FALSE	FALSE	TRUE
pLOF	4	76532365	A	C	lof	CDKL2	.	FALSE	FALSE	FALSE	FALSE
pLOF	4	76956270	ATACTGT	A	lof	CXCL11	p.I63fs	FALSE	FALSE	FALSE	FALSE
pLOF	4	78080585	CTT	C	lof	CCNG2	p.Phe70fs	FALSE	TRUE	FALSE	FALSE
dDNV/pLOF	4	79186194	C	T	lof	FRAS1	p.Arg207*	FALSE	FALSE	FALSE	FALSE
pLOF	4	87671811	C	T	lof	PTPN13	p.Q947X	FALSE	TRUE	FALSE	FALSE
pLOF	4	87720325	TAGAG	T	lof	PTPN13	p.I2163fs	FALSE	TRUE	FALSE	FALSE
pLOF	4	90857226	GTT	G	lof	MMRN1	p.V799fs	FALSE	FALSE	FALSE	FALSE
pLOF	4	90857627	C	CA	lof	MMRN1	p.Asn933fs	FALSE	FALSE	FALSE	FALSE
pLOF	4	1E+08	A	G	lof	C4orf17	p.X360W	FALSE	FALSE	FALSE	FALSE
pLOF	4	1.07E+08	G	GAA	lof	TBCK	p.F101fs	FALSE	FALSE	TRUE	FALSE
pLOF	4	1.09E+08	C	A	lof	PAPSS1	p.E236X	FALSE	TRUE	FALSE	FALSE
pLOF	4	1.11E+08	GTCTCAAAGTGTAGGTGA	G	lof	LRIT3	p.S636fs	FALSE	FALSE	FALSE	FALSE
pLOF	4	1.14E+08	C	T	lof	ZGRF1	p.W1387X	FALSE	FALSE	FALSE	FALSE
pLOF	4	1.23E+08	G	A	lof	KIAA1109	.	FALSE	FALSE	FALSE	FALSE
pLOF	4	1.35E+08	C	A	lof	PABPC4L	p.E365X	FALSE	FALSE	FALSE	FALSE
dDNV	4	1.46E+08	G	A	misD	ABCE1	p.R22Q	FALSE	TRUE	FALSE	TRUE
pLOF	4	1.49E+08	CTA	C	lof	ARHGAP10	p.Y290fs	FALSE	FALSE	FALSE	FALSE
pLOF	4	1.52E+08	A	C	lof	PRSS48	p.X329S	FALSE	FALSE	FALSE	FALSE

© 2023 Morton SU et al. *JAMA Network Open*.

pLOF	4	1.56E+08	G	GT	lof	MAP9	p.T94fs	FALSE	FALSE	FALSE	FALSE
pLOF	4	1.57E+08	C	T	lof	GUCY1A3	p.R614X	FALSE	FALSE	FALSE	FALSE
pLOF	4	1.7E+08	AG	A	lof	SH3RF1	p.P329fs	FALSE	FALSE	FALSE	FALSE
pLOF	5	7870882	G	A	lof	MTRR	.	FALSE	FALSE	FALSE	FALSE
pLOF	5	13721167	GA	G	lof	DNAH5	p.S4074fs	FALSE	FALSE	FALSE	FALSE
pLOF	5	14711397	G	T	lof	ANKH	p.S463X	FALSE	FALSE	TRUE	FALSE
pLOF	5	16699557	A	G	lof	MYO10	.	FALSE	TRUE	FALSE	FALSE
pLOF	5	35910098	GT	G	lof	CAPSL	p.T132fs	FALSE	FALSE	FALSE	FALSE
pLOF	5	38511915	C	T	lof	LIFR	p.W238X	FALSE	FALSE	FALSE	FALSE
pLOF	5	39383375	T	A	lof	DAB2	.	FALSE	FALSE	FALSE	FALSE
pLOF	5	52365994	CA	C	lof	ITGA2	p.Arg714fs	FALSE	FALSE	FALSE	FALSE
pLOF	5	54529089	T	TGGGCCGGGCC	lof	CCNO	p.Q88fs	FALSE	FALSE	FALSE	FALSE
pLOF	5	54618194	TAATGGAAA	T	lof	SKIV2L2	p.Asn59fs	FALSE	TRUE	FALSE	TRUE
pLOF	5	63496764	A	AT	lof	RNF180	p.I44fs	FALSE	FALSE	FALSE	FALSE
pLOF	5	64020297	CT	C	lof	SREK1IP1	p.K127fs	FALSE	TRUE	FALSE	FALSE
pLOF	5	64625312	T	C	lof	ADAMTS6	.	FALSE	FALSE	FALSE	TRUE
pLOF	5	65054518	C	T	lof	NLN	p.Gln56*	FALSE	FALSE	FALSE	FALSE
pLOF	5	79029202	GA	G	lof	CMYA5	p.K1539fs	FALSE	FALSE	FALSE	FALSE
pLOF	5	94731814	G	A	lof	FAM81B	.	FALSE	FALSE	FALSE	FALSE
pLOF	5	96430453	T	A	lof	LIX1	p.X283L	FALSE	FALSE	FALSE	FALSE
pLOF	5	1.02E+08	TG	T	lof	SLCO4C1	p.T449fs	FALSE	FALSE	FALSE	FALSE
pLOF	5	1.03E+08	T	TA	lof	C5orf30	p.Arg92fs	FALSE	FALSE	FALSE	FALSE
pLOF	5	1.1E+08	C	A	lof	TMEM232	p.E115X	FALSE	FALSE	FALSE	FALSE
pLOF	5	1.11E+08	C	T	lof	STARD4	.	FALSE	FALSE	FALSE	FALSE
pLOF	5	1.12E+08	C	T	lof	REEP5	.	FALSE	FALSE	FALSE	FALSE
pLOF	5	1.12E+08	G	A	lof	MCC	p.Q810X	FALSE	FALSE	FALSE	FALSE

pLOF	5	1.15E+08	G	A	lof	LVRN	p.W544X	FALSE	FALSE	FALSE	FALSE
pLOF	5	1.26E+08	GC	G	lof	ALDH7A1	p.A462fs	FALSE	TRUE	FALSE	FALSE
pLOF	5	1.39E+08	G	A	lof	SLC23A1	p.R293X	FALSE	FALSE	FALSE	FALSE
pLOF	5	1.4E+08	G	T	lof	ANKHD1	p.E564X	FALSE	TRUE	FALSE	TRUE
pLOF	5	1.4E+08	C	T	lof	TMCO6	p.Gln252*	FALSE	FALSE	FALSE	FALSE
dDNV	5	1.4E+08	C	T	misD	PCDHA3	p.A266V	FALSE	FALSE	FALSE	FALSE
pLOF	5	1.41E+08	C	G	lof	PCDHGB7	p.Y401X	FALSE	FALSE	FALSE	FALSE
pLOF	5	1.45E+08	T	TC	lof	PLAC8L1	p.Glu155fs	FALSE	FALSE	FALSE	FALSE
pLOF	5	1.47E+08	T	A	lof	STK32A	p.Tyr223*	FALSE	FALSE	FALSE	FALSE
dDNV	5	1.5E+08	G	T	misD	ARSI	p.S217R	FALSE	FALSE	FALSE	FALSE
pLOF	5	1.5E+08	G	GC	lof	MYOZ3	p.A123fs	FALSE	FALSE	FALSE	FALSE
dDNV	5	1.68E+08	C	T	misD	SLIT3	p.Glu1162Lys	FALSE	FALSE	FALSE	TRUE
pLOF	5	1.76E+08	G	T	lof	GPRIN1	p.S40X	FALSE	FALSE	FALSE	TRUE
pLOF	5	1.77E+08	G	T	lof	FGFR4	p.G705X	FALSE	FALSE	FALSE	FALSE
pLOF	5	1.77E+08	C	A	lof	DBN1	p.Met1?	FALSE	TRUE	FALSE	TRUE
pLOF	5	1.77E+08	CCT	C	lof	B4GALT7	p.Tyr194fs	FALSE	FALSE	FALSE	FALSE
dDNV	5	1.8E+08	C	T	misD	FLT4	p.Gly781Asp	FALSE	FALSE	FALSE	TRUE
pLOF	5	1.8E+08	T	A	lof	ZFP62	p.Lys831*	FALSE	TRUE	FALSE	TRUE
pLOF	5	1.8E+08	TCC	T	lof	BTNL3	p.I208fs	FALSE	FALSE	FALSE	FALSE
pLOF	5	1.8E+08	G	A	lof	BTNL9	NA	FALSE	FALSE	FALSE	FALSE
pLOF	6	4047423	A	T	lof	PRPF4B	p.K626X	FALSE	TRUE	FALSE	TRUE
pLOF	6	5431312	AC	A	lof	FARS2	p.T271fs	FALSE	FALSE	FALSE	FALSE
pLOF	6	13616731	T	TG	lof	NOL7	p.Leu122fs	FALSE	TRUE	FALSE	FALSE
pLOF	6	24415304	TC	T	lof	MRS2	p.I214fs	FALSE	FALSE	FALSE	FALSE
pLOF	6	24570129	A	T	lof	KIAA0319	.	FALSE	FALSE	FALSE	FALSE
pLOF	6	26056362	T	TG	lof	HIST1H1C	p.Thr99fs	FALSE	FALSE	FALSE	FALSE

© 2023 Morton SU et al. *JAMA Network Open*.

pLOF	6	28331535	C	T	lof	ZKSCAN3	p.Q234X	FALSE	FALSE	FALSE	FALSE
dDNV/pLOF	6	32007337	GC	G	lof	CYP21A2	0	FALSE	FALSE	FALSE	FALSE
pLOF	6	32064726	TG	T	lof	TNXB	p.Y301X	FALSE	FALSE	FALSE	FALSE
pLOF	6	38885167	G	A	lof	DNAH8	p.Trp3419*	FALSE	FALSE	FALSE	FALSE
pLOF	6	38885727	G	A	lof	DNAH8	p.Trp3433*	FALSE	FALSE	FALSE	FALSE
pLOF	6	39387746	TA	T	lof	KIF6	p.I596fs	FALSE	FALSE	FALSE	FALSE
pLOF	6	40400345	G	A	lof	LRFN2	p.R170X	FALSE	FALSE	FALSE	FALSE
pLOF	6	41117598	AAAGGCAATTCAGC	A	lof	TREML1	p.Ala223fs	FALSE	FALSE	FALSE	FALSE
dDNV/pLOF	6	41884521	A	G	lof	MED20	NA	FALSE	TRUE	FALSE	FALSE
pLOF	6	42928596	TG	T	lof	GNMT	p.W31fs	FALSE	FALSE	FALSE	FALSE
pLOF	6	43010706	G	GTT	lof	CUL7	p.T1160fs	FALSE	TRUE	TRUE	FALSE
dDNV/pLOF	6	44328227	AT	A	lof	SPATS1	NA	FALSE	FALSE	FALSE	FALSE
pLOF	6	44328227	AT	A	lof	SPATS1	p.His112fs	FALSE	FALSE	FALSE	FALSE
pLOF	6	46607355	TA	T	lof	CYP39A1	p.Met122fs	FALSE	FALSE	FALSE	FALSE
pLOF	6	50011381	G	T	lof	DEFB112	p.C83X	FALSE	FALSE	FALSE	FALSE
pLOF	6	51586784	C	G	lof	PKHD1	p.X3397Y	FALSE	FALSE	FALSE	FALSE
pLOF	6	55192375	G	A	lof	GFRAL	.	FALSE	FALSE	FALSE	FALSE
pLOF	6	74492480	T	C	lof	CD109	.	FALSE	FALSE	FALSE	FALSE
dDNV/pLOF	6	85457637	C	T	lof	TBX18	NA	FALSE	FALSE	TRUE	TRUE
pLOF	6	87994477	CA	C	lof	GJB7	p.F51fs	FALSE	FALSE	FALSE	FALSE
pLOF	6	88315682	G	GC	lof	ORC3	p.A124fs	FALSE	TRUE	FALSE	FALSE
pLOF	6	90340298	C	T	lof	ANKRD6	p.Arg587*	FALSE	TRUE	FALSE	FALSE
dDNV	6	1.07E+08	G	A	misD	PRDM1	p.C689Y	FALSE	FALSE	FALSE	TRUE
pLOF	6	1.07E+08	CT	C	lof	AIM1	p.Leu1506fs	FALSE	FALSE	FALSE	FALSE

© 2023 Morton SU et al. *JAMA Network Open*.

dDNV	6	1.1E+08	G	A	misD	FIG4	p.G281D	FALSE	FALSE	FALSE	FALSE
pLOF	6	1.13E+08	CA	C	lof	RFPL4B	p.Q82fs	FALSE	FALSE	FALSE	FALSE
pLOF	6	1.28E+08	G	GA	lof	RSPO3	p.R220fs	FALSE	FALSE	FALSE	FALSE
pLOF	6	1.33E+08	C	A	lof	MOXD1	p.G436X	FALSE	FALSE	FALSE	FALSE
pLOF	6	1.33E+08	AG	A	lof	TAAR6	p.Q329fs	FALSE	FALSE	FALSE	FALSE
pLOF	6	1.49E+08	CG	C	lof	SASH1	p.P19fs	FALSE	TRUE	FALSE	FALSE
pLOF	6	1.5E+08	AC	A	lof	LRP11	.	FALSE	FALSE	FALSE	FALSE
pLOF	6	1.56E+08	TG	T	lof	TIAM2	p.Val1657fs	FALSE	FALSE	FALSE	FALSE
pLOF	6	1.6E+08	C	A	lof	PNLDC1	p.S92X	FALSE	FALSE	FALSE	FALSE
pLOF	6	1.64E+08	TG	T	lof	PACRG	p.G261fs	FALSE	FALSE	FALSE	FALSE
pLOF	7	2644541	CA	C	lof	IQCE	p.R554fs	FALSE	FALSE	FALSE	FALSE
pLOF	7	6057531	T	A	lof	AIMP2	p.C143X	FALSE	TRUE	FALSE	FALSE
pLOF	7	6509298	G	A	lof	KDELR2	p.R94X	FALSE	TRUE	FALSE	FALSE
pLOF	7	7222452	G	A	lof	C1GALT1	NA	FALSE	FALSE	FALSE	TRUE
pLOF	7	11514154	G	A	lof	THSD7A	p.R687X	FALSE	FALSE	FALSE	TRUE
pLOF	7	17865710	A	T	lof	SNX13	p.L547X	FALSE	FALSE	FALSE	FALSE
pLOF	7	20685726	G	T	lof	ABCB5	p.G316X	FALSE	FALSE	FALSE	FALSE
dDNV	7	23340587	G	A	misD	MALSU1	p.G129E	FALSE	FALSE	FALSE	FALSE
pLOF	7	24843989	C	A	lof	OSBPL3	p.E838X	FALSE	FALSE	FALSE	FALSE
dDNV	7	36194427	G	A	misD	EEPD1	p.R165H	FALSE	FALSE	FALSE	FALSE
pLOF	7	36589044	C	A	lof	AOAH	.	FALSE	FALSE	FALSE	FALSE
pLOF	7	37960615	G	A	lof	EPDR1	p.W25X	FALSE	FALSE	FALSE	FALSE
pLOF	7	39612204	TC	T	lof	YAE1D1	p.S194X	FALSE	FALSE	FALSE	FALSE
pLOF	7	43447331	G	A	lof	HECW1	NA	FALSE	FALSE	FALSE	TRUE
pLOF	7	44579274	GCAAC	G	lof	NPC1L1	p.Val240fs	FALSE	FALSE	FALSE	FALSE
pLOF	7	45009011	C	T	lof	MYO1G	.	FALSE	FALSE	FALSE	FALSE
pLOF	7	47840312	G	A	lof	PKD1L1	p.Q2710X	FALSE	FALSE	FALSE	FALSE

© 2023 Morton SU et al. *JAMA Network Open*.

pLOF	7	55886818	A	G	lof	14-Sep	.	FALSE	FALSE	FALSE	FALSE
pLOF	7	56082817	C	A	lof	PSPH	p.G157X	FALSE	FALSE	FALSE	FALSE
pLOF	7	63982537	G	GT	lof	ZNF680	p.Gln199fs	FALSE	FALSE	FALSE	FALSE
pLOF	7	76908378	G	T	lof	CCDC146	p.E552X	FALSE	FALSE	FALSE	FALSE
pLOF	7	86991162	AGGACT	A	lof	CROT	p.R209fs	FALSE	FALSE	FALSE	FALSE
pLOF	7	87180040	C	CAAG	lof	ABCB1	.	FALSE	FALSE	FALSE	FALSE
pLOF	7	99145077	GT	G	lof	FAM200A	p.N318fs	FALSE	FALSE	FALSE	FALSE
pLOF	7	99226935	CT	C	lof	ZSCAN25	p.C310fs	FALSE	FALSE	FALSE	FALSE
pLOF	7	99802722	TC	T	lof	STAG3	p.Arg1018fs	FALSE	FALSE	FALSE	FALSE
pLOF	7	99811411	TAC	T	lof	STAG3	p.T1218fs	FALSE	FALSE	FALSE	FALSE
pLOF	7	1E+08	C	A	lof	ZCWPW1	p.G467X	FALSE	FALSE	FALSE	FALSE
pLOF	7	1E+08	TCA	T	lof	SRRT	NA	FALSE	TRUE	FALSE	TRUE
pLOF	7	1.02E+08	C	T	lof	ALKBH4	p.W97X	FALSE	FALSE	FALSE	FALSE
pLOF	7	1.05E+08	T	A	lof	RINT1	p.Y55X	FALSE	FALSE	FALSE	FALSE
pLOF	7	1.05E+08	TC	T	lof	RINT1	p.P759fs	FALSE	FALSE	FALSE	FALSE
pLOF	7	1.06E+08	C	T	lof	CDHR3	p.R166X	FALSE	FALSE	FALSE	FALSE
pLOF	7	1.07E+08	C	CA	lof	SLC26A4	p.A51fs	FALSE	FALSE	FALSE	FALSE
pLOF	7	1.08E+08	C	A	lof	PNPLA8	p.E173X	FALSE	FALSE	FALSE	FALSE
pLOF	7	1.23E+08	ACACTT	A	lof	SLC13A1	p.E185fs	FALSE	FALSE	FALSE	FALSE
dDNV	7	1.27E+08	G	A	misD	PAX4	0	FALSE	FALSE	FALSE	FALSE
pLOF	7	1.28E+08	G	A	lof	FAM71F1	.	FALSE	FALSE	FALSE	FALSE
dDNV	7	1.29E+08	G	A	misD	IRF5	p.V78M	FALSE	FALSE	FALSE	FALSE
pLOF	7	1.34E+08	G	A	lof	LRGUK	.	FALSE	FALSE	FALSE	FALSE
pLOF	7	1.34E+08	A	G	lof	LRGUK	.	FALSE	FALSE	FALSE	FALSE
pLOF	7	1.38E+08	A	G	lof	SVOPL	.	FALSE	FALSE	FALSE	FALSE
pLOF	7	1.41E+08	G	A	lof	TAS2R3	p.W89X	FALSE	FALSE	FALSE	FALSE
pLOF	7	1.42E+08	G	C	lof	MGAM	.	FALSE	FALSE	FALSE	FALSE

© 2023 Morton SU et al. *JAMA Network Open*.

pLOF	7	1.42E+08	GC	G	lof	MGAM	p.Ile345fs	FALSE	FALSE	FALSE	FALSE
pLOF	7	1.43E+08	A	AG	lof	EPHA1	p.S334fs	FALSE	FALSE	FALSE	FALSE
pLOF	7	1.5E+08	G	A	lof	SSPO	p.W2685X	FALSE	FALSE	FALSE	FALSE
pLOF	7	1.51E+08	AC	A	lof	ABCB8	p.T494fs	FALSE	FALSE	FALSE	FALSE
pLOF	7	1.59E+08	CCCCATCCGAT	C	lof	VIPR2	p.Ile388fs	FALSE	FALSE	FALSE	FALSE
pLOF	8	2065710	CG	C	lof	MYOM2	p.T1141fs	FALSE	FALSE	FALSE	FALSE
dDNV/pLOF	8	11566422	C	T	lof	GATA4	p.Arg201*	FALSE	FALSE	FALSE	FALSE
pLOF	8	17739583	C	A	lof	FGL1	p.E57X	FALSE	FALSE	FALSE	FALSE
pLOF	8	20004768	C	G	lof	SLC18A1	.	FALSE	FALSE	FALSE	FALSE
pLOF	8	20028637	G	A	lof	SLC18A1	p.Q279X	FALSE	FALSE	FALSE	FALSE
pLOF	8	22006410	AAG	A	lof	LGI3	p.S303fs	FALSE	FALSE	FALSE	FALSE
pLOF	8	22398262	G	GT	lof	PPP3CC	p.A505fs	FALSE	FALSE	FALSE	FALSE
pLOF	8	27369388	C	A	lof	EPHX2	p.C232X	FALSE	FALSE	FALSE	FALSE
pLOF	8	33358006	ACT	A	lof	TTI2	p.R420fs	FALSE	FALSE	FALSE	FALSE
pLOF	8	33369537	C	A	lof	TTI2	p.E199X	FALSE	FALSE	FALSE	FALSE
pLOF	8	37699776	A	AG	lof	ADGRA2	p.K1307fs	FALSE	FALSE	FALSE	FALSE
pLOF	8	38008325	CGCTAGCA	C	lof	STAR	p.L2fs	FALSE	FALSE	FALSE	FALSE
pLOF	8	38848927	C	T	lof	TM2D2	p.Trp190*	FALSE	TRUE	FALSE	FALSE
pLOF	8	54764525	G	A	lof	RGS20	p.Trp22*	FALSE	FALSE	FALSE	FALSE
dDNV	8	54870931	G	A	misD	RGS20	p.M360I	FALSE	FALSE	FALSE	FALSE
pLOF	8	56910991	CAT	C	lof	LYN	p.M380fs	FALSE	FALSE	FALSE	TRUE
pLOF	8	66594687	G	A	lof	MTFR1	.	FALSE	TRUE	FALSE	FALSE
pLOF	8	66619392	G	GA	lof	MTFR1	p.G222fs	FALSE	TRUE	FALSE	FALSE
pLOF	8	75928931	C	T	lof	CRISPLD1	p.Q287X	FALSE	FALSE	FALSE	FALSE
dDNV	8	81577102	C	G	misD	ZNF704	p.S292T	FALSE	FALSE	FALSE	TRUE
dDNV	8	94797492	C	G	misD	TMEM67	p.P311A	FALSE	FALSE	FALSE	FALSE

© 2023 Morton SU et al. *JAMA Network Open*.

pLOF	8	95262551	AG	A	lof	GEM	p.L293fs	FALSE	FALSE	FALSE	FALSE
pLOF	8	96057773	GA	G	lof	NDUFAF6	p.E160fs	FALSE	FALSE	FALSE	FALSE
pLOF	8	97342501	C	T	lof	PTDSS1	p.R412X	FALSE	FALSE	TRUE	FALSE
pLOF	8	1.01E+08	CA	C	lof	VPS13B	p.Q1420fs	FALSE	FALSE	TRUE	FALSE
pLOF	8	1.05E+08	T	A	lof	RIMS2	.	FALSE	FALSE	FALSE	TRUE
dDNV/pLOF	8	1.08E+08	C	G	lof	OXR1	p.S822X	FALSE	FALSE	FALSE	TRUE
pLOF	8	1.21E+08	C	T	lof	DSCC1	NA	FALSE	FALSE	FALSE	FALSE
pLOF	8	1.21E+08	G	T	lof	COL14A1	p.E674X	FALSE	FALSE	FALSE	FALSE
pLOF	8	1.21E+08	C	T	lof	COL14A1	p.Q1776X	FALSE	FALSE	FALSE	FALSE
pLOF	8	1.25E+08	C	T	lof	FER1L6	p.Q1412X	FALSE	FALSE	FALSE	FALSE
dDNV	8	1.42E+08	C	A	misD	AGO2	p.G201V	FALSE	TRUE	FALSE	TRUE
pLOF	8	1.44E+08	G	GT	lof	GML	p.Cys48fs	FALSE	FALSE	FALSE	FALSE
pLOF	8	1.44E+08	C	CCA	lof	ZNF696	p.H167fs	FALSE	FALSE	FALSE	FALSE
pLOF	8	1.45E+08	AG	A	lof	MROH6	p.L422fs	FALSE	FALSE	FALSE	FALSE
pLOF	8	1.45E+08	CG	C	lof	MROH6	p.P234fs	FALSE	FALSE	FALSE	FALSE
pLOF	8	1.45E+08	C	CG	lof	EEF1D	p.Leu272fs	FALSE	TRUE	FALSE	FALSE
pLOF	8	1.45E+08	C	T	lof	TSTA3	p.W208X	FALSE	FALSE	FALSE	FALSE
dDNV/pLOF	8	1.45E+08	GCTTT	G	lof	ZNF623	p.A343fs	FALSE	FALSE	FALSE	FALSE
pLOF	8	1.45E+08	C	A	lof	CCDC166	p.X440L	FALSE	FALSE	FALSE	FALSE
pLOF	8	1.45E+08	CT	C	lof	EPPK1	p.E1281fs	FALSE	FALSE	FALSE	FALSE
pLOF	8	1.45E+08	G	GCT	lof	OPLAH	p.S1153fs	FALSE	FALSE	FALSE	FALSE
dDNV	8	1.45E+08	G	A	misD	EXOSC4	p.G37S	FALSE	TRUE	FALSE	FALSE
pLOF	8	1.46E+08	C	CA	lof	FBXL6	p.L39fs	FALSE	TRUE	FALSE	FALSE
pLOF	8	1.46E+08	G	C	lof	SLC39A4	p.Y329X	FALSE	FALSE	FALSE	FALSE
pLOF	9	4684970	C	T	lof	CDC37L1	p.Gln76*	FALSE	FALSE	FALSE	TRUE
pLOF	9	13138141	C	A	lof	MPDZ	p.E1339X	FALSE	TRUE	FALSE	FALSE

© 2023 Morton SU et al. *JAMA Network Open*.

pLOF	9	15433562	GT	G	lof	SNAPC3	p.F136fs	FALSE	TRUE	FALSE	FALSE
pLOF	9	15745534	GT	G	lof	CCDC171	p.R859fs	FALSE	FALSE	FALSE	FALSE
pLOF	9	17502606	A	T	lof	CNTLN	p.K1393X	FALSE	FALSE	FALSE	FALSE
dDNV	9	19049689	T	C	misD	RRAGA	p.L11P	FALSE	TRUE	FALSE	FALSE
pLOF	9	21007120	T	C	lof	HACD4	.	FALSE	FALSE	FALSE	FALSE
pLOF	9	32436396	G	A	lof	ACO1	.	FALSE	TRUE	FALSE	FALSE
dDNV	9	34372784	T	G	misD	KIAA1161	p.Asp19Ala	FALSE	FALSE	FALSE	FALSE
pLOF	9	34520656	GA	G	lof	DNAI1	p.E668fs	FALSE	FALSE	FALSE	FALSE
pLOF	9	35906601	C	CCA	lof	HRCT1	p.His107fs	FALSE	FALSE	FALSE	FALSE
pLOF	9	37729761	C	T	lof	FRMPD1	p.R217X	FALSE	FALSE	FALSE	FALSE
dDNV/pLOF	9	71833276	G	T	lof	TJP2	NA	FALSE	TRUE	FALSE	FALSE
pLOF	9	72929753	G	T	lof	SMC5	.	FALSE	TRUE	FALSE	FALSE
pLOF	9	75315444	G	T	lof	TMC1	p.E83X	FALSE	FALSE	FALSE	FALSE
pLOF	9	79321843	C	A	lof	PRUNE2	p.Glu1783*	FALSE	FALSE	FALSE	FALSE
dDNV	9	85926815	G	A	misD	FRMD3	p.H254Y	FALSE	FALSE	FALSE	FALSE
pLOF	9	99227618	G	A	lof	HABP4	.	FALSE	FALSE	FALSE	FALSE
pLOF	9	99525916	T	C	lof	ZNF510	.	FALSE	FALSE	FALSE	FALSE
pLOF	9	99799532	A	G	lof	CTSV	NA	FALSE	TRUE	FALSE	FALSE
pLOF	9	1.07E+08	A	AC	lof	OR13C3	p.V316fs	FALSE	FALSE	FALSE	FALSE
pLOF	9	1.08E+08	ACGGG	A	lof	NIPSNAP3B	p.T27fs	FALSE	FALSE	FALSE	FALSE
pLOF	9	1.08E+08	C	T	lof	ABCA1	.	FALSE	FALSE	FALSE	FALSE
dDNV	9	1.08E+08	G	A	misD	SLC44A1	p.D63N	FALSE	FALSE	FALSE	TRUE
pLOF	9	1.15E+08	G	T	lof	HSDL2	p.E185X	FALSE	FALSE	FALSE	FALSE
pLOF	9	1.16E+08	G	A	lof	SLC46A2	p.R392X	FALSE	FALSE	FALSE	FALSE
pLOF	9	1.16E+08	TATATG	T	lof	SLC46A2	p.I291fs	FALSE	FALSE	FALSE	FALSE
pLOF	9	1.3E+08	T	A	lof	ZNF79	.	FALSE	FALSE	FALSE	FALSE

© 2023 Morton SU et al. *JAMA Network Open*.

pLOF	9	1.3E+08	CAT	C	lof	TOR2A	p.Y101fs	FALSE	FALSE	FALSE	FALSE
pLOF	9	1.31E+08	CAGGACTTCCTG	C	lof	PKN3	p.Q439fs	FALSE	FALSE	FALSE	FALSE
pLOF	9	1.32E+08	A	G	lof	TBC1D13	p.Met1?	FALSE	TRUE	FALSE	FALSE
pLOF	9	1.36E+08	CCTGCCCCCCGAAGA	C	lof	GBGT1	p.V264fs	FALSE	FALSE	FALSE	FALSE
pLOF	9	1.36E+08	G	A	lof	MED22	p.Q47X	FALSE	TRUE	FALSE	FALSE
pLOF	9	1.39E+08	G	T	lof	NOTCH1	p.C1521X	FALSE	TRUE	FALSE	TRUE
pLOF	9	1.39E+08	GT	G	lof	NOTCH1	p.Tyr899fs	FALSE	TRUE	FALSE	TRUE
pLOF	9	1.4E+08	G	T	lof	FAM69B	.	FALSE	FALSE	FALSE	FALSE
pLOF	9	1.4E+08	T	TG	lof	C9orf142	p.Asp2fs	FALSE	FALSE	FALSE	FALSE
pLOF	9	1.4E+08	C	T	lof	ENTPD2	.	FALSE	FALSE	FALSE	FALSE
pLOF	9	1.4E+08	C	T	lof	ENTPD2	.	FALSE	FALSE	FALSE	FALSE
pLOF	9	1.4E+08	C	CG	lof	DPP7	p.R143fs	FALSE	FALSE	FALSE	FALSE
pLOF	9	1.4E+08	G	A	lof	TPRN	p.Q586X	FALSE	FALSE	FALSE	FALSE
pLOF	9	1.4E+08	C	T	lof	EXD3	.	FALSE	FALSE	FALSE	FALSE
pLOF	9	1.4E+08	G	GT	lof	NOXA1	p.D440fs	FALSE	FALSE	FALSE	FALSE
pLOF	10	1094841	GA	G	lof	IDI1	p.H34fs	FALSE	TRUE	FALSE	FALSE
pLOF	10	5765639	G	A	lof	FAM208B	.	FALSE	FALSE	FALSE	FALSE
pLOF	10	17432555	C	A	lof	ST8SIA6	p.E89X	FALSE	FALSE	FALSE	FALSE
pLOF	10	23295906	T	TA	lof	ARMC3	p.Y609_V610delinsX	FALSE	FALSE	FALSE	FALSE
pLOF	10	26241208	GTAAGTCA	G	lof	MYO3A	.	FALSE	FALSE	FALSE	FALSE
dDNV/pLOF	10	27066140	G	A	lof	ABI1	p.R123X	FALSE	TRUE	FALSE	TRUE
pLOF	10	28413937	AC	A	lof	MPP7	.	FALSE	FALSE	FALSE	FALSE
pLOF	10	45486395	G	A	lof	RASSF4	NA	FALSE	FALSE	FALSE	FALSE

© 2023 Morton SU et al. *JAMA Network Open*.

pLOF	10	50025435	T	TC	lof	WDFY4	p.F1829fs	FALSE	FALSE	TRUE	FALSE
pLOF	10	50950998	C	CAA	lof	OGDHL	p.A630fs	FALSE	FALSE	FALSE	FALSE
pLOF	10	70105527	A	T	lof	RUFY2	p.X642R	FALSE	TRUE	FALSE	FALSE
dDNV	10	73047432	C	T	misD	UNC5B	p.R271W	FALSE	FALSE	FALSE	FALSE
pLOF	10	74879790	TGAAG	T	lof	NUDT13	p.L33fs	FALSE	FALSE	FALSE	FALSE
pLOF	10	74994978	C	T	lof	FAM149B1	p.Q502X	FALSE	TRUE	FALSE	FALSE
pLOF	10	82187174	G	A	lof	FAM213A	p.W166X	FALSE	TRUE	FALSE	FALSE
pLOF	10	82330050	C	T	lof	SH2D4B	p.Q109X	FALSE	FALSE	FALSE	FALSE
pLOF	10	85992201	G	A	lof	LRIT1	p.Q452X	FALSE	FALSE	FALSE	FALSE
pLOF	10	90574328	AC	A	lof	LIPM	p.N169fs	FALSE	FALSE	FALSE	FALSE
pLOF	10	92680094	C	CT	lof	ANKRD1	p.K13fs	FALSE	FALSE	FALSE	FALSE
pLOF	10	96066442	C	T	lof	PLCE1	p.R1961X	FALSE	FALSE	FALSE	FALSE
pLOF	10	98087253	CTG	C	lof	DNTT	p.Val303fs	FALSE	FALSE	FALSE	FALSE
pLOF	10	99969478	CAG	C	lof	R3HCC1L	p.S536fs	FALSE	FALSE	FALSE	FALSE
pLOF	10	1E+08	AG	A	lof	PYROXD2	p.L446fs	FALSE	FALSE	FALSE	FALSE
pLOF	10	1.02E+08	G	GC	lof	SEC31B	p.R247fs	FALSE	FALSE	FALSE	FALSE
pLOF	10	1.04E+08	GC	G	lof	PPRC1	p.L155fs	FALSE	TRUE	FALSE	TRUE
pLOF	10	1.04E+08	GC	G	lof	C10orf95	p.Q242fs	FALSE	FALSE	FALSE	FALSE
pLOF	10	1.06E+08	CCTGTTTT	C	lof	OBFC1	p.I54fs	FALSE	FALSE	FALSE	FALSE
pLOF	10	1.16E+08	C	A	lof	PLEKHS1	p.Y113X	FALSE	FALSE	FALSE	FALSE
pLOF	10	1.18E+08	GTCGGCGT	G	lof	PNLIP	p.S154fs	FALSE	FALSE	FALSE	FALSE
pLOF	10	1.26E+08	CG	C	lof	CPXM2	p.P263fs	FALSE	FALSE	FALSE	FALSE
pLOF	10	1.26E+08	TG	T	lof	LHPP	p.Leu142fs	FALSE	FALSE	FALSE	FALSE
pLOF	10	1.35E+08	G	A	lof	ECHS1	p.Q162X	FALSE	FALSE	FALSE	FALSE
pLOF	10	1.35E+08	G	T	lof	MTG1	.	FALSE	FALSE	FALSE	FALSE
dDNV/pLOF	11	609337	GCA	G	lof	PHRF1	p.S1293fs	FALSE	TRUE	TRUE	FALSE

© 2023 Morton SU et al. *JAMA Network Open*.

pLOF	11	613848	CTG	C	lof	IRF7	p.Pro274fs	FALSE	FALSE	FALSE	FALSE
pLOF	11	798081	AC	A	lof	PANO1	p.P153fs	FALSE	FALSE	FALSE	FALSE
pLOF	11	1078667	G	A	lof	MUC2	NA	FALSE	FALSE	FALSE	FALSE
pLOF	11	2909674	CT	C	lof	SLC22A18AS	p.K166fs	FALSE	FALSE	FALSE	FALSE
pLOF	11	3114102	G	C	lof	OSBPL5	p.Y709X	FALSE	FALSE	FALSE	FALSE
pLOF	11	3661226	G	A	lof	ART5	p.R145X	FALSE	FALSE	FALSE	FALSE
pLOF	11	4566632	TTGAC	T	lof	OR52M1	p.I71fs	FALSE	FALSE	TRUE	FALSE
pLOF	11	5529357	G	GC	lof	UBQLN3	p.P478fs	FALSE	FALSE	FALSE	FALSE
pLOF	11	5624690	C	T	lof	TRIM6,TRIM6-TRIM34	p.Q50X	FALSE	FALSE	FALSE	FALSE
pLOF	11	5701123	A	T	lof	TRIM5	p.C95X	FALSE	FALSE	FALSE	FALSE
pLOF	11	5969442	C	CA	lof	OR56A3	p.A289fs	FALSE	FALSE	FALSE	FALSE
pLOF	11	7091688	G	A	lof	NLRP14	.	FALSE	FALSE	FALSE	FALSE
pLOF	11	7726182	C	T	lof	OVCH2	p.W43X	FALSE	FALSE	FALSE	FALSE
pLOF	11	12495531	G	A	lof	PARVA	.	FALSE	TRUE	FALSE	FALSE
pLOF	11	17428606	C	T	lof	ABCC8	p.W998X	FALSE	FALSE	FALSE	FALSE
pLOF	11	18425273	GTC	G	lof	LDHA	p.V209fs	FALSE	TRUE	FALSE	FALSE
pLOF	11	18745690	TTGTC	T	lof	IGSF22	p.Thr31fs	FALSE	FALSE	FALSE	FALSE
pLOF	11	19186675	G	A	lof	ZDHHC13	p.Trp458*	FALSE	FALSE	FALSE	FALSE
pLOF	11	19197500	C	A	lof	ZDHHC13	p.S621X	FALSE	FALSE	FALSE	FALSE
pLOF	11	34218927	C	A	lof	ABTB2	p.E397X	FALSE	FALSE	FALSE	FALSE
pLOF	11	34905048	A	C	lof	APIP	p.Y155X	FALSE	FALSE	FALSE	FALSE
pLOF	11	44626642	T	A	lof	CD82	p.Y57X	FALSE	FALSE	FALSE	FALSE
pLOF	11	46385929	G	A	lof	DGKZ	NA	FALSE	TRUE	FALSE	FALSE
pLOF	11	48238802	G	A	lof	OR4B1	p.W147X	FALSE	FALSE	FALSE	FALSE
pLOF	11	59947354	CT	C	lof	MS4A6A	p.Val106fs	FALSE	FALSE	FALSE	FALSE

© 2023 Morton SU et al. *JAMA Network Open*.

pLOF	11	61041980	A	AC	lof	VWCE	p.C524fs	FALSE	FALSE	FALSE	FALSE
dDNV/pLOF	11	63065135	C	T	lof	SLC22A10	p.R256X	FALSE	FALSE	FALSE	FALSE
pLOF	11	64787900	G	T	lof	ARL2	p.G117X	FALSE	TRUE	FALSE	FALSE
pLOF	11	65546682	C	A	lof	AP5B1	p.E428X	FALSE	FALSE	FALSE	FALSE
pLOF	11	66061941	A	AGGCC	lof	TMEM151A	p.E75fs	FALSE	FALSE	FALSE	FALSE
pLOF	11	66101688	GA	G	lof	RIN1	p.V431fs	FALSE	FALSE	FALSE	FALSE
pLOF	11	66358747	G	GT	lof	CCDC87	p.N580fs	FALSE	FALSE	FALSE	FALSE
pLOF	11	66742862	T	A	lof	C11orf86	p.L10X	FALSE	FALSE	FALSE	FALSE
pLOF	11	67412557	A	AG	lof	ACY3	p.Leu198fs	FALSE	FALSE	FALSE	FALSE
pLOF	11	68204467	G	T	lof	LRP5	p.E1371X	FALSE	TRUE	FALSE	TRUE
pLOF	11	68483305	C	CT	lof	MTL5	p.K340fs	FALSE	FALSE	FALSE	FALSE
dDNV/pLOF	11	68839405	CT	C	lof	TPCN2	p.S326fs	FALSE	FALSE	FALSE	FALSE
pLOF	11	72465786	A	ATT	lof	STARD10	p.Asp138fs	FALSE	FALSE	FALSE	FALSE
pLOF	11	73834032	C	T	lof	C2CD3	.	FALSE	TRUE	FALSE	FALSE
pLOF	11	74988484	G	T	lof	ARRB1	p.Tyr209*	TRUE	TRUE	FALSE	TRUE
pLOF	11	85367363	GCT	G	lof	TMEM126A	p.Leu137fs	FALSE	TRUE	FALSE	FALSE
pLOF	11	87908538	G	A	lof	RAB38	p.Gln22*	FALSE	FALSE	FALSE	FALSE
pLOF	11	88911360	G	A	lof	TYR	p.W80X	FALSE	FALSE	FALSE	FALSE
pLOF	11	93428801	G	A	lof	KIAA1731	NA	FALSE	TRUE	FALSE	FALSE
pLOF	11	93886656	G	A	lof	PANX1	.	FALSE	TRUE	FALSE	FALSE
pLOF	11	94823392	G	C	lof	ENDOD1	NA	FALSE	FALSE	FALSE	FALSE
pLOF	11	94862041	CA	C	lof	ENDOD1	p.N268fs	FALSE	FALSE	FALSE	FALSE
pLOF	11	1.02E+08	C	A	lof	TMEM123	p.E65X	FALSE	TRUE	FALSE	TRUE
pLOF	11	1.03E+08	G	A	lof	DYNC2H1	p.Trp3297*	FALSE	FALSE	FALSE	FALSE
pLOF	11	1.07E+08	CAA	C	lof	ALKBH8	p.L52fs	FALSE	FALSE	FALSE	FALSE

© 2023 Morton SU et al. *JAMA Network Open*.

dDNV/pLOF	11	1.08E+08	T	TA	lof	ATM	p.Tyr731fs	TRUE	FALSE	FALSE	FALSE
pLOF	11	1.08E+08	T	TA	lof	ATM	p.Tyr731fs	TRUE	FALSE	FALSE	FALSE
pLOF	11	1.08E+08	T	C	lof	EXPH5	.	FALSE	FALSE	FALSE	FALSE
pLOF	11	1.1E+08	C	T	lof	ZC3H12C	p.Arg161*	FALSE	FALSE	FALSE	TRUE
pLOF	11	1.14E+08	GC	G	lof	TMPRSS5	p.G339fs	FALSE	FALSE	FALSE	FALSE
pLOF	11	1.14E+08	A	G	lof	REXO2	NA	FALSE	FALSE	FALSE	FALSE
dDNV/pLOF	11	1.21E+08	T	TCGACG	lof	TECTA	p.F1498fs	FALSE	FALSE	FALSE	FALSE
pLOF	11	1.21E+08	A	T	lof	TECTA	.	FALSE	FALSE	FALSE	FALSE
pLOF	11	1.23E+08	T	A	lof	C11orf63	.	FALSE	FALSE	FALSE	FALSE
pLOF	11	1.23E+08	G	A	lof	CLMP	p.R274X	FALSE	FALSE	FALSE	FALSE
pLOF	11	1.23E+08	G	A	lof	GRAMD1B	.	FALSE	FALSE	FALSE	TRUE
pLOF	11	1.24E+08	GC	G	lof	TMEM225	p.S202fs	FALSE	FALSE	FALSE	FALSE
pLOF	11	1.25E+08	CA	C	lof	SIAE	p.F164fs	FALSE	FALSE	FALSE	FALSE
pLOF	11	1.26E+08	CCTCTGCTGAA	C	lof	CDON	p.Phe1208fs	FALSE	TRUE	TRUE	FALSE
pLOF	11	1.26E+08	GC	G	lof	CDON	p.Ser441fs	FALSE	TRUE	TRUE	FALSE
dDNV/pLOF	11	1.26E+08	G	A	lof	RPUSD4	p.R272X	FALSE	FALSE	FALSE	FALSE
pLOF	11	1.26E+08	ATT	A	lof	RPUSD4	p.K230fs	FALSE	FALSE	FALSE	FALSE
pLOF	11	1.26E+08	C	T	lof	DCPS	p.Q332X	FALSE	TRUE	FALSE	FALSE
pLOF	11	1.3E+08	G	A	lof	TMEM45B	.	FALSE	FALSE	FALSE	FALSE
dDNV/pLOF	11	1.32E+08	CA	C	lof	NTM	p.N205fs	FALSE	FALSE	FALSE	FALSE
dDNV/pLOF	12	2973909	G	A	lof	FOXM1	p.R344X	FALSE	TRUE	FALSE	FALSE
pLOF	12	4554720	TTC	T	lof	FGF6	p.Q5fs	FALSE	FALSE	FALSE	FALSE
pLOF	12	4634436	TG	T	lof	C12orf4	p.P237fs	FALSE	FALSE	FALSE	FALSE
pLOF	12	4652929	A	AT	lof	RAD51AP1	p.Asp24fs	FALSE	FALSE	FALSE	FALSE

© 2023 Morton SU et al. *JAMA Network Open*.

dDNV/pLOF	12	7244261	G	GAGGT	lof	C1R	p.Leu5fs	FALSE	FALSE	FALSE	TRUE
pLOF	12	7480967	T	C	lof	ACSM4	p.X581Q	FALSE	FALSE	FALSE	FALSE
dDNV	12	9355165	A	T	misD	PZP	p.V128D	FALSE	FALSE	FALSE	FALSE
dDNV/pLOF	12	9747903	TGTTA	T	lof	KLRB1	0	FALSE	FALSE	FALSE	FALSE
pLOF	12	9875299	G	A	lof	CLECL1	p.Gln143*	FALSE	FALSE	FALSE	FALSE
pLOF	12	10145887	C	T	lof	CLEC1B	.	FALSE	FALSE	FALSE	FALSE
dDNV/pLOF	12	18836192	T	TAAAC	lof	PLCZ1	p.Y603fs	FALSE	FALSE	FALSE	FALSE
pLOF	12	21051414	C	G	lof	LST3	p.Ser576*	FALSE	FALSE	FALSE	FALSE
pLOF	12	21445271	C	G	lof	SLCO1A2	.	FALSE	FALSE	FALSE	FALSE
pLOF	12	22214388	T	G	lof	CMAS	.	FALSE	TRUE	FALSE	TRUE
pLOF	12	25257341	C	T	lof	LRMP	p.R365X	FALSE	FALSE	FALSE	FALSE
pLOF	12	27135750	AGCAGG	A	lof	TM7SF3	p.A302fs	FALSE	FALSE	FALSE	FALSE
dDNV	12	27470296	T	G	misD	STK38L	p.Y289D	FALSE	FALSE	FALSE	TRUE
pLOF	12	32134323	A	ACGTACCCAATATGC	lof	KIAA1551	p.N145fs	FALSE	TRUE	FALSE	FALSE
pLOF	12	40034773	A	T	lof	C12orf40	p.K14X	FALSE	FALSE	FALSE	FALSE
pLOF	12	40742210	G	C	lof	LRRK2	.	FALSE	FALSE	FALSE	FALSE
pLOF	12	40835700	G	GTACC	lof	MUC19	p.Gln685fs	FALSE	FALSE	FALSE	FALSE
pLOF	12	49170134	C	A	lof	ADCY6	.	FALSE	TRUE	FALSE	FALSE
dDNV	12	49663619	C	T	misD	TUBA1C	p.R44C	FALSE	FALSE	FALSE	FALSE
pLOF	12	50727773	T	TG	lof	FAM186A	p.Q2206fs	FALSE	FALSE	FALSE	FALSE
pLOF	12	50835386	TAAGAG	T	lof	LARP4	p.L255fs	FALSE	FALSE	FALSE	TRUE
pLOF	12	51279028	G	T	lof	TMPRSS12	.	FALSE	FALSE	FALSE	FALSE
pLOF	12	53456906	CAG	C	lof	TNS2	p.T1162fs	FALSE	FALSE	FALSE	FALSE
pLOF	12	53701431	TAAAACGTGGA	T	lof	AAAS	p.F491fs	FALSE	TRUE	FALSE	FALSE

© 2023 Morton SU et al. *JAMA Network Open*.

pLOF	12	55886553	TG	T	lof	OR6C68	p.M131fs	FALSE	FALSE	FALSE	FALSE
pLOF	12	57638387	G	A	lof	STAC3	p.Q247X	FALSE	FALSE	FALSE	FALSE
pLOF	12	57892220	AGTGGAACACC	A	lof	MARS	p.Q302fs	FALSE	TRUE	FALSE	FALSE
pLOF	12	57910957	AT	A	lof	DDIT3	p.E48fs	FALSE	FALSE	FALSE	TRUE
pLOF	12	58204710	C	T	lof	AVIL	.	FALSE	FALSE	FALSE	FALSE
pLOF	12	64509674	G	A	lof	SRGAP1	NA	FALSE	FALSE	FALSE	TRUE
pLOF	12	66563708	G	T	lof	TMBIM4	p.Y8X	FALSE	FALSE	FALSE	FALSE
pLOF	12	80200107	GGAGC	G	lof	PPP1R12A	p.C553fs	FALSE	TRUE	FALSE	TRUE
pLOF	12	96260916	GC	G	lof	CCDC38	p.Gln543fs	FALSE	FALSE	FALSE	FALSE
pLOF	12	96370453	CT	C	lof	HAL	p.Q562fs	FALSE	FALSE	FALSE	FALSE
pLOF	12	1.02E+08	AC	A	lof	SLC5A8	p.V197fs	FALSE	FALSE	FALSE	FALSE
pLOF	12	1.06E+08	CT	C	lof	APPL2	p.K613fs	FALSE	FALSE	FALSE	FALSE
pLOF	12	1.07E+08	C	T	lof	TCP11L2	p.R500X	FALSE	FALSE	FALSE	FALSE
pLOF	12	1.07E+08	G	A	lof	POLR3B	.	FALSE	FALSE	FALSE	FALSE
pLOF	12	1.07E+08	A	G	lof	CRY1	.	FALSE	FALSE	FALSE	FALSE
pLOF	12	1.1E+08	CA	C	lof	UBE3B	p.T872fs	FALSE	TRUE	FALSE	FALSE
pLOF	12	1.14E+08	G	A	lof	RASAL1	p.R472X	FALSE	FALSE	FALSE	FALSE
pLOF	12	1.15E+08	C	A	lof	TBX5	.	FALSE	FALSE	FALSE	TRUE
pLOF	12	1.17E+08	TC	T	lof	FBXW8	p.P342fs	FALSE	TRUE	FALSE	FALSE
dDNV	12	1.18E+08	G	A	misD	KSR2	p.R689W	FALSE	FALSE	FALSE	TRUE
pLOF	12	1.21E+08	G	GA	lof	UNC119B	p.K27fs	FALSE	TRUE	FALSE	TRUE
dDNV/pLOF	12	1.33E+08	G	A	lof	POLE	p.R1371X	FALSE	TRUE	FALSE	FALSE
pLOF	13	28008941	G	A	lof	GTF3A	.	FALSE	TRUE	FALSE	FALSE
pLOF	13	32937315	G	C	lof	BRCA2	.	TRUE	FALSE	FALSE	FALSE
pLOF	13	36900732	ACT	A	lof	SPG20	p.K422fs	FALSE	TRUE	FALSE	FALSE
pLOF	13	41947910	CT	C	lof	NAA16	p.L765fs	FALSE	FALSE	FALSE	FALSE

© 2023 Morton SU et al. *JAMA Network Open*.

pLOF	13	46627751	A	T	lof	CPB2	p.X424K	FALSE	FALSE	FALSE	FALSE
pLOF	13	46824577	C	CTTAGAATT	lof	LRRC63	p.Phe396fs	FALSE	FALSE	FALSE	FALSE
pLOF	13	52249371	AC	A	lof	WDFY2	p.T91fs	FALSE	FALSE	FALSE	FALSE
pLOF	13	52523865	GT	G	lof	ATP7B	p.T822fs	FALSE	FALSE	FALSE	FALSE
pLOF	13	52684702	C	T	lof	NEK5	p.W108X	FALSE	FALSE	FALSE	FALSE
pLOF	13	73401890	CTG	C	lof	PIBF1	p.Val313fs	FALSE	FALSE	FALSE	FALSE
dDNV/pLOF	13	76123966	C	T	lof	UCHL3	p.Q4X	FALSE	TRUE	FALSE	FALSE
pLOF	13	76195885	ATG	A	lof	LMO7	p.D19fs	FALSE	FALSE	FALSE	FALSE
pLOF	13	1.03E+08	A	AG	lof	CCDC168	p.F7015fs	FALSE	FALSE	FALSE	FALSE
pLOF	13	1.14E+08	CA	C	lof	MCF2L	p.Ala654fs	FALSE	FALSE	FALSE	FALSE
dDNV	13	1.14E+08	C	T	misD	TFDP1	p.P389L	FALSE	TRUE	FALSE	TRUE
pLOF	14	23863399	T	A	lof	MYH6	p.Lys855*	FALSE	FALSE	FALSE	FALSE
dDNV	14	23874859	G	A	misD	MYH6	p.R108C	FALSE	FALSE	FALSE	FALSE
pLOF	14	24683589	CT	C	lof	MDP1,NEDD8-MDP1	p.K127fs	FALSE	FALSE	FALSE	FALSE
pLOF	14	24780124	AGGCCTGGCCGCTGGGCCAGGCG	A	lof	LTB4R2	p.Q85fs	FALSE	FALSE	FALSE	FALSE
pLOF	14	24877074	G	A	lof	NYNRIN	.	FALSE	TRUE	FALSE	FALSE
pLOF	14	31771509	C	T	lof	HEATR5A	p.W1819X	FALSE	FALSE	FALSE	FALSE
pLOF	14	31814331	A	T	lof	HEATR5A	p.L1007X	FALSE	FALSE	FALSE	FALSE
pLOF	14	50801301	A	AT	lof	CDKL1	p.P260fs	FALSE	FALSE	FALSE	FALSE
pLOF	14	55168778	A	G	lof	SAMD4A	.	FALSE	FALSE	FALSE	TRUE
pLOF	14	59789695	C	T	lof	DAAM1	p.R176X	FALSE	TRUE	FALSE	TRUE
pLOF	14	59950733	CTG	C	lof	L3HYPDH	p.Tyr100fs	FALSE	FALSE	FALSE	FALSE

© 2023 Morton SU et al. *JAMA Network Open*.

pLOF	14	69994619	C	T	lof	PLEKHD1	p.R441X	FALSE	FALSE	FALSE	FALSE
pLOF	14	76249674	TC	T	lof	TTLL5	p.H930fs	FALSE	FALSE	FALSE	FALSE
pLOF	14	77872312	CT	C	lof	NOXRED1	p.K283fs	FALSE	FALSE	FALSE	FALSE
pLOF	14	88737118	TA	T	lof	KCNK10	p.F5fs	FALSE	FALSE	FALSE	FALSE
dDNV	14	90738657	G	A	misD	PSMC1	p.R410H	FALSE	TRUE	FALSE	TRUE
pLOF	14	92537354	C	CTGCTGCTGCTGCTGCTG	lof	ATXN3	p.Gly315fs	FALSE	FALSE	FALSE	FALSE
pLOF	14	92537354	C	CTGCTGCTGCTGCTGCTGCTG	lof	ATXN3	p.Gly315fs	FALSE	FALSE	FALSE	FALSE
dDNV	14	1.01E+08	T	G	misD	WARS	p.T124P	FALSE	TRUE	FALSE	FALSE
pLOF	14	1.04E+08	A	AT	lof	TDRD9	p.N634fs	FALSE	FALSE	FALSE	FALSE
dDNV	14	1.06E+08	G	A	misD	JAG2	p.P542L	FALSE	FALSE	FALSE	TRUE
pLOF	15	28211835	C	T	lof	OCA2	.	FALSE	FALSE	FALSE	FALSE
pLOF	15	40502360	C	G	lof	BUB1B	p.Y778X	FALSE	TRUE	FALSE	FALSE
pLOF	15	40864065	C	CT	lof	RPUSD2	p.S290fs	FALSE	FALSE	FALSE	FALSE
pLOF	15	42980923	C	T	lof	STARD9	p.Gln2383*	FALSE	FALSE	FALSE	FALSE
pLOF	15	43452954	CT	C	lof	TMEM62	p.L420fs	FALSE	FALSE	FALSE	FALSE
pLOF	15	44067918	ACCT	A	lof	ELL3	p.E161fs	FALSE	FALSE	FALSE	FALSE
pLOF	15	44892685	CTTGA	C	lof	SPG11	p.I1221fs	FALSE	FALSE	FALSE	FALSE
pLOF	15	45965862	A	T	lof	SQRDL	p.K173X	FALSE	FALSE	FALSE	FALSE
pLOF	15	51633962	GGC	G	lof	GLDN	p.Leu29fs	FALSE	FALSE	FALSE	FALSE
pLOF	15	52186011	G	C	lof	TMOD3	.	FALSE	TRUE	FALSE	FALSE
pLOF	15	52574978	C	A	lof	MYO5C	.	FALSE	FALSE	FALSE	FALSE
dDNV	15	52689492	C	T	misD	MYO5A	p.A409T	FALSE	TRUE	TRUE	TRUE
pLOF	15	56243788	C	A	lof	NEDD4	.	FALSE	TRUE	FALSE	FALSE

© 2023 Morton SU et al. *JAMA Network Open*.

dDNV /pLOF	15	65903443	C	T	lof	VWA9	p.Trp9*	FALSE	TRUE	FALSE	FALSE
pLOF	15	66845592	TCTTC	T	lof	LCTL	p.G308fs	FALSE	FALSE	FALSE	FALSE
pLOF	15	67652194	A	T	lof	IQCH	p.R242X	FALSE	FALSE	FALSE	FALSE
pLOF	15	67813588	T	C	lof	C15orf61	p.Met1?	FALSE	FALSE	FALSE	FALSE
pLOF	15	69682073	GC	G	lof	PAQR5	p.A156fs	FALSE	FALSE	FALSE	FALSE
pLOF	15	69745350	GAC	G	lof	RPLP1	p.T22fs	FALSE	TRUE	FALSE	FALSE
pLOF	15	74743142	C	T	lof	UBL7	NA	FALSE	TRUE	FALSE	FALSE
pLOF	15	78882254	TCA	T	lof	CHRNA5	p.V174fs	FALSE	FALSE	FALSE	FALSE
pLOF	15	82563942	A	C	lof	SAXO2	.	FALSE	FALSE	FALSE	FALSE
pLOF	15	90294294	G	GCA	lof	MESP1	p.R57fs	FALSE	FALSE	FALSE	FALSE
pLOF	15	90431856	CCAAT	C	lof	AP3S2,C15orf38-AP3S2	p.I257fs	FALSE	FALSE	FALSE	FALSE
pLOF	15	90809010	C	CGCGACCCG	lof	NGRN	p.Gly26fs	FALSE	FALSE	FALSE	FALSE
pLOF	15	91304231	TAGAA	T	lof	BLM	p.L543fs	FALSE	TRUE	FALSE	FALSE
pLOF	15	91358377	CA	C	lof	BLM	p.Ser1375fs	FALSE	TRUE	FALSE	FALSE
pLOF	15	91434410	C	T	lof	FES	p.Q507X	FALSE	FALSE	FALSE	FALSE
pLOF	15	91434410	C	T	lof	FES	p.Q507X	FALSE	FALSE	FALSE	FALSE
pLOF	15	91542943	C	CA	lof	VPS33B	p.Glu580fs	FALSE	FALSE	FALSE	FALSE
pLOF	15	91795123	C	T	lof	SV2B	p.R176X	FALSE	FALSE	FALSE	FALSE
pLOF	15	91811837	T	G	lof	SV2B	.	FALSE	FALSE	FALSE	FALSE
dDNV	15	93173457	C	T	misD	FAM174B	p.D155N	FALSE	FALSE	FALSE	FALSE
pLOF	15	93198824	G	GGGAGCGGCCA	lof	FAM174B	p.Ala23fs	FALSE	FALSE	FALSE	FALSE
pLOF	15	94942216	T	G	lof	MCTP2	p.Y605X	FALSE	FALSE	FALSE	FALSE
pLOF	15	99251313	G	A	lof	IGF1R	p.W206X	FALSE	TRUE	FALSE	TRUE

© 2023 Morton SU et al. *JAMA Network Open*.

pLOF	15	1.02E+08	C	T	lof	LRRK1	p.Q471X	FALSE	FALSE	FALSE	FALSE
pLOF	16	321537	A	G	lof	RGS11	NA	FALSE	FALSE	FALSE	FALSE
pLOF	16	708923	C	T	lof	WDR90	p.Q975X	FALSE	TRUE	FALSE	FALSE
pLOF	16	732362	A	T	lof	STUB1	.	FALSE	TRUE	FALSE	FALSE
dDNV/pLOF	16	2139950	G	GT	lof	PKD1	p.N4230fs	FALSE	FALSE	FALSE	TRUE
pLOF	16	2809175	T	TA	lof	SRRM2	.	FALSE	TRUE	FALSE	TRUE
pLOF	16	3274302	G	A	lof	ZNF200	p.Q260X	FALSE	FALSE	FALSE	FALSE
pLOF	16	4852437	C	T	lof	ROGDI	p.W21X	FALSE	FALSE	FALSE	FALSE
pLOF	16	4934846	G	C	lof	PPL	p.Y1270X	FALSE	FALSE	FALSE	FALSE
dDNV/pLOF	16	4935373	G	A	lof	PPL	p.Q1095X	FALSE	FALSE	FALSE	FALSE
pLOF	16	10788361	G	A	lof	TEKT5	p.Q124X	FALSE	FALSE	FALSE	FALSE
pLOF	16	13002408	G	A	lof	SHISA9	p.W212X	FALSE	FALSE	FALSE	TRUE
pLOF	16	21983384	A	AGCCC	lof	UQCRC2	p.S303fs	FALSE	TRUE	FALSE	FALSE
pLOF	16	21985269	C	CT	lof	UQCRC2	p.T340fs	FALSE	TRUE	FALSE	FALSE
pLOF	16	30770379	CG	C	lof	C16orf93	p.Pro322fs	FALSE	FALSE	FALSE	FALSE
pLOF	16	31092069	G	GGGGGGACTGGGGAATCATAA	lof	ZNF646	p.S1475fs	FALSE	TRUE	FALSE	FALSE
pLOF	16	31121535	C	T	lof	BCKDK	p.Q145X	FALSE	TRUE	TRUE	FALSE
pLOF	16	46766333	C	A	lof	MYLK3	p.E417X	FALSE	FALSE	FALSE	FALSE
dDNV	16	46952596	G	A	misD	GPT2	p.E322K	FALSE	FALSE	FALSE	FALSE
pLOF	16	49408002	T	C	lof	C16orf78	.	FALSE	FALSE	FALSE	FALSE
pLOF	16	50745252	C	G	lof	NOD2	p.S477X	FALSE	FALSE	FALSE	FALSE
pLOF	16	67323476	TC	T	lof	KCTD19	p.Asp926fs	FALSE	FALSE	FALSE	FALSE
pLOF	16	67375953	T	A	lof	LRRC36	p.L55X	FALSE	FALSE	FALSE	FALSE
pLOF	16	67404851	G	GT	lof	LRRC36	p.L400fs	FALSE	FALSE	FALSE	FALSE

© 2023 Morton SU et al. *JAMA Network Open*.

pLOF	16	67577259	AG	A	lof	FAM65A	p.G830fs	FALSE	TRUE	FALSE	TRUE
pLOF	16	68358622	AGCAGGGT	A	lof	PRMT7	p.S57fs	TRUE	TRUE	FALSE	FALSE
pLOF	16	69752315	CA	C	lof	NQO1	p.Y43X	FALSE	FALSE	FALSE	FALSE
dDNV	16	72110496	A	C	misD	HPR	p.Asp225Ala	FALSE	FALSE	FALSE	FALSE
pLOF	16	75258741	C	T	lof	CTRB1	p.Q257X	FALSE	FALSE	FALSE	FALSE
pLOF	16	75662505	G	A	lof	KARS	p.R581X	FALSE	TRUE	FALSE	FALSE
pLOF	16	76350368	CG	C	lof	CNTNAP4	p.E24fs	FALSE	FALSE	TRUE	FALSE
pLOF	16	77317885	G	A	lof	ADAMTS18	p.Q1212X	FALSE	FALSE	FALSE	FALSE
pLOF	16	77323309	C	G	lof	ADAMTS18	NA	FALSE	FALSE	FALSE	FALSE
pLOF	16	82034394	A	C	lof	SDR42E1	.	FALSE	FALSE	FALSE	FALSE
pLOF	16	82131883	GC	G	lof	HSD17B2	p.A336fs	FALSE	FALSE	FALSE	FALSE
pLOF	16	82131883	GC	G	lof	HSD17B2	p.A336fs	FALSE	FALSE	FALSE	FALSE
pLOF	16	84485601	A	T	lof	ATP2C2	p.R579X	FALSE	FALSE	FALSE	FALSE
pLOF	16	88499538	CCCACCCTA	C	lof	ZNF469	p.T1859fs	FALSE	FALSE	FALSE	TRUE
pLOF	16	88876854	A	AG	lof	APRT	p.S100fs	FALSE	TRUE	FALSE	FALSE
pLOF	16	88904097	AG	A	lof	GALNS	p.H166fs	FALSE	FALSE	FALSE	FALSE
pLOF	16	88931528	G	T	lof	PABPN1L	p.Tyr156*	FALSE	FALSE	FALSE	FALSE
pLOF	16	89986342	A	T	lof	MC1R	p.Lys226*	FALSE	FALSE	FALSE	FALSE
pLOF	17	694801	TGCTCCGG	T	lof	RNMTL1	p.V252fs	FALSE	FALSE	FALSE	FALSE
pLOF	17	1612805	A	T	lof	TLCD2	p.C110X	FALSE	FALSE	FALSE	FALSE
pLOF	17	1612805	A	T	lof	TLCD2	p.C110X	FALSE	FALSE	FALSE	FALSE
pLOF	17	2221210	T	A	lof	SRR	p.L72X	FALSE	FALSE	FALSE	FALSE
dDNV	17	2598535	G	A	misD	CLUH	p.H823Y	FALSE	TRUE	FALSE	TRUE
pLOF	17	3343443	A	C	lof	SPATA22	p.X364E	FALSE	FALSE	FALSE	FALSE

© 2023 Morton SU et al. *JAMA Network Open*.

pLOF	17	3438930	CG	C	lof	TRPV3	p.D240fs	FALSE	FALSE	FALSE	FALSE
pLOF	17	3808158	G	T	lof	P2RX1	p.C117X	FALSE	FALSE	FALSE	FALSE
pLOF	17	4863614	C	T	lof	SPAG7	.	FALSE	TRUE	FALSE	FALSE
pLOF	17	5126610	A	G	lof	RP11-333E1.1	NA	FALSE	FALSE	FALSE	FALSE
pLOF	17	5314065	AT	A	lof	NUP88	p.I213fs	FALSE	TRUE	FALSE	FALSE
pLOF	17	10219265	CTG	C	lof	MYH13	p.Q1272fs	FALSE	FALSE	FALSE	FALSE
pLOF	17	11757318	A	G	lof	DNAH9	.	FALSE	FALSE	FALSE	FALSE
pLOF	17	11757746	G	C	lof	DNAH9	.	FALSE	FALSE	FALSE	FALSE
pLOF	17	16330045	C	T	lof	TRPV2	p.R369X	FALSE	FALSE	FALSE	FALSE
pLOF	17	19284454	CAG	C	lof	MAPK7	p.T311fs	FALSE	TRUE	FALSE	FALSE
pLOF	17	19698933	A	T	lof	ULK2	.	FALSE	FALSE	FALSE	FALSE
pLOF	17	20200305	G	A	lof	SPECC1	p.W991X	FALSE	FALSE	FALSE	FALSE
pLOF	17	28601136	T	TA	lof	BLMH	p.Y242fs	FALSE	TRUE	FALSE	FALSE
pLOF	17	33497167	C	T	lof	UNC45B	p.R528X	FALSE	FALSE	FALSE	FALSE
dDNV/pLOF	17	33592485	C	T	lof	SLFN5	p.Q752X	FALSE	FALSE	FALSE	FALSE
pLOF	17	33806192	CT	C	lof	SLFN12L	p.R346fs	FALSE	FALSE	FALSE	FALSE
pLOF	17	35830637	G	A	lof	TADA2A	NA	TRUE	TRUE	FALSE	FALSE
pLOF	17	36486756	AG	A	lof	GPR179	p.L899fs	FALSE	FALSE	FALSE	FALSE
pLOF	17	39135216	G	A	lof	KRT40	p.Q346X	FALSE	FALSE	FALSE	FALSE
pLOF	17	39183406	A	G	lof	KRTAP1-5	p.Met1?	FALSE	FALSE	FALSE	FALSE
pLOF	17	39645871	GC	G	lof	KRT36	p.G82fs	FALSE	FALSE	FALSE	FALSE
pLOF	17	39967718	G	T	lof	P3H4	p.Y150X	FALSE	FALSE	FALSE	FALSE
pLOF	17	40259709	C	CGGTGT	lof	DHX58	p.A304fs	FALSE	FALSE	FALSE	FALSE
pLOF	17	40271595	G	A	lof	KAT2A	p.Q281X	TRUE	TRUE	FALSE	TRUE
pLOF	17	41164211	C	T	lof	IFI35	p.Q13X	FALSE	FALSE	FALSE	FALSE
pLOF	17	41570095	C	T	lof	DHX8	p.R184X	FALSE	TRUE	FALSE	FALSE

© 2023 Morton SU et al. *JAMA Network Open*.

pLOF	17	46620757	CG	C	lof	HOXB2	p.Pro248fs	FALSE	FALSE	FALSE	FALSE
pLOF	17	46628464	CCCGCCGCCGCCGCCACCGCCCCCGCTGCCACCACTGCCT	C	lof	HOXB3	p.Gly90fs	FALSE	FALSE	FALSE	TRUE
pLOF	17	47014397	T	A	lof	SNF8	p.K112X	FALSE	FALSE	FALSE	FALSE
pLOF	17	47219506	C	T	lof	B4GALNT2	p.Q169X	FALSE	FALSE	FALSE	FALSE
pLOF	17	48452656	A	AC	lof	EME1	p.E29fs	FALSE	TRUE	FALSE	FALSE
dDNV	17	48761349	A	G	misD	ABCC3	p.Met1332Val	FALSE	FALSE	FALSE	FALSE
pLOF	17	49238569	G	T	lof	NME1,NME1-NME2	p.E118X	FALSE	FALSE	FALSE	FALSE
pLOF	17	52993152	C	T	lof	TOM1L1	p.R217X	FALSE	FALSE	FALSE	FALSE
pLOF	17	53026951	CAG	C	lof	TOM1L1	p.Q419fs	FALSE	FALSE	FALSE	FALSE
pLOF	17	54893116	C	G	lof	C17orf67	.	FALSE	FALSE	FALSE	FALSE
pLOF	17	56355363	G	T	lof	MPO	p.Y343X	FALSE	FALSE	FALSE	FALSE
pLOF	17	56357349	G	GAGCCGCTGCGA	lof	MPO	p.S92fs	FALSE	FALSE	FALSE	FALSE
pLOF	17	58156125	C	A	lof	HEATR6	p.E51X	FALSE	FALSE	FALSE	FALSE
dDNV	17	61557725	G	A	misD	ACE	0	FALSE	FALSE	FALSE	FALSE
dDNV/pLOF	17	61759225	A	AG	lof	MAP3K3	p.E232fs	FALSE	TRUE	FALSE	TRUE
dDNV/pLOF	17	62558993	G	A	lof	SMURF2	p.R370X	FALSE	TRUE	FALSE	TRUE
pLOF	17	66992092	C	CA	lof	ABCA9	p.G1167fs	FALSE	FALSE	FALSE	FALSE
pLOF	17	67189740	ATAAT	A	lof	ABCA10	p.I511fs	FALSE	FALSE	FALSE	FALSE

© 2023 Morton SU et al. *JAMA Network Open*.

pLOF	17	73489944	C	CCA	lof	TMEM94	p.P785fs	FALSE	FALSE	FALSE	FALSE
pLOF	17	73570751	T	TG	lof	LLGL2	.	FALSE	FALSE	FALSE	FALSE
pLOF	17	73953636	G	A	lof	ACOX1	p.R148X	FALSE	FALSE	FALSE	FALSE
pLOF	17	76566381	GGT	G	lof	DNAH17	p.Pro331fs	FALSE	FALSE	FALSE	FALSE
pLOF	17	76676428	T	C	lof	CYTH1	.	FALSE	FALSE	FALSE	TRUE
pLOF	17	76823301	G	A	lof	USP36	p.Gln239*	FALSE	FALSE	FALSE	FALSE
pLOF	17	77708889	C	G	lof	ENPP7	p.Tyr149*	FALSE	FALSE	FALSE	FALSE
pLOF	17	78064032	A	ACGTGCACGAACAACACGGGACGCGCGCG	lof	CCDC40	p.Gln976fs	FALSE	FALSE	FALSE	FALSE
pLOF	17	78286962	A	AT	lof	RNF213	p.I936fs	FALSE	FALSE	FALSE	FALSE
pLOF	17	78399347	GGA	G	lof	ENDOV	p.R214fs	FALSE	FALSE	FALSE	FALSE
pLOF	17	80010276	GGCACGGCCGGGGAC	G	lof	GPS1	p.G32fs	FALSE	TRUE	FALSE	TRUE
pLOF	18	19236850	AG	A	lof	ABHD3	p.S343fs	FALSE	FALSE	FALSE	FALSE
dDNV/pLOF	18	19751617	ACT	A	lof	GATA6	GATA6:NM_005257:exon2:c.513_514del:p.H171fs	FALSE	FALSE	FALSE	TRUE
pLOF	18	19751617	ACT	A	lof	GATA6	p.Ser172fs	FALSE	FALSE	FALSE	TRUE
dDNV	18	21110376	T	C	misD	C18orf8	p.I565T	FALSE	FALSE	FALSE	TRUE
pLOF	18	21496564	C	T	lof	LAMA3	p.Arg2610*	FALSE	FALSE	FALSE	FALSE
pLOF	18	21736416	TA	T	lof	CABYR	p.I318fs	FALSE	FALSE	FALSE	FALSE
pLOF	18	28612216	G	T	lof	DSC3	p.Cys32*	FALSE	FALSE	FALSE	FALSE
pLOF	18	28667778	T	C	lof	DSC2	.	FALSE	FALSE	FALSE	FALSE

© 2023 Morton SU et al. *JAMA Network Open*.

dDNV	18	29116237	T	A	misD	DSG2	p.L499Q	FALSE	FALSE	FALSE	FALSE
dDNV	18	32400761	C	T	misD	DTNA	p.P295S	FALSE	FALSE	FALSE	TRUE
pLOF	18	32585514	C	G	lof	MAPRE2	p.S28X	FALSE	TRUE	FALSE	TRUE
pLOF	18	32720304	C	T	lof	MAPRE2	p.Q307X	FALSE	TRUE	FALSE	TRUE
pLOF	18	33558882	GTTAC	G	lof	C18orf21	p.L193fs	FALSE	FALSE	FALSE	FALSE
pLOF	18	34359413	C	T	lof	FHOD3	p.R1597X	FALSE	TRUE	FALSE	FALSE
pLOF	18	34740228	AC	A	lof	KIAA1328	p.D433fs	FALSE	TRUE	FALSE	FALSE
dDNV/pLOF	18	45374845	C	T	lof	SMAD2	NA	FALSE	TRUE	FALSE	TRUE
pLOF	18	47432872	TC	T	lof	MYO5B	p.Gly777fs	FALSE	FALSE	FALSE	FALSE
pLOF	18	47777259	G	A	lof	CFAP53	p.Q289X	FALSE	FALSE	FALSE	FALSE
pLOF	18	53252953	A	G	lof	TCF4	NA	FALSE	TRUE	TRUE	TRUE
pLOF	18	61600279	TAGAC	T	lof	SERPINB10	p.T212fs	FALSE	FALSE	FALSE	FALSE
dDNV/pLOF	18	72347212	C	T	lof	ZNF407	p.R1413X	FALSE	FALSE	FALSE	TRUE
pLOF	18	76754984	GC	G	lof	SALL3	p.S998fs	FALSE	FALSE	FALSE	FALSE
pLOF	18	77474559	C	T	lof	CTDP1	p.Gln367*	FALSE	TRUE	FALSE	FALSE
pLOF	19	681369	C	A	lof	FSTL3	p.Ser181*	FALSE	FALSE	FALSE	TRUE
pLOF	19	871056	G	A	lof	MED16	p.Q766X	FALSE	TRUE	FALSE	FALSE
pLOF	19	1044661	AC	A	lof	ABCA7	p.Pro379fs	FALSE	FALSE	FALSE	FALSE
pLOF	19	3547905	CA	C	lof	MFSD12	p.Ala260fs	FALSE	FALSE	FALSE	FALSE
pLOF	19	3816829	TG	T	lof	ZFR2	p.Q649fs	FALSE	FALSE	FALSE	FALSE
pLOF	19	6177262	T	TC	lof	ACSBG2	p.V254fs	FALSE	FALSE	FALSE	FALSE
pLOF	19	6908699	G	A	lof	ADGRE1	.	FALSE	FALSE	FALSE	FALSE
pLOF	19	7505232	TCGCTCAAGGAGCAC	T	lof	ARHGEF18	p.S136fs	FALSE	FALSE	FALSE	FALSE
pLOF	19	7691020	A	G	lof	XAB2	.	FALSE	TRUE	FALSE	TRUE
pLOF	19	7999124	C	A	lof	TIMM44	.	FALSE	TRUE	FALSE	FALSE

© 2023 Morton SU et al. *JAMA Network Open*.

pLOF	19	9054234	C	A	lof	MUC16	.	FALSE	FALSE	FALSE	FALSE
pLOF	19	10124273	C	T	lof	RDH8	p.Q54X	FALSE	FALSE	FALSE	FALSE
pLOF	19	11215894	A	C	lof	LDLR	.	FALSE	FALSE	FALSE	FALSE
pLOF	19	11686002	TG	T	lof	ACP5	p.S267X	FALSE	FALSE	FALSE	FALSE
pLOF	19	12880697	G	A	lof	HOOK2	p.Gln368*	FALSE	FALSE	FALSE	FALSE
pLOF	19	13080596	G	A	lof	DAND5	p.W41X	FALSE	FALSE	FALSE	FALSE
pLOF	19	13135494	C	CGT	lof	NFIX	p.A16fs	FALSE	FALSE	TRUE	TRUE
pLOF	19	13882967	A	AC	lof	MRI1	p.T281fs	FALSE	FALSE	FALSE	FALSE
pLOF	19	14867079	C	T	lof	ADGRE2	p.W388X	FALSE	FALSE	FALSE	FALSE
pLOF	19	16025185	AG	A	lof	CYP4F11	p.P442fs	FALSE	FALSE	FALSE	FALSE
pLOF	19	16861144	AC	A	lof	NWD1	p.H429fs	FALSE	FALSE	FALSE	FALSE
pLOF	19	17759726	C	A	lof	UNC13A	p.E531X	FALSE	FALSE	FALSE	TRUE
pLOF	19	17831358	C	T	lof	MAP1S	p.Arg65*	FALSE	TRUE	FALSE	FALSE
pLOF	19	18284739	C	T	lof	IFI30	p.Q30X	FALSE	FALSE	FALSE	FALSE
pLOF	19	18979514	G	T	lof	GDF1	p.C337X	FALSE	FALSE	FALSE	FALSE
pLOF	19	21607218	C	G	lof	ZNF493	p.S586X	FALSE	FALSE	FALSE	FALSE
pLOF	19	35451829	G	A	lof	ZNF792	p.Gln35*	FALSE	FALSE	FALSE	FALSE
pLOF	19	35514188	G	A	lof	GRAMD1A	p.W634X	FALSE	TRUE	FALSE	FALSE
pLOF	19	36340248	CAG	C	lof	NPHS1	p.P243fs	FALSE	FALSE	FALSE	FALSE
pLOF	19	36342694	G	GCC	lof	NPHS1	p.L16fs	FALSE	FALSE	FALSE	FALSE
pLOF	19	36558868	C	T	lof	WDR62	p.Q280X	FALSE	FALSE	FALSE	FALSE
pLOF	19	36643294	C	T	lof	COX7A1	.	FALSE	FALSE	FALSE	FALSE
pLOF	19	37917191	AT	A	lof	ZNF569	p.N35fs	FALSE	FALSE	FALSE	FALSE
dDNV/pLOF	19	38853114	C	G	lof	CATSPERG	p.Y752X	FALSE	FALSE	FALSE	FALSE
pLOF	19	38853130	AT	A	lof	CATSPERG	p.I758fs	FALSE	FALSE	FALSE	FALSE
pLOF	19	38873900	G	A	lof	PSMD8	p.Trp308*	FALSE	TRUE	FALSE	FALSE

© 2023 Morton SU et al. *JAMA Network Open*.

pLOF	19	38885396	GC	G	lof	SPRED3	p.P180fs	FALSE	FALSE	FALSE	FALSE
pLOF	19	38897596	TTC	T	lof	FAM98C	p.V266fs	FALSE	FALSE	FALSE	FALSE
pLOF	19	39096371	C	T	lof	MAP4K1	.	FALSE	FALSE	FALSE	TRUE
pLOF	19	39669106	C	T	lof	PAK4	p.R555X	FALSE	TRUE	FALSE	FALSE
pLOF	19	39688691	A	G	lof	NCCRP1	.	FALSE	FALSE	FALSE	FALSE
pLOF	19	39971376	TG	T	lof	TIMM50	p.G65fs	FALSE	TRUE	FALSE	FALSE
pLOF	19	40478298	TC	T	lof	PSMC4	p.F53fs	FALSE	TRUE	FALSE	FALSE
pLOF	19	40902869	G	A	lof	PRX	p.Arg464*	FALSE	FALSE	FALSE	FALSE
dDNV	19	41928260	C	T	misD	BCKDHA	p.R280C	FALSE	FALSE	FALSE	FALSE
pLOF	19	42820662	TG	T	lof	TMEM145	p.Gly227fs	FALSE	FALSE	FALSE	FALSE
pLOF	19	44118000	G	GC	lof	SRRM5	p.S576fs	FALSE	FALSE	FALSE	FALSE
pLOF	19	45295751	T	TGCTGCCTG	lof	CBLC	p.C373fs	FALSE	FALSE	FALSE	FALSE
pLOF	19	45912350	AG	A	lof	CD3EAP	p.Q375fs	FALSE	TRUE	FALSE	FALSE
pLOF	19	46299133	ATC	A	lof	RSPH6A	p.D716fs	FALSE	FALSE	FALSE	FALSE
pLOF	19	47549896	C	CCCCGATG	lof	TMEM160	p.V86fs	FALSE	FALSE	FALSE	FALSE
pLOF	19	48950092	C	T	lof	GRWD1	p.R154X	FALSE	TRUE	FALSE	FALSE
pLOF	19	48953589	AGC	A	lof	GRWD1	p.E163fs	FALSE	TRUE	FALSE	FALSE
pLOF	19	48953589	AGC	A	lof	GRWD1	p.Glu163fs	FALSE	TRUE	FALSE	FALSE
pLOF	19	49963000	C	T	lof	ALDH16A1	p.R132X	FALSE	TRUE	FALSE	FALSE
pLOF	19	51955787	GT	G	lof	SIGLEC8	p.T449fs	FALSE	FALSE	FALSE	FALSE
pLOF	19	53304400	TG	T	lof	ZNF28	p.H233fs	FALSE	TRUE	FALSE	FALSE
pLOF	19	55175352	A	T	lof	LILRB4	p.R71X	FALSE	FALSE	FALSE	FALSE
pLOF	19	55525985	GA	G	lof	GP6	p.S443fs	FALSE	FALSE	FALSE	FALSE
pLOF	19	55823281	G	T	lof	BRSK1	p.E728X	FALSE	FALSE	FALSE	TRUE
pLOF	19	56005093	C	T	lof	SSC5D	p.R343X	FALSE	FALSE	FALSE	FALSE
pLOF	19	57835102	GA	G	lof	ZNF543	p.G24fs	FALSE	FALSE	FALSE	FALSE

© 2023 Morton SU et al. *JAMA Network Open*.

pLOF	19	57985062	G	GT	lof	ZNF772	p.Y350_K351delinsX	FALSE	FALSE	FALSE	FALSE
pLOF	20	2375100	AT	A	lof	TGM6	p.I4fs	FALSE	FALSE	FALSE	FALSE
pLOF	20	3543875	C	T	lof	ATRN	p.R551X	FALSE	FALSE	FALSE	TRUE
pLOF	20	3758910	C	CCG	lof	SPEF1	p.R220fs	FALSE	FALSE	FALSE	FALSE
pLOF	20	3758927	G	A	lof	SPEF1	p.Gln215*	FALSE	FALSE	FALSE	FALSE
pLOF	20	5283357	T	A	lof	PROKR2	p.K162X	FALSE	FALSE	FALSE	FALSE
pLOF	20	6021974	G	GT	lof	LRRN4	p.Y639_G640delinsX	FALSE	FALSE	FALSE	FALSE
pLOF	20	13763572	TA	T	lof	ESF1	p.Tyr72fs	FALSE	TRUE	FALSE	FALSE
pLOF	20	17937639	G	A	lof	SNX5	p.Q32X	FALSE	TRUE	TRUE	FALSE
pLOF	20	31494648	GTGAC	G	lof	EFCAB8	p.Thr387fs	FALSE	FALSE	FALSE	FALSE
pLOF	20	33329542	TTTAA	T	lof	NCOA6	p.I1505fs	FALSE	TRUE	FALSE	TRUE
pLOF	20	34085825	CTGTT	C	lof	CEP250	p.S1195fs	FALSE	TRUE	FALSE	FALSE
pLOF	20	34828403	G	T	lof	AAR2	p.E205X	FALSE	TRUE	FALSE	FALSE
pLOF	20	35173466	CGCTGGGTGA	C	lof	MYL9	p.S60fs	FALSE	FALSE	FALSE	FALSE
pLOF	20	36789985	CCTCT	C	lof	TGM2	p.E8fs	FALSE	FALSE	FALSE	FALSE
pLOF	20	37263370	CTG	C	lof	ARHGAP40	p.C297fs	FALSE	FALSE	FALSE	FALSE
pLOF	20	39832029	T	A	lof	ZHX3	p.K510X	FALSE	FALSE	FALSE	FALSE
pLOF	20	44640253	C	A	lof	MMP9	p.C288X	FALSE	FALSE	FALSE	FALSE
pLOF	20	47846805	GC	G	lof	DDX27	p.G348fs	FALSE	TRUE	FALSE	TRUE
dDNV/pLOF	20	52198777	C	CT	lof	ZNF217	p.A197fs	FALSE	FALSE	FALSE	TRUE
pLOF	20	52775630	AT	A	lof	CYP24A1	p.N341fs	FALSE	FALSE	FALSE	FALSE
pLOF	20	55059207	C	T	lof	RTFDC1	p.R177X	FALSE	TRUE	FALSE	FALSE
pLOF	20	57582065	T	TC	lof	CTSZ	p.D40fs	FALSE	TRUE	FALSE	FALSE

© 2023 Morton SU et al. *JAMA Network Open*.

pLOF	20	61953465	T	C	lof	COL20A1	NA	FALSE	FALSE	FALSE	FALSE
pLOF	20	62333571	T	G	lof	ARFRP1	NA	FALSE	TRUE	FALSE	FALSE
pLOF	21	15558287	A	AT	lof	LIPI	p.I200fs	FALSE	FALSE	FALSE	FALSE
pLOF	21	30415806	TAGTG	T	lof	USP16	p.S415fs	TRUE	FALSE	FALSE	FALSE
pLOF	21	30715007	TG	T	lof	BACH1	p.E689fs	FALSE	TRUE	FALSE	TRUE
pLOF	21	34997067	C	T	lof	CRYZL1	.	FALSE	FALSE	FALSE	FALSE
pLOF	21	38516934	C	CA	lof	TTC3	p.Q628fs	FALSE	TRUE	FALSE	FALSE
pLOF	21	38524331	T	C	lof	TTC3	.	FALSE	TRUE	FALSE	FALSE
pLOF	21	40800320	CTGGG	C	lof	LCA5L	p.Ser32fs	FALSE	FALSE	FALSE	FALSE
dDNV/pLOF	21	42811689	C	T	lof	MX1	p.R169X	FALSE	FALSE	FALSE	FALSE
dDNV	21	42845382	C	T	misD	TMPRSS2	p.V257M	FALSE	FALSE	FALSE	FALSE
pLOF	21	43508540	TG	T	lof	UMODL1	p.L247fs	FALSE	FALSE	FALSE	FALSE
pLOF	21	43524017	C	T	lof	UMODL1	p.R447X	FALSE	FALSE	FALSE	FALSE
dDNV/pLOF	21	47337539	CG	C	lof	PCBP3	p.P238fs	FALSE	FALSE	FALSE	TRUE
pLOF	21	47419607	G	A	lof	COL6A1	NA	FALSE	FALSE	TRUE	TRUE
pLOF	22	18210166	A	T	lof	BCL2L13	p.R442X	FALSE	TRUE	FALSE	FALSE
dDNV	22	19076893	C	T	misD	DGCR2	p.A64T	FALSE	TRUE	FALSE	FALSE
pLOF	22	25119170	G	GA	lof	PIWIL3	p.L762fs	FALSE	FALSE	FALSE	FALSE
pLOF	22	29095881	CG	C	lof	CHEK2	p.R97fs	FALSE	FALSE	FALSE	FALSE
pLOF	22	31685303	C	CT	lof	PIK3IP1	p.K195fs	FALSE	FALSE	FALSE	FALSE
dDNV/pLOF	22	36141969	C	G	lof	RBFOX2	NA	FALSE	TRUE	FALSE	TRUE
pLOF	22	36538003	AC	A	lof	APOL3	p.W80fs	FALSE	FALSE	FALSE	FALSE
pLOF	22	37398251	GAGGTGTC	G	lof	TEX33	p.D37fs	FALSE	FALSE	FALSE	FALSE
dDNV	22	37533655	C	T	misD	IL2RB	p.R170Q	FALSE	FALSE	FALSE	FALSE
pLOF	22	38151106	A	G	lof	TRIOBP	NA	FALSE	TRUE	FALSE	FALSE

© 2023 Morton SU et al. *JAMA Network Open*.

pLOF	22	38221060	GGGCCGCGCGGGGCGCGCCATGCTGGCGGT	G	lof	GALR3	p.Gly234fs	FALSE	FALSE	FALSE	FALSE
pLOF	22	39817965	C	T	lof	TAB1	p.Q304X	FALSE	FALSE	FALSE	FALSE
pLOF	22	40757290	T	TA	lof	ADSL	p.I269fs	FALSE	TRUE	TRUE	FALSE
pLOF	22	41969738	AC	A	lof	CSDC2	p.T86fs	FALSE	FALSE	FALSE	FALSE
pLOF	22	44892952	A	T	lof	LDOC1L	p.L162X	FALSE	FALSE	FALSE	FALSE
pLOF	22	45740492	TGAGA	T	lof	SMC1B	p.S1217fs	FALSE	FALSE	FALSE	FALSE
dDNV	22	46614260	G	A	misD	PPARA	p.R157Q	FALSE	FALSE	FALSE	FALSE
dDNV	22	50898382	T	C	misD	SBF1	p.S1164G	FALSE	TRUE	TRUE	TRUE
pLOF	22	50957619	GC	G	lof	NCAPH2	p.Ser246fs	FALSE	TRUE	FALSE	FALSE
pLOF	X	41419006	TG	T	lof	CASK	p.His588fs	FALSE	FALSE	TRUE	TRUE
pLOF	X	48923276	A	G	lof	CCDC120	.	FALSE	FALSE	FALSE	FALSE
pLOF	X	54783695	CA	C	lof	ITIH6	p.P937fs	FALSE	FALSE	FALSE	FALSE
pLOF	X	67938438	C	G	lof	STARD8	p.S561X	FALSE	FALSE	FALSE	FALSE
dDNV	X	78616853	G	A	misD	ITM2A	p.R226C	FALSE	TRUE	FALSE	FALSE
pLOF	X	1E+08	TG	T	lof	TRMT2B	p.His495fs	FALSE	FALSE	FALSE	FALSE
dDNV	X	1.03E+08	C	T	misD	SLC25A53	0	FALSE	FALSE	FALSE	FALSE
dDNV	X	1.07E+08	G	A	misD	VSIG1	p.V33M	FALSE	FALSE	FALSE	FALSE
pLOF	X	1.14E+08	G	A	lof	RBMXL3	p.W317X	FALSE	FALSE	FALSE	FALSE
pLOF	X	1.31E+08	G	T	lof	RAP2C-AS1	NA	FALSE	FALSE	FALSE	FALSE
pLOF	X	1.35E+08	TGATGA	T	lof	ADGRG4	p.D2504fs	FALSE	FALSE	FALSE	FALSE

© 2023 Morton SU et al. *JAMA Network Open*.

eTable 3: Neurodevelopmental tests							
**Domain**	**Measure**	**Mode**	**Age Range**	**Completed By**	**Published Mean**	**Published Standard Deviation**	**Score Direction Indicating Impairment**
Visual-Motor	**VMI-6**	Direct Testing	≥ 8 yrs	Participant	100	15	Lower
Intelligence	**WISC-V - Core Subtests:**						
	· **Similarities**	Direct Testing	< 16 yrs	Participant	100	15	Lower
	· **Vocabulary**	Direct Testing		Participant	100	15	Lower
	· **Block Design**	Direct Testing		Participant	100	15	Lower
	· **Matrix Reasoning**	Direct Testing		Participant	100	15	Lower
	· **Figure Weights**	Direct Testing		Participant	100	15	Lower
	· **Digit Span**	Direct Testing		Participant	100	15	Lower
	· **Coding**	Direct Testing		Participant	100	15	Lower
	· **Symbol Search**	Direct Testing		Participant	100	15	Lower
Intelligence	**WAIS-IV - Core Subtests:**						
	· **Block Design**	Direct Testing	≥ 16 yrs	Participant	100	15	Lower
	· **Similarities**	Direct Testing		Participant	100	15	Lower
	· **Digit Span**	Direct Testing		Participant	100	15	Lower
	· **Matrix Reasoning**	Direct Testing		Participant	100	15	Lower
	· **Vocabulary**	Direct Testing		Participant	100	15	Lower
	· **Arithmetic**	Direct Testing		Participant	100	15	Lower
	· **Symbol Search**	Direct Testing		Participant	100	15	Lower
	· **Visual Puzzles**	Direct Testing		Participant	100	15	Lower
	· **Information**	Direct Testing		Participant	100	15	Lower
	· **Coding**	Direct Testing		Participant	100	15	Lower

© 2023 Morton SU et al. *JAMA Network Open*.

Memory	**WRAML-2 Story Memory & Picture Memory Immediate Recall Tasks**	Direct Testing	≥8 yrs	Participant	100	15	Lower
Academics	**WRAT-4 Word Reading**	Direct Testing	≥8 yrs	Participant	100	15	Lower
Memory	**WRAML-2 Story Memory & Delayed Recognition Recall Tasks**	Direct Testing	≥8 yrs	Participant	100	15	Lower
Academics	**WRAT-4 Remaining Subtests**						
	**· Sentence Comprehension**	Direct Testing	≥8 yrs	Participant	100	15	Lower
	**· Spelling**	Direct Testing		Participant	100	15	Lower
	**· Math Computation**	Direct Testing		Participant	100	15	Lower
Executive Function	**D-KEFS (Selected Subtests)**						
	**· Verbal Fluency**	Direct Testing	≥ 8 yrs	Participant	10	3	Lower
	**· Trail Making**	Direct Testing		Participant	10	3	Lower
	**· Tower Subtests**	Direct Testing		Participant	10	3	Lower
Language	**WIAT-III**						
	**· Listening Comprehension**	Direct Testing	≥ 8 yrs	Participant	100	15	Lower
	**· Oral Expression Subtests Only**	Direct Testing		Participant	100	15	Lower
Social Cognition	**ADOS-2, administered if SRS-2 total score >=60 or strong likelihood of autism spectrum disorder**	Direct Testing	≥ 8 yrs	Participant	Categorical	N/A	Higher
Social Cognition	**Social Responsiveness Scale, 2nd Edition (SRS-2)**	Questionnaire	≥ 8 yrs	Parent/caregiver	Categorical	N/A	Higher
Executive Function	**BRIEF-2 Parent Report**	Questionnaire	< 18 yrs	Parent/caregiver	50	10	Higher

© 2023 Morton SU et al. *JAMA Network Open*.

Depresson	**Children's Depression Inventory, 2nd Edition (CDI-2)**	Questionnaire	< 18 yrs	Parent/caregiver	50	10	Higher
Executive Function	**Conners-3 Parent Report**	Questionnaire	< 18 yrs	Parent/caregiver	50	10	Higher
Anxiety	**MASC-2 Parent Report**	Questionnaire	< 18 yrs	Parent/caregiver	50	10	Higher
Adaptive functioning	**Vineland-3 Caregiver Rating Form****	Questionnaire	≥ 8 yrs	Parent/caregiver	100	15	Lower
Anxiety	**Beck Anxiety Inventory**	Questionnaire	≥ 18 yrs	Participant	Categorical	N/A	Higher
Depresson	**Beck Depression Inventory, 2nd Edition (BDI-2)**	Questionnaire	≥18 yrs	Participant	Categorical	N/A	Higher
Executive Function	**Conners-3**	Questionnaire	< 18 yrs	Teacher	50	10	Higher
Executive Function	**BRIEF-2**	Questionnaire	< 18 yrs	Teacher	50	10	Higher

© 2023 Morton SU et al. *JAMA Network Open*.

eTable 4: Full results for neurodevelopmental outcomes by case/control status				
**ND test**	**Scores**	**Case**	**Control**	**P-value from Mixed model**
		**(N=109)**	**(N=110)**	
**Intelligence**				
WISC-V (< 16 yrs) & WAIS-IV (>= 16 yrs)	**Processing Speed Index All Ages Standard Score**			0.71
	N	105	107	
	Min-Max	44.0-135.0	45.0-132.0	
	Mean (SD)	93.9 (17.93)	92.6 (16.36)	
	Median (Q1, Q3)	95.0 (86.0, 105.0)	94.0 (84.0, 103.0)	
	Missing	4	3	
WAIS-IV (>= 16 yrs)	**Working Memory**			0.3
	N	44	46	
	Min-Max	49.0-136.0	60.0-136.0	
	Mean (SD)	97.6 (16.65)	95.4 (15.70)	
	Median (Q1, Q3)	97.0 (92.0, 105.0)	97.0 (83.0, 105.0)	
	Missing	65	64	
**Executive Functi on**				
Conners-3 Parent (< 18 yrs)	**Executive Functioning**			0.27
	N	56	59	
	Min-Max	0.0-88.0	35.0-89.0	
	Mean (SD)	54.3 (16.00)	56.9 (14.12)	
	Median (Q1, Q3)	54.0 (45.0, 62.0)	55.0 (45.0, 65.0)	
	Missing	53	51	
Conners-3 Teacher (< 18 yrs)	**Executive Functioning**			0.1
	N	31	42	
	Min-Max	39.0-73.0	39.0-81.0	
	Mean (SD)	51.4 (9.79)	55.8 (11.51)	
	Median (Q1, Q3)	51.0 (43.0, 59.0)	53.5 (46.0, 66.0)	
	Missing	78	68	
D-KEFS (>= 8 yrs)	**Category Switching Total Accuracy**			0.24
	N	107	108	
	Min-Max	0.0-19.0	1.0-19.0	
	Mean (SD)	10.6 (3.97)	9.8 (3.59)	

© 2023 Morton SU et al. *JAMA Network Open*.

	Median (Q1, Q3)	11.0 (9.0, 13.0)	10.0 (7.0, 12.5)	
	Missing	2	2	
**Memory**				
WRAML-2 (>= 8 yrs)	**IMMEDIATE Recall: Story Memory**			0.33
	N	108	110	
	Min-Max	0.0-18.0	1.0-19.0	
	Mean (SD)	10.5 (3.49)	10.1 (3.12)	
	Median (Q1, Q3)	11.0 (8.5, 13.0)	11.0(8,12)	
	Missing	1	0	
	**IMMEDIATE Recall: Picture Memory**			0.71
	N	104	107	
	Min-Max	0.0-16.0	3.0-16.0	
	Mean (SD)	8.4 (3.14)	8.6 (3.04)	
	Median (Q1, Q3)	9.0 (6.0, 11.0)	8.0 (7.0, 11.0)	
	Missing	5	3	
**ADOS and SRS**				
Conners-3 Parent (< 18 yrs)	**Peer Relations**			0.77
	N	56	59	
	Min-Max	1.0-99.0	41.0-99.0	
	Mean (SD)	54.7 (16.60)	55.9 (16.47)	
	Median (Q1, Q3)	49.0 (44.0, 62.0)	49.0 (43.0, 62.0)	
	Missing	53	51	
Conners-3 Teacher (< 18 yrs)	**Peer Relations**			0.44
	N	32	43	
	Min-Max	2.0-99.0	41.0-99.0	
	Mean (SD)	56.4 (18.68)	53.5 (14.34)	
	Median (Q1, Q3)	51.0 (45.5, 67.5)	48.0 (43.0, 57.0)	
	Missing	77	67	
Screening classification	**Composite outcome defined by ADOS-2 and SRS-2 Total Score[1]**			0.73
	Non Spectrum	76 (91.6%)	80 (94.1%)	
	Autism/Autism Spectrum	7 (8.4%)	5 (5.9%)	
	Missing	26	25	
**Language Deficit s**				
WIAT-III (>= 8 yrs)	**Oral Language Index**			0.83
	N	91	92	
	Min-Max	39.0-141.0	62.0-140.0	

© 2023 Morton SU et al. *JAMA Network Open*.

	Mean (SD)	104.1 (19.71)	104.5 (16.18)	
	Median (Q1, Q3)	107.0 (94.0, 116.0)	105.0 (93.0, 117.0)	
	Missing	18	18	
**Adaptive Functioning**				
Vineland-3 (>= 8 yrs)	**Communication**			0.49
	N	79	82	
	Min-Max	49.0-137.0	53.0-120.0	
	Mean (SD)	100.4 (13.99)	102.0 (11.86)	
	Median (Q1, Q3)	103.0 (96.0, 109.0)	107.0 (98.0, 109.0)	
	Missing	30	28	
	**Daily Living Skills**			0.24
	N	78	82	
	Min-Max	58.0-135.0	50.0-131.0	
	Mean (SD)	102.2 (15.06)	100.2 (14.59)	
	Median (Q1, Q3)	104.0 (93.0, 116.0)	102.0 (93.0, 108.0)	
	Missing	31	28	
	**Socialization**			0.27
	N	79	82	
	Min-Max	40.0-122.0	68.0-122.0	
	Mean (SD)	99.5 (14.35)	101.8 (11.36)	
	Median (Q1, Q3)	102.0 (94.0, 110.0)	104.5 (98.0, 110.0)	
	Missing	30	28	
	**Motor Skills**			0.06
	N	17	19	
	Min-Max	81.0-118.0	62.0-111.0	
	Mean (SD)	100.8 (11.16)	93.7 (12.64)	
	Median (Q1, Q3)	102.0 (92.0, 111.0)	96.0 (83.0, 105.0)	
	Missing	92	91	
**Anxiety and Depression**				
Beck Anxiety Inventory (>= 18 yrs)	**BAI Total Raw Score Binary**[2]			-
	Low	30 (81.1%)	31 (88.6%)	
	Moderate/Potentially concerning	7 (18.9%)	4 (11.4%)	
	Missing	72	75	

© 2023 Morton SU et al. *JAMA Network Open*.

Beck Depression Inventory (>= 18 yrs)	**BDI-2 Total Raw Score Binary[3]**			-
	None/Mild	33 (89.2%)	31 (88.6%)	
	Moderate/Severe	4 (10.8%)	4 (11.4%)	
	Missing	72	75	
**ADHD/Attention**				
Conners-3 Parent (< 18 yrs)	**DSM-5 ADHD Inattentive**			0.71
	N	56	59	
	Min-Max	6.0-90.0	35.0-90.0	
	Mean (SD)	55.6 (14.98)	56.6 (14.94)	
	Median (Q1, Q3)	52.0 (45.0, 64.5)	54.0 (45.0, 68.0)	
	Missing	53	51	
	**DSM-5 ADHD Hyperactive-Impulsive**			0.4
	N	56	59	
	Min-Max	0.0-99.0	39.0-89.0	
	Mean (SD)	55.9 (17.46)	54.3 (13.81)	
	Median (Q1, Q3)	53.0 (43.5, 68.5)	50.0 (43.0, 60.0)	
	Missing	53	51	
Conners-3 Teacher (< 18 yrs)	**DSM-5 ADHD Inattentive**			0.13
	N	31	43	
	Min-Max	39.0-80.0	41.0-89.0	
	Mean (SD)	52.1 (10.78)	56.3 (13.39)	
	Median (Q1, Q3)	46.0 (44.0, 61.0)	53.0 (44.0, 62.0)	
	Missing	78	67	
	**DSM-5 ADHD Hyperactive-Impulsive**			0.92
	N	31	43	
	Min-Max	42.0-91.0	42.0-90.0	
	Mean (SD)	55.7 (14.47)	55.7 (14.85)	
	Median (Q1, Q3)	51.0 (44.0, 61.0)	49.0 (44.0, 63.0)	
	Missing	78	67	
Conners' Adult ADHD Rating Scales (>= 18 yrs)	**DSM-IV ADHD Symptoms Total**			0.24
	N	37	36	
	Min-Max	31.0-91.0	31.0-85.0	
	Mean (SD)	53.5 (16.56)	50.1 (12.65)	
	Median (Q1, Q3)	49.0 (41.0, 57.0)	48.5 (42.5, 57.5)	
	Missing	72	74	

© 2023 Morton SU et al. *JAMA Network Open*.

[1] Screening classification outcome is defined as the following: = Autism/Autism Spectrum, if “Autism” or “Autism Spectrum” were marked on ADOS form; = Non-Spectrum, if 1) “Non-Spectrum” was marked on ADOS form; or 2) no ADOS data and SRS total score < 60; = missing, if 1) neither ADOS nor SRS was done; or 2) no ADOS data and SRS total score >= 60.		
[2] BAI Total Raw Score Binary is defined as the following: 0-21: low, >=22: Moderate/Potentially concerning.		
[3] BDI-2 Total Raw Score Binary Binary is defined as the following: 0-19: None/Mild, >=20: Moderate/Severe.		
Absent p-value is because the model did not converge due to limited sample size and small event rate.		
Normative values: The normative mean score is 100 and standard deviation (SD) is 15 for all tests with these exceptions: Behavior Rating Inventory of Executive Function, Second Edition (BRIEF-2), Children’s Depression Index, Second Edition (CDI-2), Conners’ Scales, Multidimensional Anxiety Scale for Children, Second Edition (MASC-2) has mean 50, SD 10; Delis-Kaplan Executive Function System (D-KEFS) has mean 10, SD 3; Social Responsiveness Scale, Second Edition (SRS-2) reports T-score ≥ 76 for severe symptoms, 66-75 for moderate, 60-65 for mild, and < 60 typical; Beck Anxiety Inventory (BAI) and Beck Depression Inventory, Second Edition (BDI-II) score 30-63 for severe symptoms, 17-29 for moderate, 10-16 for mild, and 0-9 for minimal.		

© 2023 Morton SU et al. *JAMA Network Open*.

**eTable 5: Brain MRI findings by case/control status**			
	**Case**	**Control**	**P-value***
**MRI Findings**	**(N=53) Number(%)**	**(N=55) Number (%)**	
**Focal infarct: number**			0.56
0	48 (90.6%)	47 (85.5%)	
1-5	5 (9.4%)	8 (14.5%)	
**Focal infarct: size (out of Focal infarct number unequal 0)**			
None	48 (90.6%)	47 (85.5%)	
<1 cm	4 (7.5%)	5 (9.1%)	
1-3 cm	1 (1.9%)	2 (3.6%)	
> 3 cm		1 (1.8%)	
**Foci of low T2: number**			0.49
0	52 (98.1%)	55 (100%)	
1-5	1 (1.9%)		
**Foci of high T2: number**			0.97
0	37 (69.8%)	39 (70.9%)	
1-5	14 (26.4%)	13 (23.6%)	
6-10	2 (3.8%)	2 (3.6%)	
>20		1 (1.8%)	
**Other abnormalities missing or not**			>0.99
No	29 (54.7%)	31 (56.4%)	
Yes	24 (45.3%)	24 (43.6%)	
* Fisher's exact test p-value.			

© 2023 Morton SU et al. *JAMA Network Open*.

eTable 6: MRI differences by case/control status and post hoc *analysis*

	Case/ Control		Mixed model p-value	HBE LOF Variant		Mixed model p-value	NDD LOF Variant		Mixed model p-value	pLI LOF Variant		Mixed model p-value	Chromatin LOF Variant		Mixed model p-value
**MRI Scores**	**Case**	**Control**		**Yes**	**No**		**Yes**	**No**		**Yes**	**No**		**Yes**	**No**	
	**(N=50)**	**(N=49)**		**(N=62)**	**(N=38)**		**(N=8)**	**(N=92)**		**(N=40)**	**(N=60)**		**(N=9)**	**(N=91)**	
**Cortical Thickness**			0.33			0.61			0.71			0.85			0.87
N	47	47		59	36		8	87		38	57		8	87	
Min-Max	2.4-2.9	2.3-3.1		2.4-3.0	2.3-3.1		2.5-2.8	2.3-3.1		2.4-3.0	2.3-3.1		2.6-2.8	2.3-3.1	
Mean (SD)	2.7 (0.14)	2.7 (0.16)		2.7 (0.14)	2.6 (0.15)		2.6 (0.10)	2.7 (0.15)		2.7 (0.14)	2.7 (0.15)		2.7 (0.10)	2.6 (0.15)	
**Cortical Surface Area**			0.72			0.87			0.79			0.45			0.69
N	47	47		59	36		8	87		38	57		8	87	
Min-Max	152691-223062	142443-219342		142443-223062	148797-220943		168102-197667	142443-223062		142443-223062	152691-220943		142443-208264	147078-223062	
Mean (SD)	177002 (17183.49)	179026 (17504.49)		177256 (16488.30)	178445 (19137.49)		181490 (11727.87)	177358 (17891.93)		177740 (18496.76)	177684 (16881.60)		174739 (22436.08)	177979 (17055.88)	
**Whole Brain Volume**			0.64			0.81			0.69			0.27			0.60
N	47	47		59	36		8	87		38	57		8	87	
Min-Max	971595-1403608	926895-1393940		926895-1403608	965679-1393940		1045175-1290853	926895-1403608		926895-1403608	965679-1393940		926895-1382723	965679-1403608	
Mean (SD)	1142510 (114597.50)	1153743 (113885.57)		1141161 (106609.15)	1154673 (127705.76)		1158059 (88489.53)	1145199 (117040.24)		1152890 (119706.42)	1141876 (111893.33)		1123947 (155149.70)	1148335 (111085.07)	
**Ventricular Volume**			0.33			0.40			**0.02**			0.86			0.81

© 2023 Morton SU et al. *JAMA Network Open*.

N	47	47		59	36		**8**	**87**		38	57		8	87	
Min-Max	6361-82836	6626-40141		6361-82836	6601-30983		**9261-58017**	**6361-82836**		6361-58017	6601-82836		7565-20074	6361-82836	
Mean (SD)	16318 (13519.40)	14276 (7398.05)		15797 (12748.09)	14237 (6796.41)		**24792 (16597.92)**	**14325 (9862.87)**		14870 (9691.28)	15430 (11660.87)		14107 (5040.27)	15307 (11262.46)	
**Diffusion DTI**			0.51			0.84			**0.05**			0.65			0.10
N	45	44		55	35		**7**	**83**		37	53		8	82	
Min-Max	0.73-0.86	0.73-0.85		0.74-0.86	0.73-0.84		**0.76-0.86**	**0.73-0.84**		0.73-0.84	0.73-0.86		0.78-0.84	0.73-0.86	
Mean (SD)	0.78 (0.02)	0.78 (0.03)		0.79 (0.03)	0.78 (0.02)		**0.80 (0.04)**	**0.78 (0.02)**		0.78 (0.03)	0.78 (0.03)		0.80 (0.02)	0.78 (0.03)	
**Diffusion RSI**			0.28			0.07			0.06			0.25			0.71
N	45	45		55	35		7	83		37	53		8	82	
Min-Max	0.60-0.72	0.64-0.73		0.60-0.73	0.64-0.73		0.60-0.69	0.64-0.73		0.64-0.72	0.60-0.73		0.64-0.69	0.60-0.73	
Mean (SD)	0.68 (0.02)	0.69 (0.02)		0.68 (0.02)	0.69 (0.02)		0.67 (0.03)	0.69 (0.02)		0.68 (0.02)	0.69 (0.02)		0.67 (0.02)	0.69 (0.02)	
**RS-fMRI Gordon Network**			0.43			0.16			0.46			**0.02**			0.50
N	42	42		52	33		4	81		**34**	**51**		8	77	
Min-Max	0.12-0.48	0.11-0.42		0.12-0.48	0.11-0.42		0.15-0.28	0.11-0.48		**0.17-0.37**	**0.11-0.48**		0.22-0.34	0.11-0.48	
Mean (SD)	0.26 (0.07)	0.26 (0.06)		0.25 (0.06)	0.27 (0.07)		0.22 (0.05)	0.26 (0.06)		**0.25 (0.04)**	**0.26 (0.08)**		0.27 (0.04)	0.26 (0.07)	

© 2023 Morton SU et al. *JAMA Network Open*.

eTable 7: Neurodevelopmental outcomes in post hoc analysis													
		Presence of HBE			Presence of NDD			Presence of PLI			Presence of Chromatin		
ND test	Scores	Yes	No	P-value from Mixed model	Yes	No	P-value from Mixed model	Yes	No	P-value from Mixed model	Yes	No	P-value from Mixed model
		(N=132)	(N=86)		(N=25)	(N=193)		(N=95)	(N=123)		(N=16)	(N=202)	
Primary outcome measure: Academics													
WRAT-IV (>= 8 yrs)	Reading Composite			0.60			0.11			0.28			0.08
	N	118	74		23	169		89	103		15	177	
	Min-Max	54.0-137.0	67.0-133.0		54.0-122.0	54.0-137.0		54.0-132.0	66.0-137.0		54.0-131.0	54.0-137.0	
	Mean (SD)	102.5 (16.52)	105.1 (13.84)		97.9 (15.82)	104.3 (15.41)		102.1 (16.26)	104.7 (14.91)		95.5 (20.30)	104.2 (14.97)	
	Spelling			0.50			0.16			0.58			0.15
	N	119	73		24	168		89	103		15	177	
	Min-Max	54.0-145.0	55.0-142.0		54.0-122.0	54.0-145.0		54.0-145.0	55.0-142.0		54.0-117.0	54.0-145.0	
	Mean (SD)	102.8 (17.61)	102.7 (14.76)		98.3 (16.81)	103.4 (16.46)		103.1 (17.61)	102.4 (15.65)		95.4 (15.86)	103.4 (16.49)	
	Math Computation			0.24			0.71			0.73			0.51
	N	129	80		23	186		93	116		16	193	
	Min-Max	41.0-139.0	59.0-130.0		54.0-136.0	41.0-139.0		54.0-136.0	41.0-139.0		54.0-124.0	41.0-139.0	
	Mean (SD)	96.3 (19.26)	99.4 (16.05)		96.9 (20.62)	97.6 (17.85)		97.3 (19.36)	97.6 (17.15)		94.0 (19.89)	97.8 (18.00)	
Intelligence													

© 2023 Morton SU et al. *JAMA Network Open*.

WISC-V (< 16 yrs) & WAIS-IV (>= 16 yrs)	Full-Scale IQ All Ages Standard Score			0.18			0.49			0.52			0.13
	N	129	80		23	186		93	116		16	193	
	Min-Max	39.0-136.0	45.0-130.0		39.0-130.0	39.0-136.0		39.0-136.0	45.0-130.0		39.0-129.0	39.0-136.0	
	Mean (SD)	95.4 (18.40)	100.2 (16.49)		94.9 (20.97)	97.5 (17.42)		97.0 (19.00)	97.5 (16.88)		89.9 (20.47)	97.8 (17.49)	
	Verbal Comprehension Index All Ages Standard Score			0.24			0.26			0.26			0.01
	N	131	85		24	192		95	121		16	200	
	Min-Max	44.0-143.0	55.0-145.0		49.0-136.0	44.0-145.0		44.0-143.0	55.0-145.0		49.0-130.0	44.0-145.0	
	Mean (SD)	100.9 (18.70)	104.9 (17.38)		98.8 (19.41)	103.0 (18.11)		101.3 (19.28)	103.4 (17.43)		91.4 (20.42)	103.4 (17.83)	
WAIS-IV (>= 16 yrs)	Working Memory			0.28			0.62			0.90			0.03
	N	51	41		11	81		43	49		5	87	
	Min-Max	49.0-136.0	60.0-136.0		49.0-136.0	49.0-136.0		49.0-136.0	63.0-117.0		49.0-95.0	49.0-136.0	
	Mean (SD)	96.9 (17.12)	94.6 (15.75)		95.5 (25.76)	96.0 (15.03)		96.1 (20.76)	95.7 (11.73)		73.8 (16.39)	97.2 (15.65)	
Executive Function													
Conners-3 Teacher (< 18 yrs)	Executive Functioning			0.79			0.56			0.80			0.09
	N	51	22		8	65		33	40		9	64	
	Min-Max	39.0-74.0	39.0-81.0		40.0-73.0	39.0-81.0		39.0-71.0	39.0-81.0		49.0-71.0	39.0-81.0	

© 2023 Morton SU et al. *JAMA Network Open*.

	Mean (SD)	54.1 (11.31)	53.6 (10.36)		57.6 (12.64)	53.5 (10.76)		53.4 (10.61)	54.4 (11.36)		58.7 (7.23)	53.3 (11.27)	
BRIEF-2 Teacher (< 18 yrs)	Global Executive Composite			0.25			0.58			0.88			0.62
	N	59	30		7	82		40	49		10	79	
	Min-Max	38.0-87.0	40.0-75.0		47.0-66.0	38.0-87.0		40.0-84.0	38.0-87.0		46.0-73.0	38.0-87.0	
	Mean (SD)	55.8 (12.77)	52.8 (9.64)		55.6 (7.85)	54.7 (12.15)		54.7 (11.48)	54.9 (12.24)		56.5 (9.96)	54.6 (12.09)	
Memory													
WRAML-2 (>= 8 yrs)	IMMEDIATE Recall: Story Memory			0.16			0.38			0.03			0.24
	N	132	85		25	192		95	122		16	201	
	Min-Max	0.0-18.0	1.0-19.0		0.0-16.0	0.0-19.0		0.0-16.0	3.0-19.0		0.0-14.0	0.0-19.0	
	Mean (SD)	9.9 (3.56)	10.7 (2.96)		9.8 (3.52)	10.3 (3.34)		9.7 (3.70)	10.7 (3.01)		9.3 (4.00)	10.3 (3.30)	
	IMMEDIATE Recall: Picture Memory			0.35			0.41			0.008			0.12
	N	129	80		23	186		93	116		16	193	
	Min-Max	0.0-16.0	3.0-15.0		0.0-14.0	0.0-16.0		0.0-14.0	3.0-16.0		0.0-13.0	0.0-16.0	
	Mean (SD)	8.3 (3.23)	8.8 (2.77)		7.8 (3.45)	8.6 (3.01)		7.8 (3.13)	9.0 (2.91)		7.4 (3.65)	8.6 (3.00)	
	DELAYED Recall: Story Memory Recall			0.11			0.53			0.08			0.17
	N	132	85		25	192		95	122		16	201	
	Min-Max	0.0-18.0	1.0-19.0		0.0-16.0	0.0-19.0		0.0-16.0	1.0-19.0		0.0-15.0	0.0-19.0	
	Mean (SD)	9.9 (3.50)	10.8 (3.01)		10.0 (3.77)	10.3 (3.28)		9.8 (3.77)	10.6 (2.92)		9.3 (4.30)	10.3 (3.25)	

© 2023 Morton SU et al. *JAMA Network Open*.

	DELAYED Recall: Picture Memory Recognition			0.89			0.34			0.93			0.70
	N	129	80		23	186		93	116		16	193	
	Min-Max	0.0-19.0	5.0-14.0		0.0-16.0	0.0-19.0		0.0-19.0	1.0-18.0		0.0-19.0	0.0-18.0	
	Mean (SD)	9.3 (3.74)	9.5 (2.12)		8.8 (3.37)	9.4 (3.19)		9.4 (3.59)	9.4 (2.89)		9.2 (5.08)	9.4 (3.02)	
ADOS and SRS													
Screening classification	Composite outcome defined by ADOS-2 and SRS-2 Total Score[1]			-			0.88			0.21			0.01
	Non Spectrum	96 (88.9%)	61 (100%)		18 (90.0%)	139 (93.3%)		73 (91.3%)	84 (94.4%)		10 (71.4%)	147 (94.8%)	
	Autism/Autism Spectrum	12 (11.1%)			2 (10.0%)	10 (6.7%)		7 (8.8%)	5 (5.6%)		4 (28.6%)	8 (5.2%)	
Social Responsiveness Scale (SRS) (>= 8 yrs)	SRS-2 Total Score			0.08			0.86			0.94			0.03
	N	123	75		22	176		86	112		15	183	
	Min-Max	11.0-99.0	36.0-67.0		36.0-83.0	11.0-99.0		11.0-99.0	26.0-87.0		38.0-99.0	11.0-99.0	
	Mean (SD)	51.6 (13.43)	47.4 (8.10)		50.9 (10.84)	49.9 (12.00)		49.8 (12.91)	50.1 (11.03)		57.3 (17.22)	49.4 (11.16)	
Language Deficits													
WIAT-III (>= 8 yrs)	Listening Comprehension			0.10			0.64			0.05			0.10
	N	116	73		23	166		88	101		14	175	

© 2023 Morton SU et al. *JAMA Network Open*.

	Min-Max	39.0-133.0	61.0-139.0		39.0-128.0	39.0-139.0		39.0-133.0	65.0-139.0		39.0-126.0	39.0-139.0	
	Mean (SD)	102.0 (18.10)	106.3 (15.22)		100.7 (22.07)	104.1 (16.38)		100.7 (19.68)	106.2 (14.16)		96.6 (24.80)	104.2 (16.34)	
Adaptive Functioning													
Vineland-3 (>= 8 yrs)	Adaptive Behavior Composite			0.67			0.87			0.77			0.23
	N	102	59		19	142		75	86		11	150	
	Min-Max	56.0-125.0	69.0-124.0		75.0-125.0	56.0-124.0		56.0-125.0	60.0-124.0		56.0-118.0	58.0-125.0	
	Mean (SD)	99.8 (14.17)	102.7 (11.83)		99.8 (13.63)	101.0 (13.40)		100.6 (13.32)	101.1 (13.53)		94.0 (16.24)	101.4 (13.09)	
	Motor Skills			0.57			0.90			0.08			0.52
	N	26	10		2	34		14	22		4	32	
	Min-Max	62.0-118.0	81.0-105.0		89.0-105.0	62.0-118.0		85.0-118.0	62.0-111.0		81.0-111.0	62.0-118.0	
	Mean (SD)	97.5 (13.00)	95.7 (10.88)		97.0 (11.31)	97.0 (12.54)		101.2 (9.18)	94.4 (13.49)		102.8 (14.57)	96.3 (12.09)	
Anxiety and Depression													
CDI-2 (< 18 yrs)	Total T-Score			0.19			0.82			0.10			0.53
	N	74	38		14	98		48	64		10	102	
	Min-Max	35.0-79.0	36.0-74.0		35.0-79.0	35.0-74.0		35.0-74.0	35.0-79.0		39.0-71.0	35.0-79.0	
	Mean (SD)	49.7 (10.23)	50.3 (9.86)		51.0 (12.32)	49.8 (9.77)		48.4 (8.62)	51.1 (10.95)		52.5 (10.00)	49.7 (10.09)	

© 2023 Morton SU et al. *JAMA Network Open*.

Beck Anxiety Inventory (>= 18 yrs)	BAI Total Raw Score			0.17			0.19			>0.99			0.09
	N	41	32		8	65		37	36		4	69	
	Min-Max	0.0-64.0	0.0-56.0		0.0-64.0	0.0-64.0		0.0-64.0	0.0-56.0		5.0-64.0	0.0-64.0	
	Mean (SD)	13.4 (14.41)	8.8 (11.66)		17.8 (19.59)	10.6 (12.42)		11.9 (14.97)	10.8 (11.74)		21.3 (28.58)	10.8 (12.16)	
Beck Depression Inventory (>= 18 yrs)	BDI-2 Total Raw Score			0.18			0.13			0.61			0.07
	N	41	32		8	65		36	73		4	69	
	Min-Max	0.0-64.0	0.0-41.0		2.0-64.0	0.0-64.0		0.0-41.0	0.0-64.0		5.0-64.0	0.0-64.0	
	Mean (SD)	12.2 (14.46)	7.9 (9.05)		17.4 (20.83)	9.4 (11.01)		9.2 (10.67)	10.3 (12.50)		20.0 (29.34)	9.7 (11.02)	
ADHD/Attention													
Conners-3 Parent (< 18 yrs)	DSM-5 ADHD Hyperactive-Impulsive			0.44			0.28			0.11			0.72
	N	76	39		14	101		51	64		11	104	
	Min-Max	0.0-99.0	40.0-86.0		41.0-71.0	0.0-99.0		0.0-99.0	40.0-90.0		40.0-86.0	0.0-99.0	
	Mean (SD)	55.2 (16.71)	54.7 (13.54)		50.9 (8.46)	55.6 (16.34)		53.3 (16.32)	56.5 (15.07)		55.7 (15.25)	55.0 (15.76)	
Conners-3 Teacher (< 18 yrs)	DSM-5 ADHD Hyperactive-Impulsive			0.48			0.17			0.11			0.46
	N	51	23		66	74		33	41		9	65	

© 2023 Morton SU et al. *JAMA Network Open*.

	Min-Max	42.0-91.0	43.0-89.0		42.0-91.0	42.0-91.0		42.0-87.0	42.0-91.0		43.0-77.0	42.0-91.0	
	Mean (SD)	57.1 (15.58)	52.6 (11.85)		55.9 (14.78)	55.7 (14.59)		52.5 (11.71)	58.2 (16.26)		53.6 (11.67)	56.0 (15.00)	
Conners' Adult ADHD Rating Scales (>= 18 yrs)	DSM-IV ADHD Symptoms Total			0.007			0.45			0.59			0.43
	N	42	33		9	66		37	38		4	71	
	Min-Max	32.0-91.0	31.0-85.0		35.0-91.0	31.0-91.0		32.0-91.0	31.0-85.0		41.0-91.0	31.0-91.0	
	Mean (SD)	55.5 (15.36)	46.6 (12.29)		54.9 (20.13)	51.2 (13.93)		52.8 (15.12)	50.5 (14.38)		55.5 (23.74)	51.4 (14.25)	

*Normative values: The normative mean score is 100 and standard deviation (SD) is 15 for all tests with these exceptions: Behavior Rating Inventory of Executive Function, Second Edition (BRIEF-2)[26], Children's Depression Index, Second Edition (CDI-2), Conners' Scales, Multidimensional Anxiety Scale for Children, Second Edition (MASC-2) has mean 50, SD 10); Delis-Kaplan Executive Function System (D-KEFS) has mean 10, SD 3)[27]; Social Responsiveness Scale, Second Edition (SRS-2) reports T-score ≥ 76 for severe symptoms, 66-75 for moderate, 60-65 for mild, and < 60 typical[28]; Beck Anxiety Inventory (BAI) and Beck Depression Inventory, Second Edition (BDI-II) score 30-63 for severe symptoms, 17-29 for moderate, 10-16 for mild, and 0-9 for minimal[29,30].

© 2023 Morton SU et al. *JAMA Network Open*.

eFigure 1. Patient enrollment flow diagram.

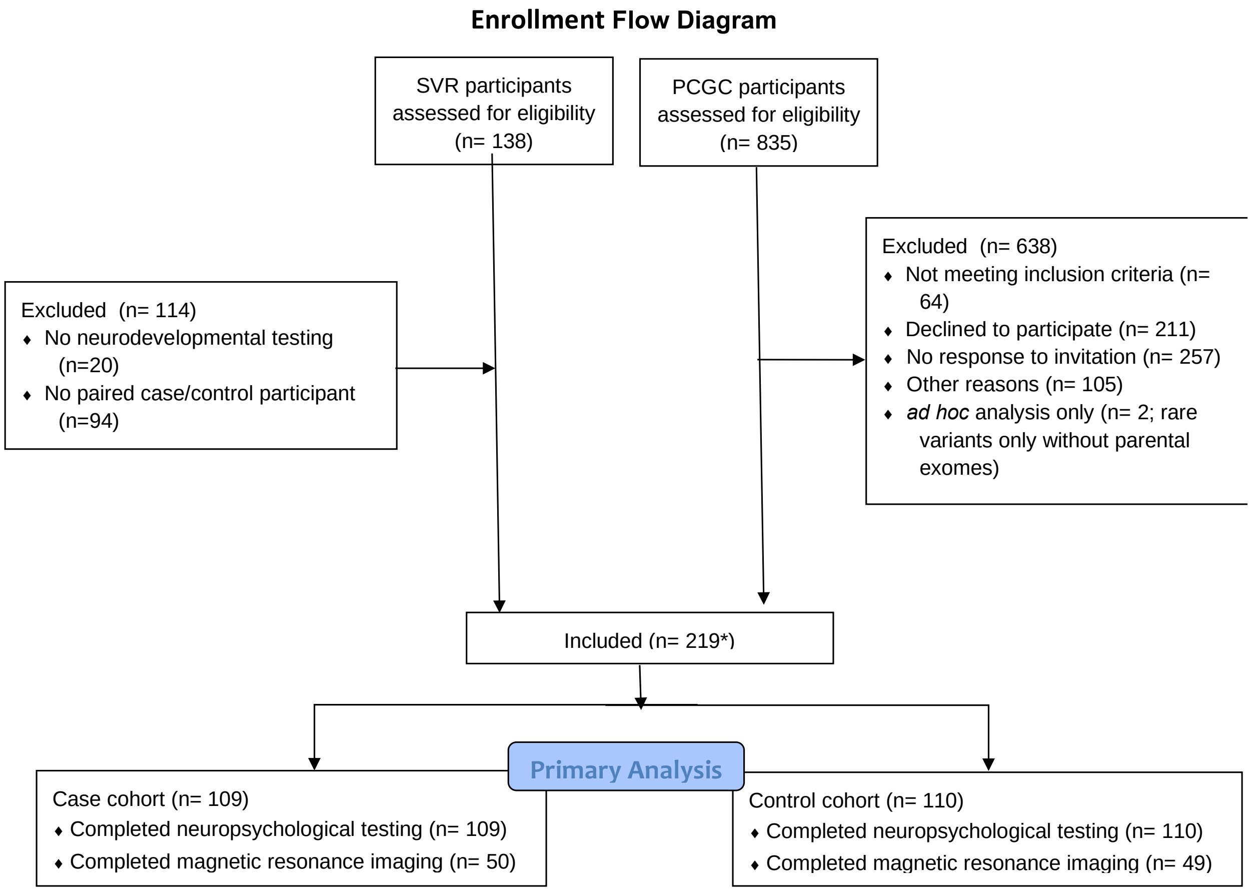

*All 211 included in *ad hoc* analysis based on rare *de novo* or inherited predicted loss-of-function variants

© 2023 Morton SU et al. *JAMA Network Open*.

eFigure 2. Venn diagram of shared genes between the four gene lists used in the *ad hoc* analysis.

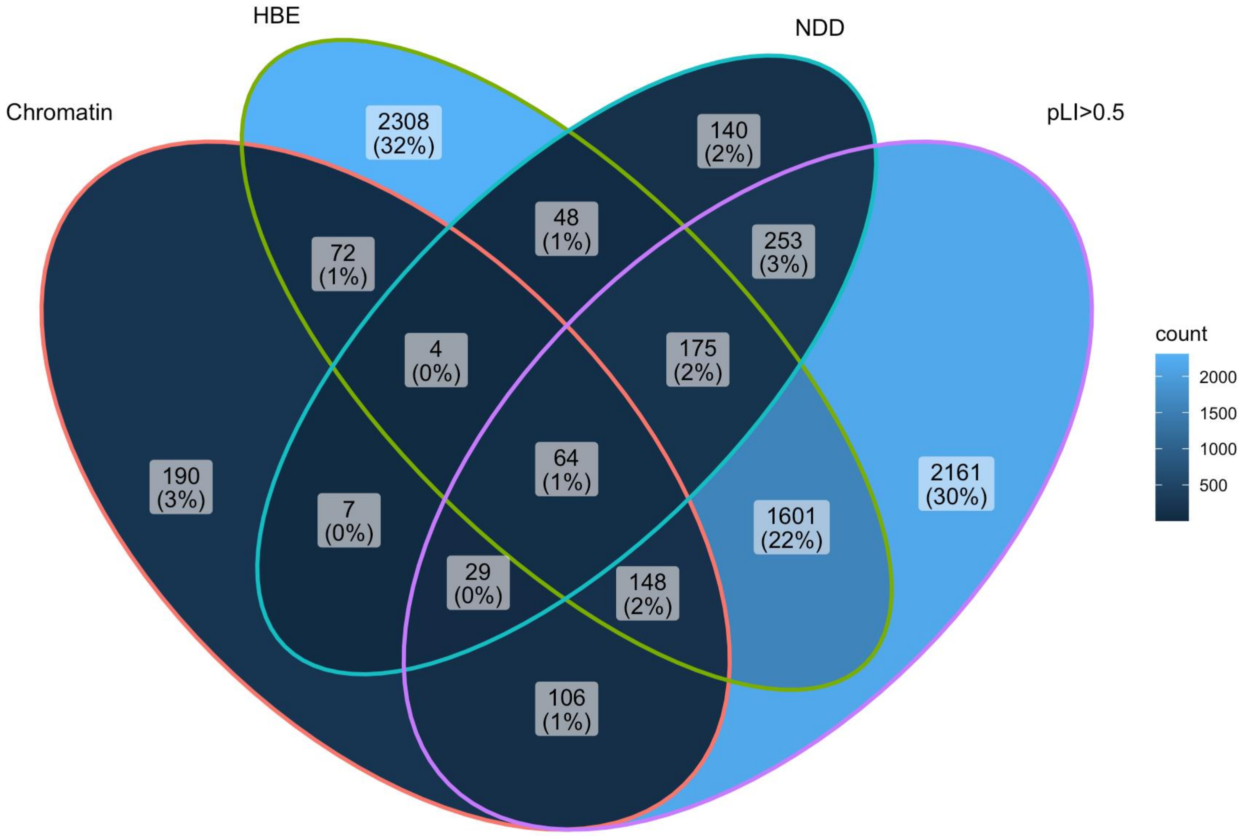

© 2023 Morton SU et al. *JAMA Network Open*.

eMethods

Study Population
Among 613 eligible PCGC participants, 197 consented to the study (97 cases, 98 controls, and 2 controls with singleton ES included only in the rare variant analysis; Supplemental Figure 1, Supplemental Table 1). SVR subjects had undergone neurodevelopmental testing and trio exome sequencing (ES) in the parent study; our analysis included 12 with dDNVs and 12 SVR controls without such variants.

Determination of Heterozygous Status
Genomic results for the PCGC participants included ES (n=188), genome sequencing (GS; n=67 who also had ES), and molecular inversion probe sequencing (MIPS; 9 participants). A dDNV-NA was defined as a *de novo* coding variant in a gene not associated with risk of NDD[1] that was predicted to be damaging based on a MetaSVM rankscore > 0.83 on available genomic analysis [2,3] (Supplemental Table 2). Case participants were selected based on the absence of dDNV-NAs. Analysis of dDNV-NAs in GS data did not change the case/control status of any participants. The nine PCGC participants classified as cases by MIPS were also studied by singleton (participant-only) exome sequencing to ensure the absence of other pathogenic variants. Two control PCGC participants had singleton exome sequencing and therefore were only included in rare variant analyses.

In *post-hoc* analysis, dDNV or rare pLOF variants from cases and controls were identified in ES data as previously described[3,4]. Each participant (case or control) was re-categorized based on presence or absence of a rare (allele frequency < $1x10^{-5}$ in gnomAD) pLOF variant in a chromatin gene (620 genes), high brain-expressed (HBE) gene (upper quartile of mouse brain expression at E14.5[2], 4420 genes), constrained gene with probability of intolerance to loss (pLI) > 0.5 in gnomAD 2.0 cohort (high pLI, 4543 genes), or gene known to be associated with risk of NDD (based on literature review through April 2017; 720 genes, Supplemental Tables 3-4). The number of participants with pLOF heterozygous status in genes from four non-mutually exclusive categories were: chromatin modifying (n = 16), HBE (n = 132), high pLI (n = 95) or NDD (n = 25). The other participants were not heterozygous in each category. As participants with a dDNV-NA in a gene known to be associated with risk of NDD at the time of the study launch were excluded from enrollment, the only NDD gene variants present in the cohort were 1) transmitted from parents, 2) single variants in genes with recessive disease mechanisms, and/or 3) not known to be associated with NDD at the time of enrollment. Twenty pLOF variants in NDD risk genes were inherited from a parent (five were single variants in NDD risk genes with recessive disease mechanism, while 15 were in genes with autosomal dominant mechanism); three were pLOF dDNV-NAs in genes with an autosomal recessive mechanism of disease (CAD, MYO5A, and SBF1); and two were pLOF dDNV-NAs in genes newly associated with NDD risk after study enrollment began (COL6A1 and TBX18).

Neuropsychological Testing
Academic Achievement: All participants completed the Wide Range Achievement Test 4 (WRAT4), a norm-referenced, examiner-administered test that measures the basic academic skills of word reading, sentence comprehension, spelling, and math computation[5]. The WRAT4 was standardized on a representative national sample of over 3,000 individuals ranging in age from 5 to 94 years. Scores have a mean of 100 and standard deviation of 15.

Intelligence: Participants under 16 years of age completed the norm-referenced, examiner-administered Wechsler Intelligence Scale for Children, Fifth Edition (WISC-V)[6]. Scores were calculated for the Full Scale Intelligent Quotient (FSIQ), Verbal Comprehension Index, Fluid Reasoning Index, and Processing Speed Index. For participants 16 years or older, the norm-referenced, examiner-administered Wechsler Adult Intelligent Scale, Fourth Edition (WAIS-IV)[7] was used. The WAIS-IV generates an FSIQ as well as Verbal Comprehension, Perceptual Reasoning, Working Memory, and Processing Speed Index scores. The mean of the WISC-V and WAIS-IV is 100, and the standard deviation is 15.

Learning and Attention: Participants, parents and teachers of all participants < 18 years old completed a Conners-3 multi-informant questionnaire measure of Attention-Deficit/Hyperactivity Disorder (ADHD) and its most common associated conditions in children and adolescents[8]. Symptom scales measure domains including, but not limited to: learning problems, DSM-V inattentive symptoms, DSM-V hyperactive-impulsive symptoms. Participants over the age of 18 years completed the Conners' Adult ADHD Rating Scales (CAARS), a self-report questionnaire. The CAARS yields scores including DSM-IV Total ADHD symptoms. Scores on the Conners-3 and CAARS domains are presented as T-scores with a mean of 50 and a standard deviation of 10.

© 2023 Morton SU et al. *JAMA Network Open*.

Executive Function: Parents and teachers of all participants < 18 years old completed the Behavior Rating Inventory of Executive Function, Second Edition (BRIEF-2)[9], which measures executive skills functioning. The Global Executive Composite score was used in the current study. Executive function was also assessed with the Conners-3 questionnaires completed by parents and teachers for participants < 18 years old[8]. BRIEF-2 and Conners-3 scores are presented as T-scores, which have a mean of 50 and a standard deviation of 10. All participants completed selected subtests of the norm-referenced, examiner-administered Delis-Kaplan Executive Function System (D-KEFS)[10], which measures overall executive functioning. The specific subtests used were the Tower test (planning and inhibition of impulsive behavior; Total Achievement Score) and Verbal Fluence-Category Switching (cognitive flexibility, set-shifting; Total Accuracy score). The mean is 10, and the standard deviation is 3.

Memory: All participants completed selected subtests of the norm-referenced, examiner-administered Wide Range Assessment of Memory and Learning, Second Edition (WRAML2)[11]. The Story Memory subtest measures the ability to remember contextual verbal information both immediately (independent recall) and after a brief (approx. 15 minute) delay (independent recall and cued recognition). The Picture Memory subtest assesses the ability to remember visual stimuli, both immediately and with delay. The mean is 100, and the standard deviation is 15.

Social Cognition: Parents administered the Social Responsiveness Scale, Second Edition (SRS-2)[12], a questionnaire. The SRS-2 measures social awareness and cognition, social communication, social motivation, and repetitive/stereotypic behavior that are characteristic of an autism spectrum disorder. The SRS-2 scale is measured via T-score, with a T score of 76 or higher indicating severe symptom presence associated with an autism spectrum disorder, T scores of 66-75 indicate a moderate symptom presence, T scores of 60-65 reflect a mild symptom presence, and a T score of 59 or lower indicates functioning that is age typical. Participants who obtained a T score at or above 60 went on to be evaluated with the examiner-administered Autism Diagnostic Observation Schedule, Second Edition (ADOS-2)[13], a test that allows for the assessment and diagnosis of autism spectrum disorders across age, developmental level, and language skills. There are four primary modules used for the age group included in the current study. Across the four modules, there is no mean or standard deviation; thus, cutoff scores are utilized to determine the potential for an autism spectrum disorder. Peer relations were also assessed with Conners-3 questionnaires completed by parents and teachers of participants < 18 years old.

Language: All participants completed the norm-referenced, examiner-administered Wechsler Individual Achievement Test, Third Edition (WIAT-III)[14], Oral Language Component; included subtests were listening comprehension, oral expression and oral language. The mean is 100, and the standard deviation is 15.

Fine Motor: All participants completed the examiner-administered Beery-Buktenica Developmental Test of Visual Motor Integration Test, Sixth Edition (BEERYBEERY VMI)[15], which measures the extent to which individuals can integrate their visual and motor abilities. The mean is 100, and the standard deviation is 15.

Adaptive Functioning: All parents of participants completed the Vineland Adaptive Behavior Scales, Third Edition (Vineland-3)[16], which measures the adaptive functioning to assess whether subjects perform their activities of daily living at an age appropriate level. Five components were included – Adaptive Behavior Composite, as well as the four indices: Communication, Daily Living, Socialization, and Motor skills. The mean of the Vineland-3 is 100, and the standard deviation is 15.

Anxiety: Parents of participants < 18 years old completed the Multidimensional Anxiety Scale for Children, Second Edition (MASC-2)[17], which measures children's level of overall anxiety as well as subscale scores. MASC-2 scores have a mean of 50 and a standard deviation of 10. For participants 18 years or older, the Beck Anxiety Inventory (BAI)[18], a self-report inventory, was administered. The BAI is measured via a cut off score. There is no mean and standard deviation, but cut-off scores are used to determine the severity of anxiety symptoms: minimal, 0-9; mild, 10-16; moderate, 17-29; severe 30-63.

Depression: Parents of participants < 18 years old completed the Children's Depression Index, Second Edition (CDI-2)[19] to measure depressive symptoms in children. The CDI-2 assesses for both emotional problems and functional problems related to depressive symptomology. The CDI-2 is reported in T-scores, with a mean of 50 and a standard deviation of 10. Participants 18 years or older completed the Beck Depression Inventory, Second Edition

© 2023 Morton SU et al. *JAMA Network Open*.

(BDI-II)[20], a self-report measure of depression. Scores on the BDI-II are tallied and compared to cut-off scores that indicate the level of severity of depression: minimal, 0-13; mild, 14-19; moderate, 20-28; severe, 29-63.

MRI Acquisition

The following is a brief description of those methods, as detailed previously. A small number of changes to the processing pipeline as described previously are indicated with footnotes. Magnetic resonance imaging was performed on either Siemens Prisma (two sites) or Prisma Fit (three sites), or General Electric MR750 (two sites). 32 channel head coils were used on all but one Prisma fit scanner, which used a 64 channel head coil. A standard scan session included sMRI series (T_1w and T_2w), one dMRI series, and four rs-fMRI series, in the following order: localizer, 3D T1-weighted images, 2 runs of resting state fMRI, diffusion weighted images, 3D T2-weighted images, and 2 runs of resting state fMRI. Scan sessions typically required ~50 minutes to complete. The T_1w acquisition (1 mm isotropic) is a 3D T_1w inversion prepared RF-spoiled gradient echo scan using prospective motion correction (Tisdall, et al., 2012; White, et al., 2010). The T_2w acquisition (1 mm isotropic) is a 3D T_2w variable flip angle fast spin echo scan, also using prospective motion correction. Prospective motion correction has not been implemented for the dMRI or fMRI acquisition. The dMRI acquisition (1.7 mm isotropic) uses multiband EPI (Moeller, et al., 2010; Setsompop, et al., 2012) with slice acceleration factor 3 and includes 96 diffusion directions, seven b=0 frames, and four b-values (6 directions with b=500 s/mm^2, 15 directions with b=1000 s/mm^2, 15 directions with b=2000 s/mm^2, and 60 directions with b=3000 s/mm^2). The fMRI acquisitions (2.4 mm isotropic, TR=800 ms) also use multiband EPI with slice acceleration factor 6. Each of the dMRI and fMRI acquisition blocks include spin-echo EPI fieldmap scans for B_0 distortion correction. The imaging protocol was developed in collaboration with each scanner manufacturer using commercially available system upgrades, and where possible, product sequences. Imaging parameters were made as similar as possible across scanner manufacturers, although some hardware and software constraints were unavoidable (for details, see Image acquisition parameters).

Siemens	Matrix	Slices	FOV	% FOV phase	Resolution (mm)	TR (ms)	TE (ms)	TI (ms)	Flip Angle (deg)	Parallel Imaging	MultiBand Acceleration	Phase partial Fourier	Diffusion Directions	b-values	Acquisition Time
T1	256 x 256	176	256 x 256	100%	1.0 x 1.0 x 1.0	2500	2.88	1060	8	2x	Off	Off	N/A	N/A	7:12
T2	256 x 256	176	256 x 256	100%	1.0 x 1.0 x 1.0	3200	565	N/A	Variable	2x	Off	Off	N/A	N/A	6:35
Diffusion	140 x 140	81	240 x 240	100%	1.7 x 1.7 x 1.7	4100	88	N/A	90	Off	3	6/8	96	500, 1000, 2000, 3000	7:31
fMRI	90 x 90	60	216 x 216	100%	2.4 x 2.4 x 2.4	800	30	N/A	52	Off	6	Off	N/A	N/A	5:00

GE	Matrix	Slices	FOV	% FOV phase	Resolution (mm)	TR (ms)	TE (ms)	TI (ms)	Flip Angle (deg)	Parallel Imaging	MultiBand Acceleration	Phase partial Fourier	Diffusion Directions	b-values	Acquisition Time
T1	256 x 256	208	256 x 256	100%	1.0 x 1.0 x 1.0	2500	2	1060	8	2x	Off	Off	N/A	N/A	6:09
T2	256 x 256	208	256 x 256	100%	1.0 x 1.0 x 1.0	3200	60	N/A	Variable	2x	Off	Off	N/A	N/A	5:50
Diffusion	140 x 140	81	240 x 240	100%	1.7 x 1.7 x 1.7	4100	81.9	N/A	90	Off	3	5.5/8	96	500, 1000, 2000, 3000	7:30
fMRI	90 x 90	60	216 x 216	100%	2.4 x 2.4 x 2.4	800	30	N/A	52	Off	6	Off	N/A	N/A	5:00

Image acquisition parameters. Scanner models: Siemens Prisma VE11B-C. GE MR759 DV25-26. For dMRI acquisition, number of diffusion directions for each b-value: 500 (6-dirs), 1000 (15-dirs) 2000 (15-dirs), 3000 (60-dirs).

Data Transfer and Initial Quality Control

Imaging data in DICOM format were packaged as tgz files, sent electronically from participating sites to the MRI Core, and automatically categorized into the different types of imaging series: T_1w, T_2w, dMRI, and rs-fMRI. Using web-based REDCap reports, MRI Core staff identified participants with missing data, and worked with sites to facilitate data transfer if needed. DICOM files were extracted from the tgz files received at the MRI Core[1] and then reviewed for problems such as incorrect acquisition parameters, imaging artifacts, or corrupted data files, using a combination of automated and manual methods. Automated protocol compliance checks providing information about the completeness of the imaging series and the adherence to the intended imaging parameters. Automated quality control procedures include the calculation of metrics such as signal-to-noise ratio (SNR) and head motion statistics. Trained technicians visually reviewed image series for poor image quality, noting various imaging artifacts and flagging unacceptable data, typically those with the most severe artifacts or irregularities. All

[1] Offline reconstruction of multiband EPI data from raw k-space files into DICOM files was performed for dMRI and fMRI data collected on a GE scanner with software version DV25, using software supplied by GE. In late 2018, GE scanners were upgraded to software version DV26, supporting online reconstruction of multiband data and providing DICOM files.

© 2023 Morton SU et al. *JAMA Network Open*.

series were consensus rated by two or more reviewers. Series rejected based on data quality criteria were excluded from subsequent processing and analysis. T1w and T2w images for each participant were reviewed by a neuroradiologist who recorded the number, approximate size, and general location of focal infarcts, number and location of foci of low or high T2w signal, and other abnormalities.

sMRI Processing and Analysis

T_1w and T_2w structural images were corrected for gradient nonlinearity distortions, and T_2w images were registered to T_1w images using mutual information (Jovicich, et al., 2006; Wald, et al., 2001). Intensity inhomogeneity correction was performed by applying smoothly varying bias fields estimated using sparse spatial smoothing and white matter segmentation, with the assumption of uniform T_1w (or T_2w) intensity values within white matter (Hagler, et al., 2019). Images were rigidly registered and resampled into alignment with an averaged reference brain in standard space. Manual quality control (QC) performed prior to full image processing was used to exclude poor-quality structural scans or select one for processing in the case of multiple acceptable scans of a given type.

Cortical surface reconstruction and subcortical segmentation were performed using FreeSurfer (Fischl, 2012) (version 5.3), a software package validated for use in children (Ghosh, et al., 2010) and used successfully in large pediatric studies (Jernigan, et al., 2016; Levman, et al., 2017). The T2w MRI volumes were not used in the cortical surface reconstruction and subcortical segmentation. Subcortical structures were labeled using automated, atlas-based, volumetric segmentation procedure (Fischl, et al., 2002). Labels for cortical gray matter and underlying white matter voxels were assigned based on cortical folding patterns and Bayesian classification rules for two standard parcellation schemes (Desikan, et al., 2006; Destrieux, et al., 2010). Fuzzy-cluster parcellations based on genetic correlation of surface area were used to calculate averages of cortical surface measures for each parcel (Chen, et al., 2012). Functionally-defined parcels based on resting-state correlations in fMRI (Gordon, et al., 2016) were resampled from atlas-space to individual subject-space and used for resting-state fMRI analysis. Morphometric measures include cortical thickness (Fischl and Dale, 2000; Rimol, et al., 2010), area (Chen, et al., 2012; Joyner, et al., 2009), volume, and sulcal depth (Fischl, et al., 1999). Image intensity measures include T1w, T2w, and T1w and T2w cortical contrast (Westlye, et al., 2009). Cortical contrast was calculated as the normalized difference of gray versus white matter, sampled at a distance of ±0.2 mm relative to the gray-white boundary.

To ensure the quality of derived measures trained technicians reviewed the accuracy of cortical surface reconstruction. For each cortical surface reconstruction reviewers gauged the severity of five categories of image artifact or reconstruction inaccuracy: motion, intensity inhomogeneity, white matter underestimation, pial overestimation, and magnetic susceptibility artifact. The reviewers assigned an overall QC score indicating whether the cortical surface reconstruction is recommended for use (1) or recommended for exclusion (0). Exclusion was recommended if any of the five categories were rated as severe.

dMRI Processing and Analysis

Preprocessing of dMRI series included corrections for eddy current distortions, head motion, B0 distortion, and gradient nonlinearity distortion. A model-based approach based on diffusion gradient orientations and amplitudes was used to correct for eddy current distortions (Zhuang, et al., 2006), with corrections limited to displacement along the phase-encode direction (Andersson and Sotiropoulos, 2016; Barnett, et al., 2014; Rohde, et al., 2004; Zhuang, et al., 2006). To correct images for head motion, we rigid-body-registered each frame to the corresponding volume synthesized from a robust tensor fit, accounting for image contrast variation between frames. Dark slices caused by abrupt head motion were replaced with values synthesized from the robust tensor fit, and the diffusion gradient matrix was adjusted for head rotation. Spatial and intensity distortions caused by B_0 field inhomogeneity were minimized using FSL's TOPUP[2], a robust and accurate tool for reducing spatial and intensity distortions in EPI images that relies on reversing phase-encode polarities (Andersson, et al., 2003; Chang and Fitzpatrick, 1992; Holland, et al., 2010; Morgan, et al., 2004). Gradient nonlinearity distortions were then corrected for each frame (Jovicich, et al., 2006). The b=0 dMRI images were registered to T1w structural images using mutual information (Wells, et al., 1996), after coarse pre-alignment via within-modality registration to atlas brains. dMRI images were then resampled in a standard orientation (relative to the T1w image rigidly registered to an atlas) with 1.7 mm isotropic resolution (equal to the dMRI acquisition resolution).

---

[2] Note that this differs from the description in Hagler et al., 2019. The previous tool for estimation of B0 distortion was replaced with FSL's TOPUP, which provides more accurate B_0 distortion correction, particularly in the presence of head motion between forward and reverse phase-encode polarity scans. This change is consistent with ABCD Release 3.0.

© 2023 Morton SU et al. *JAMA Network Open*.

Several measures related to microstructural tissue properties were calculated using Diffusion Tensor Imaging (DTI) (Basser, et al., 1994; Basser and Pierpaoli, 1996) and Restriction Spectrum Imaging (RSI) (White, et al., 2013a; White, et al., 2014; White, et al., 2013b). The DTI full shell model (DTI_{FS}) was calculated using all gradient strengths/shells (6 directions at b=500 s/mm^2, 15 directions at b=1000 s/mm^2, 15 directions at b=2000 s/mm^2, and 60 directions at b=3000 s/mm^2). The DTI inner shell (DTI_{IS}) model fit was calculated with high b-value frames excluded (leaving 6 directions at b=500 s/mm^2 and 15 directions at b=1000 s/mm^2) so that derived diffusivity measures better correspond to traditional single-b-value acquisitions. Measures derived from each DTI model included fractional anisotropy (FA), mean diffusivity (MD), logitudinal diffusivity (LD), and transverse diffusivity (TD). We use RSI to model mixtures of "restricted", "hindered", and "free" diffusion within individual voxels[3]. The signal fractions representing restricted (e.g., intracellular) and hindered (e.g., extracellular) diffusion were modeled as fourth order spherical harmonic, fiber orientation density (FOD) functions. For both fractions, the LD is modeled as 1x10^{-3} mm^2/s. For the restricted fraction, TD is modelled as 0. For the hindered fraction, TD is modelled as 0.9 x 10^{-3} mm^2/s. The free water fraction was modeled as isotropic diffusion with an apparent diffusion coefficient (ADC) of 3.0x10^{-3} mm^2/s. Measures derived from this RSI model fit included restricted normalized isotropic (RNI), restricted normalized directional (RND), restricted normalized total (RNT), hindered normalized isotropic (HNI), hindered normalized directional (HND), and hindered normalized total (HNT), and free normalized isotropic (FNI)[4]. These normalized RSI measures are unitless and range from 0 to 1.

Mean DTI and RSI measures were calculated for white matter fiber tract ROIs created with AtlasTrack(Hagler, et al., 2009; Hagler, et al., 2019), ROIs derived from FreeSurfer's automated subcortical segmentation (Fischl, et al., 2002), and cortical surface parcellations (Desikan, et al., 2006; Destrieux, et al., 2010), for cortical gray matter and white matter adjacent to the cortex (Elman, et al., 2017). Visual inspection of dMRI data after processing involved the comparison of RSI-derived RND images to corresponding co-registered T_1w images. Two reviewers rated each dMRI series based on residual B_0 distortion, registration to the T_1w image, image quality, segmentation integrity, and field of view (FOV) cutoff, and gave an overall pass-fail QC rating. Exclusion was recommended if any of the four categories were rated as severe.

fMRI Processing and Analysis

Preprocessing of fMRI series included corrections for head motion using AFNI's 3dvolreg(Cox, 1996), B0 distortion correction using FSL's TOPUP2 (Andersson, et al., 2003), and gradient nonlinearity distortion correction (Jovicich, et al., 2006). The displacement field for B_0 distortion correction was estimated from spin-echo calibration scans with reversing phase-encode polarities and applied to each gradient-echo fMRI series. Between-scan motion was corrected through image registration to a mid-session reference scan and resampling with cubic interpolation. Processed fMRI images remained in "native-space", with 2.4 mm isotropic resolution. Registration between the spin-echo B_0 calibration scans and T_1w structural images was performed using mutual information (Wells, et al., 1996), with coarse pre-alignment based on within-modality registration to atlas brains.

Measures of functional connectivity were computed using a seed-based correlational approach (Van Dijk, et al., 2010), adapted for cortical surface-based analysis (Seibert and Brewer, 2011). Additional processing steps included the removal of initial frames and normalization by the mean across time. Linear regression to remove quadratic trends, signals correlated with estimated motion time courses, and the mean time courses of cerebral white matter, ventricles, and whole brain, as well as their first derivatives (Power, et al., 2014; Satterthwaite, et al., 2012) Estimated motion time courses were temporally filtered to attenuate signals linked to respiration (Fair, et al., 2018). Frames with displacement (FD) greater than 0.3 mm were excluded from the regression (Power, et al., 2014). After regression and residualization, values for censored frames were replaced through linear interpolation[5], and time courses were band-pass filtered between 0.009 and 0.08 Hz (Hallquist, et al., 2013).

---

[3] Note that this differs from the RSI model described in Hagler et al., 2019, which included only restricted and hindered fractions. The addition of a free water fraction is consistent with the upcoming ABCD Release 4.0.

[4] Note that thes abbreviations differ from those used in Hagler et al., 2019. RNI and HNI were previously called N0 and N0_s2, respectively. RND and HND were previously called ND and ND_s2. RNT and HNT were previously called NT and NT_s2. FNI was not previously included.

[5] Note that this differs from the rs-fMRI preprocessing described in Hagler et al., 2019. Specifically, the linear interpolation step, which prevents motion contamination in the subsequent band-pass filtering, was previously omitted. The inclusion of this step is consistent with the upcoming ABCD Release 4.0.

© 2023 Morton SU et al. *JAMA Network Open*.

Preprocessed time courses were projected onto the cortical surface for each individual subject by sampling cortical gray matter voxels 1 mm from the gray/white boundary. Average time courses were calculated for cortical surface-based ROIs using FreeSurfer's anatomically-defined parcellations (Desikan, et al., 2006; Destrieux, et al., 2010) and a functionally-defined parcellation based on resting-state functional connectivity patterns (Gordon, et al., 2016), resampled from atlas-space to individual subject-space. Average time courses were also calculated for subcortical ROIs (Fischl, et al., 2002).

Variance across time was calculated for each ROI, a measure that reflects the magnitude of low frequency oscillations. Correlation values were calculated for each pair of ROIs, Fisher transformed to z-statistics, and averaged within or between networks to provide summary measures of network correlation strength (Van Dijk, et al., 2010). Average correlations within and between networks defined in the Gordon parcellation were calculated as the average of the Fisher-transformed correlations for each unique pairwise combination of ROIs belonging to each network. Average correlations were also calculated for correlation between each network and each subcortical gray matter ROI.

Motion censoring was used to reduce residual effects of head motion (Power, et al., 2012; Power, et al., 2014). Time points with FD greater than 0.2 mm were excluded from the variance and correlation calculations. Time periods with fewer than five contiguous sub-threshold time points were also excluded, and an additional round of censoring was applied based outliers with respect to spatial variation across the brain. ROI variance and network correlation values were averaged across rs-fMRI runs, weighted by the number of frames per run remaining after motion censoring. Runs with fewer than 100 usable time points (out of 375 acquired) were excluded from the average.

Visual inspection of fMRI data after processing involved the comparison to corresponding co-registered T_1w images. Two reviewers rated each participant's fMRI images, averaged across all series, based on residual B_0 distortion, registration to the T_1w image, image quality, and field of view (FOV) cutoff, and gave an overall pass-fail QC rating. Exclusion was recommended if any of the four categories were rated as severe.

REFERENCES

1. McKusick-Nathans Institute of Genetic Medicine JHU. Online Mendelian Inheritance in Man, OMIM®. https://omim.org/
2. Homsy J, Zaidi S, Shen Y, et al. De novo mutations in congenital heart disease with neurodevelopmental and other congenital anomalies. *Science (80-)*. 2015;350(6265):1262-1266. doi:10.1126/science.aac9396
3. Jin SC, Homsy J, Zaidi S, et al. Contribution of rare inherited and de novo variants in 2,871 congenital heart disease probands. *Nat Genet*. 2017;49(11):1593-1601. doi:10.1038/ng.3970
4. Morton SU, Shimamura A, Newburger PE, et al. Association of Damaging Variants in Genes with Increased Cancer Risk among Patients with Congenital Heart Disease. *JAMA Cardiol*. Published online October 21, 2020. doi:10.1001/jamacardio.2020.4947
5. Wilkinson G, Robertson G. *Wide Range Achievement Test 4 Professional Manual*. Psychological Assessment Resources; 2006.
6. Wechsler D. *WISC-V Wechsler Intelligence Scale for Children - Fifth Ed*. 5th ed. Psychological Corporation; 2014.
7. Wechsler D. *Wechsler Adult Intelligence Scale--Fourth Edition (WAIS-IV)*. Pearson; 2008.
8. Conners C. *Conners 3rd Edition (Conners 3)*. 3rd ed. Pearson Clinical; 2008.
9. Gioia G, Isquith P, Guy S, Kenworthy L. Behavior Rating Inventory of Executive Function Second Edition. In: 2nd ed. Pyschological Assessment Resources; 2015.
10. Baron IS. *Delis-Kaplan Executive Function System*. Vol 10. (Kaplan E, Kramer JH, Corporation P, eds.). Psychological Assessment Resources Inc; 2004.
11. Sheslow D, Adams W. *WRAML2 Wide Range Assessment of Memory & Learning Second Edition*. Wide Range; 2003.
12. Constantino J. *Social Responsiveness Scale, Second Edition*. Western Psychological Services; 2012.
13. Lord C, Rutter M, Dilavore P, Risi S, Gotham K, Bishop S. *Autism Diagnostic Observation Schedule, Second Edition*. Western Psychological Services; 2012.
14. Wechsler D. *Wechsler Individual Achievement Test*. Second Edi. The Psychological Corporation; 2009.
15. Beery K, Buktenica N, Beery N. *Beery-Buktenica Visual-Motor Integration Sixth Edition*. Pearson; 2010.
16. Sparrow S, Cicchetti D, Saulnier C. *Vineland Adaptive Behavior Scales Third Edition*. 3rd ed. Pearson; 2016.

© 2023 Morton SU et al. *JAMA Network Open*.

17. March JS. *Multidimensional Anxiety Scale for Children: Third Edition*. 3rd ed. Multi-Health Systems; 2013.
18. Beck A. *Beck Anxiety Inventory*. Psychological Corporation; 1993.
19. Kovacs M. *Children's Depression Inventory Second Edition*. 2nd ed. Multi-Health Systems; 2011.
20. Beck A, Steer R, Brown G. *Beck Depression Inventory Second Edition*. 2nd ed. Harcourt; 1996.

© 2023 Morton SU et al. *JAMA Network Open*.
