# Supplementary material for: Association of Potentially Damaging De Novo Gene Variants With Neurologic Outcomes in Congenital Heart Disease
Source: JAMA Netw Open. 2023 Jan 26;6(1):e2253191. doi: 10.1001/jamanetworkopen.2022.53191 (PMC9880793; doi:10.1001/jamanetworkopen.2022.53191)
Supplement: Supplement 2. — Data Sharing Statement [file jamanetwopen-e2253191-s002.pdf]

Data Sharing Statement

Morton. Association of Potentially Damaging De Novo Gene Variants With Neurologic Outcomes in Congenital Heart Disease. *JAMA Netw Open*. Published January 26, 2023. doi:10.1001/jamanetworkopen.2022.53191

Data
Data available: No

Additional Information
Explanation for why data not available: Additional information available to qualified investigators upon application to the Pediatric Cardiac Genomics Consortium.
